# A taxonomic review of the Selenophori group (Coleoptera, Carabidae, Harpalini) in the West Indies, with descriptions of new species and notes about classification and biogeography

**DOI:** 10.3897/zookeys.690.13751

**Published:** 2017-08-16

**Authors:** Danny Shpeley, Wesley Hunting, George E. Ball

**Affiliations:** 1 Department Of Biological Sciences, University Of Alberta, Edmonton, Alberta, T6g 2E9 Canada

**Keywords:** Carabidae, Harpalini, Selenophori, new genus, new species, classification, biogeography, West Indies

## Abstract

Primarily a taxonomic review of the West Indian elements of the selenophorine Harpalini, this paper includes a classification, a key, descriptions and illustrations of taxa, re-rankings, and new synonymies. In total, 45 species and subspecies are treated, six of which are described as new. A new genus and new species are as follows, with type localities in parentheses: *Paraulacoryssus* **gen. n.**, (type species *Selenophorus puertoricensis* Mutchler, 1934); *Neodiachipteryx davidsoni* **sp. n.**, (Zamba, Dominican Republic); *Selenophorus spinosus* **sp. n.**, *seriatoporus* species group (Benjamin Constant, state of Amazonas, Brazil); *Selenophorus obtusoides* **sp. n.**, *parumpunctatus* species group (near Soroa, Pinar del Rio Province, Cuba); *Selenophorus iviei* **sp. n.**, *nonseriatus* species group (Big River, Montserrat, 16°45.719N', 62°11.335W'); *Selenophorus irec* **sp. n.**, *nonseriatus* species group (Vernou, Guadeloupe, Lesser Antilles); and *Selenophorus fabricii* **sp. n.**, *opalinus* species group (Cabo Rojo, Pedernales Province, Dominican Republic). This last species was misidentified as *Selenophorus integer* (Fabricius). In turn, that species was misidentified as *Selenophorus chalybeus* Dejean. *Selenophorus chalybeus* Dejean is a junior synonym of *Selenophorus integer* Fabricius, **syn. n.**; and *Isopleurus macleayi* Kirby is a junior synonym of *Selenophorus pyritosus* Dejean, **syn. n.**

Biogeographically, log of land area plotted against log of number of species shows that the equilibrium theory of biogeography applies to the West Indian selenophorine fauna.

Taxonomically, the selenophorine taxa of the West Indies are arranged in eight genera. The 30 species/subspecies of *Selenophorus* (*sensu stricto*) are arranged in 10 species groups. Geographically, the major sources of the selenophorines are the Bahamas, the Greater Antilles and Lesser Antilles. The West Indian islands probably have been invaded by 26 taxa. Of the currently extant taxa, 11 are classified as **immigrant**, meaning that they are represented both in the islands and on the mainland (South America or Middle America and southern Florida). Thirty three taxa are classified as **precinctive**, meaning that they originated where they are now living, the implication being that they have descended from immigrants, thus older in the islands than the current-day immigrants.

It is postulated that the West Indian taxa represent three age groups: oldest, ancestors having reached the proto-Antilles by a landspan known as GAARlandia; a middle-age group (Neogene period), their ancestors having reached the islands by dispersal over water, between islands; and a young group of extant taxa, no older than the Pleistocene, also having reached the islands over water.

## Introduction

This contribution is intended to honor the memory of Philip J. Darlington, Jr. (1904–1983), and his pioneering taxonomic and biogeographical publications (1934–1953) on the Carabidae of the West Indies. It is part of a growing body of literature of similar intent and design (see Erwin and Sims 1984: 354; Noonan 1992: v–vi; and Ball and Shpeley 2009: 85). The expectation is to produce a revision of the West Indian Carabidae, and thus to fulfill DarlingtoN's (1934: 66) own plan.

Turning to specifics (meaning the Selenophori) Darlington (1934, 1935, 1937, 1939, 1941, 1947, 1953a, and 1953b) recognized a total of 28 West Indian selenophorine species and subspecies. These were arranged in three genera: *Stenomorphus* Dejean, *Gynandropus* LeConte and *Selenophorus* Dejean. Subsequent study by Lindroth (1968: 823) affected the arrangement of West Indian selenophorines by recognizing three species groups originally proposed by Casey (1914) of *Selenophorus* (*opalinus*, *palliatus*, and *ellipticus*), and *Discoderus* LeConte. Noonan (1985a, b) introduced the new West Indian genera *Neoaulacoryssus and Neodiachipteryx*, and the previously described *Amblygnathus* Dejean. Noonan also synonomized *Gynandropus* with *Selenophorus*.

Ball and Shpeley (1992) accepted the changes proposed by Noonan. They added 12 (seven new) selenophorine taxa to those recognized by Darlington, giving a total of 39 species and subspecies. Those authors also transferred three species from *Selenophorus* to *Discoderus*. The descriptions of new taxa were perfunctory, and required additional attention to make them maximally useful. Here, we provide the required detailed treatment, including illustrations of structural features, a key, and maps showing known distribution of the taxa in the West Indies. In the process of doing this work, we discovered new species, and a new genus, and can now propose a detailed classification.

## Material, methods, and terms

### Material

This study is based on examination of 27,471 specimens of the Selenophori group. Some of the material was available in the Strickland Museum, Department of Biological Sciences, University of Alberta (UASM). Additional material was borrowed from, or deposited in, the following institutions and private collections, noted in the text by the associated codens. Names of owners or curators are included, in parentheses.

**AMNH** Department of Entomology, American Museum of Natural History, Central Park West at 79th Street, New York, New York, U.S.A. 10024 (L. H. Herman)

**BDVC** Barry D. Valentine Collection, 5704 Lake Breeze Court, Sarasota, Florida, U.S.A. 34233-5015

**BMNH** Department of Entomology, British Museum (Natural History), Cromwell Road, London, England SW7 5BD (M. J. D. Brendell, S. J. Hine, B. Garner)

**BPBM** Bernice P. Bishop Museum, Department of Entomology, 1355 Kalihi St., P.O. Box 1900-A, Honolulu, Hawaii, U.S.A. 96819 (G. Allan Samuelson)

**CASC** Department of Entomology, California Academy of Sciences, Golden Gate Park, San Francisco, California, U.S.A. 94118 (D. H. Kavanaugh)

**CMNC** Canadian Museum of Nature, P.O. Box 3443 Stn. D, Ottawa, Ontario, Canada K1P 6P4 (R. S. Anderson, S. B. Peck)

**CMNH** Section of Entomology, Carnegie Museum of Natural History, 4400 Forbes Avenue, Pittsburgh, Pennsylvania, U.S.A. 15213 (R. L. Davidson, J. E. Rawlins)

**CNCI** Canadian National Collection, Agriculture and Agri-Food Canada, K. W. Neatby Building, Ottawa, Ontario, Canada K1A 0C6 (Y. Bousquet)

**CUIC** Department of Entomology, Comstock Hall, Cornell University, Ithaca, New York, U.S.A. 14850 (J. K. Liebherr)

**DEFW** Department of Entomology, Fisheries and Wildlife Collection, University of Minnesota, St. Paul, Minnesota, U.S.A. 55101 (P. J. Clausen)

**DRMC** David R. Maddison Collection, Department of Integrative Biology, 3029 Cordley Hall, Oregon State University, Corvallis, Oregon, U.S.A. 97331

**FSCA** Florida State Collection of Arthropods, Division of Plant Industry, Florida Department of Agriculture, Gainesville, Florida, U.S.A. 32601 (R. E. Woodruff)

**HNHM** Hungarian Natural History Museum, Zoological Department, Baross utca, 13 H- 1088, Budapest, Hungary (O. Merkl)

**INHS** Illinois State Natural History Survey, Urbana, Illinois, U.S.A. 61803 (C. Grinter)

**IJSM** Natural History Museum, Institute of Jamaica, 12-16 East Street, Kingston, Jamaica (T. Farr, deceased)

**IREC** Institut de Recherches Entomologique de la Caribe, B.P. 119, Pointe-a-Pitre, Guadeloupe (Fortune Chalumeau)

**IRSB** Institut Royal des Sciences Naturelle de Belgique, Rue Vautier 29, B-1000, Bruxelles, Belgique (G. Demoulin)

**IZAC** Instituto de Zoologia, Academia de Ciencias de Cuba, Capitolio Nacional, La Habana 2, Ciudad de la Habana 10200, Cuba (Lic. Luis F. de Armas C.)

**JMLC** Jean-Michel Lemaire Collection, 2162 chemin du Destey, F-06390 Contes, France.

**JMPR** Julio Micheli (deceased), 14 Baldorioty St.-Mariani, Ponce, Puerto Rico, U.S.A. 00731

**MBCN** Michiel Boeken Collection, Dillestraat 42, 2034 MR Haarlem, The Netherlands

**MCSN** Museo Civico di Storia Naturale “Giacomo Doria”, via Brigata Liguria 9, I-16121 Genoa, Italy (R. Poggi)

**MCZC** Department of Entomology, Museum of Comparative Zoology, Harvard University, Cambridge, Massachusetts, U.S.A. 02138 (P. D. Perkins, B. D. Farrell)

**MEMU** Mississippi Entomological Museum, P.O. Box 9775, Mississippi State, Mississippi, U.S.A. 39762-9775 (T. L. Schiefer)

**MNHC** Museo Nacional de Historia Natural, Capitolio Nacional, La Habana 2, Ciudad de La Habana 10200, Cuba (Luis R. Hernández)

**MNHP** Entomologie, Muséum National d’Histoire Naturelle, 45 Rue Buffon, Paris, 75005, France (T. Deuve)

**OSUC** Department of Entomology, Ohio State University, 1735 Neil Avenue, Columbus, Ohio, U.S.A. 43210 (C. A. Triplehorn, N. Johnson)

**PVRC** Pavel Valdez Ruiz Collection, Havana, Cuba.

**RHTC** Robert H. Turnbow, Directorate of Engineering and Housing, Building 1404, Fort Rucker, Alabama, U.S.A. 36362-5137

**RLDC** Robert L. Davidson Collection, Section of Entomology, Carnegie Museum of Natural History, 4400 Forbes Avenue, Pittsburgh, Pennsylvania, U.S.A. 15213

**SEMC** Snow Entomological Museum, University of Kansas, Lawrence, Kansas, U.S.A. 66044 (A. E. Short)

**UMMZ** Division of Insects, Museum of Zoology, University of Michigan, Ann Arbor, Michigan. U.S.A. 48109-1079 (M. F. O’Brien)

**USNM** Department of Entomology, United States National Museum of Natural History, Smithsonian Institution, Washington, D. C., U.S.A. 20560 (T. L. Erwin, W. Steiner)

**WIBF** West Indian Beetle Fauna Project, Department of Entomology, Montana State University, Bozeman, Montana, U.S.A. 59717 (M. A. Ivie)

**ZMAN** Instituut voor Taxonomische Zoologie, Zoologisch Museum, Universiteit van Amsterdam, Plantage Middenlaan 64, 1018 DH Amsterdam, The Netherlands (J. P. Duffels)

**ZMUC** Department of Entomology, Zoological Museum, University of Copenhagen, Universitetsparken, DK-2100 Copenhagen, Denmark (N. Møller Anderson (deceased), A. Solidovnikov)

### Methods

Taxonomic concepts, principles, criteria for ranking, and general working methods were the same as those described previously (Ball 1975, 1978; Allen and Ball 1980; Ball and Shpeley (2002 and 2009).

**Measurements.** Measurements were made with an ocular micrometer in a Wild M5 stereoscopic microscope, at 12×, 25×, and 50×. Measurements of external body parts and abbreviations used for them in the text are:

Length of head (**HL**) linear distance from base of left mandible to posterior margin of left eye;

Length of pronotum (**PL**) linear distance from anterior to posterior margin, measured along the midline;

Length of elytra (**EL**) linear distance from basal ridge to apex of longer elytron (if the pair of elytra is asymmetrical), measured along the suture.

Standardized Body Length (**SBL**), used as an index of overall size, is the sum of HL, PL, and EL. Values for length (more or less diagnostic for species groups or species) were computed (Table 1), using the measurements above. We determined the central point of each range (median) to identify central tendency. We treat these morphometric data as illustrative rather than definitive.

**Table 1.** Variation in Standardized Body Length (SBL, in mm) among the West Indian species and subspecies of the Selenophori group

	Males			Females		
	N	Range	Mean	N	Range	Mean
*Neoaulacoryssus*						
*N. cupripennis*	1	13.32		2	12.52–13.20	12.86
*Paraulacoryssus*						
*P. puertoricensis*	2	8.72–9.00	8.86	6	9.56–10.12	9.79
*Athrostictus*						
*A. paganus*	**11**	**7.08–7.56**	**7.29**	**8**	**7.00–7.72**	**7.49**
Barbados	5	7.08–7.56	7.33	2	7.00–7.72	7.36
Martinique	2	7.04–7.20	7.12	2	7.24–7.60	7.42
St. Croix	4	7.08–7.40	7.24	4	7.28–7.72	7.56
*Amblygnathus*						
*A. cephalotes* ^1^	2	9.48–9.68	9.58	2	10.36–10.60	10.48
*A. puncticollis*	**7**	**5.00–5.64**	**5.37**	**3**	**5.08–5.60**	**5.37**
Jamaica	3	5.12–5.52	5.35	2	5.08–5.44	5.26
Domin. Repub.	4	5.00–5.64	5.38	1	5.60	
*A. gilvipes gilvipes* ^2^	3	5.38–5.57	5.47	2	5.28–5.54	5.41
*Neodiachipteryx*						
*N. davidsoni*	1	8.12				
*N. cariniger*	5	8.40–9.36	8.86	2	8.28–8.36	8.32
Selenophorus (Celiamorphus)						
*discopunctatus* species group						
*S. discopunctatus*	**20**	**5.92–6.88**	**6.57**	**20**	**6.16–7.28**	**6.81**
Cuba	10	6.48–6.88	6.74	10	6.16–7.08	6.71
Guadeloupe	10	5.92–6.80	6.39	10	6.60–7.28	6.91
*S. yucatanus*	10	6.12–7.00	6.60	10	6.48–7.28	6.81
*latior* species group						
S. *barbadensis*	4	4.93–5.32	5.11	7	5.09–5.60	5.38
*S. latior*	**11**	**5.04–5.84**	**5.46**	**16**	**4.88–5.68**	**5.31**
Virgin Islands	5	5.28–5.84	5.51	10	5.04–5.68	5.35
Guadeloupe	6	5.04–5.72	5.42	6	4.88–5.48	5.23
*S. solitarius*	1	5.04		1	5.04	
*seriatoporus* species group						
*S. spinosus*	1	7.88				
Selenophorus (Selenophorus)						
*hylacis* species group						
S. *clypealis*	1	4.88		1	5.12	
*S. dessalinesi*	4	6.01–7.07	6.68	3	6.62–6.94	6.79
*S. dubius*				1	5.78	
S. *parvus*	10	3.76–4.12	3.95	10	3.76–4.48	4.18
*S. subquadratus*	**16**	**5.12–5.84**	**5.59**	**12**	**5.16–5.68**	**5.64**
Domin. Repub.	10	5.12–5.76	5.51	4	5.16–5.68	5.39
St. Kitts	6	5.60–5.84	5.71	8	5.52–6.16	5.77
*mundus* species group						
*S. mundus*	10	3.96–4.60	4.32	10	3.96–4.88	4.64
*S. paramundus*				1	5.32	
*S. pseudomundus*	4	3.60–4.00	3.76	6	3.82–4.32	4.09
*nonseriatus* species group						
*S. irec*				2	4.64–4.76	4.70
*S. iviei*	9	4.00–4.92	4.64	7	4.24–5.28	4.66
*S. nonseriatus*	10	4.00–4.88	4.60	10	4.32–5.32	4.76
*opalinus* species group						
*S. fabricii*	**20**	**8.64–9.60**	**9.14**	**20**	**8.36–9.40**	**9.01**
Cuba	10	8.88–9.60	9.25	10	8.64–9.40	9.13
Swan Island	10	8.64–9.28	9.03	10	8.36–9.28	8.88
*S. flavilabris cubanus*	10	6.08–7.08	6.70	10	6.32–7.36	6.98
*S. flavilabris flavilabris*	**13**	**6.72–8.44**	**7.49**	**16**	**7.08–8.64**	**7.86**
Puerto Rico	10	6.72–7.56	7.35	10	7.08–8.00	7.58
St. Martin	3	7.64–8.44	7.96	6	8.00–8.64	8.31
*S. flavilabris ubancus*	10	6.84–7.56	7.21	10	6.88–7.96	7.35
*S. integer*	14	8.72–9.40	9.06	19	8.60–9.52	9.05
St. Croix	10	8.80–9.40	9.10	10	8.60–9.52	9.18
Guadeloupe	4	8.72–9.16	8.96	9	8.68–9.32	8.91
*S. opalinus*	1	8.52				
*S. propinquus*	**20**	**7.20–8.36**	**7.74**	**20**	**6.80–8.44**	**7.71**
Jamaica	10	7.20–8.36	7.93	10	7.36–8.44	7.97
Guadeloupe	10	7.40–7.96	7.60	10	6.80–8.20	7.44
*palliatus* species group						
*S. alternans*	**20**	**6.12–6.92**	**6.55**	**20**	**6.40–7.32**	**6.84**
Domin. Rep.	10	6.12–6.84	6.47	10	6.48–7.12	6.89
St. Croix	10	6.24–6.92	6.62	10	6.40–7.32	6.79
*S. palliatus*	10	6.20–7.64	7.05	10	6.28–8.08	7.62
*S. pyritosus*	**12**	**6.92–8.60**	**8.14**	**12**	**7.12–9.12**	**8.17**
Cuba	10	6.92–8.60	8.14	10	7.12–8.96	8.08
Jamaica	2	7.72–8.52	8.12	2	8.16–9.12	8.64
*S. woodruffi*	10	6.92–7.84	7.47	10	7.12–7.92	7.40
*parumpunctatus* species group						
*S. obtusoides*	1	4.28				
*S. parumpunctatus*	**20**	**4.76–5.40**	**5.04**	**20**	**4.68–5.84**	**5.28**
Cuba	10	4.76–5.12	4.87	10	4.68–5.28	5.05
Desirade	10	5.04–5.40	5.20	10	5.28–5.84	5.51
*striatopunctatus* species group						
*S. striatopunctatus*	**20**	**5.20–6.04**	**5.74**	**20**	**5.28–6.24**	**5.90**
Cuba	10	5.44–6.04	5.78	10	5.28–6.24	5.87
Puerto Rico	10	5.20–5.96	5.69	10	5.68–6.20	5.92
*Stenomorphus*						
*S. californicus manni*	3	10.93–12.33	11.84	3	9.62–10.93	10.11
*S. cubanus* ^3^	1	8.97		1	11.21	
*Discoderus*						
*D. beauvoisi*	**20**	**6.32–7.56**	**7.00**	**20**	**6.32–7.44**	**6.85**
Cuba	10	6.32–7.36	6.88	10	6.36–7.44	6.95
Puerto Rico	10	6.88–7.56	7.12	10	6.32–7.08	6.75
*D. cinctus*	7	6.56–7.88	7.43	10	7.12–7.68	7.40
*D. cyaneopacus*	2	10.20–10.32	10.26	9	8.72–9.96	9.96
*D. thoracicus*	10	5.92–6.80	6.58	10	6.00–6.84	6.40

^1^ Data for French Guiana specimens from Ball GE, Maddison DR (1987) ^2^ Data for Surinam specimens from Ball GE, Maddison DR (1987) ^3^ Data from Ball GE, Shpeley D, Currie DC (1991)

**Preparation of material.** Dissections were made by using standard techniques. Genitalia and other small structures were preserved in glycerine in microvials, pinned beneath the specimens from which they were removed. Larger structures and those that were gold-coated for study with the SEM were glued to cards pinned beneath the specimens from which they were removed.

Photographs of isolated structures were taken with a JEOL JSM 6301 FXV field emission SEM. Line drawings of selected body parts were prepared by using a camera lucida on a Wild W5 stereoscopic microscope. Stacks of images were taken using a Nikon CoolPix 8400 digital camera mounted to an Olympus SZX16 stereomicroscope. The stacked images were then rendered into a single image using Helicon Focus 5.3.7. All specimens, regardless of luster or color, were imaged using the same identical conditions. A piece of mylar drafting film was shaped into a cylinder to surround the specimen. Four fibre optic wands were then shone on the mylar film, two at angles toward the head, and two at angles toward the elytral apex. Final plates were prepared using Adobe Photoshop CS4.

### Terms

Terms used in this publication are either in common usage, or have been defined in previous publications, such as Lindroth (1974), Deuve (1993), Liebherr and Will (1998) and Ball and Shpeley (2002 and 2009). The only new term relates to the male genitalia: “phallic” is added to “median lobe” to ensure that the meaning of the latter is established.

## Systematic zoology

### Order Coleoptera Linnaeus, 1758

#### Family Carabidae Latreille, 1802

##### Tribe Harpalini Bonelli, 1810

###### Selenophori

Taxon classificationAnimaliaColeopteraCarabidae

####### Classification.

For the general ranking and arrangement of the West Indian Selenophori, we accept that proposed by Noonan (1985a, b), with treatment of the taxa of *Amblygnathus* Dejean as proposed by Ball and Maddison (1987), and treatment of *Stenomorphus* Dejean as proposed by Ball et al. (1991).

The classification of *Selenophorus* Dejean is based principally on details of the male genitalia, with sequence of species groups being alphabetical. Like the selenophorine groups, the members are arranged alphabetically according to species name.

Because of the remarkable structure of the female genital tract and its similarity to that of *Neoaulacoryssus* Noonan, we place *Selenophorus puertoricensis* Mutchler in a new monobasic genus named *Paraulacoryssus* gen. n., following *Neoaulacoryssus* in a linear arrangement.

####### Diagnosis.

Noonan (1985a: 4–8) discussed the definition and composition of the New World Selenophori group, and included a detailed description of adult selenophorines. In this paper we limit the West Indian Selenophori group to harpaline adults that have seta bearing punctures in striae 2 and 5 or in striae 2, 5 and 7 which includes the following genera: *Neoaulacoryssus* Noonan, *Paraulacoryssus* gen. n., *Athrostictus* Bates, *Amblygnathus* Dejean, *Neodiachipteryx* Noonan, *Selenophorus* Dejean, *Stenomorphus* Dejean and *Discoderus* LeConte.

####### Way of life.

Information available in the form of label data about this topic is limited, as shown by number of species (Table 2) and number of specimens per species (Table 3). Basically, selenophorines are geophilous and lowland. Collectively, they occupy habitats ranging from swamps to desert and from fresh water to brackish tidal flats. They are night-active, adults of most species being macropterous, many being taken by light traps.

**Table 2.** Label data for West Indies collection of selenophorine species with number of species collected at each type of site.

**Habitat**	**No. spp.**	**Habitat, etc.**	**No. spp.**
swamp/marsh community	2	brackish tidal flats/salt marsh	5
wet deciduous forest	1	grassland	1
riparian growth/ thorn for.	1		
riparian woodland	7	night beating	2
mesic lowland forest	8		
pine forest	5	under cow dung	4
forest leaf litter	3		
semi arid lowland w/pastures	6	u- v /m- v trap	19
dry seasonal/dry deciduous forest	5		
semi-arid /arid thorn scrub	11	Elev. data (sea level- 3000m)	20
desert scrub	1		

**Table 3.** Number of specimens of Selenophori species in West Indies with habitats or collection methods or elevations known, and total number of specimens per species.

	X	XX = total
*Athrostictus paganus*	2	96
*Amblygnathus puncticollis*	7	19
*Neodiachipteryx cariniger*	1	1
*Selenophorus discopunctatus*	14	1,398
*Selenophorus clypealis*	2	6
*Selenophorus subquadratus*	2	65
*Selenophorus parvus*	2	5,451
*Selenophorus mundus*	4	58
*Selenophorus pseudomundus*	1	41
*Selenophorus nonseriatus*	17	182
*Selenophorus iviei*	1	42
*Selenophorus fabricii*	19	162
*Selenophorus flavilabris flavilabris*	4	77
*Selenophorus flavilabris cubanus*	5	175
*Selenophorus flavilabris ubancus*	15	798
*Selenophorus integer*	7	1,626
*Selenophorus propinquus*	13	677
*Selenophorus alternans*	3	90
*Selenophorus pyritosus*	4	1,201
*Selenophorus woodruffi*	1	135
*Selenophorus parumpunctatus*	8	9,779
*Selenophorus striatopunctatus*	12	1,731
*Discoderus beauvoisii*	15	1,874
*Discoderus cinctus*	1	83
*Discoderus cyaneopacus*	1	17
*Discoderus thoracicus*	4	225

####### Key to genera and species of West Indian Selenophori Group

**Table:** 

01	Body elongate, narrow, cylindrical (habitus, Fig. 62). Elytron with punctures only in striae 2 and 5	***Stenomorphus* Dejean**...**02**
01'	Body not elongate, various in form. Elytron with punctures in striae 2, 5 and 7	**03**
02 (01)	Middle femur anteroventrally obtusely angulate or sinuate near apex. Geographical range: Cuba	***S. cubanus* Darlington**, p. 116
02'	Middle femur anteroventrally angulate or more-or-less sharply dentate near apex. Geographical range: Hispaniola (habitus, Fig. 62)	***S. californicus manni* Darlington**, p. 113
03 (01')	Elytron with dorsal surface densely punctate, each puncture round, with seta shorter than those of striae 2, 5 and 7. Habitus, Fig. 1C. Geographical range: Lesser Antilles	***Athrostictus* Bates**...***A. paganus* (Dejean)**, p. 24
03'	Elytron with dorsal surface impunctate (except the standard setigerous punctures in striae 2, 5 and 7), or intervals catenate, with elongate punctures. Geographical range in West Indies various	**04**
04 (03')	Elytron with dorsal surface with interconnected chains of punctures. Dorsal surface generally coppery (habitus, Fig. 1A). Geographical range: Lesser Antilles, Windward Islands – Mustique, in the Grenadines	***Neoaulacoryssus* Noonan**...***N. cupripennis* (Gory)**, p. 18
04'	Elytron with dorsal surface smooth, glabrous, except few setigerous punctures in each of striae 2, 5 and 7	**05**
05 (04')	Elytron with prominent ridge bordered laterally by preapical fused portion of striae 7 and 8. Dorsal surface of head, pronotum and elytra greenish iridescent (habitus, Fig. 9), microlines evident only in irregularly distributed isolated spaces. Geographical range: Hispaniola	***Neodiachipteryx* Noonan**...**06**
05'	Elytron preapically without prominent ridge, normally declivous. Color and surface various. Geographical range in the West Indies various	**07**
06 (05)	Labrum with anterior margin deeply notched medially. Elytron with interval 2 markedly convex at apex, intervals 3–5 moderately convex at apex (Fig. 10B)	***N. davidsoni* sp. n.**, p. 36
06'	Labrum with anterior margin shallowly concave, not notched. Elytron with interval 2 slightly convex, intervals 3–5 flat, as on elytral disc (Fig. 10A)	***N. cariniger* (Putzeys)**, p. 33
07 (05')	Front tibia with lateral margin near apex with row of three or four stout spines. Pronotum with posteriolateral angles more or less broadly rounded. Elytron dorsally with mesh pattern isodiametric. Habitus Fig. 66A–D. Geographical range: Bahamas and Greater Antilles	***Discoderus* LeConte**...**08**
07'	Front tibia with lateral margin with not more than two spines. Posteriolateral angles of pronotum various. Geographical range: Greater and Lesser Antilles and Bahamas	**11**
08 (07)	Pronotum rufous. Elytra bicolored: intervals 2–5 piceous or piceous with faintly metallic green luster; intervals 1 and 6–8 rufous	**09**
08'	Pronotum metallic blue or green. Elytra metallic blue (like pronotum), green or bronze	**10**
09 (08)	Pronotum with lateral margins and posteriolateral angles broadly rounded. Habitus, Fig. 66B. Geographical range: Cuba	***D. cinctus* (Putzeys)**, p. 120
09'	Pronotum with lateral margins narrowly rounded; posteriolateral angles narrowly rounded in females, in males angles projected posteriorly, posterior margin slightly excised laterally. Habitus, Fig. 66D. Geographical range: Hispaniola	***D. thoracicus* (Putzeys)**, p. 124
10 (08')	Labrum with anterior margin deeply emarginate. Clypeus with anterior margin angularly emarginated, less deeply so than the labrum. Habitus, Fig. 66C. Geographical range: Hispaniola	***D. cyaneopacus* (Darlington)**, p. 121
10'	Labrum with anterior margin subtruncate. Clypeus with anterior margin shallowly concave. Habitus, Fig. 66A. Geographical range: Bahamas and islands of the Greater Antilles	***D. beauvoisii* (Dejean)**, p. 117
11 (07')	Head large (Fig. 5A–C); clypeus with anterior margin concave, basal membrane of labrum exposed medially. Labrum with anterior margin broadly notched. Elytra iridescent	***Amblygnathus* Dejean**...**12**
11'	Head average; clypeus and labrum as above in few individuals, most with anterior margins subtruncate or slightly concave. Elytra iridescent or not	**14**
12 (11)	Legs black. Habitus, Fig. 5A. Geographical range: Lesser Antilles, Windward Islands, Guadeloupe	***A. cephalotes* Dejean**, p. 27
12'	Legs testaceous or flavous. Geographical range: Greater and Lesser Antilles	**13**
13 (12')	Pronotum with posteriolateral angles subangulate. Habitus, Fig. 5C. Geographical range: Guadeloupe	***A. g. gilvipes* Ball & Maddison**, p. 32
13'	Pronotum with posteriolateral angles rounded. Habitus, Fig. 5 B. Geographical range: Greater Antilles	***A. puncticollis* (Putzeys)**, p. 29
14 (11')	Pterothorax with lateral margin of metepisternum only slightly longer than wide at anterior margin, specimen without membranous flight wing. Habitus, Fig. 1B. Geographical range: Puerto Rico		***Paraulacoryssus*, gen. n.**...***P. puertoricensis* (Mutchler)**, p. 22
14'	Pterothorax with lateral margin of metepisternum distinctly longer than wide at anterior margin, specimen with membranous flight wing. Geographical range: West Indies	***Selenophorus* Dejean**...**15**
15 (14')	Elytron with striae 1–7 distinctly punctate, in addition to the serial punctures in striae 2, 5 and 7. Habitus, Fig. 58. Geographical range: Greater Antilles and Lesser Antilles, St. Lucia, Windward Islands	***S. striatopunctatus* species group**...***S. striatopunctatus* Putzeys**
15'	Elytron with striae 1–7 impunctate, except for serial punctures in striae 2, 5 and 7 (interruptions in the striae may appear as punctures)	**16**
16 (15')	Elytron with preapical notch on lateral margin	***S. parumpunctatus* species group**...**17**
16'	Elytron with preapical margin laterally hardly or not sinuate	**18**
17 (16)	Pronotum markedly narrow posteriorly, posteriolateral angles broadly rounded. Elytron with the standard setigerous punctures of striae 2, 5 and 7 not foveate. Habitus, Fig. 54B. Geographical range: throughout West Indies	***S. parumpunctatus* Dejean**, p. 106
17'	Pronotum markedly narrowed posteriorly, posteriolateral angles angulate, obtuse. Elytron with the standard setigerous punctures of striae 2, 5 and 7 markedly foveate. Habitus, Fig. 54A. Geographical range: Cuba	***S. obtusoides* sp. n.**, p. 104
18 (16')	Ventral surface of basitarsus of hind tarsus with inner spines forming a single contiguous row of spines	***S. hylacis* species group**...**19**
18'	Ventral surface of basitarsus of hind tarsus with inner spines not forming a single contiguous row of spines	**23**
19 (18)	Elytra distinctly bicolored, rufo-testaceous with dark discal cloud. Habitus, Fig. 25C	***S. dubius* Putzeys**, p. 59
19'	Elytra unicolorous, rufo-piceous to piceous	**20**
20 (19')	Pronotum subcordate, posteriolateral angles nearly rectangular, prominent. Habitus, Fig. 25B. Geographical range: Hispaniola	***S. dessalinesi* Ball and Shpeley**, p. 55
20'	Pronotum not subcordate, posteriolateral angles obtuse to rounded	**21**
21 (20')	Hind angles of pronotum broadly rounded. Habitus, Fig. 25A. Geographical range: Hispaniola	***S. clypealis* Ball and Shpeley**, p. 55
21'	Hind angles of pronotum obtuse, not broadly rounded	**22**
22 (21')	Pronotum with posteriolateral area coarsely punctate. Mesh pattern of elytra with sculpticells slightly transverse, about 2 to 3 times wide as long; elytra not iridescent. Habitus, Fig. 26. Geographical range: Greater and Lesser Antilles	***S. subquadratus* (Putzeys)**, p. 63
22'	Pronotum with posteriolateral area impunctate. Mesh pattern of elytra with sculpticells moderately transverse; elytra faintly iridescent. Habitus, Fig. 25D. Geographical range: Puerto Rico and Lesser Antilles	***S. parvus* Darlington**, p. 60
23 (18')	Elytron with dorsal surface shining or matte, lacking iridescence; microlines evident at 100×; mesh pattern isodiametric to slightly transverse	**24**
23'	Elytron with dorsal surface slightly to markedly iridescent; microlines evident or not at 100×; mesh pattern slightly to markedly transverse	**30**
24 (23)	Pronotum with posteriolateral angles broadly rounded. Hind tarsus with tarsomeres long and slender, length about the same as length of hind tibia	**25**
24'	Pronotum with posteriolateral angles rectangular or slightly rounded. Hind tarsus with tarsomeres short, length about 2/3 length of hind tibia	***S. palliatus* species group**...**27**
25 (24)	Pronotum wider, posteriolateral impressions impunctate, or with only a few small punctures. Habitus, Fig. 22. Geographical range: Windward Islands of Lesser Antilles	***S. seriatoporus* species group**...***S. spinosus*, new species**, p. 52
25'	Pronotum narrower, with posteriolateral impressions and adjoining areas moderately to densely punctate	***S. discopunctatus* species group**...**26**
26 (25')	Pronotum with posteriolateral impressions moderately punctate, but not rugose. Habitus, Fig. 14A. Geographical range: throughout West Indies	***S. discopunctatus* Dejean**, p. 39
26'	Pronotum with posteriolateral impression and adjacent areas densely punctate, rugose. Habitus, Fig. 14B. Geographical range: Windward Islands of Lesser Antilles	***S. yucatanus* Putzeys**, p. 42
27 (24')	Pronotum with posteriolateral angles narrowly rounded. Habitus, Fig. 49B. Geographical range: Bahama Islands	***S. palliatus* (Fabricius)**, p. 96
27'	Pronotum with posteriolateral angles rectangular	**28**
28 (27')	Elytron with apical portion and disc concolorous, or apical area narrowly and slightly paler. Body size larger, SBL 6.92–8.60 mm. Habitus, Fig. 49C. Geographical range: Bahamas, Cayman Brac and Greater Antillean islands of Jamaica, Cuba and Hispaniola	***S. pyritosus* Dejean**, p. 97
28'	Elytron with apical area distinctly paler than discal area	**29**
29 (28')	Elytron with apical area and preapical part of suture distinctly paler than disc; intervals without punctures basally. Habitus, Fig. 49A. Geographical range: Lesser and Greater Antilles (not recorded from Jamaica) and Bahama Islands	***S. alternans* Dejean**, p. 95
29'	Elytron with apical area distinctly paler than disc and intervals 6 and 7 slightly paler than disc; intervals with fine punctures basally. Habitus, Fig. 49D. Geographical range: Lesser Antillean islands of Grenada and Mayreau	***S. woodruffi* Ball and Shpeley**, p. 101
30 (23')	Smaller in size, SBL 3.60–5.84 mm	**31**
30'	Larger in size, SBL 6.72–10.12 mm	***S. opalinus* species group**...**39**
31 (30)	Elytron without parascutellar stria	***S. nonseriatus* species group**...**32**
31'	Elytron with parascutellar stria	**34**
32 (31)	Posterior margin of pronotum beaded only laterally. Habitus, Fig. 35A. Geographical range: Lesser Antilles – Guadeloupe	***S. irec* sp. n.**, p. 71
32'	Posterior margin of pronotum not beaded	**33**
33 (32')	Pronotum unicolorous, lateral bead same color as disc. Elytral striae wider apically than on disc, 2–3 sculpticells wide. Habitus, Fig. 35C. Geographical range: Greater Antillean islands of Jamaica and Hispaniola	***S. nonseriatus* Darlington**, p. 77
33'	Pronotum bicolored, lateral bead paler than disc. Elytral striae same width apically as on disc, 1 sculpticell wide. Habitus, Fig. 35B. Geographical range: Lesser Antillean islands of Montserrat, St. Lucia, St. Vincent and Grenada	***S. iviei*, sp n.**, p. 72
34 (31')	Dorsum with no visible microlines at 100×; moderate green and blue metallic luster. Pronotum with posteriolateral impressions impunctate. Habitus, Fig. 31B. Geographical range: Jamaica	***S. mundus* species group**, in part...***S. paramundus* Ball and Shpeley**, p. 67
34'	Combination of characters not as above	**35**
35 (34')	Smaller in size, SBL 3.60–4.88 mm	***S. mundus* species group**, in part...**36**
35'	Larger in size, SBL 4.93–5.84 mm	***S. latior* species group**...**37**
36 (35)	Head with microlines effaced on frons and vertex, not visible at 100×, surface very shiny. Habitus, Fig. 31C. Geographical range: Hispaniola	***S. pseudomundus* Ball and Shpeley**, p. 69
36'	Head with microlines distinct, meshes isodiametric, visible at 100×, surface shiny. Habitus, Fig. 31A. Geographical range: Hispaniola	***S. mundus* Putzeys**, p. 65
37 (35')	Pronotum with broad base, lateral margin little rounded, posteriolateral impressions impunctate. Habitus, Fig. 18B. Geographical range: Greater Antilles— Hispaniola, Puerto Rico, and Virgin Islands; and Lesser Antilles— Guadeloupe, St. Lucia and Grenada	***S. latior* Darlington**, p. 48
37'	Pronotum with narrow base, lateral margins more rounded, posteriolateral impressions punctate	**38**
38 (37')	Pronotum with posteriolateral angles broadly rounded, posteriolateral impressions coarsely punctate. Habitus, Fig. 18C. Geographical range: Cuba	***S. solitarius* Darlington**, p. 50
38'	Pronotum with posteriolateral angles rounded, posteriolateral impressions finely punctate. Habitus, Fig. 18A. Geographical range: Windward Islands, Lesser Antilles	***S. barbadensis* Ball and Shpeley**, p. 45
39 (30')	Pronotum impunctate	**40**
39'	Pronotum with fine punctures at least in posteriolateral impressions	**42**
40 (39)	Dorsal surface dark, elytron with transverse mesh, microlines evident at 100×. Habitus, Fig. 39C. Geographical range: Puerto Rico and Virgin Islands	***S. flavilabris flavilabris* Dejean**, p. 85
40'	Dorsal surface bright metallic green, microlines not evident at 100×. Geographical range: Bahamas and Greater Antilles, except Puerto Rico	**41**
41 (40')	Legs bicolored, femora infuscated, tibiae and tarsi testaceous. Habitus, Fig. 39D. Geographical range: Bahamas, Hispaniola, Jamaica and Cayman Islands	***S. flavilabris ubancus* Ball and Shpeley**, p. 84
41'	Legs unicolorous, testaceous. Habitus, Fig. 39B. Geographical range: Cuba and Andros Island in the Bahamas	***S. flavilabris cubanus* Darlington**, p. 84
42 (39')	Size smaller, SBL 6.8–8.4 mm. Tibiae darkened apically. Habitus, Fig. 40C. Geographical range: Greater and Lesser Antilles	***S. propinquus* Putzeys**, p. 94
42'	Size larger, SBL 8.6–9.6 mm. Tibiae unicolorous	**43**
43 (42')	Elytron with striae widened preapically, each about half width of adjacent portions of intervals. Habitus, Fig. 39A. Geographical range: Bahamas and Greater Antilles	***S. fabricii* sp n.**, p. 79
43'	Elytron with striae only slightly widened preapically, each less than half width of adjacent intervals	**44**
44 (43')	Femora infuscated. Habitus, Fig. 40A. Geographical range: Lesser Antilles and Greater Antilles to the Dominican Republic	***S. integer* (Fabricius)**, p. 87
44'	Femora testaceous. Habitus, Fig. 40B. Geographical range: Bahamas	***S. opalinus* LeConte**, p. 91

###### Genus Neoaulacoryssus

Taxon classificationAnimaliaColeopteraCarabidae

Noonan

Neoaulacoryssus Noonan, (1985a: 37). TYPE SPECIES: Selenophorus speciosus Dejean, 1829: 117–118 (designation by Noonan 1985a: 37).— Lorenz 1998: 355.— Lorenz 2005: 376.

####### Recognition.

Both *Neoaulacoryssus* and *Athrostictus* are the only New World selenophorine genera whose species have pubescence on the elytral disc. In *Neoaulacoryssus* the elytral punctures are elongated, in places confluent and chain-like, with extremely short pubescence, length approximately half or less the width of the elongated punctures. In *Athrostictus*, the elytral punctures are round with long pubescence, length approximately 3 or more times the width of the round punctures.

####### Included species.

Two species known; only one, *N. cupripennis* (Gory), is recorded in the West Indies.

###### Neoaulacoryssus cupripennis

Taxon classificationAnimaliaColeopteraCarabidae

(Gory)

**Figure 1.**
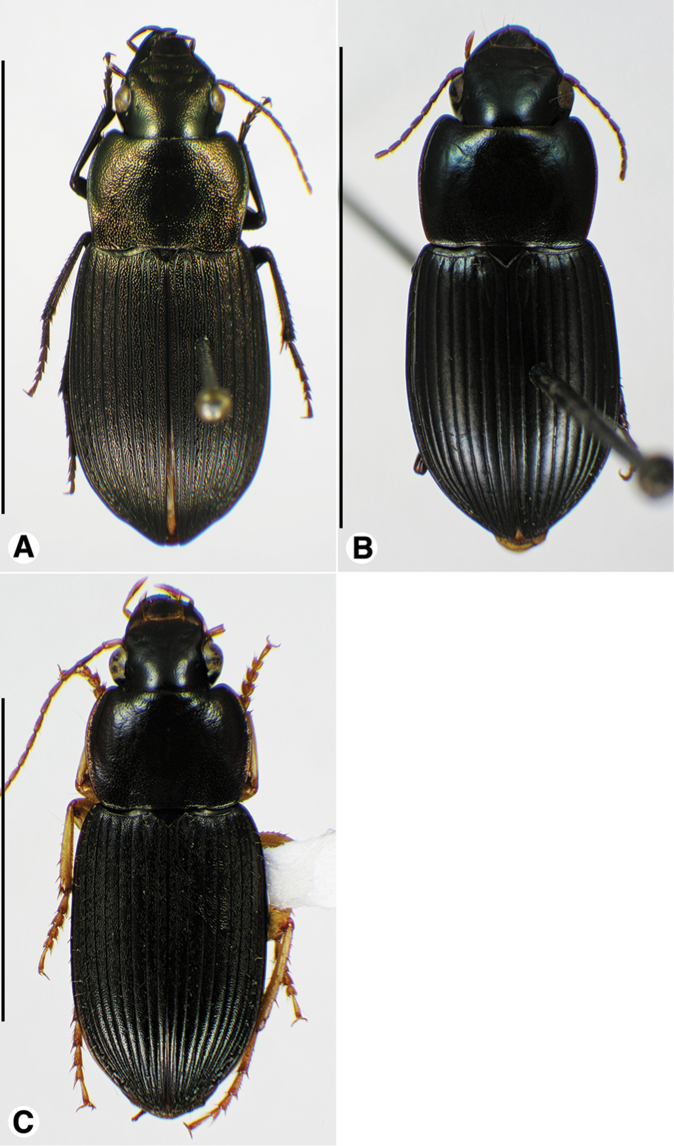
Habitus digital images, dorsal aspect. **A** *Neoaulacoryssus cupripennis* (Gory) **B** *Paraulacoryssus puertoricensis* (Mutchler) **C** *Athrostictus paganus* (Dejean). Scale bars: **A** 15 mm; **B** 10 mm; **C** 5 mm.

**Figure 2.**
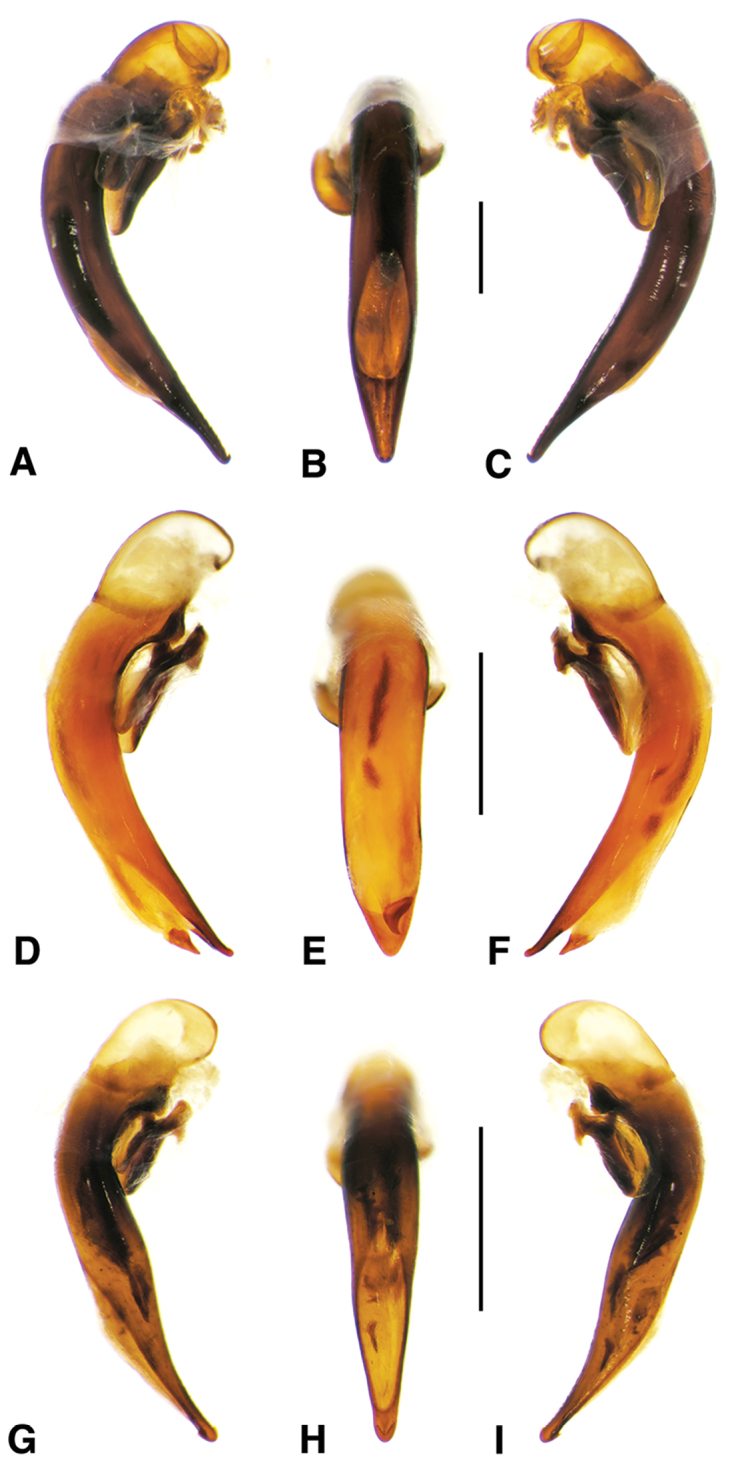
Digital images of male genitalia. **A, D, G** right lateral aspect **B, E, H** dorsal aspect **C, F, I** left lateral aspect **A–C** *Neoaulacoryssus cupripennis* (Gory) **D–F** *Paraulacoryssus puertoricensis* (Mutchler) **G–I** *Athrostictus paganus* (Dejean). Scale bars 1 mm.

**Figure 3.**
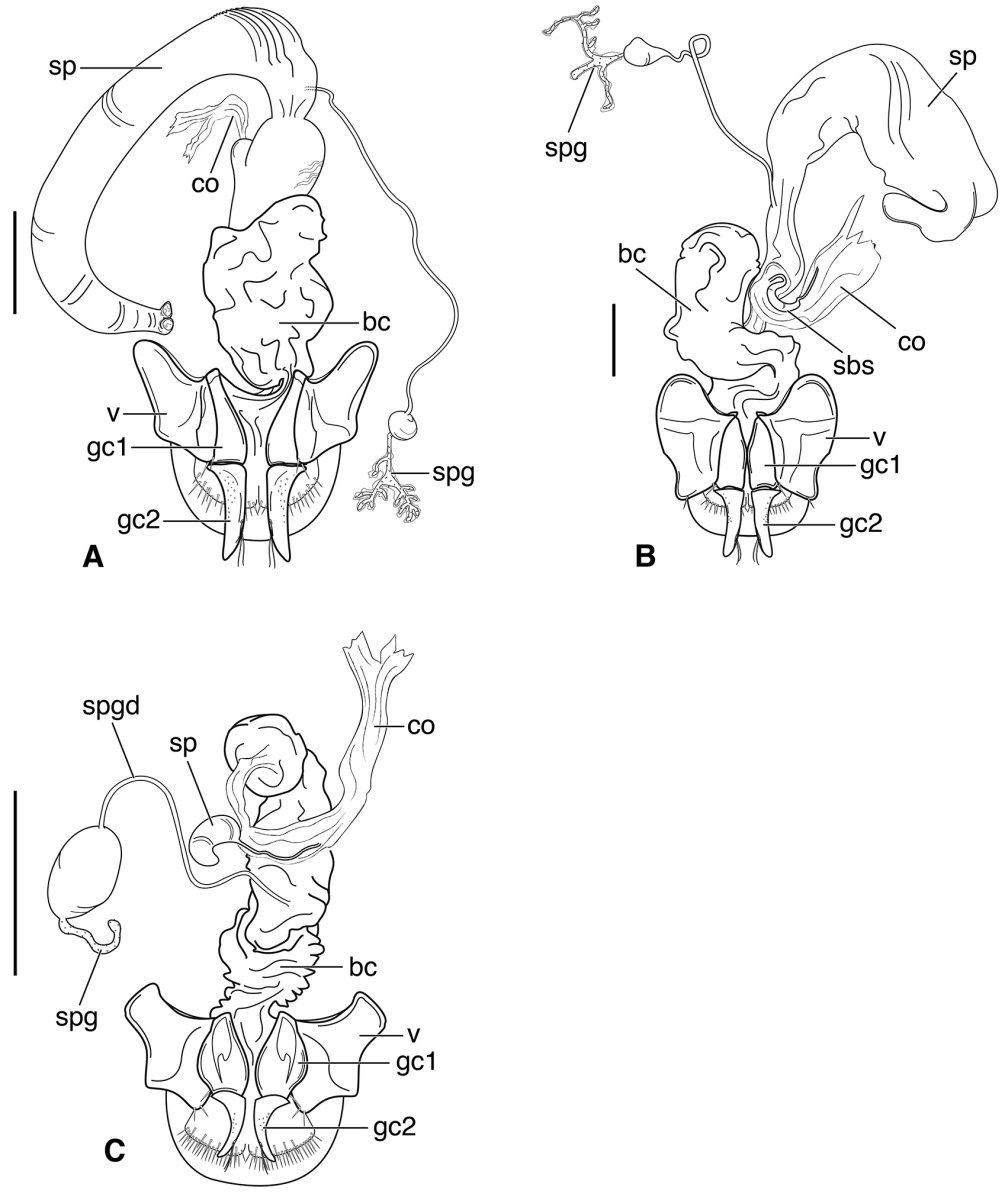
Line drawings of female reproductive tract, ventral aspect. **A** *Neoaulacoryssus speciosus* (Dejean) **B** *Paraulacoryssus puertoricensis* (Mutchler) **C** *Athrostictus paganus* (Dejean). Legend: **bc** bursa copulatrix; **co** common oviduct; **gc1** gonocoxite 1; **gc2** gonocoxite 2; **sbs** spermathecal basal sclerite; **sp** spermatheca; **spg** spermathecal gland; **spgd** spermathecal gland duct; **v** valvifer. Scale bars 1 mm.

**Figure 4.**
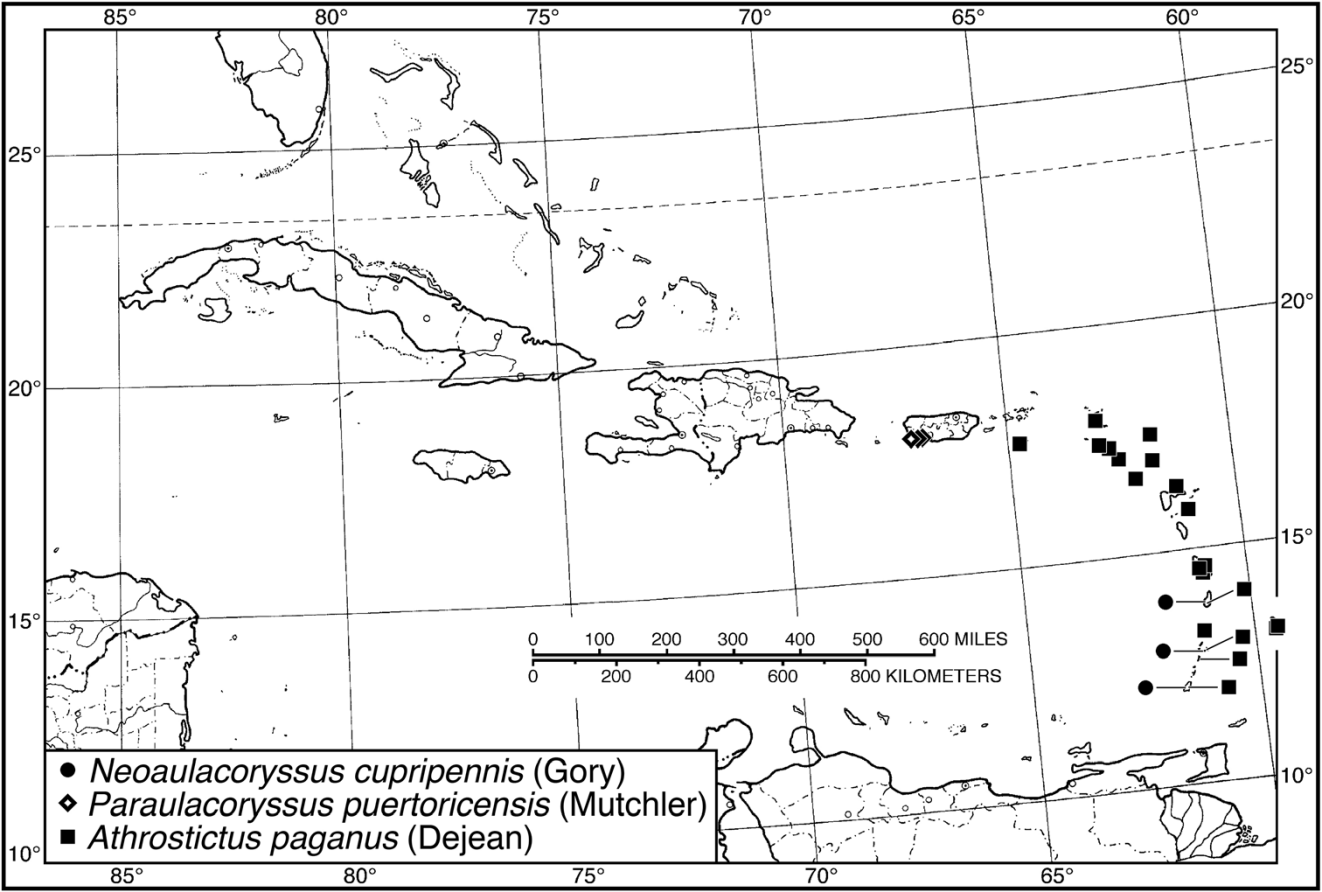
Map of West Indies showing known localities for species of *Neoaulacoryssus* Noonan, *Paraulacoryssus*, gen. n., and *Athrostictus* Bates.

Figs 1A , 2A–C , 3A , 4

Selenophorus cupripennis Gory, 1833: 239. TYPE MATERIAL: not seen by present authors; only a single specimen from “Cayenne”; sex unspecified.— Gemminger and Harold 1868: 266.— Csiki 1932: 1197.— Blackwelder 1944: 49.
Neoaulacoryssus cupripennis ; Noonan 1985a: 38.— Ball 1992: 85.— Lorenz 1998: 355.— Lorenz 2005: 376.

####### Taxonomic note.

Noonan (1985a: 38) suggested that *N. cupripennis* and *N. speciosus* (Dejean) may be conspecific. The everted endophallus of both *N. cupripennis* and *N. speciosus* was examined, as the form of the phallic median lobe was nearly identical. The three spine fields were similar in placement on the surface of the everted endophallus and length of spines, but differed in size and shape of the field. We believe that both of these are valid species.

####### Type area.

Cayenne.

####### Diagnosis.

The elytral macrosculpture, consisting of elongate punctures in places confluent and chain-like, readily separates this species from other West Indian selenophorine species. Specimens of *N. cupripennis* have the entire dorsum metallic cupreous, whereas specimens of *N. speciosus* have a greenish-bluish-violaceous head, greenish pronotum and reddish elytra.

####### Descriptive notes.

Data for SBL in Table 1. Habitus as in Fig. 1A. Labrum with anterior margin shallowly concave; clypeus with anterior margin moderately concave. Antennae and mouthparts rufo-brunneous to dark brunneous; antennal scape paler than remaining antennomeres. Legs rufo-brunneous; ventral surface rufo-brunneous to rufo-piceous. Entire dorsal surface with metallic cupreous luster. Pronotum with posteriolateral angles more or less obtuse; densely and more or less uniformly punctate, some punctures near lateral and posterior margins each with a very short seta. Elytral intervals densely punctate with elongate punctures, some of which are confluent and chain-like; each puncture with a very short seta near edge; setae longer in outer intervals. Males with fore- and mid-tarsi with biseriate adhesive vestiture. Both males and females with two terminal setae near the posterior margin on sternum VII.

**Male genitalia.** Fig. 2A–C. Apical portion of phallic median lobe long, narrowly tapered, symmetrically rounded in dorsal/ventral aspect, with 2 small ventral hooks; endophallus with three fields of short fine spines, a longer and wider field in dorsal aspect, a shorter and narrow field in left lateral aspect, and a small field near the ostium; without lamina. Ventral surface of shaft with two rows of basad directed sharp saw-toothed ridges.

**Ovipositor and female reproductive tract.** Very similar to those of *N. speciosus*, which is illustrated, Fig. 3A. Gonocoxite 2 (**gc2**) moderately thick, nearly straight. Moderately large bursa copulatrix (**bc**); long curved inflated spermatheca (**sp**) originating near base of common oviduct (**co**); spermatheca terminated with two sausage-like extensions; spermathecal gland duct originating near base of spermatheca. Spermathecal gland duct moderately long, gland triramous (**spg**), with bulb-like swelling of duct basad gland.

####### Geographical distribution.

Fig. 4. This is an eastern South American species, known from Cayenne on the mainland, the islands of the Dutch Antilles, and the islands of St. Lucia, Mustique and Grenada in the Lesser Antilles.

####### Chorological affinities and relationships.

The putative adelphotaxon of the eastern South American *N. speciosus*, this is the only species of *Neoaulacoryssus* currently recorded from the West Indies.

####### Material examined.

We have seen a total of 17 specimens (6 males, 11 females). See Appendix for details.

###### Genus Paraulacoryssus

Taxon classificationAnimaliaColeopteraCarabidae

Shpeley, Hunting & Ball gen. n.

http://zoobank.org/95B798F1-613D-48F3-BB18-03A23015A8F1

####### Type species.

*Selenophorus puertoricensis* Mutchler, 1934: 5; here designated.

####### Recognition.

Size larger, elytral mesh pattern transverse, sculpticells distinctly wider than long and metepisterum short, with lateral margin and anterior margin nearly equal.

####### Included species.

*Paraulacoryssus* includes only one species, *P. puertoricensis*.

####### Geographical distribution.

This genus is known only from Puerto Rico.

####### Chorological affinities and relationships.

Based on similarities in the remarkable female genitalia shared with *Neoaulacoryssus*, we postulate that that genus and *Paraulacoryssus* are adelphotaxa. In size and general appearance, members of this genus markedly resemble adults of the *opalinus* species group of *Selenophorus*. The marked morphological distinctness and single island distribution of this taxon suggests that it is a relict group in the West Indies.

###### Paraulacoryssus puertoricensis

Taxon classificationAnimaliaColeopteraCarabidae

(Mutchler)

Figs 1B , 2D–F , 3B , 4

Selenophorus puertoricensis Mutchler, 1934: 5. HOLOTYPE male: Desengano, Puerto Rico, December 1, W.T.M. Forbes (AMNH). PARATYPE female: Manidos, Puerto Rico, March 17, W.M. Wheeler (AMNH).— Darlington 1934: 104.— Blackwelder 1944: 50.— Erwin and Sims 1984: 440.— Ball 1992: 84, 85.— Lorenz 1998: 356.— Lorenz 2005: 377.

####### Type locality.

Desengano, Lajas Municipality, Puerto Rico.

####### Diagnosis.

This species is readily separated from all other West Indian selenophorine species by the reduced metepisternum, which has the anterior and lateral margins nearly equal in length.

####### Descriptive notes.

Data for SBL in Table 1. Habitus as in Fig. 1B. Labrum with anterior margin shallowly convex and clypeus with anterior margin shallowly concave. Antennae and mouthparts rufo-testaceous to nearly brunneous, with antennomere 1 paler than remainder of antenna. Legs rufo-brunneous to dark brunneous. Dorsal and ventral surfaces rufo-brunneous to brunneo-piceous. Elytra and ventral surface with faint iridescence. Head, pronotum and elytra shiny, without microlines visible at 100×. Pronotum with posteriolateral angles rounded; posteriolateral impressions and laterally near the bead finely punctate. Elytral striae impunctate, except the standard setigerous punctures in striae 2, 5 and 7. Intervals with fine micro-punctures. Both males and females with two terminal setae near the posterior margin on sternum VII.

**Male genitalia.** Fig. 2D–F. Apical portion of phallic median lobe triangular, symmetrically rounded in dorsal/ventral aspect; endophallus with three fields of short spines, best seen in left lateral aspect; well sclerotized, sharply pointed lamina present, short, triangular in form, rounded on right, concave on the left.

**Ovipositor and female reproductive tract.** Fig. 3B. Gonocoxite 2 moderately thick, nearly straight. Bursa copulatrix short; small kidney-shaped spermathecal basal sclerite (**sbs**) and long, inflated spermatheca (**sp**) originating near base of common oviduct, terminated with one or two sausage like extensions; spermathecal gland duct originating well above base of spermatheca 2. Spermathecal gland duct long, bulbous swelling of duct basad triramous gland (**spg**).

####### Geographical distribution.

Fig. 4. This species is only known from the Greater Antillean island of Puerto Rico.

####### Chorological affinities and relationships.

See above for treatment of the genus *Paraulacoryssus*.

####### Material examined.

In addition to type material, we have seen a total of 8 specimens (2 males, 6 females). See Appendix for details.

###### Genus Athrostictus

Taxon classificationAnimaliaColeopteraCarabidae

Bates

Athrostictus Bates, 1878: 592. TYPE SPECIES: Athrostictus sericatus Bates, 1878: 592 (designation by Noonan 1976: 41).— Blackwelder 1944: 48.— Reichardt 1977: 428.— Erwin and Sims 1984: 441.— Noonan 1985a: 35.— Lorenz 1998: 354.— Lorenz 2005: 376.
Arthrostictus Rye, 1880: 33 (misspelling).— Csiki 1932: 1195.

####### Recognition.

Both *Athrostictus* and *Neoaulacoryssus* are the only New World selenophorine genera whose species have short, dense setae on the elytral disc. In *Athrostictus*, the elytral punctures are round with longer setae, length approximately 3 or more times the width of the round punctures. In *Neoaulacoryssus* the elytral punctures are elongate, in places confluent and chain-like, with extremely short setae, length approximately half or less the width of the elongate punctures.

####### Included species.

Only one species, *Athrostictus paganus* (Dejean), is known from the West Indies.

###### Athrostictus paganus

Taxon classificationAnimaliaColeopteraCarabidae

(Dejean)

Figs 1C , 2G–I , 3C , 4

Hypolithus paganus Dejean (1831: 834). TYPE MATERIAL: 4 specimens in Chaudoir-Oberthür Collection (MNHP), in front of following box label: paganus/ Dej./ Colombie/ C. Dejean//. LECTOTYPE (here selected) male, [first in series] labelled: //[male]// Hypolithus// paganus m/ Carthagene [previous 3 labels hand printed on green paper]; second, female, labelled 202//; third, female, unlabelled; fourth, male, labelled “ Columb”.— Gemminger and Harold 1868: 268.
Hypolithus iridescens Chaudoir (1843: 783). TYPE MATERIAL: Not located; however, according to the original description, the holotype is a female that had been collected in Guadeloupe. — Gemminger and Harold 1868: 268.
Selenophorus puberulus Putzeys (1874: 119) (nec Dejean 1829). = S. pubifer Putzeys.
Selenophorus pubifer Putzeys (1878a: 69). TYPE MATERIAL: 5 specimens in Chaudoir-Oberthür Collection (MNHP), in front of the following box label: puberulus/ Chaud./ Venezuela/ Sallé. LECTOTYPE: male, labelled //337//.— Darlington 1934: 103.— Ball and Shpeley 1992: 96.
Selenophorus glabripennis Putzeys (1878a: 66). Since the name has not been used with reference to the West Indian fauna, might as well drop it. Nonetheless, data recorded *pro tem*. as if glabripennis is conspecific with paganus. 3 specimens in Chaudoir-Oberthür Collection (MNHP), in front of the following box label: glabripennis/ Chaud/ Colombie// LECTOTYPE: male, unlabelled, except for “Lectotype”.
Arthrostictus paganus ; Csiki 1932: 1195.— Blackwelder 1944: 48.— Ball 1992: 85.— Ball and Shpeley 1992: 96.— Lorenz 1998: 354.— Lorenz 2005: 376.— Ivie et al. 2008: 238.
Athrostictus iridescens ; Csiki 1932: 1195.— Erwin and Sims 1984: 441.

####### Type locality.

Vicinity of Carthagena, Bolivar Department, Colombia.

####### Diagnosis.

The long setae on the elytra readily separate this species from other West Indian selenophorine species.

####### Descriptive notes.

Data for SBL in Table 1. Habitus as in Fig. 1C. Clypeus and labrum each with anterior margin shallowly concave. Antennae, mouthparts and legs testaceous to rufo-testaceous; antennomere 1 paler than remaining antennomeres. Dorsal and ventral surfaces rufo-piceous to piceous; lateral margins of pronotum paler. Elytra and ventral surface with metallic blue iridescence. Basal third of pronotum markedly punctate, each puncture with a seta. Elytra with all intervals markedly punctate, each puncture with a seta about half the length of the serial setae in striae 2, 5 and 7. Males with fore and mid-tarsi with biseriate adhesive vestiture. Males with two terminal setae and females with four terminal setae near the posterior margin on sternum VII.

**Male genitalia.** Fig. 2G–I. Apical portion of phallic median lobe moderately long, parallel-sided, symmetrically rounded in dorsal/ventral aspect, small median ventral hook; endophallus with numerous spine fields, spines of varying base size and length; without lamina.

**Ovipositor and female reproductive tract.** Fig. 3C. Gonocoxite 2 falcate, with moderately wide base. Bursa copulatrix moderately long; small kidney-shaped spermatheca (**sp**) with proximal half attached to common oviduct, spermathecal duct originating well above base of common oviduct. Spermathecal gland duct (**sgd**) long, originating about mid-length of bursa copulatrix, gland long, thin, sausage-like (**spg**), with large bulbous swelling of duct basad gland. This unusual configuration of the spermathecal gland duct appended to the bursa was also observed in Bolivian specimens of *Athrostictus chlaenioides* Dejean.

####### Habitat.

Under the name *Selenophorus puberulus* Putzeys (not Dejean), M. J. Purves (1874: 12) noted this species (and *S. propinquus* Putzeys) as occurring in sugar cane fields in the Lesser Antillean island of Antigua.

####### Geographical distribution.

Fig. 4. The known range of this species in the West Indies extends from the Greater Antillean island of St. Croix through the Lesser Antilles to Grenada and south to Tobago.

####### Chorological affinities and relationships.

This is the only species of *Athrostictus* currently recorded from the West Indies. Its relationships are undetermined.

####### Material examined.

In addition to type material, we have seen a total of 76 specimens (36 males, 39 females, 1 unknown). See Appendix for details.

###### Genus Amblygnathus

Taxon classificationAnimaliaColeopteraCarabidae

Dejean

Amblygnathus Dejean, (1829: 62). TYPE SPECIES: Amblygnathus cephalotes Dejean (designation by Brullé 1835a: 10).— Gemminger and Harold 1868: 251.— Csiki 1932: 1193.— Blackwelder 1944: 48.— Noonan 1976: 42.— Reichardt 1977: 428.— Erwin and Sims 1984: 441.— Noonan 1985a: 44.— Ball and Maddison 1987: 196.— Lorenz 1998: 356.— Lorenz 2005: 378.— Bousquet 2012: 1134.

####### Recognition.

Within the Selenophori group, this genus is readily recognized by the enlarged head, and concave clypeus, with basal membrane of the labrum exposed medially. Additionally, the outer elytral intervals sparsely to moderately densely setose.

####### Included species.

Only three species of *Amblygnathus* are recorded in the West Indies: *A. cephalotes* Dejean (*cephalotes* species group), *A. puncticollis* (Putzeys) (*iripennis* species group) and *A. gilvipes gilvipes* Ball & Maddison (*suturalis* species group).

###### cephalotes species group

Taxon classificationAnimaliaColeopteraCarabidae

####### Recognition.

This species group is readily recognized by the large size of its adults: SBL more than 7.4 mm.

####### Included species.

The *cephalotes* species group includes in the West Indies only one species: *A. cephalotes* Dejean.

###### Amblygnathus cephalotes

Taxon classificationAnimaliaColeopteraCarabidae

Dejean

**Figure 5.**
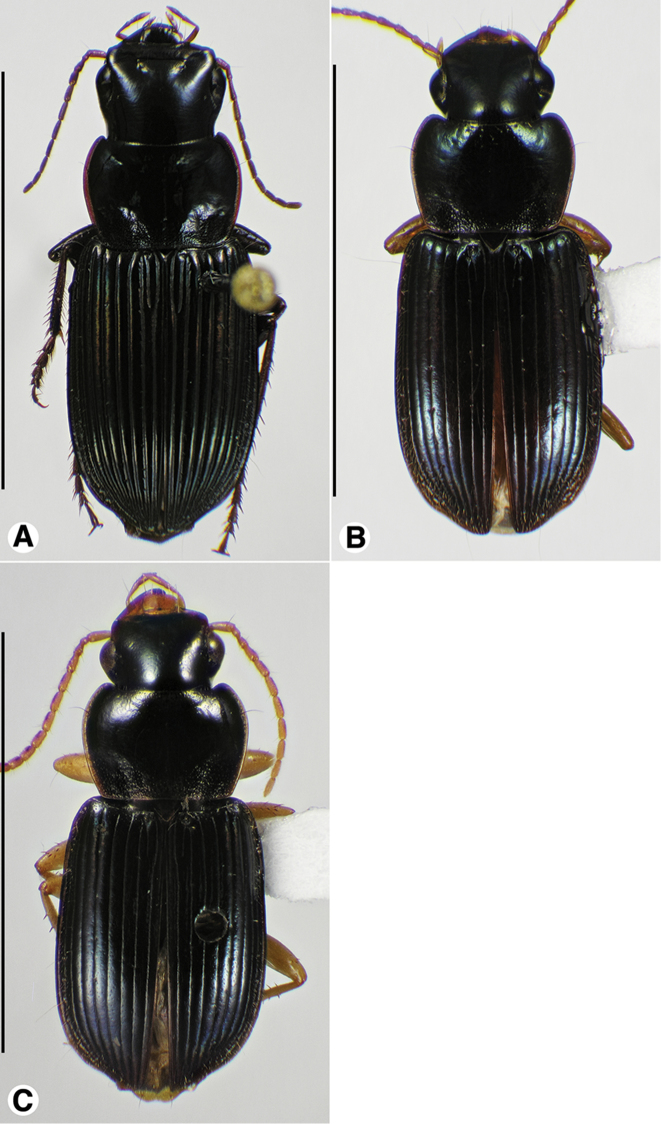
Habitus digital images of *Amblygnathus* species, dorsal aspect. **A** *A. cephalotes* Dejean **B** *A. puncticollis* (Putzeys) **C** *A. gilvipes gilvipes* Ball & Maddison. Scale bars: **A** 10 mm; **B–C** 5 mm.

**Figure 6.**
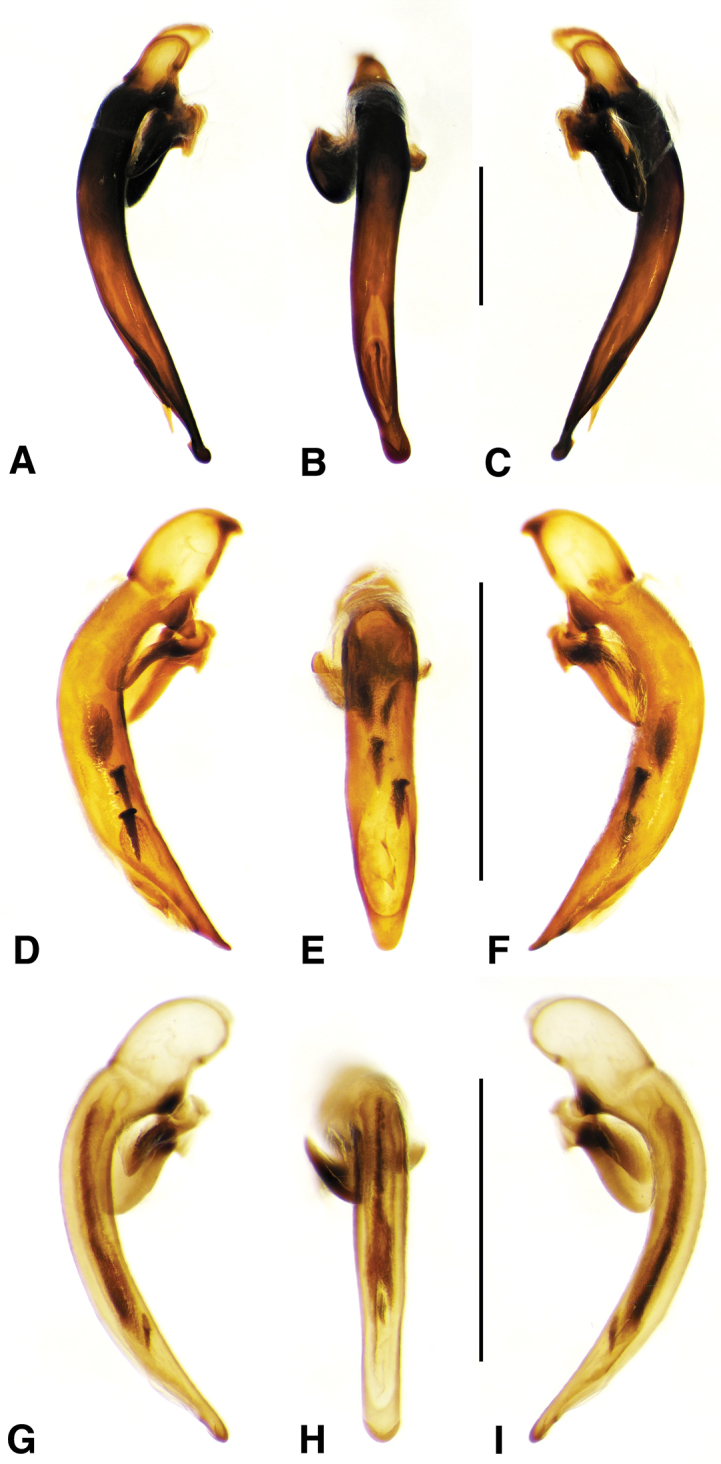
Digital images of male genitalia of *Amblygnathus* species. **A, D, G** right lateral aspect **B, E, H** dorsal aspect **C, F, I** left lateral aspect. **A–C** *A. cephalotes* Dejean **D–F** *A. puncticollis* (Putzeys) **G–I** *A. gilvipes gilvipes* Ball & Maddison. Scale bars 1 mm.

**Figure 7.**
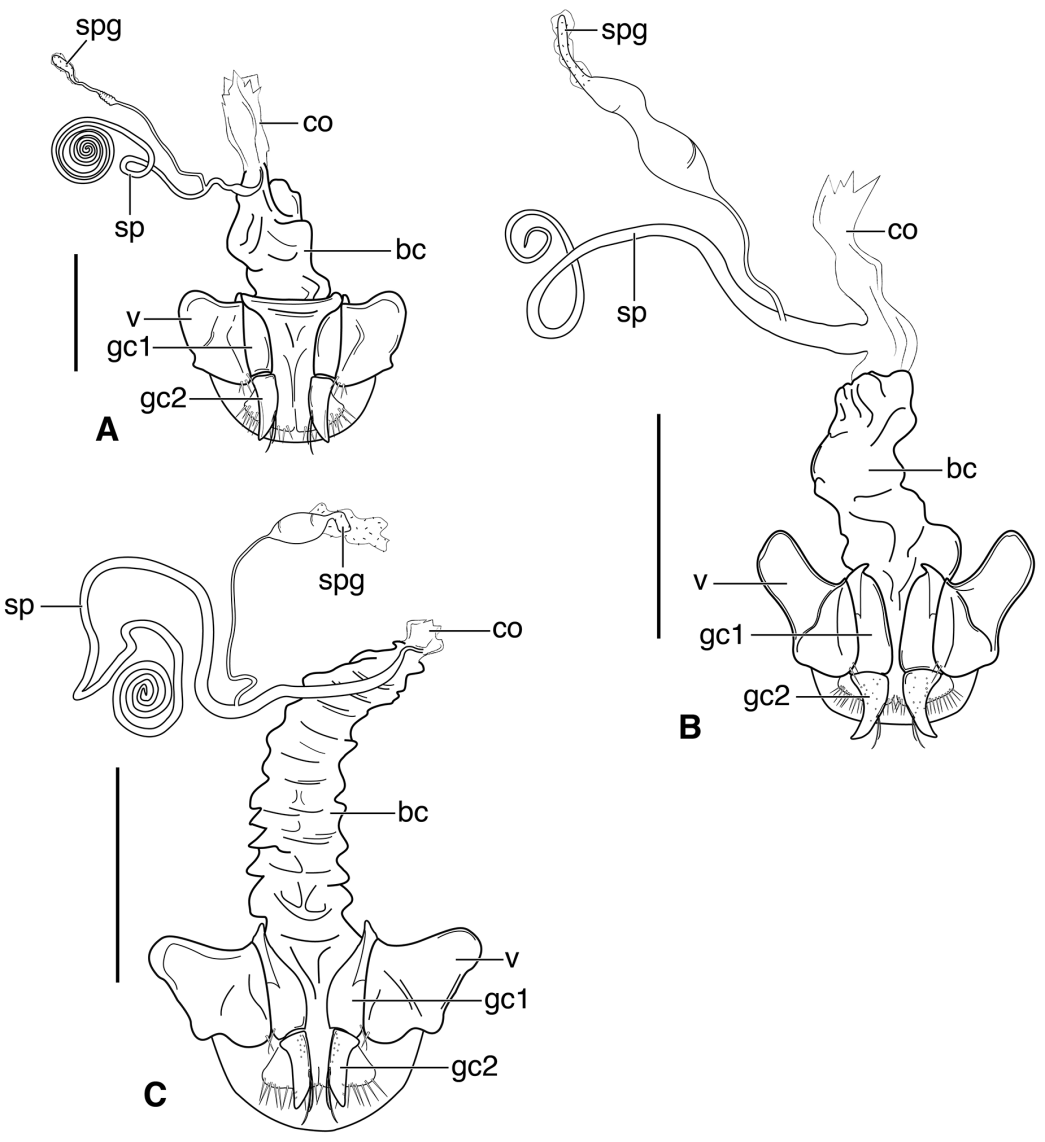
Line drawings of female reproductive tract of *Amblygnathus* species, ventral aspect. **A** *A. cephalotes* Dejean **B** *A. puncticollis* (Putzeys) **C** *A. gilvipes gilvipes* Ball & Maddison. Legend: **bc** bursa copulatrix; **co** common oviduct; **gc1** gonocoxite 1; **gc2** gonocoxite 2; **sp** spermatheca; **spg** spermathecal gland; **v** valvifer. Scale bars 1 mm.

**Figure 8.**
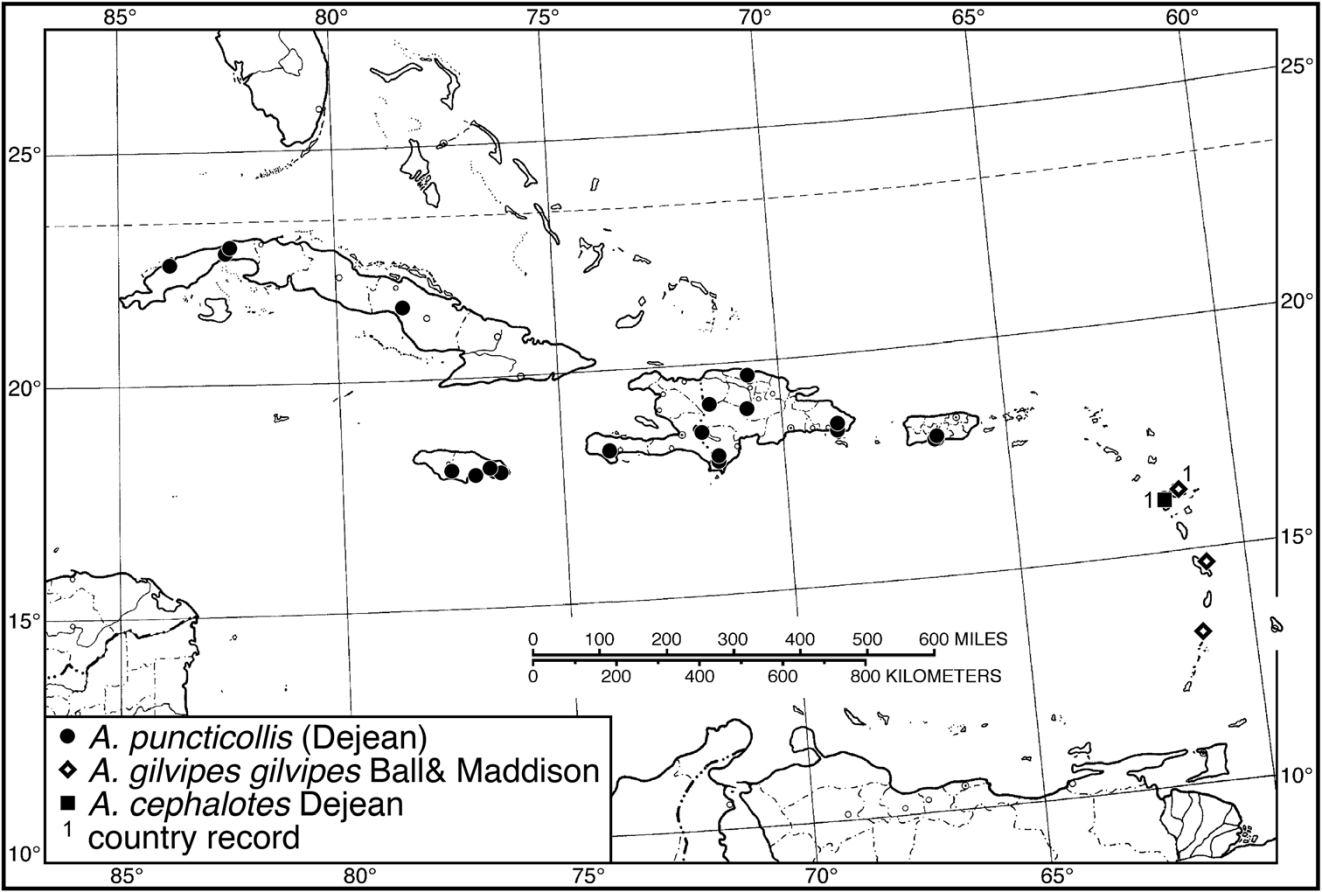
Map of West Indies showing known localities for species of *Amblygnathus* Dejean.

Figs 5A , 6A–C , 7A , 8

Amblygnathus cephalotes Dejean, 1829: 63. LECTOTYPE female, Oberthür coll. Box 204, labeled: cephalotes m. Cayenne [green paper]; ex Museo Chaudoir [red print] (MNHP) (selected by Ball and Maddison 1987: 245).— Gemminger and Harold 1868: 251.— Csiki 1932: 1193.— Blackwelder 1944: 48.— Ball and Maddison 1987: 245.— Ball 1992: 85.— Lorenz 1998: 356.— Lorenz 2005: 378.— Peck 2006: 176.— Peck et al. 2014: 15.
Amblygnathus vitraci Fleutiaux & Sallé, 1889: 364. HOLOTYPE female, labeled: Type; Guadeloupe Vitrac; Museum Paris collections Fleutiaux [handwritten]; Amblygnathus vitraci Fleutiaux and Sallé type [handwritten] (MNHP).

####### Type area.

French Guiana.

####### Diagnosis.

Larger size readily separates this species from *A. puncticollis* and *A. gilvipes gilvipes*.

####### Descriptive notes.

Data for SBL in Table 1. Habitus as in Fig. 5A. Both males and females with four terminal setae near the posterior margin on sternum VII.

**Male genitalia.** Fig. 6A–C. Apical portion of phallic median lobe moderately long, broadly rounded in dorsal aspect, with prominent dorsal flange; endophallus without darkened microtrichial fields; lamina present, long and narrow, tapered but rounded at apex.

**Ovipositor and female reproductive tract.** Fig. 7A. Gonocoxite 2 (**gc2**) very thick, nearly straight. Bursa copulatrix (**bc**) moderately short; spermatheca (**sp**) long, tightly coiled, attached near the base of the common oviduct (**co**). Spermathecal gland duct originating above the base of the spermatheca, spermathecal gland (**spg**) small, sausage-like, small swelling of duct before gland.

####### Geographical distribution.

Fig. 8. The range of this species extends from Bolivia and central Brazil northeast to Surinam, and north to the island of Guadeloupe in the Lesser Antilles.

####### Chorological affinities and relationships.

Within *Amblygnathus*, the West Indian range of this species is overlapped only by the range of *A. g. gilvipes*. The putative adelphotaxon of *A. cephalotes* is the Brazilian *A. gigas* Ball and Maddison (1987: 265, Fig. 70D).

####### Material examined.

We have not seen any additional specimens other than those reported by Ball and Maddison (1987: 247).

###### iripennis species group

Taxon classificationAnimaliaColeopteraCarabidae

####### Recognition.

This species group is readily recognized by the small size of its adults with SBL 4.45–5.64 mm, and the distinctly rounded posteriolateral angles of the pronotum.

####### Included species.

Within the West Indies, the *iripennis* species group includes only one species: *A. puncticollis* (Putzeys).

###### Amblygnathus puncticollis

Taxon classificationAnimaliaColeopteraCarabidae

(Putzeys)

Figs 5B , 6D–F , 7B , 8

Selenophorus puncticollis Putzeys, 1878a: 34. LECTOTYPE male, labelled: St. Domingo [green paper, handwritten]; Soc. Ent. Belg. Coll. Putzeys; det. Putzeys Selenophorus puncticollis Put.; Type [red print]; Amblygnathus puncticollis Putz. V. Emd. Det. 1937; R.I.Sc.N.B.I.G. (IRSB).— Csiki 1932: 1200; Darlington 1934: 104.— Blackwelder 1944: 50.
Amblygnathus puncticollis ; Erwin and Sims 1984: 441.— Ball and Maddison 1987: 223.— Ball 1992: 85.— Lorenz 1998: 356.— Lorenz 2005: 378.— Perez-Gelabert 2008: 79.

####### Type area.

“Santo Domingo” = Greater Antillean island of Hispaniola.

####### Diagnosis.

This species is readily separated from the other two West Indian *Amblygnathus* species on the basis of small size and its range restricted to the Greater Antilles.

####### Descriptive notes.

Data for SBL in Table 1. Habitus as in Fig. 5B. Males with two terminal setae and females with four terminal setae near the posterior margin on sternum VII.

**Male genitalia.** Figs 6D–F. Apical portion of phallic median lobe moderate in length, trapezoidal and broadly rounded in dorsal aspect, with very narrow dorsal flange visible only laterally on both sides; endophallus with two moderately long spines and two darkened microtrichial fields; lamina present, short, broad at base, tapered sharply at apex. A single male from San Vicente in Cuba has the apical portion of the phallic median lobe with a fully developed dorsal flange and the spines and michrotrichial fields of the endophallus are a bit differently oriented. At this time, we prefer to consider this a variant rather than a different species.

**Ovipositor and female reproductive tract.** Fig. 7B. Gonocoxite 2 falcate with moderately wide base. Bursa copulatrix moderately long; spermatheca (**sp**) long, loosely coiled, broadly attached near the base of the common oviduct. Spermathecal gland duct originating above the base of the spermatheca, spermathecal gland (**spg**) small, sausage-like, long double swelling of duct basad gland.

####### Geographical distribution.

Fig. 8. This species is known only from the Greater Antillean islands of Cuba, Hispaniola, Jamaica and Puerto Rico.

####### Chorological affinities and relationships.

The range of this species is not overlapped by the other West Indian species of *Amblygnathus*. Its putative adelphotaxon is the Middle American *A. woodruffi* Ball & Maddison. (See Ball and Maddison 1987: 261, Fig. 70B).

####### Material examined.

In addition to type material, we have seen a total of 19 specimens (12 males, 7 females). See Appendix for details.

###### suturalis species group

Taxon classificationAnimaliaColeopteraCarabidae

####### Recognition.

This species group is readily recognized by the small size of its adults with SBL 5.38–6.20 mm, and the more prominent posteriolateral angles of the pronotum.

####### Included species.

The *iripennis* species group, in the West Indies, includes only one taxon: *A. gilvipes gilvipes* Ball & Maddison.

##### *Amblygnathus gilvipes* Ball & Maddison

###### Amblygnathus gilvipes gilvipes

Taxon classificationAnimaliaColeopteraCarabidae

Ball & Maddison

Figs 5C , 6G–I , 7C , 8

Amblygnathus gilvipes gilvipes Ball & Maddison, 1987: 230. HOLOTYPE male, labeled: Chapada, Brazil Acc. No.2966; Insect Collection CARNEGIE MUSEUM OF NATURAL HISTORY Pittsburgh, Pa. [yellow paper] (CMNH). ALLOTYPE female, labeled same as holotype (CMNH). 41 PARATYPES from various Brazil localities, Venezuela, Surinam and French Guiana, and Guadeloupe in the Lesser Antilles.— Ball 1992: 85.— Lorenz 1998: 356.— Lorenz 2005: 378.— Peck et al. 2014: 15.

####### Type locality.

Chapada, State of Bahia, Brazil.

####### Diagnosis.

This species is readily separated from the other two West Indian *Amblygnathus* species on the basis of small size and its range restricted to the Lesser Antilles.

####### Descriptive notes.

Data for SBL in Table 1. Habitus as in Fig. 5C. Males with two terminal setae and females with four terminal setae near the posterior margin on sternum VII.

**Male genitalia.** Fig. 6G–I. Apical portion of phallic median lobe shorter than in *A. puncticollis*, broadly rounded in dorsal aspect, with well developed but narrow dorsal flange; endophallus with one moderate sized spine and an extensive darkened microtrichial field nearly as long as the shaft. Ball and Maddison (1987: 230) reported, evidently incorrectly, that a long slender lamina was present. The male genitalia of three previously dissected specimens were checked for the lamina, but it did not appear to be present.

**Ovipositor and female reproductive tract.** Fig. 7C. Gonocoxite 2 thick, nearly straight. Bursa copulatrix markedly long; spermatheca (**sp**) long, tightly coiled, attached near the base of the common oviduct. Spermathecal gland duct originating above the base of the spermatheca, spermathecal gland (**spg**) small, sausage-like, short bulbous swelling of duct basad gland.

####### Geographical distribution.

Fig. 8. The known range of this subspecies extends from Rio de Janeiro in southern Brazil north to Manaus in western Brazil, to Venezuela, Surinam and French Guiana, and to the islands of St. Vincent and Guadeloupe in the Lesser Antilles.

####### Chorological affinities and relationships.

The West Indian range of this subspecies is overlapped by only the range of *A. cephalotes*. This subspecies is the putative adelphotaxon of the Peruvian *A. gilvipes peruanus* Ball & Maddison.

####### Material examined.

In addition to type material, we have seen a total of 4 specimens (3 males, 1 female). See Appendix for details.

##### Genus Neodiachipteryx

Taxon classificationAnimaliaColeopteraCarabidae

Noonan

Neodiachipteryx Noonan (1985: 42). TYPE SPECIES: Selenophorus cariniger Putzeys, 1878a: 44 (designation by Noonan 1985a: 42).— Ball 1992: 84.— Lorenz 1998: 356.— Lorenz 2005: 378.

###### Recognition.

This genus is readily separated from others within the Selenophori group by the pronounced apical carina that extends from the lateral angle to the suture of the elytron. Noonan (1985a: 42, 43) states: “... by having the posterior portion of the seventh and eight elytral intervals joined into a raised longitudinal ridge extended from interval 8 to the suture and formed by the dorsum of the disc sloped over a prominent concave inflexion of the distal portion of the elytron...”.

###### Included species.

Both species of *Neodiachipteryx* are recorded from the Greater Antillean island of Hispaniola: *N. cariniger* (Putzeys) and *N. davidsoni*, new species.

##### Neodiachipteryx cariniger

Taxon classificationAnimaliaColeopteraCarabidae

(Putzeys)

**Figure 9.**
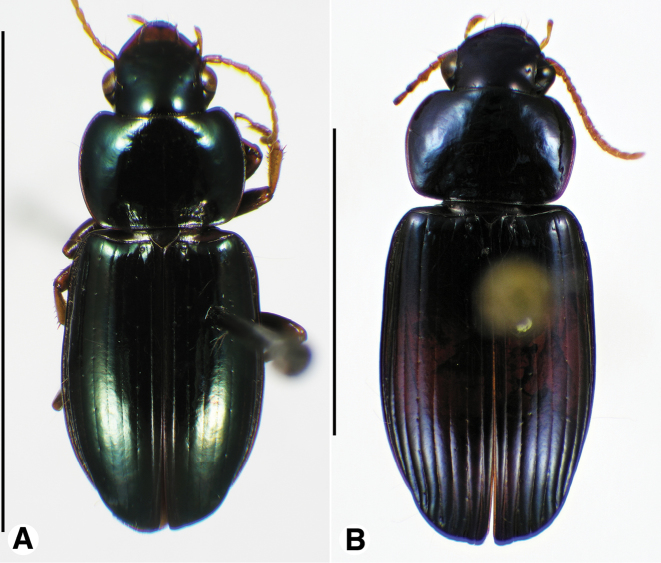
Habitus digital images, dorsal aspect, of *Neodiachipteryx* species. **A** *N. cariniger* (Putzeys) **B** *N. davidsoni* sp. n. Scale bars: **A** 10 mm; **B** 5 mm.

**Figure 10.**
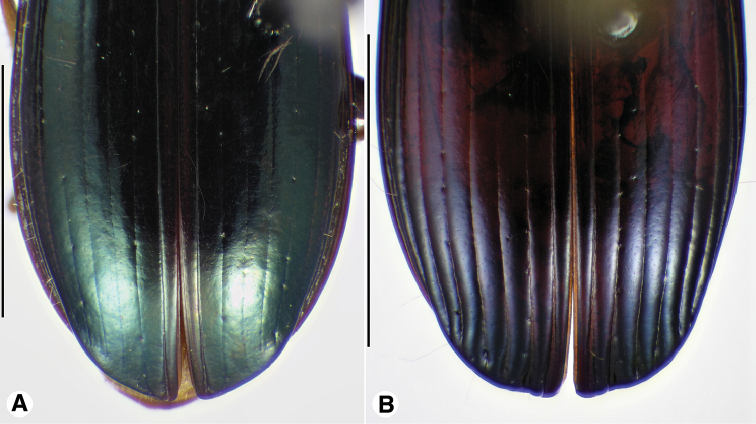
Digital images of elytra excluding base of *Neodiachipteryx* species, dorsal aspect. **A** *N. cariniger* (Putzeys) **B** *N. davidsoni* sp. n.. Scale bars 3 mm.

**Figure 11.**
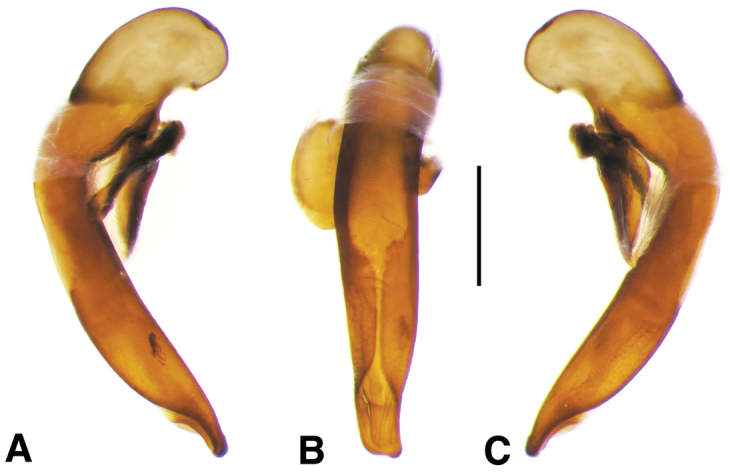
Digital images of male genitalia of *Neodiachipteryx cariniger* (Putzey). **A** right lateral aspect **B** dorsal aspect **C** left lateral aspect. Scale bar 1 mm.

**Figure 12.**
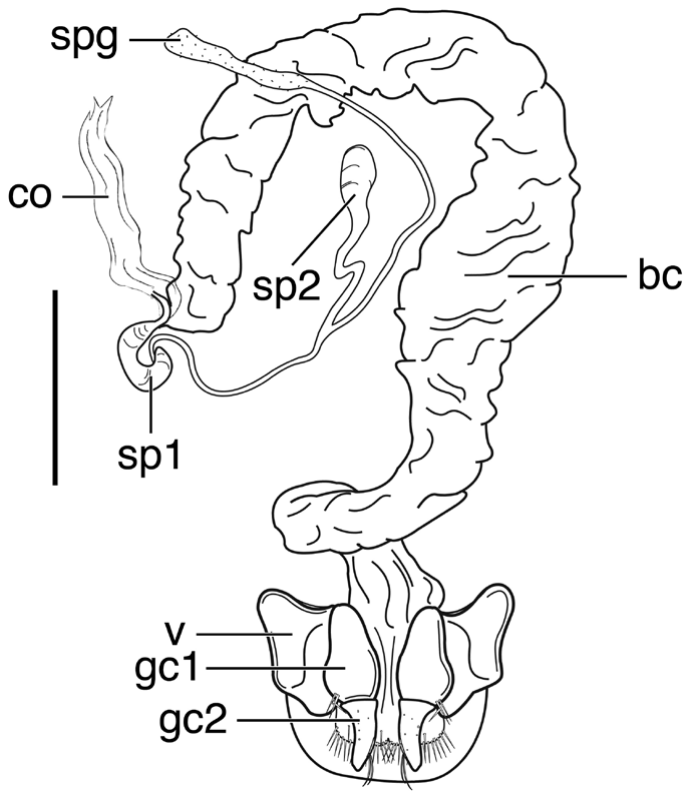
Line drawing of female reproductive tract of *Neodiachipteryx cariniger* (Putzeys). Legend: **bc** bursa copulatrix **co** common oviduct **gc1** gonocoxite 1 **gc2** gonocoxite 2 **sp** spermatheca **spg** spermathecal gland **spgd** spermathecal gland ductaaaa **v** valvifer. Scale bar 1 mm.

**Figure 13.**
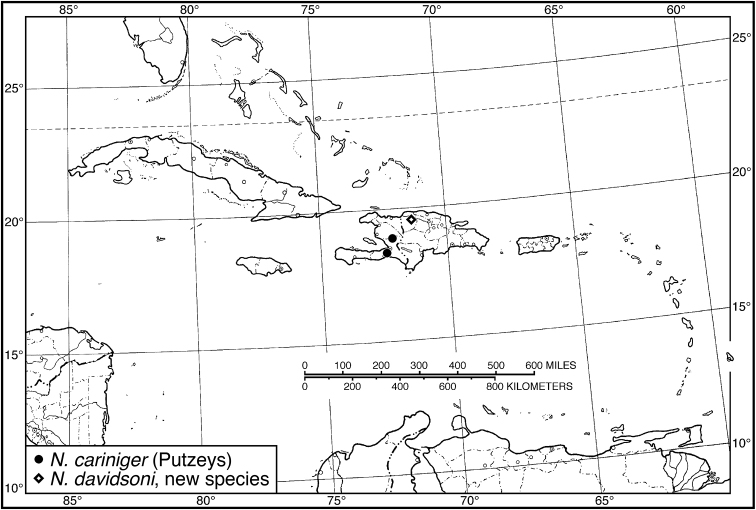
Map of West Indies showing known localities for species of *Neodiachipteryx* Noonan.

Figs 9A , 10A , 11A–C , 12 , 13

Selenophorus cariniger Putzeys, 1878a: 44. Three specimens, Chaudoir-Oberthür Collection (MNHP), in front of the following box label: careniger/ Chaud// Rep. Dominic/ Sallé//. LECTOTYPE male, (here selected), labelled: Ex. Musaeo/ Chaudoir// Type// LECTO// TYPE// Ball det. ‘72//.— Csiki 1932: 1196.— Blackwelder 1944: 49.— Erwin and Sims 1984: 440.
Selenophorus carniger Darlington, 1934: 103 (misspelling).
Neodiachipteryx cariniger ; Noonan 1985a: 42.— Ball 1992: 84, 85.— Lorenz 1998: 356.— Lorenz 2005: 378.— Perez-Gelabert 2008: 79.

###### Type area.

The Dominican Republic, the Spanish part of the Greater Antillean island of Hispaniola.

###### Diagnosis.

This species is readily separated from *N. davidsoni* by a combination of: labrum with anterior margin shallowly concave, not notched, and elytral intervals 3–5 flat at the apex of the elytra.

###### Descriptive notes.

Data for SBL in Table 1. Habitus as in Fig. 9A. Clypeus and labrum with anterior margin of each shallowly concave. Antennae, mouthparts and legs rufo-testaceous to rufo-brunneous; legs bicolored, with femora darker than remainder of leg. Dorsal and ventral surfaces rufo-brunneous to dark brunneous; dorsal surface with greenish blue metallic luster. Head, pronotum and elytra shiny, microlines not visible at 100×. Labrum with mesh pattern slightly transverse, sculpticells about 1.5–2× wide as long. Pronotum with posteriolateral angles rounded; posteriolateral impression impunctate. Elytral striae impunctate, except the standard setigerous punctures in striae 2, 5 and 7. Elytral interval 2 slightly convex at elytral apex; intervals 3–5 flat at elytral apex (Fig. 10A). The membranous hind wings are folded, not reduced in length. Males with two terminal setae and females with four terminal setae near the posterior margin on sternum VII.

**Male genitalia.** Fig. 11A–C. Apical portion of phallic median lobe markedly reduced, tip obliquely truncate in ventro/dorsal aspects; endophallus with one small darkened microtrichial field, right lateral ventral aspect; without lamina.

**Ovipositor and female reproductive tract.** Fig. 12. Gonocoxite 2 (**gc2**) falcate with moderately wide base. Bursa copulatrix (**bc**) quite long; small kidney-shaped spermatheca 1 (**sp1**) originating at base of common oviduct (**co**); subapical duct from spermatheca 1 connects to ducts of spermatheca 2 and spermathecal gland. Spermatheca 2 (**sp2**) with long duct, apical portion inflated. Both spermatheca 1 and spermatheca 2 the same in transparency of issue. Spermathecal gland (**spg**) with quite long duct, gland sausage-like.

###### Geographical distribution.

Fig. 13. This species is known only from the Greater Antillean island of Hispaniola.

###### Chorological affinities and relationships.

Both this species and its putative adelphotaxon, *N. davidsoni*, new species, are recorded from Hispaniola, but their known ranges do not overlap.

###### Material examined.

In addition to type material, we have seen a total of 8 specimens (6 males, 2 females). See Appendix for details.

##### Neodiachipteryx davidsoni sp. n.

Taxon classificationAnimaliaColeopteraCarabidae

http://zoobank.org/300330AA-2A55-420B-8621-AD68B1D54245

Figs 9B , 10B , 13

###### Specific epithet.

A Latinized eponym, genitive case, based on the surname of Robert L. Davidson, Section of Invertebrate Zoology, Carnegie Museum who recognized the single specimen to represent a new species and provided the specimen to the authors so that it could be included in this paper.

###### Type material.

Holotype male, labelled: “DOMINICAN/ REPUBLIC/ Sabaneta Prov./ Santiago Rodrigues/ Zamba/ August 2, 1980” (CMNH).

###### Type locality.

Zamba, municipality of Sabaneta, province of Santiago Rodrigues, Dominican Republic.

###### Diagnosis.

 This species is readily separated from *N. cariniger*, the only other species of *Neodiachipteryx*, by a combination of: labrum with anterior margin deeply notched medially and elytral intervals 3–5 moderately convex at the apex of the elytra. 

###### Descriptive notes.

Data for SBL in Table 1. Habitus as in Fig. 9B. Labrum with anterior margin shallowly concave; labrum with anterior margin deeply notched medially. Antennae and mouthparts rufo-testaceous; legs bicolored, femora rufo-brunneous, remainder of leg rufo-testaceous. Dorsal and ventral surfaces rufo-brunneous to dark brunneous; dorsal surface with greenish blue metallic luster. Head, pronotum and elytra shiny, microlines not visible at 100×. Labrum with mesh pattern slightly transverse, sculpticells about 1.5–2× wide as long. Pronotum with posteriolateral angles rounded; posteriolateral impression impunctate. Elytral striae impunctate, except the standard setigerous punctures in striae 2, 5 and 7. Elytral interval 2 markedly convex at elytral apex; intervals 3–5 moderately convex at elytral apex (Fig. 10B). The membranous hind wings are folded, not reduced in length.

Male genitalia. Unknown, the abdomen is missing from the holotype.

Ovipositor and female reproductive tract. Female unknown.

###### Geographical distribution.

Fig. 13. This species is known only from the Greater Antillean island of Hispaniola.

###### Chorological affinities and relationships.

 Both this species and *N. cariniger* are recorded from Hispaniola, but their known ranges do not overlap. 

###### Material examined.

Only the male holotype; for details, see above.

##### Genus Selenophorus

Taxon classificationAnimaliaColeopteraCarabidae

Dejean

Selenophorus Dejean, 1829: 80. TYPE SPECIES: Carabus palliatus Fabricius (designation by Hope 1838: 84).— Gemminger and Harold 1868: 251.— Csiki 1932: 1195.— Blackwelder 1944: 48.— Lindroth 1968: 821.— Noonan 1976: 41.— Reichardt 1977: 428.— Erwin and Sims 1984: 441.— Noonan 1985a: 38.— Ball 1992: 84.— Lorenz 1998: 355.— Lorenz 2005: 376.— Bousquet 2012: 1137.
Gynandropus Dejean, 1831: 810, 817. TYPE SPECIES: Gynandropus americanus Dejean (= G. hylacis Say) by monotypy.— Gemminger and Harold 1868: 259.— Csiki 1932: 1194.— Blackwelder 1944: 48.— Lindroth 1968: 820.— Noonan 1976: 42.— Reichardt 1977: 428.— Erwin and Sims 1984: 440 (listed as a junior synonym here).— Noonan 1985a: 39 (formally synonomized here).— Lorenz 1998: 355.— Lorenz 2005: 377.— Bousquet 2012: 1143.
Hemisopalus Casey, 1914: 134, 135. — TYPE SPECIES: Selenophorus opalinus LeConte (by original designation).— Csiki 1932: 1196.— Blackwelder 1944: 49.— Lindroth 1968: 823.— Noonan 1976: 41.— Reichardt 1977: 428.— Erwin and Sims 1984: 440.— Lorenz 1998: 355.— Lorenz 2005: 376.— Bousquet 2012: 1140.
Celiamorphus Casey, 1914: 134, 141. TYPE SPECIES: Selenophorus ellipticus Dejean (designated by Lindroth 1968: 828).— Csiki 1932: 1196. — Blackwelder 1944: 49.— Noonan 1976: 41.— Reichardt 1977: 428.— Erwin and Sims 1984: 440.— Lorenz 1998: 356.— Lorenz 2005: 378.— Bousquet 2012: 1137.

###### Recognition.

This genus is markedly divergent in its external features, includes a large number of species, and therefore, it is not possible to give an easy means of recognition. Identification of its members is best accomplished by use of the keys provided here, above.

###### Included taxa.

The 30 taxa of *Selenophorus* (*sensu lato*) recorded in the West Indies plus one doubtful species are arranged in two subgenera, and 10 species groups, with number of species in each group in parentheses: subgenus Celiamorphus-- *discopunctatus* species group (2), *latior* species group (3) and *seriatoporus* species group (1); subgenus Selenophorus (*sensu stricto*)-- *hylacis* species group (5), *mundus* species group (3), *nonseriatus* species group (3), *opalinus* species group (7), *palliatus* species group (4), *parumpunctatus* species group (2) and *striatopunctatus* species group (1).

##### Subgenus Celiamorphus

Taxon classificationAnimaliaColeopteraCarabidae

Casey

###### Synonymy.

See synonymy for genus *Selenophorus*.

###### Recognition.

Members of this subgenus have the hind tarsus nearly as long as the hind tibia. Additionally, males of all species in this subgenus have a lamina present near the base of the endophallus of the phallic median lobe. Identification of members is best done by using keys.

###### Description.

Basal lamina present on the endophallus at the apical opening of the phallic median lobe.

###### Included taxa.

Six species of subgenus Celiamorphus, arranged in three species groups, inhabit the West Indies.

##### Selenophorus discopunctatus

Taxon classificationAnimaliaColeopteraCarabidae

species group

###### Recognition.

Combination of the following characters: intermediate size (SBL 5.92–7.28 mm); elytra with mesh pattern isodiametric to slight transverse; and pronotum with posteriomedial area of disc moderately to densely punctate.

**SBL.** Males, 5.92–6.88 mm; females, 6.16–7.28 mm.

**Color.** Antennae and legs testaceous to slightly darker; palpi infuscated, tip testaceous. Dorsal and ventral surfaces brunneous to dark brunneous, not quite piceous; elytral epipleuron paler than disc.

**Luster**. Shiny without metallic reflection.

**Dorsal microsculpture.** Mesh pattern isodiametric to slightly transverse, microsculpture visible or not at 100× in males; microlines more impressed in females, visible at 100×.

**Male genitalia.** Apical portion of phallic median lobe with long taper, apex with prominent dorsal hook, or without hook. Preapical orifice anopic, moderately long; endophallus without macro spines, lamina present.

**Ovipositor and female reproductive tract.** Gonocoxite 2 moderately thick, nearly straight. Bursa copulatrix short, bowl-like apically; long spermatheca originating near base of common oviduct, without distinct narrowing basally; spermathecal gland duct originating near base of spermatheca; spermathecal gland small, somewhat bulbous.

###### Included species.

The *discopunctatus* species group includes two species in the West Indies: *S. discopunctatus* Dejean and *S. yucatanus* Putzeys.

###### Geographical distribution.

In the West Indies, the range of this species group is virtually co-extensive with the islands themselves.

##### Selenophorus discopunctatus

Taxon classificationAnimaliaColeopteraCarabidae

Dejean

**Figure 14.**
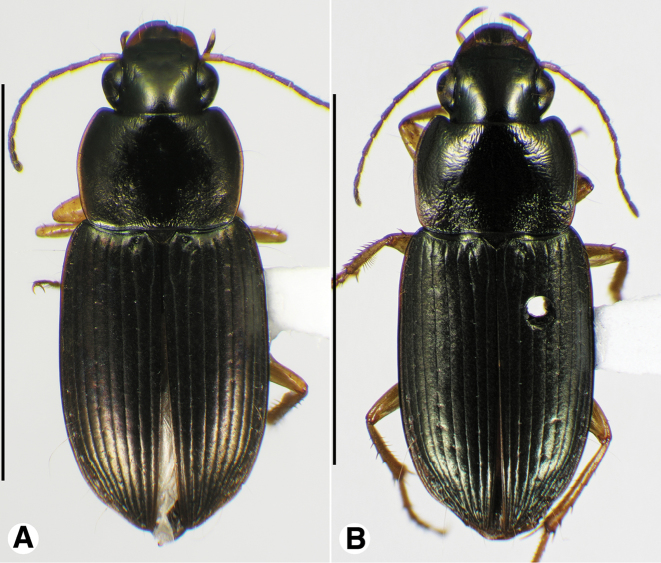
Habitus digital images of *Selenophorus discopunctatus* species group, dorsal aspect. **A** *S. discopunctatus* Dejean **B** *S. yucatanus* Putzeys. Scale bars 5 mm.

**Figure 15.**
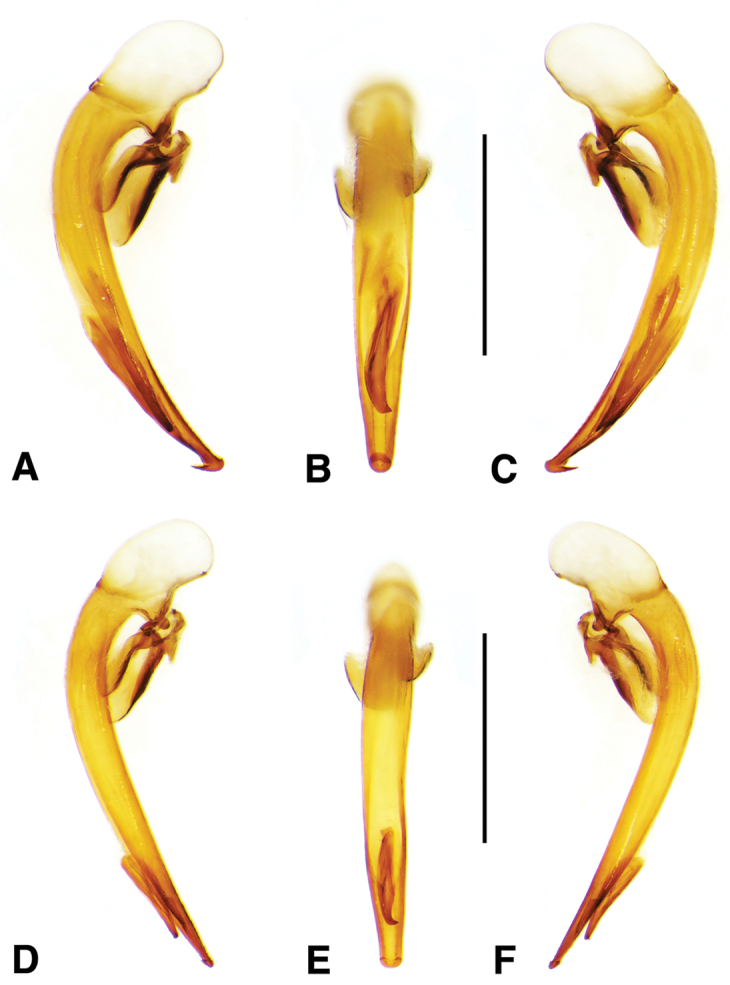
Digital images of male genitalia of *Selenophorus discopunctatus* species group. **A, D** right lateral aspect **B, E** dorsal aspect **C, F** left lateral aspect. **A–C** *S. discopunctatus* Dejean **D–F** *S. yucatanus* Putzeys. Scale bars 1 mm.

Figs 14A , 15A–C , 17

Selenophorus discopunctatus Dejean, 1829: 92. 39 specimens in Chaudoir-Oberthür Collection (MNHP) in front of following box label: discopunctatus/ Forsström/ Antilles/ C. Dejean; LECTOTYPE (here selected), male, labelled Schönherr// discopunctatus Sturm Forst/ palliatus Sch mihi/ in ins. St Barthelemy // [both labels hand printed on green paper]; //LECTO// //TYPE// Ball det. ‘72.— Gemminger and Harold 1868: 266.— Putzeys 1878a: 25.— Gundlach 1894: 293.— Csiki 1932: 1197.— Darlington 1934: 105.— Darlington 1935a: 161.— Blackwelder 1944: 49.—Erwin and Sims 1984: 440.— Ball 1992: 85.— Lorenz 1998: 356.— Peck and Thomas 1998: 22.— Lorenz 2005: 378.— Peck 2005: 32.— Peck 2006: 176.— Ivie et al. 2008: 238.— Perez-Gelabert 2008: 79.— Turnbow and Thomas 2008: 14.— Peck 2011: 13.— Bousquet 2012: 1137.
Selenophorus cuprinus Dejean, 1829: 96. TYPE MATERIAL: not located in Chaudoir-Oberthür Collection (MNHP).— Gemminger and Harold 1868: 266.— Putzeys 1878a: 25 (established the synonymy).
Selenophorus aeratus Reiche, 1843: 142. LECTOTYPE: male, in Chaudoir-Oberthür Collection (MNHP), labelled: aeratus Reiche/ Venezuela// LECTO// TYPE// [type labels hand printed, on red paper].— Gemminger and Harold 1868: 265.— Putzeys 1878a: 25 (established the synonymy).
Selenophorus harpaloides Reiche, 1843: 142. LECTOTYPE: female, in Chaudoir-Oberthür Collection (MNHP), labelled harpaloides/ Reiche Rev./Cuv. 1843/ Caracas// LECTO// TYPE// [type labels hand printed, on red paper].— Gemminger and Harold 1868: 266.— Putzeys 1878a: 25 (established the synonymy).
Selenophorus subpunctatus Reiche, 1843: 143. LECTOTYPE: female, in Chaudoir-Oberthür Collection (MNHP), labelled: subpunctatus/ Reiche Rev/ Cuv.[...illegible]//.— Gemminger and Harold 1868: 267. According to the original description, the provenance of this specimen is Venezuela, near Caracas (Putzeys 1878a: 72 [entry in index]). This specimen was found among the members of S. discopunctatus, as recorded above, suggesting that it was regarded as conspecific with that species. However, Putzeys did not record the name in the synonymy of S. discopunctatus, nor did he include the name in the text of his treatment of Selenophorus. We treat it here as the name of a species *incertae sedis*.
Selenophorus chokoloskei Leng, 1915: 596. Synonymy established by Darlington 1935a: 161. According to Bousquet (2012: 1138) location of the syntypes is unknown.

###### Type area.

Saint Barthélemy, Leeward Islands, Lesser Antilles.

###### Diagnosis.

This species is readily separated from the other member of the *discopunctatus* species group by the posteriolateral impressions of pronotum, which are moderately to densely punctate, but smooth, not rugose. Additionally, apical portion of male genitalia with a prominent dorsal hook (Fig. 15A, C; cf. Fig. 15D, F) .

###### Descriptive notes.

Data for SBL in Table 1. Habitus as in Fig. 14A. Labrum with anterior margin shallowly concave; clypeus with anterior margin moderately concave. Antennae and legs testaceous to slightly darker; palpi infuscated, tip testaceous, base slightly to much darker, maxillary palpomere 3 same color as base of maxillary palpomere 4. Dorsal and ventral surfaces brunneous to dark brunneous, not quite piceous; elytral epipleuron paler than disc. Frons and disc of pronotum shiny, with isodiametric microsculpture visible at 100×, microlines more impressed in females; posteriolateral impressions of pronotum with mesh pattern isodiametric; elytra granular, with mesh pattern isodiametric. Elytral striae impunctate, except the standard setigerous punctures in striae 2, 5 and 7. Punctures of striae 2, 5 and 7 foveate. Males with two terminal setae and females with four terminal setae near the posterior margin on sternum VII.

**Male genitalia.** Fig. 15A–C. Apical portion of phallic median lobe with long taper, symmetrically rounded in dorsal/ventral aspect, with prominent dorsal hook; endophallus with four long spines, approximately medial in position; lamina present, more or less banana shaped, pointed at apex.

**Figure 16.**
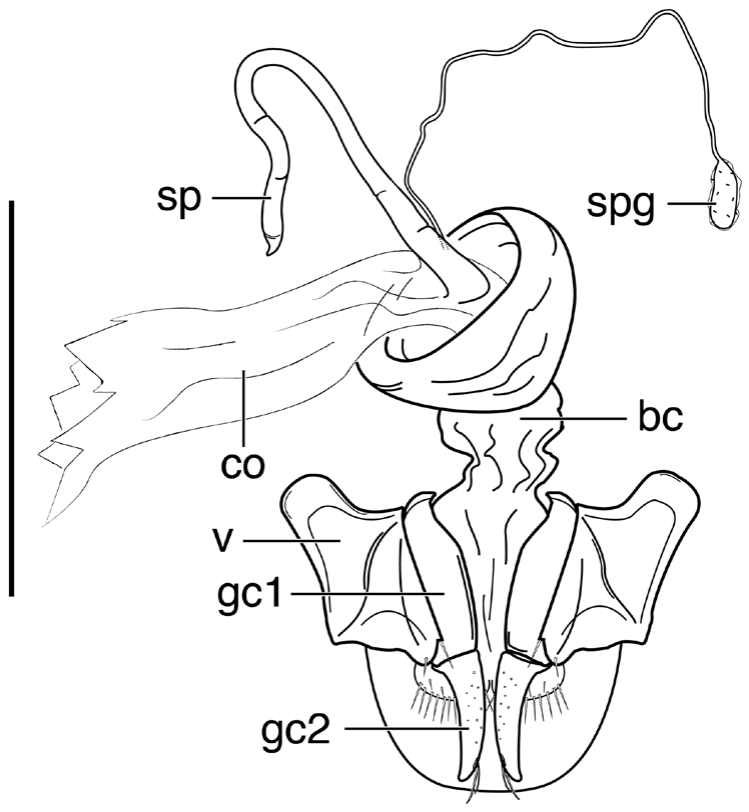
Line drawing of female reproductive tract of *Selenophorus discopunctatus* species group, in part, *S. yucatanus* Putzeys, ventral aspect. Legend: **bc** bursa copulatrix; **co** common oviduct **gc1** gonocoxite 1 **gc2** gonocoxite 2 **sp** spermatheca **spg** spermathecal gland **v** valvifer. Scale bar 1 mm.

**Ovipositor and female reproductive tract.** Very similar to that of *S. yucatanus*, Fig. 16. For details, see this topic for *S. yucatanus*, below.

###### Geographical distribution.

**Figure 17.**
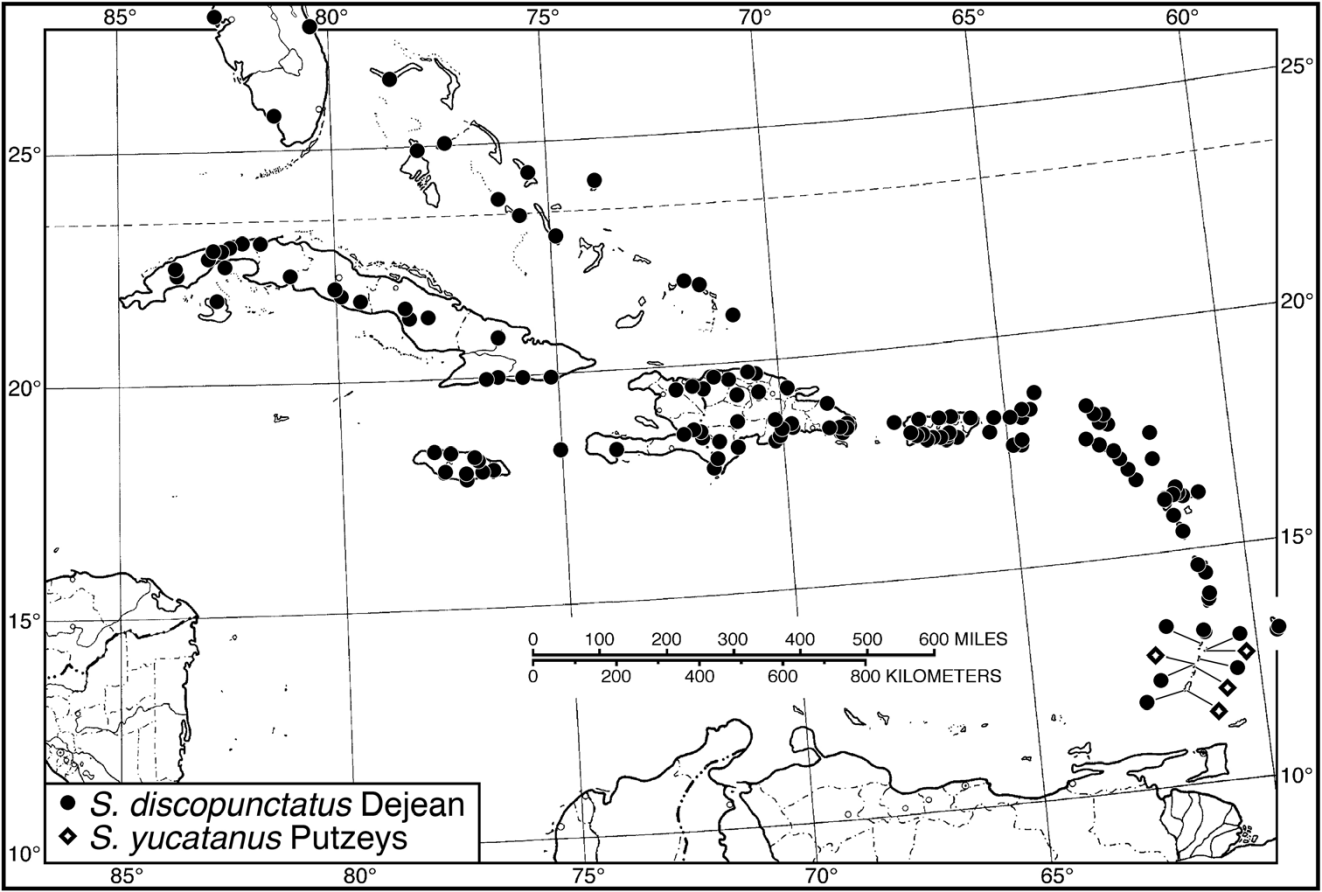
Map of West Indies showing known localities for species of *Selenophorus discopunctatus* species group.

Fig. 17. This wide-ranging species is found on most of the island groups in the West Indies, with the exception of the Greater Antillean Caymans.

###### Chorological affinities and relationships.

The West Indian range of this widely distributed species overlaps the range of *S. yucatanus* in the Lesser Antillean Grenadines. Its relationships are not postulated beyond species group membership.

###### Material examined.

In addition to type material, we have seen a total of 1,435 specimens (714 males, 720 females, 1 unknown). See Appendix for details.

##### Selenophorus yucatanus

Taxon classificationAnimaliaColeopteraCarabidae

Putzeys

Figs 14B , 15D–F , 16 , 17

Selenophorus yucatanus Putzeys, 1878a: 24. TYPE MATERIAL: female, in front of the following box label: yucatanus/ Chaud/ Yucatan/ Pilate; LECTOTYPE female, labelled: Ex Musaeo/ Chaudoir// Bates vidit/ X^le^ 1881// Type//. — Csiki 1932: 1202.— Blackwelder 1944: 50.— Ball 1992: 85.— Ball and Shpeley 1992: 96.— Lorenz 1998: 356.— Lorenz 2005: 378.

###### Notes.

According to the original description (Putzeys 1878a: 24), this species description is based on a single specimen, sex not specified (see details above). In spite of the statement in the original description, in the Chaudoir-Oberthür Collection are two males and a female, in front of the following box label: “Yucat”. Each of the specimens is labelled “Type”. Under the circumstances, it seems best to treat one of the specimens as a lectotype, rather than as a holotype.

###### Type area.

Yucatan Peninsula, México.

###### Diagnosis.

This species is readily separated from the other West Indian member of the *discopunctatus* species group by the posteriolateral impressions of pronotum, which are densely punctate and rugose. Additionally, the apical portion of the phallic median lobe lacks a hook.

###### Descriptive notes.

Data for SBL in Table 1. Habitus as in Fig. 14B. Clypeus with anterior margin moderately concave. Labrum with anterior margin shallowly concave. Antennae and legs testaceous to slightly darker; palpi infuscated, tip testaceous, base slightly to much darker, maxillary palpomere 3 same color as base of maxillary palpomere 4. Dorsal and ventral surfaces brunneous to dark brunneous, not quite piceous; elytral epipleuron paler than disc. Frons shiny in males and females, with mesh pattern isodiametric; disc of pronotum shiny in males and females, males without microlines visible at 100×, females with microlines visible at 100×, sculpticells about 2× wide as long; posteriolateral surface of pronotum in males and females with mesh pattern isodiametric. Elytra with mesh pattern slightly transverse, sculpticells about 1.5–2× wide as long. Elytral striae impunctate, except the standard setigerous punctures in striae 2, 5 and 7. Punctures of striae 2, 5 and 7 foveate. Males with two terminal setae and females with four terminal setae near the posterior margin on sternum VII.

**Male genitalia.** Fig. 15D–F. Apical portion of phallic median lobe with long taper, symmetrically rounded in dorsal/ventral aspect, with two small dorsal projections; endophallus without spines or dark microtrichial fields; lamina present, long, more or less ovoid, with tip curved to left, pointed at apex. Ventral surface of shaft with two rows of finely saw-toothed ridges.

**Ovipositor and female reproductive tract.** Fig. 16. Gonocoxite 2 (**gc2**) moderately thick, nearly straight. Bursa copulatrix (**bc**) short, bowl-like apically; long spermatheca (**sp**) originating near base of common oviduct (**co**), without distinct narrowing basally; spermathecal gland duct originating near base of spermatheca; spermathecal gland (**spg**) small, somewhat bulbous.

###### Geographical distribution.

Fig. 17. This species is only recorded from the Lesser Antillean islands of Grenada, Mustique and Union in the West Indies. On the mainland it is known from the Middle American Yucatan Peninsula.

###### Chorological affinities and relationships.

The known West Indian range of this species is overlapped by that of the closely related *S. discopunctatus*. Its relationships are not postulated beyond species group membership.

###### Material examined.

In addition to type material, we have seen a total of 53 specimens (30 males, 23 females). See Appendix for details.

##### Selenophorus latior

Taxon classificationAnimaliaColeopteraCarabidae

species group

###### Recognition.

Combination of the following characters: smaller size (SBL 4.88–5.84 mm); elytra with mesh pattern slight transverse to very transverse or absent; and pronotum with posteriomedial area of disc impunctate, or with reduced punctation.

**SBL.** Males, 4.93–5.84 mm; females, 4.88–5.68 mm.

**Color.** Antennae variously colored: unicolorous testaceous; or with basal one or two antennomeres testaceous to brunneous and remaining antennomeres darker. Mouthparts: testaceous to infuscated, rufous to rufo-brunneous, with tips testaceous. Legs: testaceous to rufo-brunneous or femora bicolored, rufous to brunneous, base paler, tibae paler than femora, testaceous to rufo-testaceous. Dorsal surface: rufo-brunneous to brunneo-piceous, elytral disc with or without a darker central cloud in intervals 1–6. Ventral surface rufous to brunneo-piceous. Elytral epipleuron paler than disc.

**Luster.** Shiny, elytra with faint blue-green metallic reflection or subiridescent.

**Dorsal microsculpture.** Dorsal surface with no microlines or just a few visible at 100×, or head with mesh pattern isodiametric, pronotum and elytra with mesh pattern transverse, sculpticells 1.5–4× wide as long.

**Male genitalia.** Apical portion of phallic median lobe with long to very long taper, apex with small dorsal hook, blunted, or curved dorsally. Preapical orifice anopic, moderately long to very long; endophallus with or without macro spines, lamina present.

**Ovipositor and female reproductive tract.** Only *S. latior* was examined. Gonocoxite 2 moderately thick, somewhat falcate. Bursa copulatrix moderately long; spermatheca moderately long, sausage-like, originating near base of common oviduct; markedly long spermathecal gland duct originating near base of spermatheca; spermathecal gland small, sausage-like, with bulbous swelling of duct, larger than gland, basad gland.

###### Included species.

The *latior* species group includes three species: *S. barbadensis* Ball and Shpeley, *S. latior* Darlington, and *S. solitarius* Darlington.

###### Geographical distribution.

The range of this species group extends in the Greater Antilles from Cuba to Hispaniola, Puerto Rico, the Virgin Islands, and through the Lesser Antilles to Grenada.

##### Selenophorus barbadensis

Taxon classificationAnimaliaColeopteraCarabidae

Ball & Shpeley

**Figure 18.**
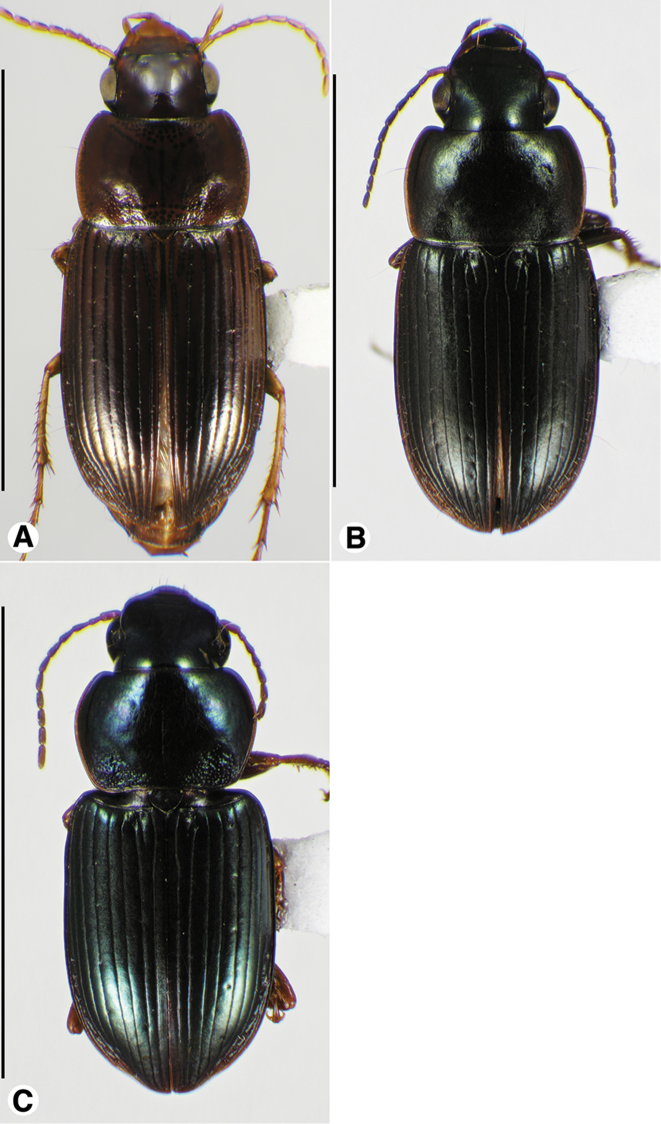
Habitus digital images of *Selenophorus latior* species group, dorsal aspect. **A** *S. barbadensis* Ball & Shpeley **B** *S. latior* Darlington **C** *S. solitarius* Darlington. Scale bars 5 mm.

**Figure 19.**
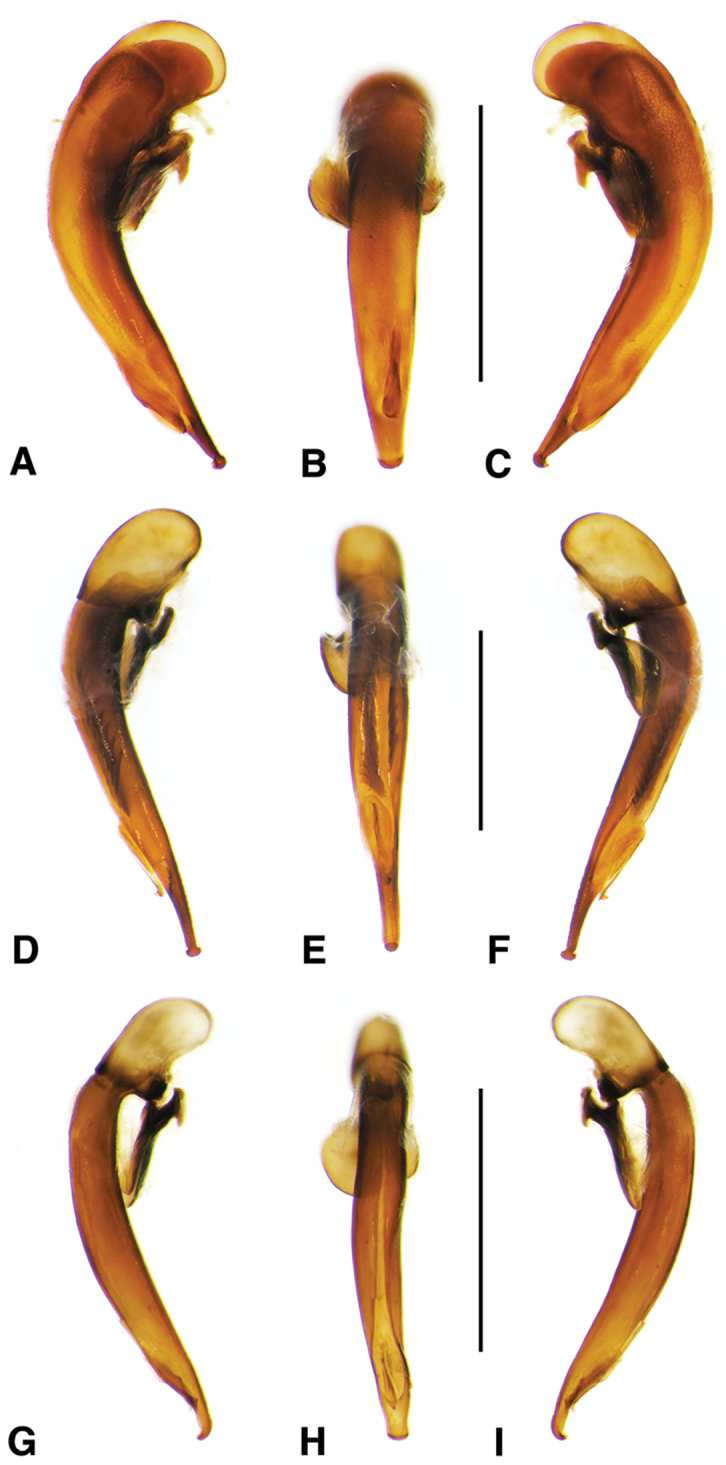
Digital images of male genitalia of *Selenophorus latior* species group. **A, D, G** right lateral aspect **B, E, H** dorsal aspect **C, F, I** left lateral aspect **A–C** *S. barbadensis* Ball & Shpeley **D–F** *S. latior* Darlington **G–I** *S. solitarius* Darlington. Scale bars 1 mm.

Figs 18A , 19A–C , 21

Selenophorus barbadensis Ball & Shpeley, 1992: 100.— Ball 1992: 84, 85.— Lorenz 1998: 355.— Lorenz 2005: 376.— Peck 2009: 13.

###### Type material.

Complete label data for type material (holotype (FSCA), allotype, and 9 paratypes) are provided in the original description.

###### Type locality.

Cavehill, Parish of St. Michael, Barbados, Lesser Antilles.

###### Diagnosis.

This species is readily separated from the other species in the *latior* species group by a combination of: dorsal surface without visible microlines and pronotum with posteriolateral impressions finely punctate.

###### Descriptive notes.

Data for SBL in Table 1. Habitus as in Fig. 18A. Clypeus and labrum with anterior margin of each shallowly concave. Antennae, mouthparts and legs testaceous. Dorsal surface rufo-brunneous to brunneous; elytral disc with darker central cloud in intervals 1–6. Ventral surface rufous to rufo-brunneous; elytral epipleuron paler. Elytra and ventral surface with faint bluish iridescence. Head, pronotum and elytra shiny, microlines not visible at 100×. Pronotum with posteriolateral impressions finely punctate; posteriolateral angles rounded. Interruptions in the elytral striae give the appearance of punctures; standard setigerous punctures in striae 2, 5 and 7. Elytral intervals finely punctate. Males with two terminal setae and females with four terminal setae near the posterior margin on sternum VII.

**Male genitalia.** Fig. 19A–C. Apical portion of phallic median lobe long, narrowly tapered, symmetrically rounded in dorsal/ventral aspect, tip finely capped, bulb-like, dorsal flange turned up, hook-like; endophallus with one dark microtrichial field near basal bulb; lamina present, widened distally, rounded at apex. Ventral surface of shaft with two short rows of basad directed finely saw-toothed ridges.

**Ovipositor and female reproductive tract.** Not studied.

###### Geographical distribution.

Fig. 21. This species is known only from the Lesser Antillean islands of Barbados and St. Vincent.

###### Chorological affinities and relationships.

This species is the only member of the *latior* species group recorded from Barbados. Its relationships are not postulated beyond species group membership.

###### Material examined.

In addition to type material, we have seen a total of 12 specimens (5 males, 7 females). See Appendix for details.

**Figure 20.**
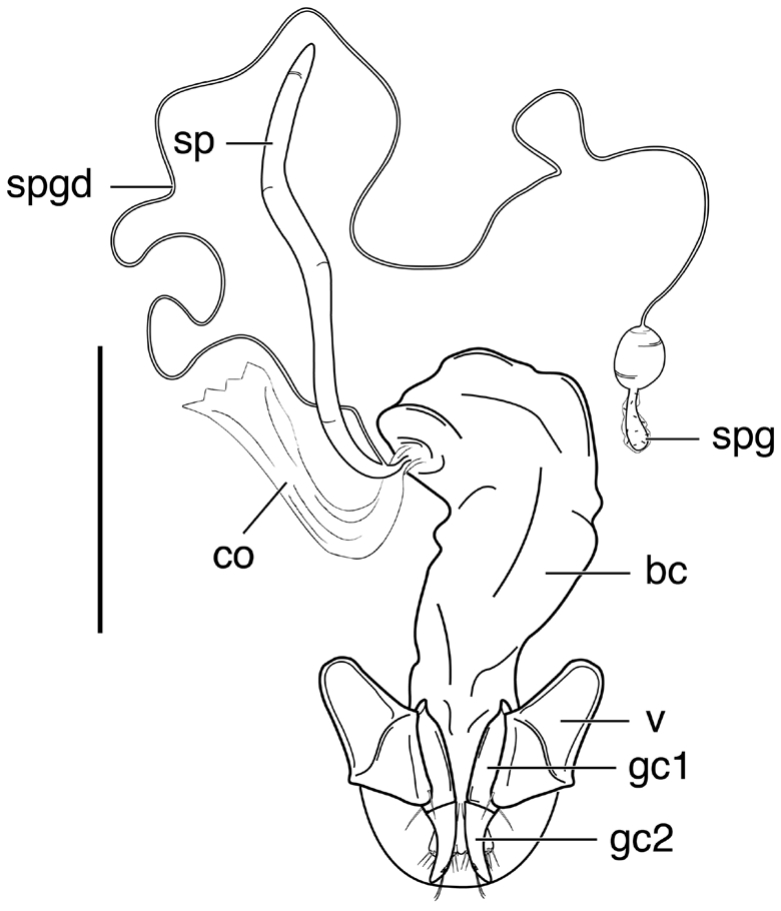
Line drawing of female reproductive tract of *Selenophorus latior* species group, in part, *S. latior* Darlingtoni, ventral aspect. Legend: **bc** bursa copulatrix **co** common oviduct **gc1** gonocoxite 1 **gc2** gonocoxite 2 **sp** spermatheca **spg** spermathecal gland **spgd** spermathecal gland duct **v** valvifer. Scale bar 1 mm.

##### Selenophorus latior

Taxon classificationAnimaliaColeopteraCarabidae

Darlington

**Figure 21.**
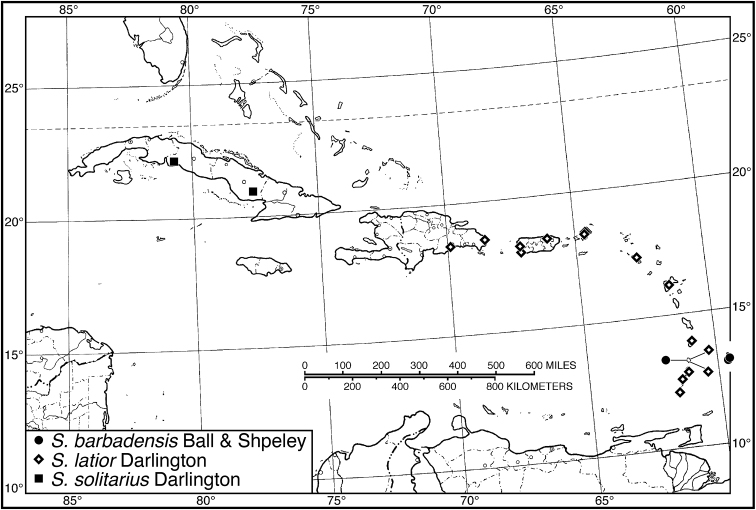
Map of West Indies showing known localities for species of *Selenophorus latior* species group.

Figs 18B , 19D–F , 20 , 21

Selenophorus latior Darlington, 1934: 109. HOLOTYPE male: Haina, Santo Domingo, G.N. Wolcott (AMNH). One female PARATYPE: Pt. Congrejos, Puerto Rico, Feb. 8, 1920, G.N. Wolcott (USNM).— Blackwelder 1944: 49. — Erwin and Sims 1984: 440.— Ball 1992: 85.— Lorenz 1998: 355.— Lorenz 2005: 377.— Perez-Gelabert 2008: 79.

###### Type locality.

Haina, Santo Domingo Province, Dominican Republic, Hispaniola.

###### Diagnosis.

This species is readily separated from the other species in the *latior* species group by a combination of: elytra with slightly transverse microsculpture, sculpticells about 2–4× wide as long and pronotum with posteriolateral impressions impunctate.

###### Descriptive notes.

Data for SBL in Table 1. Habitus as in Fig. 18B. Clypeus and labrum with anterior margin of each shallowly concave. Antennae with one or two basal antennomeres testaceous, remaining antennomeres darker; palpi infuscated, rufous to rufo-brunneous, tips testaceous; femora bicolored, rufous to brunneous, base paler; tibae paler than femora, testaceous to rufo-testaceous. Dorsal and ventral surfaces brunneous to brunneo-piceous; elytral epipleuron paler than disc. Head with mesh pattern isodiametric; pronotum with mesh pattern slightly transverse, sculpticells about 1.5× wide as long; elytra subiridescent, with mesh pattern transverse, sculpticells about 2–4× wide as long. Pronotum with posteriolateral impressions impunctate; posteriolateral angles rounded. Elytral striae impunctate, except the standard setigerous punctures in striae 2, 5 and 7. Males with a brush of about 24 long setae and females with only about 7 long setae on anterioventral margin of fore-femur. Males with two terminal setae and females with four terminal setae near the posterior margin on sternum VII.

**Male genitalia.** Fig. 19D–F. Apical portion of phallic median lobe markedly long, narrowly tapered, tip capped, bulb-like, with sharp edges in right and left lateral aspects; endophallus with two rows of long spines, the left row longer than the right row; lamina with tip rounded, hook on left side. Ventral surface of shaft with two rows of basally directed saw-toothed ridges.

**Ovipositor and female reproductive tract.** Fig. 20. Gonocoxite 2 (**gc2**) moderately thick, somewhat falcate. Bursa copulatrix (**bc**) moderately long; spermatheca (**sp**) moderately long, sausage-like, originating near base of common oviduct (**co**); markedly long spermathecal gland duct (**spgd**) originating near base of spermatheca. Spermathecal gland (**spg**) small, sausage-like, with bulbous swelling of duct, larger than gland, basad gland.

###### Geographical distribution.

Fig. 21. The known range of this species extends in the Greater Antilles from eastern Hispaniola, east to Puerto Rico and the Virgin Islands, and then southward through the Lesser Antilles to Grenada.

###### Chorological affinities and relationships.

The range of this species overlaps only that of *S. barbadensis* on the Lesser Antillean island of St. Vincent. Its relationships are not postulated beyond species group membership.

###### Material examined.

In addition to type material, we have seen a total of 131 specimens (58 males, 73 females). See Appendix for details.

##### Selenophorus solitarius

Taxon classificationAnimaliaColeopteraCarabidae

Darlington

Figs 18C , 19G–I , 21

Selenophorus solitarius Darlington, 1934: 106. HOLOTYPE male: Zaza del Medio, Cuba, Sept. 3, 1913 (AMNH). One female PARATYPE: Cayamas, Santa Clara, Cuba, Jan. 14, E.A. Schwarz (USNM).— Blackwelder 1944: 50.— Erwin and Sims 1984: 441.— Ball 1992: 85.— Lorenz 1998: 356.— Lorenz 2005: 377.— Peck 2005: 33.

###### Type locality.

Zaza del Medio, Sancti Spiritus Province, Cuba.

###### Diagnosis.

This species is readily separated from the other species in the *latior* species group by a combination of: elytra with mesh pattern slightly transverse, sculpticells about 1.5–2× wide as long, pronotum with posteriolateral angles rounded and pronotum with posteriolateral impressions coarsely punctate.

###### Descriptive notes.

Data for SBL in Table 1. Habitus as in Fig. 18C. Clypeus and labrum with anterior margin of each shallowly concave. Antennae with antennomere 1 rufo-testaceous to brunneous, antennomeres 2–11 darker; palpi infuscated, rufous to rufo-brunneous, tips testaceous; legs rufous to rufo-brunneous. Dorsal and ventral surfaces brunneous to brunneo-piceous; elytral epipleuron paler than disc. Male with faint bluish-green metallic luster; female with faint cupreous metallic luster. Male: head and pronotum shiny, few microlines visible at 100×. Female: head shiny, with mesh pattern isodiametric; pronotum shiny, few microlines visible at 100×. Elytra with mesh pattern slightly transverse, sculpticells about 1.5–2× wide as long in both sexes. Pronotum with posteriolateral impressions coarsely punctate; posteriolateral angles broadly rounded. Elytral striae impunctate, except the standard setigerous punctures in striae 2, 5 and 7. Both male and female with four terminal setae near the posterior margin on sternum VII.

**Male genitalia.** Figs 19G–I. Apical portion of phallic median lobe long, narrowly tapered, tip curved up dorsally, hook-like; endophallus without spines or dark microtrichial fields; lamina widened distally, tip pointed. Ventral surface of shaft smooth.

**Ovipositor and female reproductive tract.** Not studied.

###### Geographical distribution.

Fig. 21. This species is known only from the Greater Antillean island of Cuba.

###### Chorological affinities and relationships.

The range of this species is allopatric in relation to the other members of the *latior* species group. Its relationships are not specified beyond group membership.

###### Material examined.

In addition to the holotype, we have seen one female paratype. See Appendix for details.

##### Selenophorus seriatoporus

Taxon classificationAnimaliaColeopteraCarabidae

species group

###### Recognition.

Combination of the following characters: larger size (SBL 7.88 mm); elytra with mesh pattern isodiametric; and pronotum with posteriomedial area of disc impunctate.

**SBL.** Male, 7.88 mm.

**Color.** Antennae and legs rufo-testaceous to slightly darker; palpi infuscated, tip testaceous. Dorsal and ventral surfaces dark brunneous, not quite piceous; elytral epipleuron diffusely paler than disc.

**Luster.** Dull with faint metallic green reflection.

###### Dorsal microsculpture.

Head, pronotum and elytra with mesh pattern coarse isodiametric.

###### Male genitalia.

Apical portion of phallic median lobe with long taper, apex without hook. Preapical orifice anopic, moderately long; endophallus with macro spines, lamina present.

**Ovipositor and female reproductive tract**. Not studied.

###### Included species.

The *seriatoporus* species group includes only one species in the West Indies: *S. spinosus* sp. n..

###### Geographical distribution.

In the West Indies, this species group is known only from the Lesser Antillean island of Grenada. On the mainland, the species is known from Brazil.

##### Selenophorus spinosus

Taxon classificationAnimaliaColeopteraCarabidae

sp .n.

http://zoobank.org/B488CFC7-EA04-46FF-9E31-2671E2D61EF0

**Figure 22.**
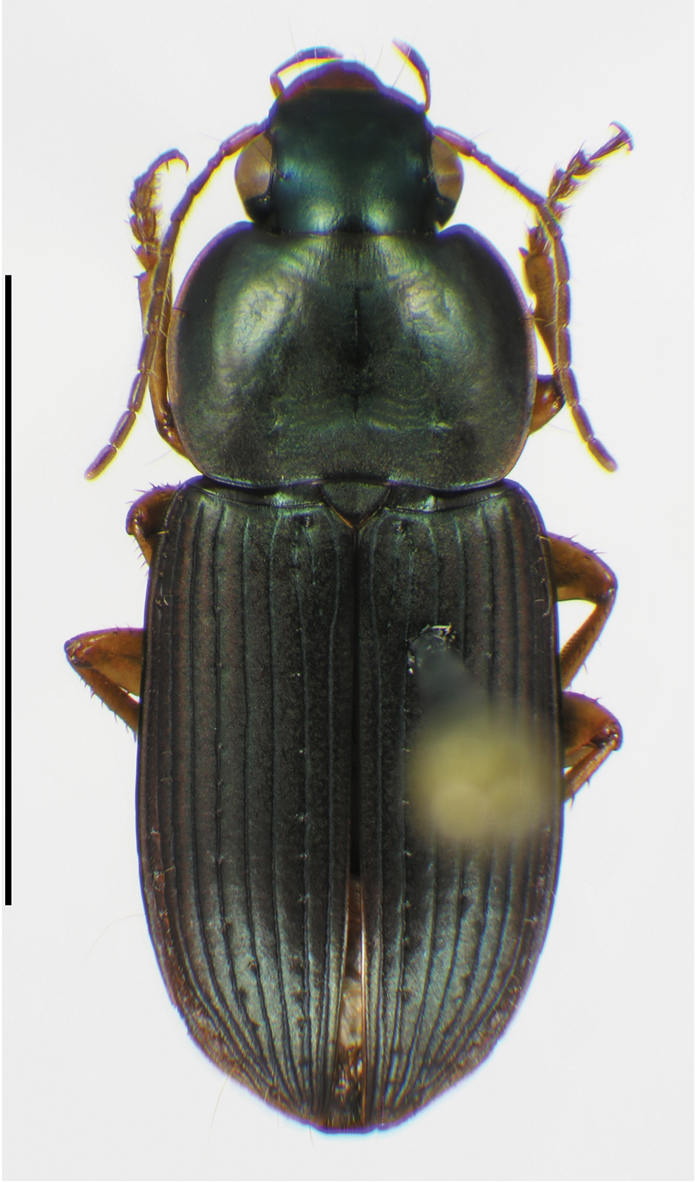
Habitus digital image of *Selenophorus seriatoporus* species group, dorsal aspect, *S. spinosus* sp. n.. Scale bar 5 mm.

**Figure 23.**
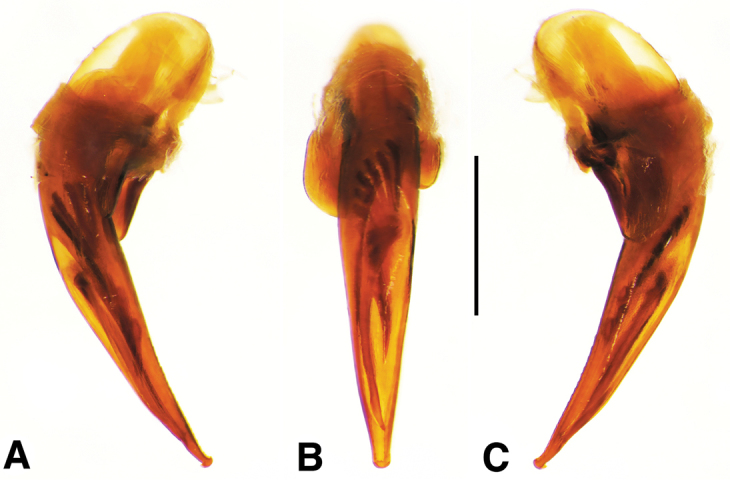
Digital images of male genitalia of *Selenophorus seriatoporus* species group, *S. spinosus* sp. n. **A** right lateral aspect **B** dorsal aspect **C** left lateral aspect. Scale bar 1 mm.

**Figure 24.**
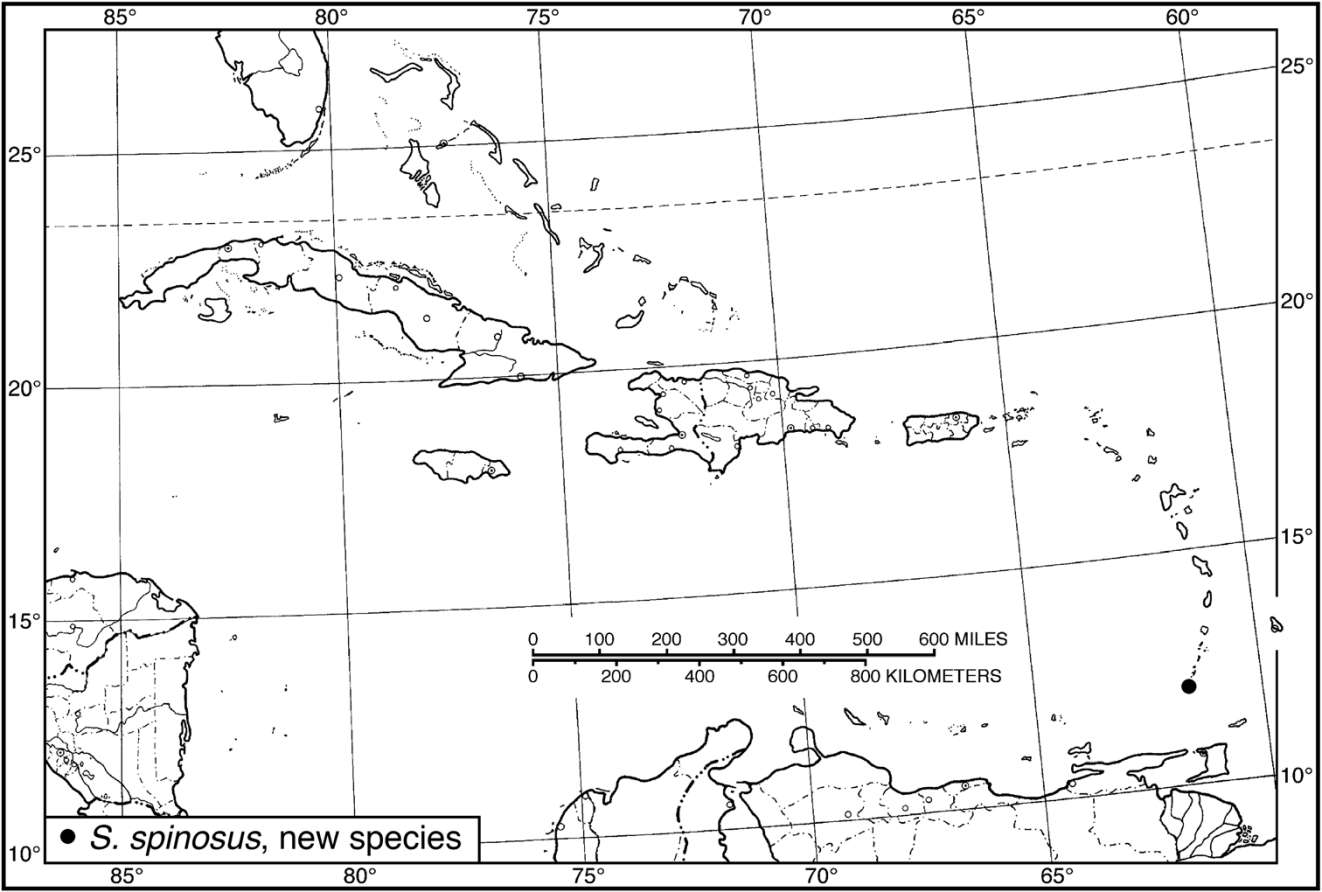
Map of West Indies showing known localities for species of *Selenophorus seriatoporus* species group.

Figs 22 , 23A–C , 24

###### Specific epithet.

From Latin, “*spina*”, in reference to the numerous large spines on the endophallus of the male genitalia.

###### Type material.

Seven specimens, 5 males, 2 females. HOLOTYPE male, labelled: “BRAZIL: Amazonas/ Benjamin Constant/ Rio Javary/ II-15-III-15-1942”; “August Robaus/ Collector” (AMNH). Six PARATYPES, sex and label data as follows. Three males, one female, labelled same as holotype (AMNH). Male, labelled “Rio Caiary-Uaupes,/ State of Amazonas,/ Brazil, IX 1906./ H. Schmidt.” (AMNH). Female, labelled “Rio Caiary-Uaupes,/ State of Amazonas,/ Brazil, 1906./ H. Schmidt.” (AMNH).

###### Type locality.

Benjamin Constant, state of Amazonas, Brazil.

###### Diagnosis.

This species, the only member of the *seriatoporus* species group in the West Indies, is readily recognized by a combination of large size, faint metallic green luster, broad pronotum with rounded posteriolateral angles and posteriolateral impressions smooth, or with only a few punctures. Additionally, endophallus with 13 long spines.

###### Descriptive notes.

Data for SBL in Table 1. Habitus as in Fig. 22. Labrum with anterior margin shallowly concave; clypeus with anterior margin moderately concave. Antennae and legs rufo-testaceous to slightly darker; palpi infuscated, tip testaceous, base darker, maxillary palpomere 3 same color as base of maxillary palpomere 4. Dorsal and ventral surface dark brunneous, with faint metallic green luster; elytral epipleuron diffusely paler than disc. Head, pronotum and elytra dull, with mesh pattern coarse isodiametric. Elytral striae impunctate, except the standard setigerous punctures in striae 2, 5 and 7. Punctures of striae 2, 5 and 7 foveate. Males with two terminal setae and females with four terminal setae near the posterior margin on sternum VII.

**Male genitalia.** Fig. 23A–C. Apical portion of phallic median lobe long, narrowly tapered, symmetrically rounded in dorsal/ventral aspect; endophallus with twisting row of 13 conspicuous long, thick spines; markedly long lamina present, banana-shaped; ostium anopic.

**Ovipositor and female reproductive tract.** Not studied.

###### Geographical distribution.

Fig. 24. This species is recorded only from the Lesser Antillean island of Grenada in the West Indies and from Brazil.

###### Chorological affinities and relationships.

The West Indian range of this species is overlapped by the ranges of its putative close relatives, *S. discopunctatus* and *S. yucatanus*.

###### Material examined.

In addition to the type material noted above, we have seen a single male specimen. See Appendix for details.

##### Subgenus Selenophorus

Taxon classificationAnimaliaColeopteraCarabidae

(sensu stricto)

###### Synonymy.

See *Selenophorus* (*sensu lato*), above.

###### Recognition.

Members of this subgenus have the hind tarsus distinctly shorter than the hind tibia. Additionally, males of all species in this subgenus do not have a lamina present near the base of the endophallus of the phallic median lobe. Identification of members is best done by using keys based on external structural features.

###### Included taxa.

Twenty-two species of subgenus Selenophorus, arranged in seven species groups, inhabit the West Indies.

##### Selenophorus hylacis

Taxon classificationAnimaliaColeopteraCarabidae

species group

###### Recognition.

Dorsal surface of tarsi with short setae; ventral surface of basitarsus of hind tarsus with inner row of spines touching each other, outer rows of spines more widely spaced. Species formerly placed in the genus *Gynandropus*, here treated as a species group of subgenus Selenophorus.

**SBL.** Males, 3.76–7.07 mm; females, 3.76–6.94 mm.

**Color.** Antennae variously colored: unicolorous testaceous; or with basal one to three antennomeres testaceous, remaining antennomeres darker. Mouthparts testaceous. Legs testaceous to rufo-testaceous. Dorsal and ventral surfaces rufo-brunneous to brunneo-piceous; elytra unicolorous or bicolored, with dark discal cloud.

**Luster.** Shiny, with or without faint iridescence.

**Dorsal microsculpture.** Microlines not visible at 100× on head and pronotum. Elytra with mesh pattern transverse, sculpticells about 3–4× wide as long.

**Male genitalia.** Apical portion of phallic median lobe moderately long and wide. Preapical orifice anopic, moderately long; endophallus variously armored with spines and/or darkened microtrichial fields, or without spines or darkened microtrichial fields, without lamina.

**Ovipositor and female reproductive tract.** Only *S. dessalinesi* and *S. parvus* were examined. Bursa copulatrix moderately short; spermatheca moderately long to long, with apical portion coiled, originating near base of common oviduct; moderately long to markedly long spermathecal gland duct originating well above base of spermatheca. Spermathecal gland small, bulbous, without swelling of duct basad gland.

###### Included species.

The West Indian members of the *hylacis* species group includes five species: *S. clypealis* Ball and Shpeley, *S. dubius* Putzeys, *S. dessalinesi* Ball and Shpeley, *S. parvus* Darlington and *S. subquadratus* (Putzeys).

###### Geographical distribution.

The range of this species group extends in the Greater Antilles from Cuba to the Virgin Islands and through the Lesser Antilles to Grenada.

##### Selenophorus clypealis

Taxon classificationAnimaliaColeopteraCarabidae

Ball & Shpeley

**Figure 25.**
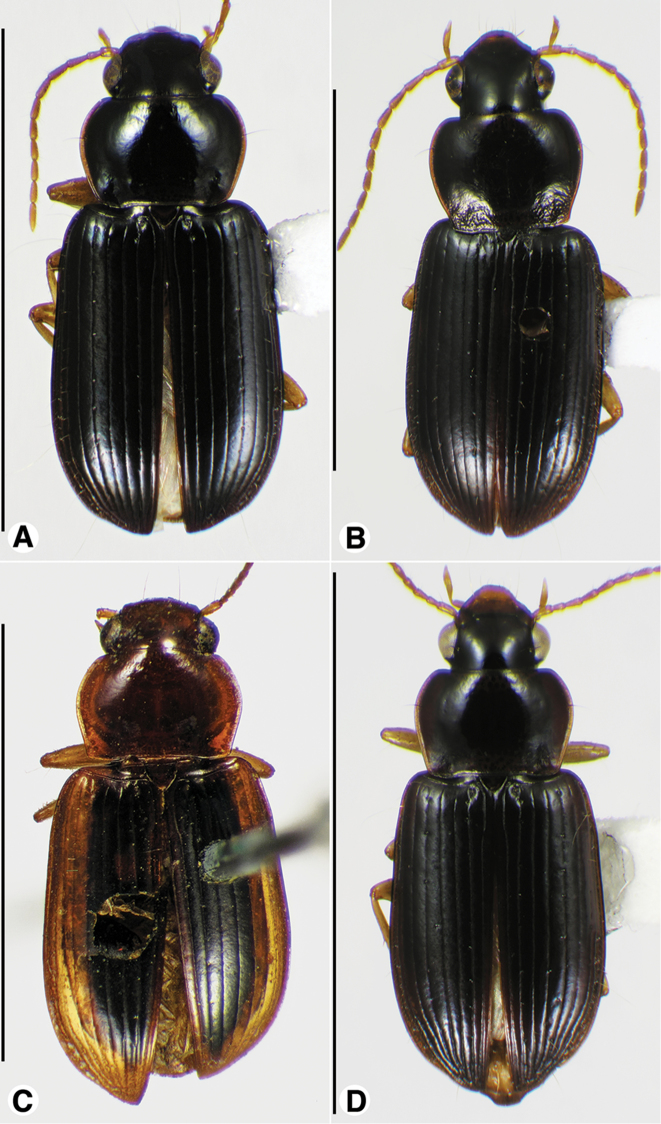
Habitus digital images of *Selenophorus hylacis* species group, in part, dorsal aspect. **A** *S. clypealis* Ball & Shpeley **B** *S. dessalinesi* Ball & Shpeley **C** *S. dubius* Putzeys **D** *parvus* Darlington. Scale bars 5 mm.

Figs 25A , 27A–C , 30

Selenophorus clypealis Ball & Shpeley, 1992: 101.— Ball 1992: 85.— Lorenz 1998: 355.—Lorenz 2005: 376.— Perez-Gelabert 2008: 79.

###### Type material.

Complete label data for type material (holotype (MCZC) and allotype (WIBF)) are provided in the original description.

###### Type locality.

Source of the Matelas (River), near Ennery, Artibonite Department, Haiti, Hispaniola.

###### Diagnosis.

This species is readily separated from the other four members of the *hylacis* species group on a combination of: clypeus with anterior margin markedly concave, small size and pronotum with hind angles rounded.

###### Descriptive notes.

Data for SBL in Table 1. Habitus as in Fig. 25A. Labrum with anterior margin deeply notched; clypeus with anterior margin markedly concave. Antennae, mouthparts and legs testaceous. Dorsal and ventral surfaces rufo-brunneous to brunneo-piceous; lateral bead of pronotum paler. Head and pronotum shiny, microlines not visible at 100×. Elytra shiny, with mesh pattern transverse, transverse microlines just visible at 100×; iridescent, brighter than observed in *S. parvus*. Pronotum with posteriolateral impressions impunctate; posteriolateral angles rounded. Elytral striae impunctate, except the standard setigerous punctures in striae 2, 5 and 7. Males with adhesive vestiture on tarsomeres 1–4 of fore- and mid-tarsi; females without adhesive vestiture on tarsomeres 1–4 of fore- and mid-tarsi. Tarsomere 1 of fore- and mid-tarsus in females not expanded. Both males and females with four terminal setae near the posterior margin on sternum VII.

**Male genitalia.** Fig. 27A–C. Apical portion of phallic median lobe moderately long, trapezoidal, symmetrically broadly rounded in dorsal/ventral aspect, with narrow dorsal flange; endophallus medially with three patches of short, thin spines, one darkened microtrichial field near basal bulb in left lateral aspect; without lamina; ostium anopic. Ventral surface of shaft smooth.

**Ovipositor and female reproductive tract.** Very similar to *S. dessalinesi*, Fig. 29A. For details, see this topic for *S. dessalinesi*, below.

###### Geographical distribution.

Fig. 30. This species is known only from the Greater Antillean island of Hispaniola and the island of Little St. James in the Virgin Islands.

###### Chorological affinities and relationships.

Within the West Indian *hylacis* species group, the range of *S. clypealis* is overlapped by the ranges of *S. subquadratus* and *S. dessalinesi*. Relationships of *S. clypealis* are not postulated beyond species group membership.

###### Material examined.

In addition to type material, we have seen a total of 6 specimens (1 male, 5 females). See Appendix for details.

**Figure 26.**
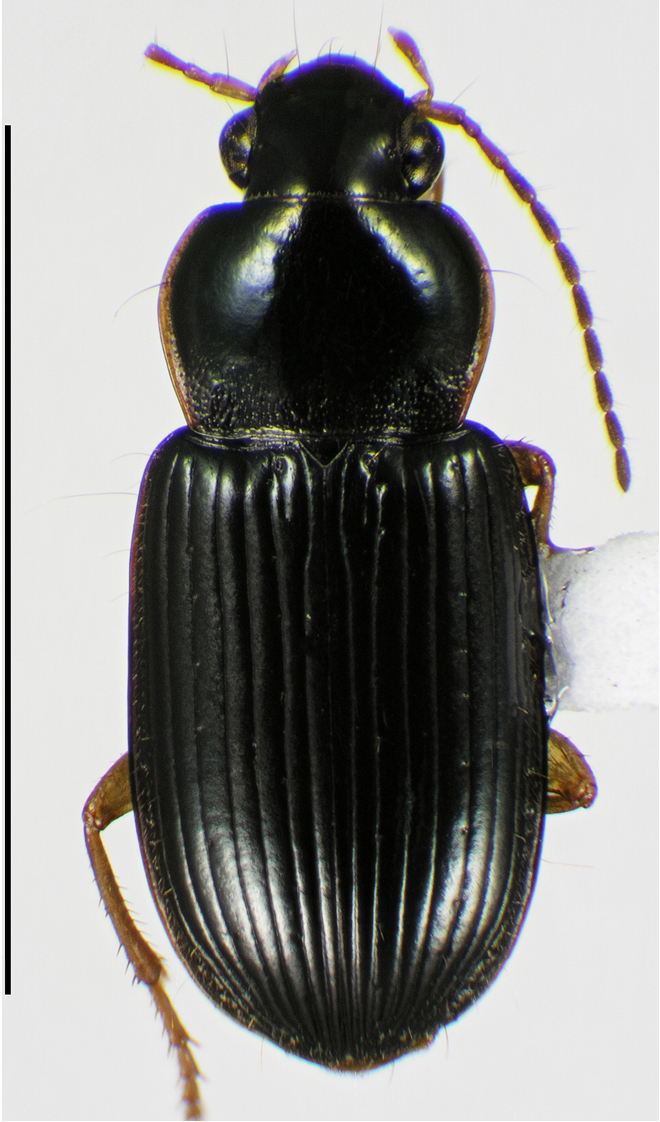
Habitus digital image of *Selenophorus hylacis* species group, in part, dorsal aspect, *S. subquadratus* (Putzeys). Scale bar 5 mm.

**Figure 27.**
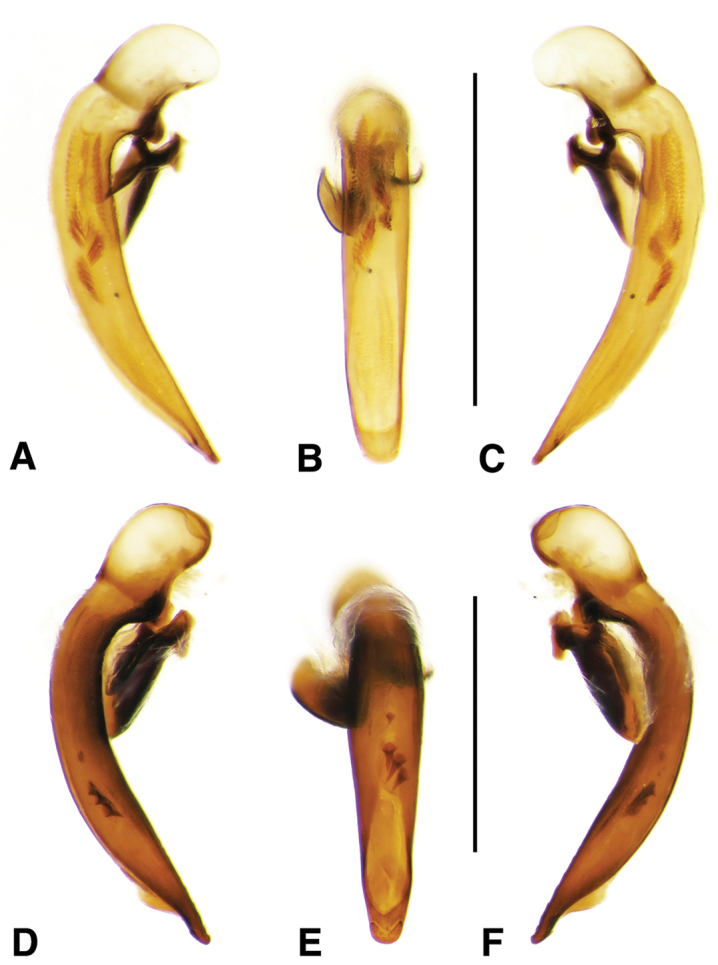
Digital images of male genitalia of *Selenophorus hylacis* species group, in part. **A, D** right lateral aspect **B, E** dorsal aspect **C, F** left lateral aspect **A–C** *S. clypealis* Ball & Shpeley **D–F** *S. dessalinesi* Ball & Shpeley. Scale bars 1 mm.

**Figure 28.**
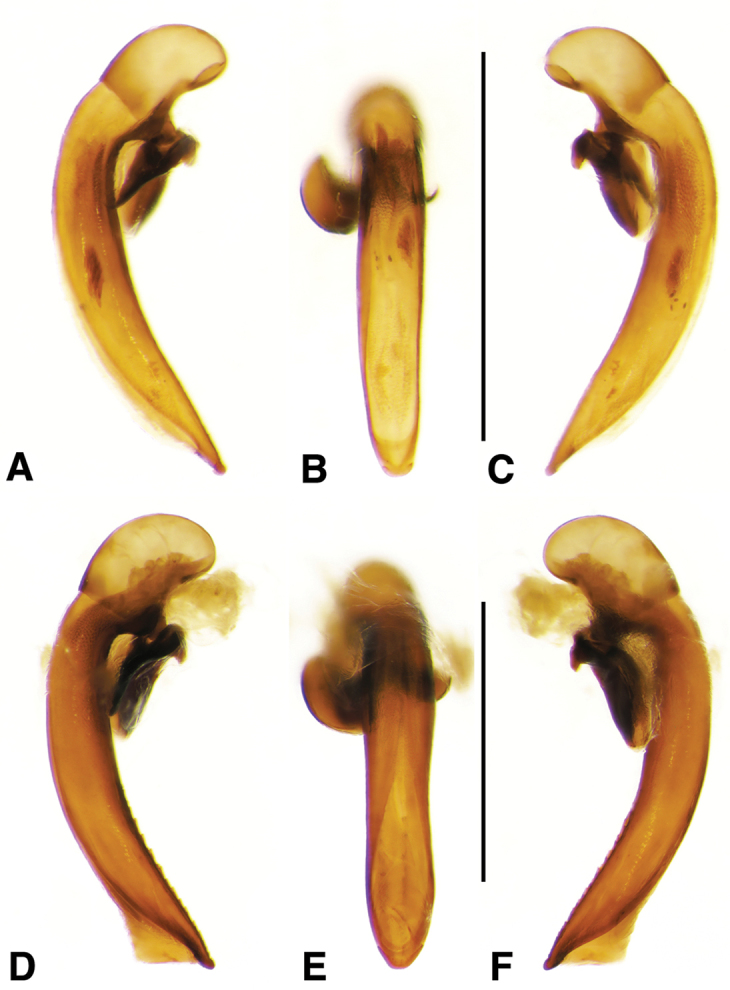
Digital images of male genitalia of *Selenophorus hylacis* species group, in part. **A, D** right lateral aspect **B, E** dorsal aspect **C, F** left lateral aspect **A–C** *S. parvus* Darlington **D–F** *S. subquadratus* (Putzeys). Scale bars 1 mm.

**Figure 29.**
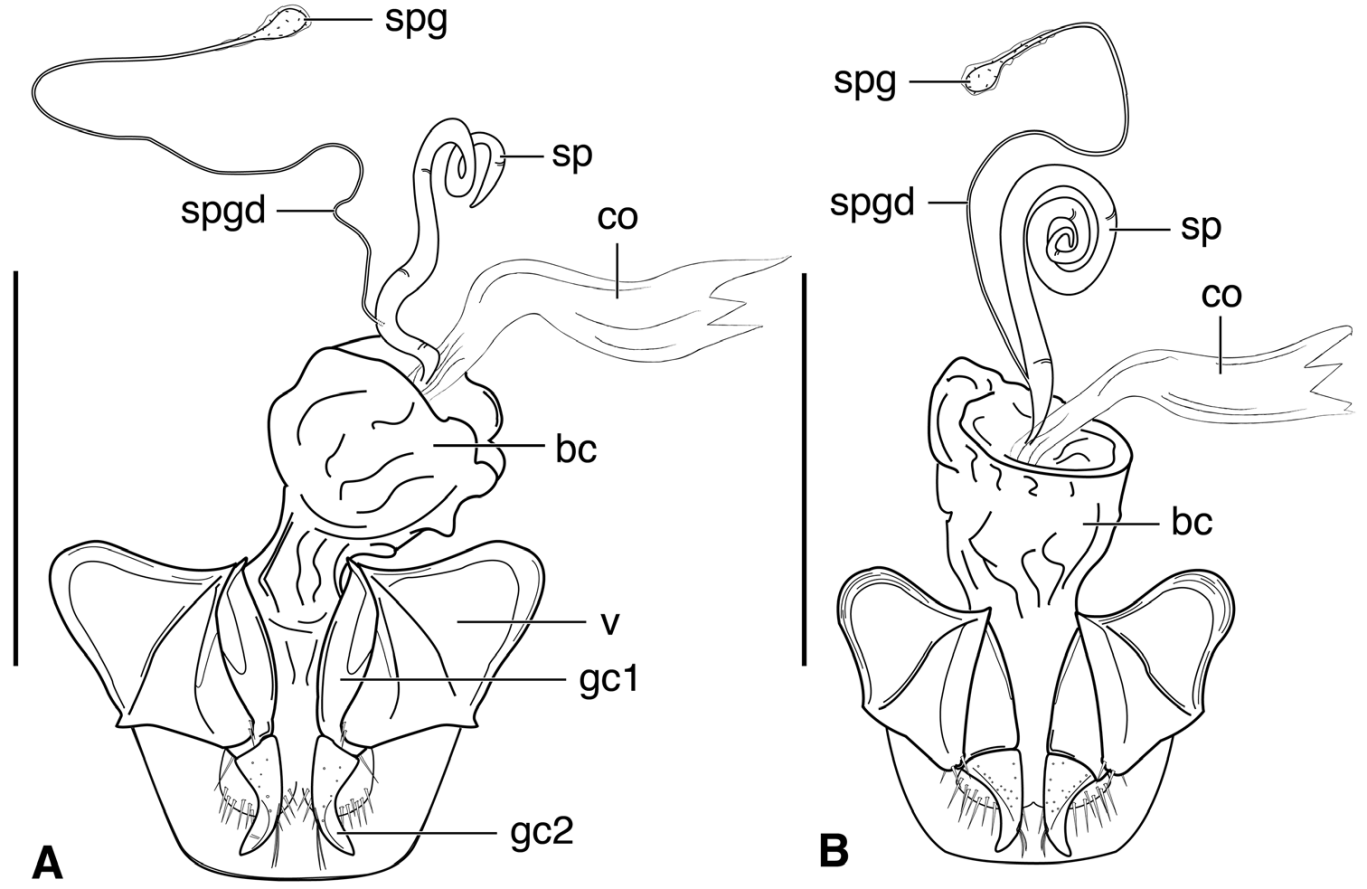
Line drawings of female reproductive tract of *Selenophorus hylacis* species group, in part, ventral aspect. **A** *S. dessalinesi* Ball & Shpeley **B** *S. parvus* Darlington. Legend: **bc** bursa copulatrix **co** common oviduct **gc1** gonocoxite 1 **gc2** gonocoxite 2 **sp** spermatheca **sp1** spermatheca 1 **sp2** spermatheca 2 **spg** spermathecal gland **spgd** spermathecal gland duct; **v** valvifer. Scale bars 1 mm.

**Figure 30.**
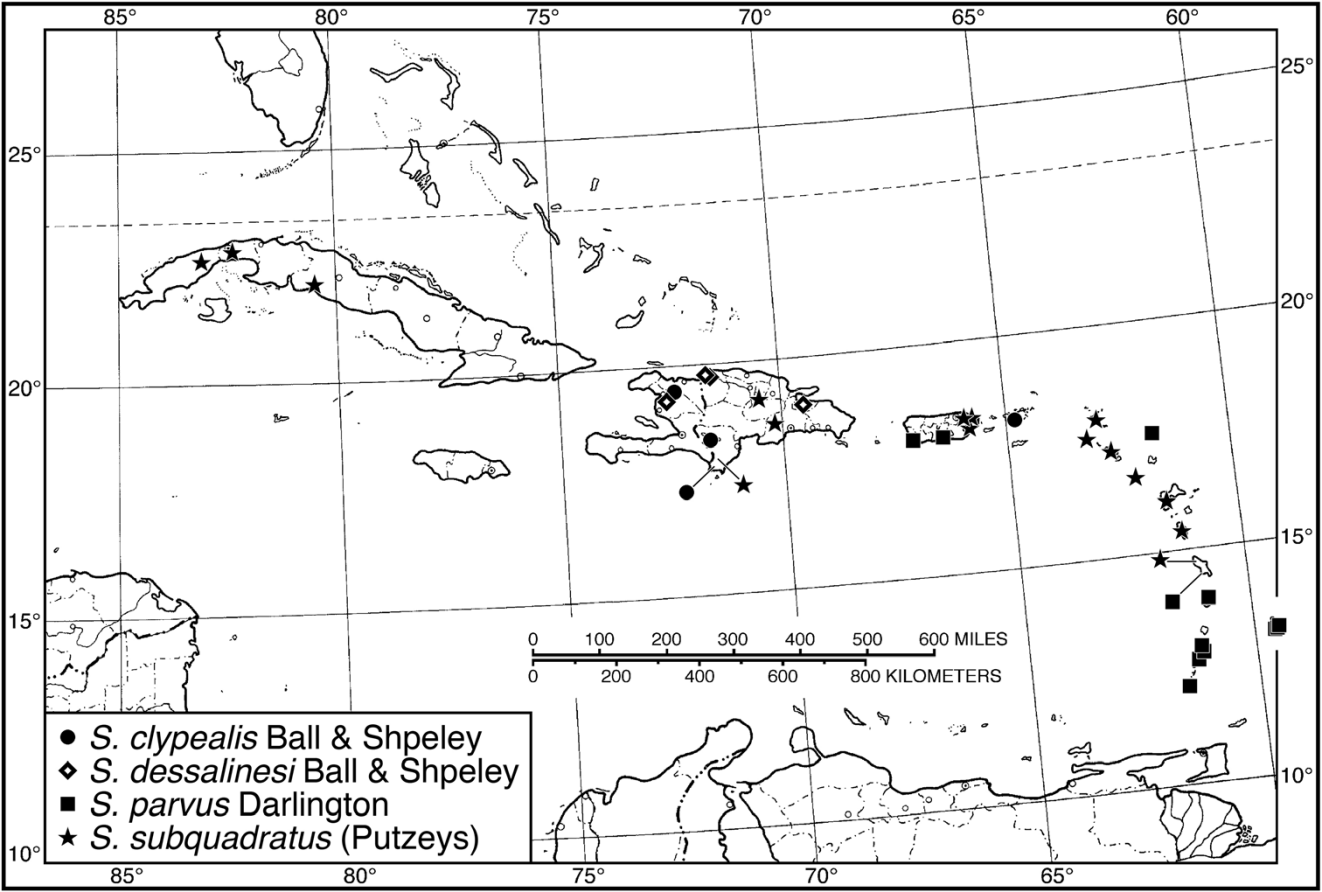
Map of West Indies showing known localities for species of *Selenophorus hylacis* species group.

##### Selenophorus dessalinesi

Taxon classificationAnimaliaColeopteraCarabidae

Ball & Shpeley

Figs 25B , 27D–F , 29A , 30

Selenophorus dessalinesi Ball & Shpeley, 1992: 102.— Ball 1992: 85.— Lorenz 1998: 355.— Lorenz 2005: 377.— Perez-Gelabert 2008: 79.

###### Type material.

Complete label data for type material (holotype (MCZC), allotype and 5 paratypes) are provided in the original description.

###### Type locality.

Just north of Dessalines, Artibonite Department, Haiti, Hispaniola.

###### Diagnosis.

This species is readily separated from the other three West Indian members of the *hylacis* species group on a combination of: larger size and subcordate pronotum with nearly rectangular posteriolateral angles.

###### Descriptive notes.

Data for SBL in Table 1. Habitus as in Fig. 25B. Clypeus with anterior margin moderately concave. Labrum with anterior margin shallowly concave. Antennae and mouthparts testaceous; legs testaceous to rufo-testaceous. Dorsal surface dark brunneous to brunneo-piceous, lateral bead of pronotum paler. Ventral surface rufo-brunneous to dark brunneous. Elytra with very faint iridescence. Head and pronotum shiny, microlines not visible at 100×. Elytra with mesh pattern slightly transverse, sculpticells about 3–4× wide as long. Pronotum with posteriolateral impressions punctate; posteriolateral angles rectangular. Elytral striae impunctate, except the standard setigerous punctures in striae 2, 5 and 7. Both males and females with adhesive vestiture on tarsomeres 1–4 of fore- and mid-tarsi. Tarsomere 1 of fore-tarsus of females expanded, about 2× the width of tarsomere 2, adhesive vestiture dense, not biseriate. Tarsomere 1 of mid-tarsus of females less expanded, about 1.5× the width of tarsomere 2, adhesive vestiture dense, not biseriate. Both males and females with four terminal setae near the posterior margin on sternum VII.

**Male genitalia.** Fig. 27D–F. Apical portion of phallic median lobe moderately long, narrowly tapered, symmetrically rounded in dorsal/ventral aspect, with dorsal flange; endophallus, apicad of medial, with two rows of short, stout spines, three spines on the left and four spines on the right, without darkened microtrichial fields; without lamina; ostium anopic. Ventral surface of shaft with two rows of basad directed fine saw-toothed ridges.

**Ovipositor and female reproductive tract.** Fig. 29A. Gonocoxite 2 (**gc2**) moderately thick, falcate. Bursa copulatrix (**bc**) moderately short; spermatheca (**sp**) long, with apical portion coiled, spring-like, originating near base of common oviduct (**co**); markedly long spermathecal gland duct (**spgd**) originating well above base of spermatheca. Spermathecal gland (**spg**) small, bulbous, without swelling of duct basad gland.

###### Geographical distribution.

Fig. 30. This species is known only from the type locality in Haiti and Monte Cristi in the northwest corner of the Dominican Republic.

###### Chorological affinities and relationships.

Within the *hylacis* species group, the range of *S. dessalinesi* is overlapped only by the range of *S. clypealis*. Relationships of *S. dessalinesi* are not postulated beyond species group membership.

###### Material examined.

In addition to type material, we have seen a total of 9 specimens (8 males, 1 female). See Appendix for details.

##### Selenophorus dubius

Taxon classificationAnimaliaColeopteraCarabidae

Putzeys

Fig. 25C

Selenophorus dubius Putzeys, 1878a: 54. HOLOTYPE, female (unlabelled): Chaudoir-Oberthür Collection (MNHP), in front of following box label: “dubius/ Chaud/ Espagne mer?”.— Csiki 1932: 1198.— Darlington 1934: 104.— Blackwelder 1944: 49.— Erwin and Sims 1984: 440.— Lorenz 1998: 356.— Lorenz 2005: 377.

###### Note regarding type locality.

Putzeys (1878a) in his original description stated that the specimen was from “Espagne meridionale” (southernmost Spain). In his next sentence, Putzeys stated that he believed that this specimen was “Antillean” (West Indies). Csiki (1932) listed this species from “? Antillean”, Darlington (1934) followed with “doubtfully Antillean” and both Blackwelder (1944) and Erwin and Sims (1984) simply listed it as “West Indies”. Until another specimen is found, neither a type locality nor a type area can be designated.

We have seen two undetermined *Selenophorus* (*hylacis* species group) specimens, both different species, collected in Brazil, one from São Paulo and the other from the Federal District, that are quite similar in habitus and coloration to the holotype of *S. dubius*. Even though the holotype of *S. dubius* is missing the hind tarsi, we believe that this species is a member of the *hylacis* species group.

###### Descriptive notes.

SBL 5.78 mm. Habitus as in Fig. 25C. Clypeus and labrum each with anterior margin slightly concave. Antennae, mouthparts and legs testaceous to slightly darker. Head and pronotum rufo-testaceous; ventral surface rufo-testaceous, markedly infuscated medially. Elytra bicolored, rufo-testaceous, with darker median cloud in intervals 2–5, and in basal half of interval 1. Head and disc of pronotum shiny, no microlines visible at 100×; elytral with mesh pattern moderately transverse, sculpticells about 3–4× wide as long. Elytral striae impunctate, except the standard setigerous punctures in striae 2, 5 and 7. Female with four terminal setae near the posterior margin on sternum VII.

**Male genitalia.** Not known.

**Ovipositor and female reproductive tract.** Not studied.

###### Geographical distribution.

The locality of this species is unknown, and this species may not even be in the West Indies (see note about type locality above).

###### Chorological affinities and relationships.

We are unable to comment on these topics due to the unknown locality of this species.

###### Material examined.

Holotype only.

##### Selenophorus parvus

Taxon classificationAnimaliaColeopteraCarabidae

Darlington

Figs 25D , 28A–C , 29B , 30

Selenophorus parvus Darlington, 1934: 105. HOLOTYPE, male: Coamo Springs, Puerto Rico, Sept. 28, 1929, S.T. Danforth (MCZC).— Woodruff 1944: 50.— Erwin and Sims 1984: 440.— Bennett and Alam 1985: 20.— Ball 1992: 85.— Lorenz 1998: 356.— Lorenz 2005: 377.— Peck 2009: 13.

###### Type locality.

Coamo Springs, Coamo Municipality, Puerto Rico.

###### Diagnosis.

This species is readily separated from the other members of the *hylacis* species group by a combination of: small size and pronotum with obtuse hind angles.

###### Descriptive notes.

Data for SBL in Table 1. Habitus as in Fig. 25D. Clypeus with anterior margin moderately concave. Labrum with anterior margin shallowly concave. Antennae with antennomeres 1–3 testaceous, antennomeres 4–11 darker; mouthparts and legs testaceous. Dorsal and ventral surfaces rufo-brunneous to brunneo-piceous; lateral bead of pronotum paler. Head shiny, microlines not visible at 100× in males, just visible at 100× as isodiametric mesh pattern in females; pronotum shiny, microlines not visible at 100×. Elytra shiny, with mesh pattern transverse, sculpticells about 3–4× wide as long; slightly iridescent, less than observed in *S. clypealis*. Pronotum with posteriolateral impressions impunctate; posteriolateral angles obtuse. Elytral striae impunctate, except the standard setigerous punctures in striae 2, 5 and 7. Males with adhesive vestiture on tarsomeres 1–4 of fore- and mid-tarsi; females without adhesive vestiture on tarsomeres 1–4 of fore- and mid-tarsi. Tarsomere 1 of fore- and mid-tarsus in females not expanded. Both males and females with four terminal setae near the posterior margin on sternum VII.

**Male genitalia.** Fig. 28A–C. Apical portion of phallic median lobe moderately long, symmetrically broadly rounded in dorsal/ventral aspect; endophallus with one field of short, thin spines medially, a few scattered shorter spines near apex; without lamina; ostium anopic. Ventral surface of shaft smooth.

**Ovipositor and female reproductive tract.** Fig. 29B. Gonocoxite 2 falcate, with wide base. Bursa copulatrix moderately short; spermatheca (**sp**) moderately long, with apical portion coiled, originating near base of common oviduct; moderately long spermathecal gland duct originating well above base of spermatheca. Spermathecal gland (**spg**) small, bulbous, without swelling of duct basad gland.

###### Geographical distribution.

Fig. 30. The range of this species includes the Greater Antillean island of Puerto Rico, and the Lesser Antillean islands of Barbuda, Martinique, St. Lucia, Barbados, Bequia, Mustique, Canouan and Grenada.

###### Chorological affinities and relationships.

Within the species of the *hylacis* species group, the range of *S. parvus* is overlapped by the range of *S. subquadratus*. However, with the exception of Puerto Rico, the two species have not been recorded from the same island within their respective ranges. Relationships of *S. parvus* are not postulated beyond species group membership.

###### Material examined.

In addition to type material, we have seen a total of 5,451 specimens (2,412 males, 3,040 females). See Appendix for details.

##### Selenophorus subquadratus

Taxon classificationAnimaliaColeopteraCarabidae

(Putzeys)

Figs 26 , 28D–F , 30

Gynandropus subquadratus Putzeys, 1878b: 293. LECTOTYPE: in Chaudoir-Oberthür collection (MNHP); male in front of following box label: Haiti//; specimen labelled: Haiti C. Chd [green paper] //; [blank oblong piece of paper]// Soc. Ent. Belg// Coll. Putzeys/ Type//.— Csiki 1932: 1195.—Blackwelder 1944: 48.
Gynandropus guadeloupensis Fleutiaux & Sallé, 1889: 365. TYPE MATERIAL: 3 specimens, 2 males and 1 female in Fleutiaux Collection (MNHP). LECTOTYPE: first male, labelled: Type// Guadeloupe/ Delauney// Gynandropus/ guadeloupen/sis Fleutiaux et Sallé type/ obscuricornis (Chd); second male and female, each labelled Guadeloupe/ Vitrac.
Selenophorus subquadratus ; Erwin & Sims, 1984: 441.— Ball 1992: 85.— Ball and Shpeley 1992: 96.— Lorenz 1998: 356.— Lorenz 2005: 377.— Peck 2006: 176.— Ivie et al. 2008: 238.— Perez-Gelabert 2008: 80.
Selenophorus guadeloupensis ; Ball & Shpeley, 1992: 96.

###### Note.

Noted above is the name “*Gynandropus obscuricornis* (Chd)”. It is a junior secondary homonym of *Selenophorus obscuricornis* Waterhouse, and was re-named *Selenophorus neobscuricornis* by Noonan (1985a: 40).

###### Type locality.

“Tablasco” in the Greater Antillean island of Hispaniola.

###### Diagnosis.

This species is readily separated from the other three West Indian members of the *hylacis* species group on a combination of: intermediate size, pronotum with obtuse posteriolateral angles and pronotum with posteriolateral impressions punctate.

###### Descriptive notes.

Data for SBL in Table 1. Habitus as in Fig. 26. Clypeus and labrum with anterior margin of each shallowly concave. Antennae with antennomeres 1 or 1and 2 testaceous, antennomeres 2–11 or 3–11 darker. Mouthparts and legs testaceous. Dorsal surface dark brunneous to brunneo-piceous, lateral bead of pronotum paler. Ventral surface rufo-brunneous to dark brunneous. Elytra with very faint iridescence. Head and pronotum shiny, microlines not visible at 100×. Elytra with mesh pattern slightly transverse, sculpticells about 3–4× wide as long. Pronotum with posteriolateral impressions punctate; posteriolateral angles obtuse. Elytral striae impunctate, except the standard setigerous punctures in striae 2, 5 and 7. Both males and females with adhesive vestiture on tarsomeres 1–4 of fore- and mid-tarsi. Tarsomere 1 of fore-tarsus of females expanded, about 1.5× the width of tarsomere 2, adhesive vestiture dense, not biseriate. Tarsomere 1 of mid-tarsus of females less expanded, about same width as tarsomere 2, adhesive vestiture dense, not biseriate. Both males and females with four terminal setae near the posterior margin on sternum VII.

**Male genitalia.** Fig. 28D–F. Apical portion of phallic median lobe short, broad, symmetrically rounded in dorsal/ventral aspect; endophallus without spines or darkened microtrichial fields; without lamina; ostium anopic. Ventral surface of shaft with two rows of basally directed saw-toothed ridges.

**Ovipositor and female reproductive tract.** Very similar to *S. dessalinesi*, Fig. 29A. For details, see this topic for *S. dessalinesi*, above.

###### Geographical distribution.

Fig. 30. The known range of this species extends eastward from Greater Antillean Cuba to Puerto Rico, and then in the Lesser Antilles southward from St. Barthélemy and Saba to Martinique.

###### Chorological affinities and relationships.

The range of this species overlaps the ranges of the other three West Indian members of the *hylacis* species group. Relationships of *S. subquadratus* are not postulated beyond species group membership.

###### Material examined.

In addition to type material, we have seen a total of 65 specimens (41 males, 24 females). See Appendix for details.

##### Selenophorus mundus

Taxon classificationAnimaliaColeopteraCarabidae

species group

###### Recognition.

Small species, shiny, with faint to moderate metallic luster, posteriolateral angles of pronotum moderately coarsely punctate or impunctate.

**SBL.** Males, 3.60–4.60 mm; females, 3.82–5.32 mm.

**Color.** Antennae testaceous to rufo-testaceous or with one, two or three basal antennomeres testaceous, remaining antennomeres darker. Mouthparts and legs testaceous. Head and pronotum rufo-brunneous to dark brunneous; elytra brunneous to brunneo-piceous; elytral epipleuron paler than disc.

**Luster.** Pronotum with bluish metallic luster or without metallic luster. Elytra with greenish iridescence or with very faint to moderate cupreous metallic luster.

**Dorsal microsculpture.** Head and pronotum shiny, microlines not visible at 100× or microlines visible at 100×, isodiametric on head, slightly transverse on pronotum, sculpticells about 1.5–2× wide as long. Elytra shiny, microlines not visible at 100×, or with mesh pattern transverse, sculpticells about 2–4× wide as long.

**Male genitalia.** Apical portion of phallic median lobe moderately long, broadly triangular, symmetrically rounded in dorsal/ventral aspect, tip curved up dorsally; endophallus without spines or dark microtrichial fields; without lamina. Ventral surface of shaft smooth.

**Ovipositor and female reproductive tract**. Gonocoxite 2 moderately thick, somewhat falcate. Bursa copulatrix short; spermatheca sausage-like, originating near base of common oviduct; moderately long to long spermathecal gland duct originating near or below mid-length of spermatheca. Spermathecal gland small, bulbous, with swelling of duct, larger than gland, basad gland.

###### Included species.

The *mundus* species group includes three species: *S. mundus* Putzeys, *S. paramundus* Ball and Shpeley and *S. pseudomundus* Ball and Shpeley.

###### Geographical distribution.

This species group is known only from the Greater Antillean islands of Hispaniola and Jamaica.

##### Selenophorus mundus

Taxon classificationAnimaliaColeopteraCarabidae

Putzeys

**Figure 31.**
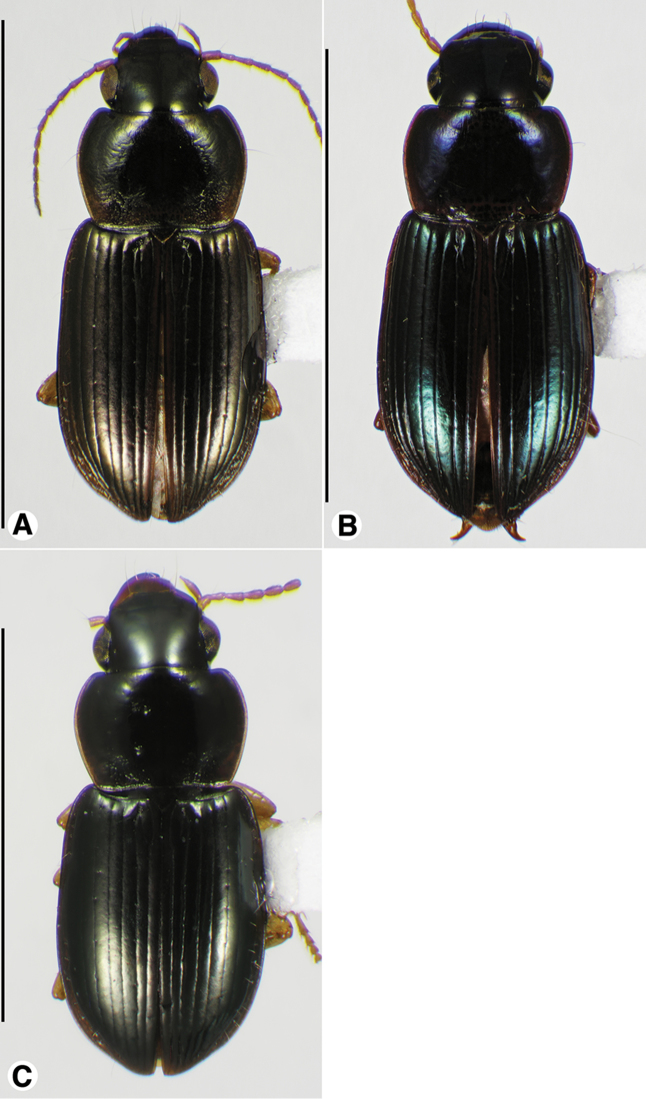
Habitus digital images of *Selenophorus mundus* species group, dorsal aspect. **A** *S. mundus* Putzeys **B** *S. paramundus* Ball & Shpeley **C** *S. pseudomundus* Ball & Shpeley. Scale bars: **A, B** 5 mm **C** 3 mm.

**Figure 32.**
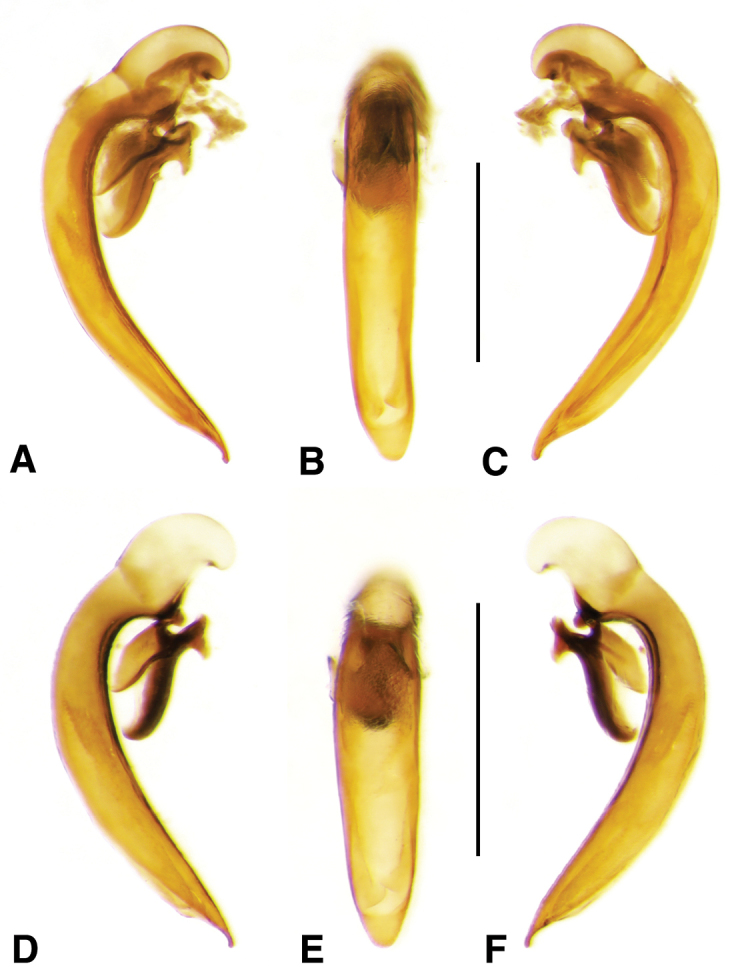
Digital images of male genitalia of *Selenophorus mundus* species group, in part. **A, D** and **G** right lateral aspect **B, E, H** dorsal aspect **C, F, I** left lateral aspect **A–C** *S. mundus* Putzeys **D–F** *S. pseudomundus* Ball & Shpeley. Scale bars 0.5 mm.

**Figure 33.**
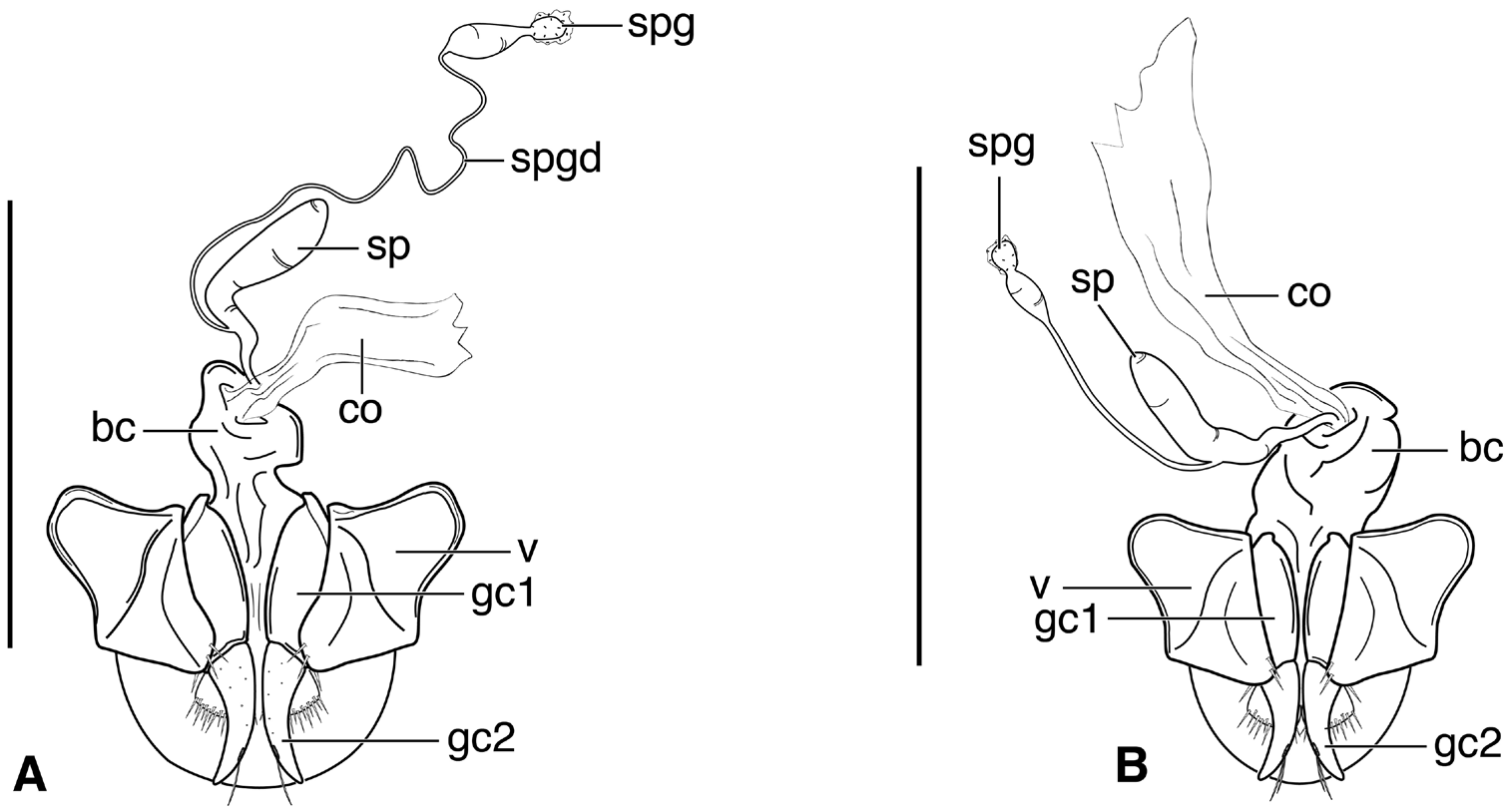
Line drawings of female reproductive tract of *Selenophorus mundus* species group, in part, ventral aspect. **A** *S. mundus* Putzeys **B** *S. pseudomundus* Ball & Shpeley. Legend: **bc** bursa copulatrix **co** common oviduct **gc1** gonocoxite 1 **gc2** gonocoxite 2 **sp** spermatheca **spg** spermathecal gland **spgd** spermathecal gland duct; **v** valvifer. Scale bars 1 mm.

**Figure 34.**
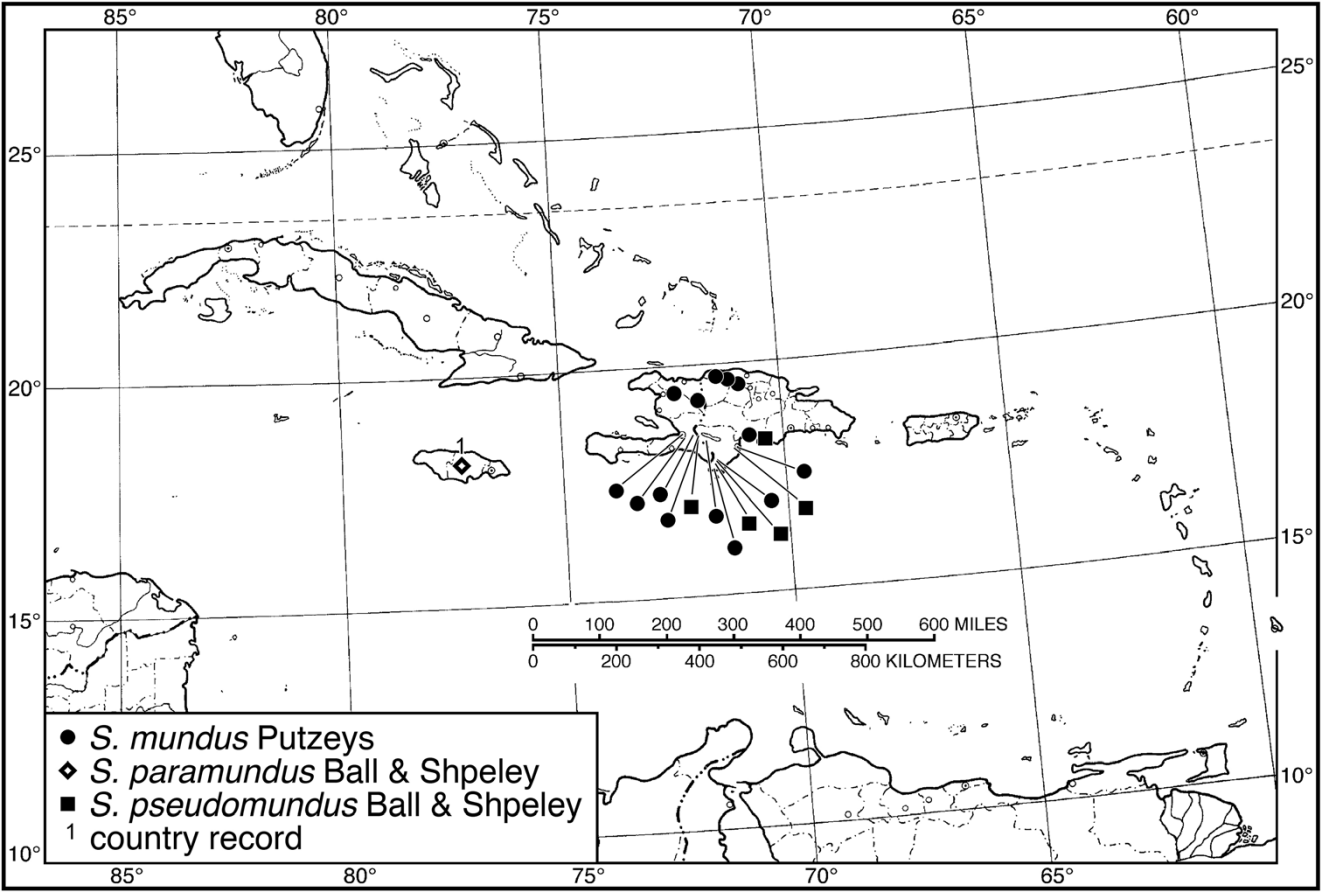
Map of West Indies showing known localities for species of *Selenophorus mundus* species group.

Figs 31A , 32A–C , 33A , 34

Selenophorus mundus Putzeys, 1878a: 29. In Chaudoir-Oberthür Collection, a single specimen, HOLOTYPE, female (unlabelled), handwritten label to right of specimen, //mundus? van Emden//, in front of the following box label: // insularis/ Chaud./ Antilles/ Jamaique? Jaeger [? illegible]// [MNHP].— Csiki 1932: 1199.— Darlington 1934: 105.— Blackwelder 1944: 50.— Erwin and Sims 1984: 440.— Ball 1992: 86.— Ball and Shpeley 1992: 96.— Lorenz 1998: 355.— Lorenz 2005: 377.—Perez-Gelabert 2008: 79.
Selenophorus haitianus Darlington, 1934: 107. HOLOTYPE female: Manneville, Haiti, W.M. Mann (MCZC). One female PARATYPE: Pont Beudet, Haiti, March 3–4, 1922, ca. 100' (AMNH).— Ball and Shpeley 1992: 96.

###### Type area.

“Antilles” (Putzeys 1878a: 29), here restricted to the Greater Antillean island of Hispaniola.

###### Diagnosis.

This species is readily separated from the other species in the *mundus* species group by a combination of: elytra with slightly transverse microsculpture, sculpticells about 2–4× wide as long, pronotum with posteriolateral angles obtuse and posteriolateral impressions finely punctate.

###### Descriptive notes.

Data for SBL in Table 1. Habitus as in Fig. 31A. Clypeus and labrum with anterior margin of each shallowly concave. Antennae with antennomeres 1, 1–2 or 1–3 testaceous, antennomeres 2–11, 3–11 or 4–11 darker. Mouthparts and legs testaceous. Head and pronotum rufo-brunneous to dark brunneous; elytra brunneous to brunneo-piceous, with very faint cupreous metallic luster. Ventral surface rufo-brunneous to dark brunneous; elytral epipleuron paler than disc. Head and pronotum shiny, microlines visible at 100×, isodiametric on head, slightly transverse on pronotum, sculpticells about 1.5–2× wide as long; elytra with mesh pattern transverse, sculpticells about 2–4× wide as long. Pronotum with posteriolateral impressions moderately coarsely punctate; posteriolateral angles rounded. Elytral striae impunctate, except the standard setigerous punctures in striae 2, 5 and 7. Males with two terminal setae and females with four terminal setae near the posterior margin on sternum VII.

**Male genitalia.** Fig. 32A–C. Apical portion of phallic median lobe moderately long, broadly triangular, symmetrically rounded in dorsal/ventral aspect, tip curved up dorsally; endophallus without spines or dark microtrichial fields; without lamina. Ventral surface of shaft smooth.

**Ovipositor and female reproductive tract.** Fig. 33A. Gonocoxite 2 (**gc2**) moderately thick, somewhat falcate. Bursa copulatrix (**bc**) short; spermatheca (**sp**) sausage-like, originating near base of common oviduct (**co**); moderately long spermathecal gland duct (**spgd**) originating below mid-length of spermatheca. Spermathecal gland (**spg**) small, bulbous, with swelling of duct, about twice the size of the gland, basad gland.

###### Geographical distribution.

Fig. 34. This species is restricted to the Greater Antillean island of Hispaniola.

###### Chorological affinities and relationships.

The range of this species is overlapped by the range of *S. pseudomundus*. Relationships of *S. mundus* are not postulated beyond species group membership.

###### Material examined.

In addition to type material, we have seen a total of 57 specimens (28 males, 29 females). See Appendix for details.

##### Selenophorus paramundus

Taxon classificationAnimaliaColeopteraCarabidae

Ball & Shpeley

Figs 31B , 34

Selenophorus paramundus Ball & Shpeley, 1992: 98.— Ball 1992: 85.— Lorenz 1998: 356.— Lorenz 2005: 377.

###### Type material.

Complete label data for type material (holotype (BMNH)) are provided in the original description.

###### Type area.

Jamaica.

###### Diagnosis.

This species is readily separated from other members of the *mundus* species group by a combination of: dorsal surface without visible microlines and pronotum with posteriolateral impressions impunctate.

###### Descriptive notes.

Data for SBL in Table 1. Habitus as in Fig. 31B. Labrum with anterior margin shallowly concave; clypeus with anterior margin moderately concave. Antennae and mouthparts testaceous to rufo-testaceous; legs rufo-brunneous. Dorsal surface dark brunneous; ventral surface rufo-brunneous, elytral epipleuron paler than disc. Pronotum with bluish metallic luster; elytra with greenish iridescence. Head, pronotum and elytra shiny, microlines not visible at 100×. Pronotum with posteriolateral impressions impunctate; posteriolateral angles rounded. Elytral striae impunctate, except the standard setigerous punctures in striae 2, 5 and 7. Female with four terminal setae near the posterior margin on sternum VII.

**Male genitalia.** Male unknown.

**Ovipositor and Female Reproductive Tract**: Very similar to that of *S. pseudomundus* below, except the spermathecal gland duct is shorter, such that the distal tip of the spermathecal gland is just past the distal tip of the spermatheca.

###### Geographical distribution.

Fig. 34. This species is known only from Jamaica.

###### Chorological affinities and relationships.

The range of this species is allopatric relative to the other species in the *mundus* species group. The form of the female reproductive tract suggests that this species belongs in the *mundus* species group. If a male of the species is collected, the form of the male genitalia will either confirm or refute this placement. Relationships of *S. paramundus* are not postulated beyond species group membership.

###### Material examined.

Only the female holotype.

##### Selenophorus pseudomundus

Taxon classificationAnimaliaColeopteraCarabidae

Ball & Shpeley

Figs 31C , 32D–F , 33B , 34

Selenophorus pseudomundus Ball & Shpeley, 1992: 99.— Ball 1992: 85.— Lorenz 1998: 356.— Lorenz 2005: 377.— Perez-Gelabert 2008: 80.

###### Type material.

Complete label data for type material (holotype (CMNH), allotype, and 8 paratypes) are provided in the original description.

###### Type locality.

Las Mercedes, Pedernales Province, Dominican Republic.

###### Diagnosis.

This species is readily separated from the other species in the *mundus* species group by a combination of: elytra with slightly transverse microsculpture, sculpticells about 2–4× wide as long and head and pronotum shiny, without visible microlines.

###### Descriptive notes.

Data for SBL in Table 1. Habitus as in Fig. 31C. Clypeus and labrum with anterior margin of each shallowly concave. Antennae and mouthparts testaceous to rufo-testaceous; legs testaceous. Dorsal and ventral surfaces brunneous to dark brunneous; elytral epipleuron paler than disc. Elytra with cupreous metallic luster. Head and pronotum shiny, microlines not visible at 100×; elytra with mesh pattern transverse, sculpticells about 2–4× wide as long. Pronotum with posteriolateral impressions moderately coarsely punctate; posteriolateral angles rounded. Elytral striae impunctate, except the standard setigerous punctures in striae 2, 5 and 7. Males with two terminal setae and females with four terminal setae near the posterior margin on sternum VII.

**Male genitalia.** Fig. 32D–F. Apical portion of phallic median lobe moderately long, broadly triangular, symmetrically rounded in dorsal/ventral aspect, tip curved up dorsally; endophallus without spines or dark microtrichial fields; without lamina. Ventral surface of shaft smooth.

**Ovipositor and female reproductive tract.** Fig. 33B. Gonocoxite 2 moderately thick, somewhat falcate. Bursa copulatrix short; spermatheca (**sp**) sausage-like, originating near base of common oviduct; long spermathecal gland duct originating about mid-length of spermatheca. Spermathecal gland (**spg**) small, bulbous, with swelling of duct, larger than gland, basad gland.

###### Geographical distribution.

Fig. 34. This species is known only from the Greater Antillean Island of Hispaniola, specifically the southwestern regions of the Dominican Republic.

###### Chorological affinities and relationships.

The range of this species is overlapped by the range of *S. mundus*. Relationships of *S. pseudomundus* are not postulated beyond species group membership.

###### Material examined.

In addition to type material, we have seen a total of 40 specimens (19 males, 21 females). See Appendix for details.

##### Selenophorus nonseriatus

Taxon classificationAnimaliaColeopteraCarabidae

species group

###### Recognition.

Small species without parascutellar stria, elytral punctures very small (*i.e.*, easily overlooked) and female internal genitalia with spermathecal basal sclerite.

**SBL.** Males, 4.00–4.92 mm; females, 4.24–5.32 mm.

**Color.** Antennae and mouthparts testaceous to slightly darker rufo-testaceous. Legs testaceous to slightly darker rufo-testaceous, tarsi darker than tibia or not. Dorsal surfaces rufo-brunneous to piceous, lateral bead of pronotum paler or not. Ventral surface rufo-brunneous to brunneo-piceous, elytral epipleuron paler.

**Luster.** Shiny, with faint to moderate iridescence.

**Dorsal microsculpture.** Microlines not visible at 100× on head, prontum and elytra.

###### Male genitalia.

Males of *S. irec* are not known. Apical portion of phallic median lobe symmetrically rounded in dorsal/ventral aspect; preapical orifice anopic, moderately long; endophallus with two dark, dense microtrichial fields nearly the length of the phallic median lobe, left dorsal markedly long, medial ventral slightly shorter; without lamina.

**Ovipositor and female reproductive tract.** Gonocoxite 2 somewhat falcate, moderately wide base. Bursa copulatrix short to markedly long; moderately to markedly long spermatheca, originating near base of common oviduct; melanized spermathecal basal sclerite present, rather short to nearly half as long as spermatheca; moderately to markedly long spermathecal gland duct originating near mid-length of spermatheca apicad to spermathecal basal sclerite. Spermathecal gland bulbous to sausage-like.

###### Included species.

In the West Indies, the *nonseriatus* species group includes three species: *S. irec* sp. n., *S. iviei* sp. n., and *S. nonseriatus* Darlington.

###### Geographical distribution.

In the West Indies, the range of this species group extends from the Greater Antillean islands of Cuba, Jamaica and Hispaniola to the Lesser Antillean islands of Montserrat to Grenada.

##### Selenophorus irec sp. n.

Taxon classificationAnimaliaColeopteraCarabidae

http://zoobank.org/FFE632E6-4BD4-41A5-AA3D-2E0C96A4C002

Figs 35A , 37A , 38

###### Specific epithet.

Based on the coden “IREC” for the Institut de Recherches Entomologique de la Caribe, from which the type specimens were borrowed for this project.

###### Type material.

HOLOTYPE female, labelled: “Guadeloupe/ Vernou/ 10.8.71 Chalumeau” [IREC]. PARATYPE female, labelled: “Guadeloupe/ Vernou/ 14.9.73 Chalumeau” [IREC].

###### Type locality.

Vernou, Guadeloupe, Lesser Antilles.

###### Diagnosis.

This species is readily separated from the other two species in the *nonseriatus* species group by a combination of: broad pronotum with rectangular posteriolateral angles and elytral intervals distinctly convex.

###### Descriptive notes.

**Figure 35.**
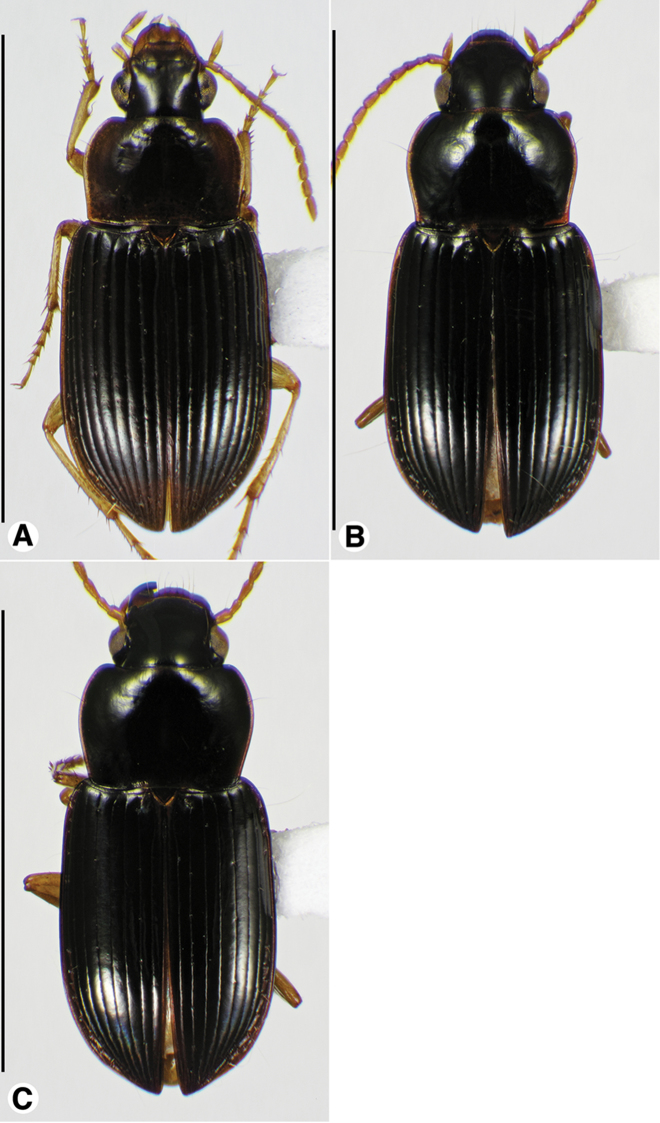
Habitus digital images of *Selenophorus nonseriatus* species group, dorsal aspect. **A** *S. irec* sp. n. **B** *S. iviei* sp. n. **C** *S. nonseriatus* Darlington. Scale bars 5 mm.

Data for SBL in Table 1. Habitus as in Fig. 35A. Antennae, mouthparts and legs testaceous. Dorsal and ventral surfaces rufo-brunneous; elytral epipleuron paler. Elytra and ventral surface with faint bluish iridescence. Head, pronotum and elytra shiny, microlines not visible at 100×. Pronotum with posteriolateral impressions with only a few fine punctures next to shallow longitudinal fovea; posteriolateral angles rectangular. Elytral intervals distinctly convex. Elytral striae impunctate, except the standard setigerous punctures in striae 2, 5 and 7. Female with four terminal setae near the posterior margin on sternum VII.

**Male genitalia.** Not known.

**Ovipositor and female reproductive tract.** Fig. 37A. Gonocoxite 2 (**gc2**) somewhat falcate, moderately wide base. Bursa copulatrix (**bc**) short; moderately long spermatheca (**bc**) originating near base of common oviduct (**co**); melanized spermathecal basal sclerite (**sbs**) present, rather short; markedly long spermathecal gland duct (**spgd**) originating below mid-length of spermatheca apicad to spermathecal basal sclerite. Spermathecal gland (**spg**) sausage-like.

###### Geographical distribution.

Fig. 38. This species is known only from the island of Guadeloupe in the Lesser Antilles.

###### Chorological affinities and relationships.

The range of this species overlaps the ranges of the other two species in the *nonseriatus* species group, though neither of the two has been collected on the island of Guadeloupe. Relationships of *S. irec* are not postulated beyond species group membership.

###### Material examined.

Type material only; for details, see above.

**Figure 36.**
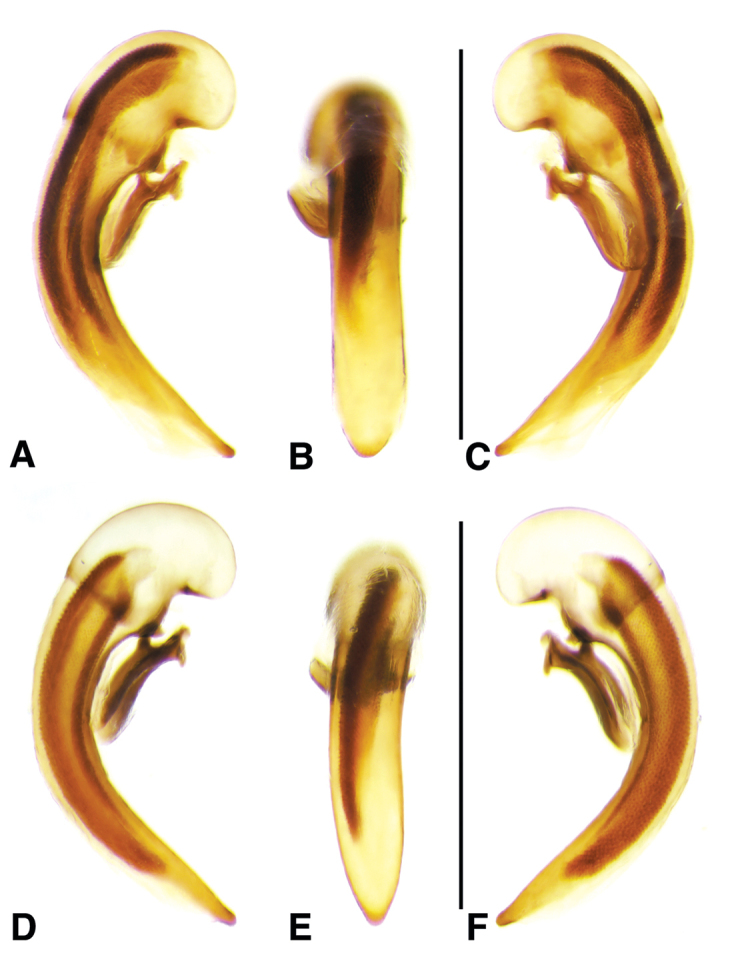
Digital images of male genitalia of *Selenophorus nonseriatus* species group, in part. **A, D** right lateral aspect **B, E** dorsal aspect **C, F** left lateral aspect **A–C** *S. iviei* sp. n. **D–F** *S. nonseriatus* Darlington. Scale bars 1 mm.

**Figure 37.**
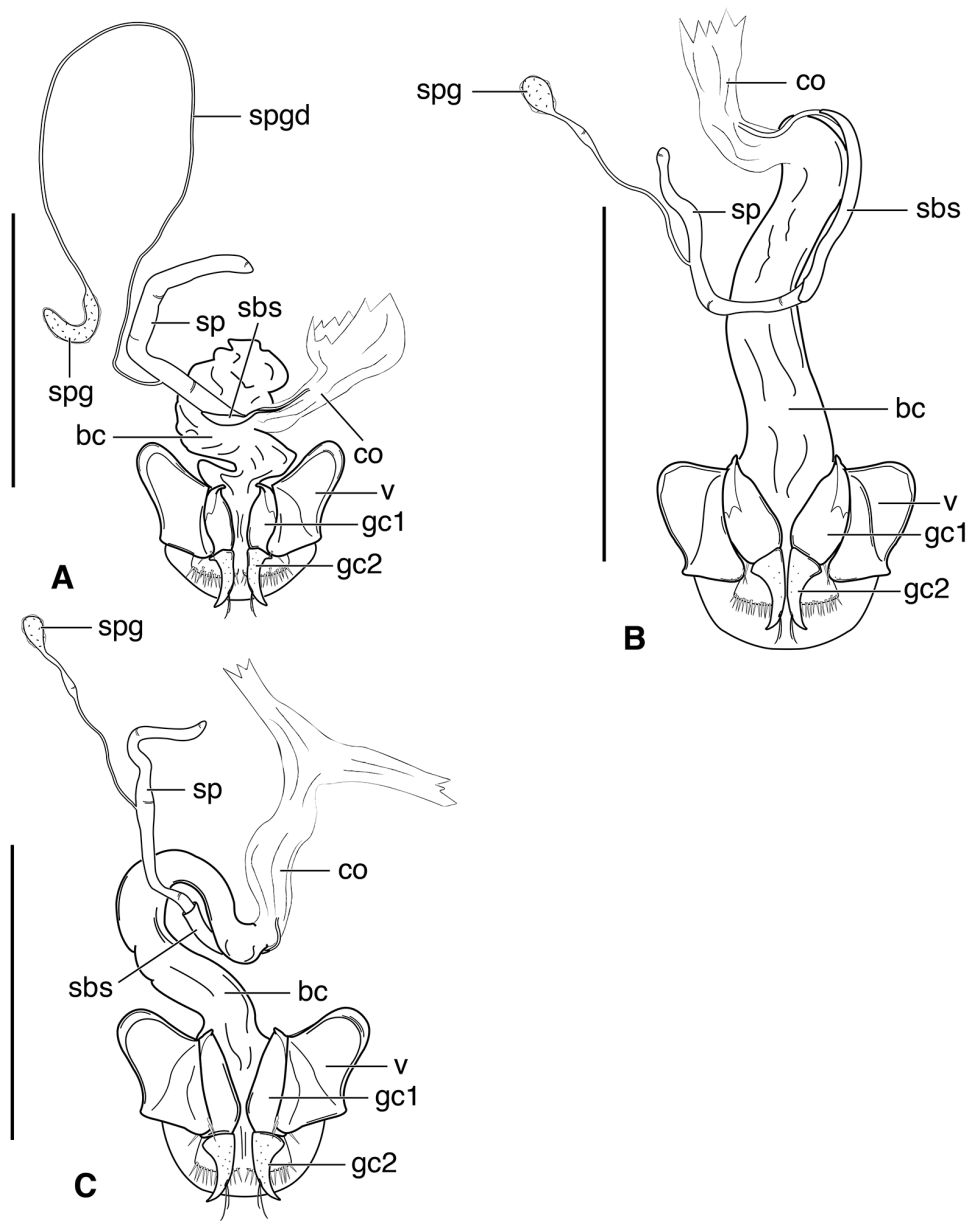
Line drawings of female reproductive tract of *Selenophorus nonseriatus* species group, ventral aspect. **A** *S. irec* sp. n. Dejean **B** *S. ivei* sp. n. **C** *S. nonseriatus* Darlington. Legend: **bc** bursa copulatrix **co** common oviduct **gc1** gonocoxite 1 **gc2** gonocoxite 2 **sbs** spermathecal basal sclerite **sp** spermatheca; **spg** spermathecal gland **spgd** spermathecal gland duct; **v** valvifer. Scale bars 1 mm.

**Figure 38.**
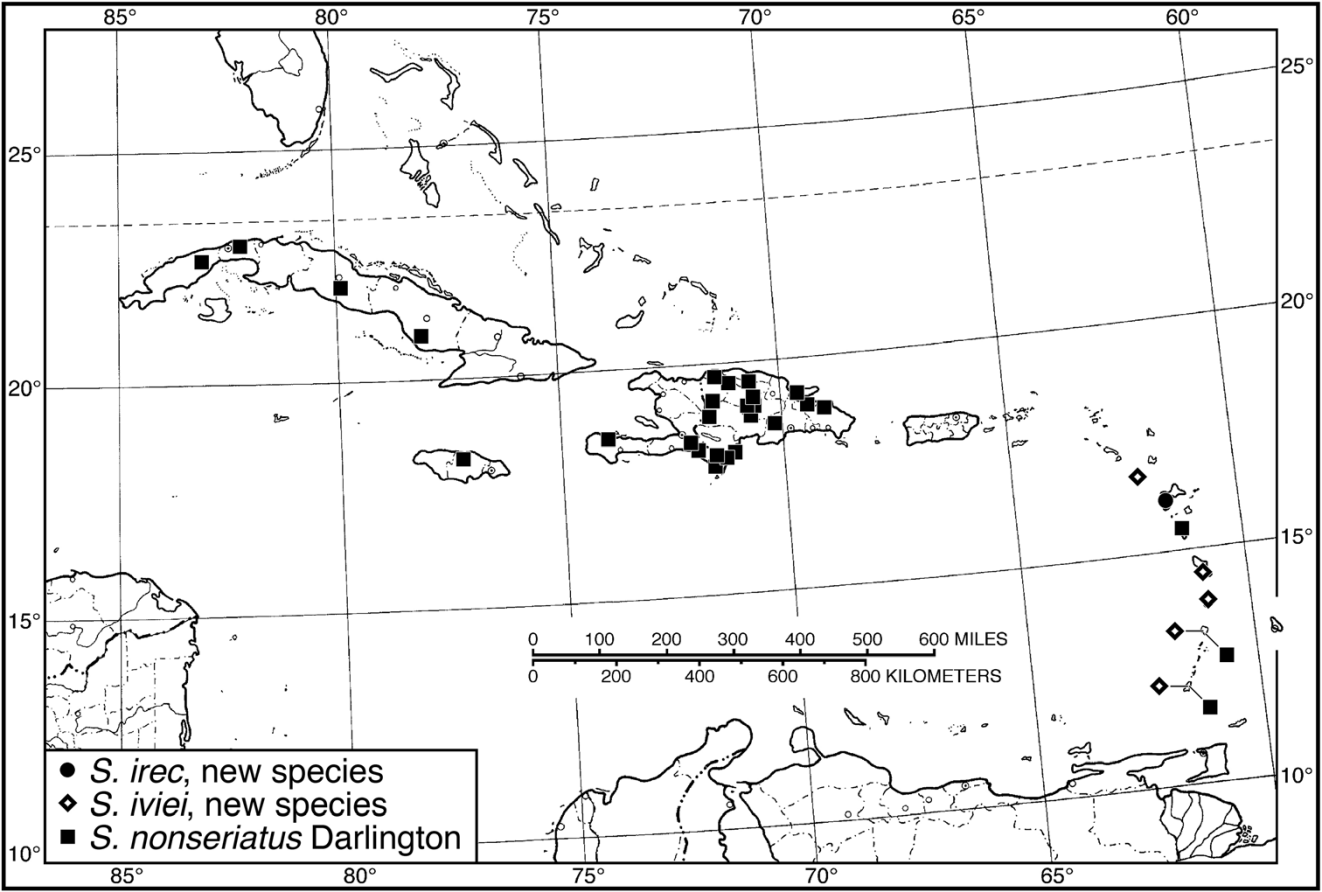
Map of West Indies showing known localities for species of *Selenophorus nonseriatus* species group.

##### Selenophorus iviei sp. n.

Taxon classificationAnimaliaColeopteraCarabidae

http://zoobank.org/C0B02316-6879-4F44-8CE3-4A847781B31C

Figs 35B , 36A–C , 37B , 38

###### Specific epithet.

A Latinized eponym, genitive case, based on the surname of Michael A. Ivie, Department of Entomology, Montana State University, Bozeman, Montana who collected not only the type series of this species, but many other carabid species during his extensive field work in the West Indies.

###### Type material.

42 specimens. HOLOTYPE male, “Montserrat:Big River/ 16°45.719'N, 62°11.335'W/ 05 JULY 2005, 1230ft/ I. A. Foley colr” (WIBF, to de deposited in USNM). PARATYPES, 41: 1 female: “Montserrat: Big River/ 16°45.719'N, 62°11.335'W/ 05 JULY 2005, 1230ft/ I. A. Foley colr (WIBF, to de deposited in USNM). 1 male: “Montserrat: Centre Hills/ Jubilee Heights, 1600'/ 20JUNE2002, mesic forest/ M.A. Ivie & K.A. Guerrero/ Berlese leaf litter” (WIBF, to de deposited in USNM). 1 male: “Montserrat: Centre Hills/ Cassava Ghaut, 800'/ 16°45.944'N, 62°12.727'W/ 22 JUNE 2000,/ M.A. Ivie & K.A. Guerrero” (WIBF, to de deposited in USNM). 1 male: “Montserrat: trail to/ Katy Hill just below/ heli pad, 2300 ft/ 11-14 AUG 2005/ WIBF group/ uv light trap” (WIBF). 1 male: “Montserrat:/ Cassava Ghaut/ 29MAR-11JUN2002/ K.A.Marske colr./ baited pitfall” (WIBF). 3 males, 1 female: “Montserrat:/ Cassava Ghaut/ 29 MAY 2002/ K. A. Marske colr./ berlese leaf litter” (WIBF). 1 female: “Montserrat:/ Cassava Ghaut/ 28 MAY 2002/ K. A. Marske colr./ berlese leaf litter” (WIBF). 1 male: “Montserrat:/ Cassava Ghaut/ 18 JUNE 2002/ K. A. Marske colr./ berlese leaf litter” (WIBF). 1 male: “Montserrat: Hope Ghaut/16°45.347'N, 62°12.560'W/ 26 JUNE 2002, 315m/ M. Ivie & K. Marske/ at night” (WIBF). 1 female: “Montserrat:/ Gun Hill/ 16JUNE-07JULY2002/ K. A. Marske/ F.I.T. & pitfall” (WIBF, to de deposited in USNM). 2 females: “Montserrat:/ Jubilee Heights/ 04 JAN 2002/ K. A. Marske colr./ Heliconia leaf litter” (WIBF). 1 female: “Montserrat:/ Jubilee Heights/ 16°45.393'N, 62°12.58'W/ 1441ft, 10JULY2003/ K. A. Marske, leaf litter” (10 of date handwritten over 08) (WIBF). 1 female: “Montserrat:/ Jubilee Heights/ 04 JUNE 2002/ K. A. Marske” (WIBF). 4 males, 3 females: “MARTINIQUE: Morne/ Constant, Diamant,/ 14.50836 -61.02125,/ intercept trap,/ 10.X.2015, E. Poirier/ & J. Tourlout (SEAG)” (JMLC). 1 male, 1 female, same as previous: (UASM). 1 male: “ST.LUCIA:Barre de L’Isle/ 13.9368°N, 60.9593°W 340m/ 03-08JULY2009,uvlight/ M.L. Gimmel” (WIBF). 1 female: “ST:LUCIA:Barre de/ l’Isle trap site/ 13.9368°N, 60.9594°W 25-28JUNE2009, 340m/E.A.Ivie,uv light” (WIBF). 4 females: “ ST.LUCIA:Barre de L’Isle/ 13.93682°N, 60.95936°W/ 340m,08 JULY 2009/ M.L. Gimmel colr/ at uv light” (WIBF). 2 males: “ST.LUCIA:Barre de L’Isle/ 13.93682°N, 60.95936°W/ 29JUNE-03JULY2009,340m/uv light trap/ C.A. Maier,M.L. Gimmel” (WIBF). 1 male: “ST. LUCIA:Quielles For.Res/ LaPorte cabin, 272m/ 13.84041°N, 60.97408°W/ 05-07 MAY 2009,uv light/ I.A.Foley and R.C.Winton” (WIBF). 1 female: “ST. LUCIA:Quielles For.Res/ LaPorte cabin, 272m/ 13.84041°N, 60.97408°W/ 10 MAY 2009,uvlight/ R.C.Winton and I.A.Foley” (WIBF). 1 male: “ST. LUCIA:Ravine Chabot/ 14.0010°N, 60.9734°W,62m/ 06JULY2009,litter berlese/ K.J. Hopp & M.L.Gimmel” (WIBF). 1 female: “WEST INDIES: St. Vincent/ Hermitage Forest, E of Spring/ Village, N13°14.86' W61°12.77'/ 15-27.VIII.06, clearing malaise trap,/ 348 m, S. & J. Peck, 06-101A” (CMNC). 1 male: “WEST INDIES: St. Vincent/ Hermitage Forest, E of Spring/ Village, N13°14.86' W61°12.77'/ 16-27.VIII.06, forest edge malaise,/ 340 m, S. & J. Peck, 06-104A” (CMNC). 1 male: “WEST INDIES: GRENADA/ Par. St. Andrews/ Mirabeau, Malaise trap/ 6.V.1990/ A. Thomas” (CMNC). 1 male: “ WEST INDIES: GRENADA/ Par. St. Andrews/ Mirabeau, malaise trap/ 2-6.III.1990/ R.E. Woodruff” (CMNC).

###### Type locality.

Montserrat, Big River, 16°45.719'N, 62°11.335'W.

###### Diagnosis.

This species is most like *S. nonseriatus*, from which it can be readily separated by a combination of: elytral striae same width from base to apex and pronotum bicolored, with paler lateral margin.

###### Descriptive notes.

Data for SBL in Table 1. Habitus as in Fig. 35B. Antennae and mouthparts rufo-testaceous to slightly darker. Legs with femora and tibiae testaceous to slightly darker, tarsus darker than femora and tibiae. Dorsal surface rufo-piceous to piceous, lateral bead of pronotum paler. Ventral surface rufo-brunneous to rufo-piceous, elytral epipleuron paler. Elytra moderately iridescent, ventral surface with less iridescence. Head, pronotum and elytra shiny, without microlines visible at 100×. Pronotum with posteriolateral impressions impunctate; without basal bead; posteriolateral angles obtuse, nearly rectangular. Elytral intervals distinctly convex, not flat. Elytral striae with interruptions, appearing punctate, in addition to the standard setigerous punctures in striae 2, 5 and 7. Males with two terminal setae and females with four terminal setae near the posterior margin on sternum VII.

**Male genitalia.** Fig. 36A–C. Very similar to those of *S. nonseriatus*, apical portion of phallic median lobe symmetrically rounded in dorsal/ventral aspect; endophallus with two dark, dense microtrichial fields nearly the length of the phallic median lobe, left dorsal markedly long, medial ventral slightly shorter; without lamina.

**Ovipositor and female reproductive tract.** Fig. 37B. Gonocoxite 2 somewhat falcate, moderately wide base. Bursa copulatrix markedly long, wide; markedly long spermatheca (**sp**) originating near base of common oviduct; melanized spermathecal basal sclerite (**sbs**) present, nearly half as long as spermatheca; moderately long spermathecal gland duct originating near mid-length of spermatheca apicad spermathecal basal sclerite. Spermathecal gland (**spg**) bulbous, with slight swelling of duct basad gland.

###### Geographical distribution.

Fig. 38. This species is known only from the Lesser Antillean islands of Montserrat, Martinique, St. Lucia, St. Vincent and Grenada.

###### Chorological affinities and relationships.

The range of this species overlaps the ranges of the other two species in the *nonseriatus* species group. Relationships of *S. iviei* are not postulated beyond species group membership.

###### Material examined.

Type material only; for details see above.

##### Selenophorus nonseriatus

Taxon classificationAnimaliaColeopteraCarabidae

Darlington

Figs 35C , 36D–F , 37C , 38

Selenophorus nonseriatus Darlington, 1934: 109. HOLOTYPE male: San Francisco Mts., Santo Domingo, Sept. 14, A. Busck (USNM). 2 female PARATYPES, same as holotype. One male PARATYPE: Claremont, Jamaica, March 14 (AMNH).— Erwin and Sims 1984: 440.— Ball 1992: 85.— Lorenz 1998: 355.— Lorenz 2005: 377.— Peck 2005: 32.— Peck 2006: 176.— Perez-Gelabert 2008: 80.

###### Type locality.

San Francisco Mountains, Elias Pinas Province, Dominican Republic, Hispaniola.

###### Diagnosis.

This species is most like *S. iviei*, from which it can be readily separated by a combination of: elytral striae wider preapically than on elytral disc and pronotum unicolorous, without paler lateral margins.

###### Descriptive notes.

Data for SBL in Table 1. Habitus as in Fig. 35C. Antennae and mouthparts rufo-testaceous to slightly darker; legs testaceous to rufo-testaceous. Dorsal surface rufo-brunneous to brunneo-piceous. Ventral surface rufo-brunneous to brunneo-piceous, elytral epipleuron paler. Elytra moderately iridescent, ventral surface with less iridescence. Head, pronotum and elytra shiny, without microlines visible at 100×. Pronotum with posteriolateral impressions impunctate; without basal bead; posteriolateral angles obtuse. Elytral intervals slightly convex on disc. Elytral striae impunctate, except the standard setigerous punctures in striae 2, 5 and 7. Males with two terminal setae and females with four terminal setae near the posterior margin on sternum VII.

**Male genitalia.** Fig. 36D–F. Very similar to those of *S. iviei*, apical portion of phallic median lobe short, narrowly rounded, symmetrically rounded in ventral/dorsal aspects; endophallus with two darkened microtrichial fields, nearly the length of the median lobe, left dorsal markedly long, medial ventral slightly shorter; without lamina.

**Ovipositor and female reproductive tract.** Fig. 37C. Gonocoxite 2 somewhat falcate, moderately wide base. Bursa copulatrix moderately long, recurved; markedly long spermatheca (**sp**) originating near base of common oviduct; melanized spermathecal basal sclerite (**sbs**) present, about one fifth as long as spermatheca; long spermathecal gland duct originating above mid-length of spermatheca apicad of spermathecal basal sclerite. Spermathecal gland (**spg**) small, bulbous, with slight swelling of duct basad gland.

###### Geographical distribution.

Fig. 38. This species is known from the Greater Antillean islands of Cuba, Hispaniola and Jamaica and the Lesser Antillean islands of Dominica, St. Vincent and Grenada.

###### Chorological affinities and relationships.

The range of this species overlaps the ranges of the other two species in the *nonseriatus* species group. Relationships of *S. nonseriatus* are not postulated beyond species group membership.

###### Material examined.

In addition to type material, we have seen a total of 180 specimens (99 males, 76 females, 5 unknown). See Appendix for details.

##### Selenophorus opalinus

Taxon classificationAnimaliaColeopteraCarabidae

species group

###### Recognition.

Larger species, elytral mesh pattern transverse, sculpticells distinctly wider than long, with microlines visible only in *S. flavilabris* and metepisterum elongate, lateral margin much longer than anterior margin.

**SBL.** Males, 6.08–9.60 mm; females, 6.32–9.52 mm.

**Color.** Antennae and mouthparts testaceous to rufo-testaceous. Legs testaceous to nearly piceous, tibiae unicolorous or gradually darkened apically. Dorsal and ventral surfaces rufo-brunneous to piceous.

**Luster.** Shiny, with faint to brilliant iridescence, or with metallic blue and green reflections.

**Dorsal microsculpture**. Head with mesh pattern isodiametric; pronotum with mesh pattern slightly transverse, sculpticells about 1.5–2× wide as long; elytra with mesh pattern transverse, sculpticells about 2–4× wide as long, or dorsal surface with no microlines visible at 100×.

###### Male genitalia.

Apical portion of phallic median lobe short to long, narrowly tapered to broadly rounded, apex with extreme apex curved ventrad, with short ventrad projection or unmodified. Endophallus without spines, with or without dark microtrichial fields, without lamina, ostium anopic to somewhat anopic-left pleuropic. Ventral surface of shaft smooth or with two ridges.

**Ovipositor and female reproductive tract.** Gonocoxite 2 moderately thick to thicker, moderately falcate. Bursa copulatrix moderately long; moderately long spermatheca, originating near base of common oviduct, with proximal swelling well above base or with basal swelling. Spermathecal gland duct moderately long to long, originating about mid-length of the distal swelling of spermatheca or originating just above basal swelling of spermatheca. Spermathecal gland bulbous or sausage-like, with swelling of duct basad gland.

###### Included species.

In the West Indies the *opalinus* species group includes seven taxa, one of which is represented by three subspecies: *S. fabricii*, **new species**, *S. flavilabris flavilabris* Dejean, *S. f. cubanus* Darlington, *S. f. ubancus* Ball and Shpeley, *S. integer* Fabricius, *S. opalinus* LeConte, and *S. propinquus* Putzeys.

###### Geographical distribution.

**T**he range of this species group in the West Indies is virtually co-extensive with the islands themselves.

##### Selenophorus fabricii sp. n.

Taxon classificationAnimaliaColeopteraCarabidae

http://zoobank.org/EFCD958C-8523-4164-8112-0799CDFE173E

**Figure 39.**
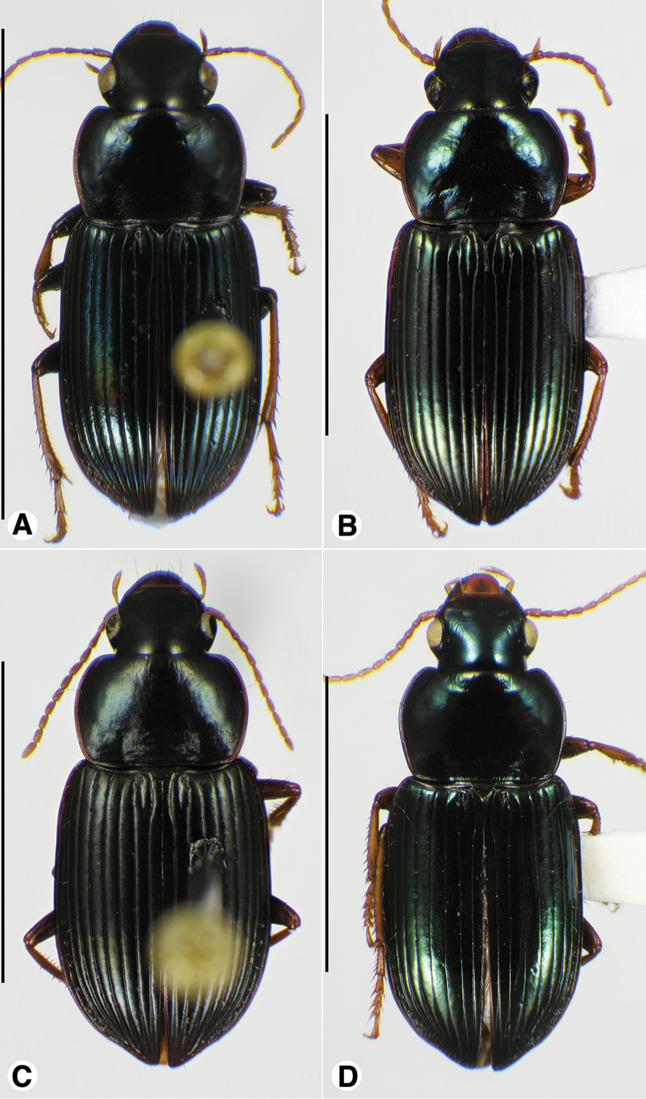
Habitus digital images of *Selenophorus opalinus* species group, in part, dorsal aspect. **A** *S. fabricii* sp. n. **B** *S. flavilabris cubanus* Darlington **C** *S. flavilabris flavilabris* Dejean **D** *S. flavilabris ubancus* Ball & Shpeley. Scale bars: **A** 10 mm; **B–D** 5 mm.

Figs 39A , 41A , 42A–C , 45

###### Specific epithet.

A Latinized eponym, genitive case, based on the surname of Johann Christian Fabricius, who described *Carabus integer*, the species with which this one has been confused.

###### Type material.

Total of 283 specimens collected on the Greater Antillean island of Hispaniola, 156 males and 127 females. HOLOTYPE male, labelled: “DOMINICAN REPUBLIC:/ Pedernales, Cabo Rojo/ 10 m.17-55N, 71-39W/ 26-27 September 1991”; “C. Young, S. Thompson,/ R.Davidson, J.Rawlins/ Coastal desert” (CMNH). PARATYPES 282, sex and label data as follows. 53 males, 35 females, labelled same as holotype (CMNH). 5 males, 5 females, labelled same as holotype (UASM). 1 male, 1 female, labelled same as holotype (CASC). 50 males, 45 females, labelled: “DOM.REP.:Prov.Pedernales/ Cabo Rojo, 0-10 m/ 10 SEP 1988, at light/ M. A. Ivie, TK. Philips/ & K. A. Johnson colrs.” (WIBF). 5 males, 5 females, labelled same as previous (UASM). 25 males, 25 females, labelled: “DOM.REP:/ Prov.La Romana/ La Romana IX.18.1976/ E.Folch blacklight trap/ in sugar cane field” (FSCA). 6 males, 1 female, labelled: “DOM.REP:Dajabon Prov/ Rio Massacre, 40m.,/ Balneario Don Miguel/ 7 km sw. Dajabon/ 26 May 1973/ Don & Mignon Davis (USNM). 4 males, 3 females, labelled: Rio Massacre, Balneario Don Miguel, 40 m, 7 km SW Dajabon, V.20.1973, D & M Davis (USNM). 3 males, labelled: “DOMINICANREP/ San Cristobal”; “8/9-VI-1969/ Flint &Gomez” (USNM). 1 male, 1 female labelled: “DOMINICANREP/ San Cristobal”; “8-9-VI-1969/ Flint&Gomez” (USNM). 2 females, labelled: “DOMINICANREP/ San Cristobal”; “8-9-VI-1969/ Flint &Gomez” (USNM). 2 females, labelled: “DOMINICAN REP./ Los Hidalgos”; “4-5 VI 1969/ Flint&Gomez” (USNM). 2 females, labelled: “DOMINICAN REP./ Los Hidalgos/ 4-5-June 1969/ Flint & Gomez” (USNM). 1 male, labelled: “DOMINICAN REP./ Jarabacoa/ 3-4 June 1969/ Flint&Gomez” (USNM). 1 male, labelled: “DOMINICAN REP./ Cachon de la Rubia/ nr.Central Ozama/ 10 June 1969 Flint & Gomez (USNM).

###### Type locality.

Cabo Rojo, Pedernales province, Dominican Republic.

###### Diagnosis.

This species is readily separated from the other members of the *opalinus* species group by the very wide striae in the preapical portion of the elytron, relative to the width of the striae on the elytral disc.

###### Descriptive notes.

Data for SBL in Table 1. Habitus as in Fig. 39A. Clypeus and labrum with anterior margin of each shallowly concave. Antennae and mouthparts testaceous to rufo-testaceous. Legs rufo-testaceous to dark brunneous, femur slightly darker than remainder of leg. Dorsal and ventral surfaces rufo-brunneous to nearly piceous. Elytra with moderate to brilliant iridescence, varying with angles to light source. Ventral surface with faint to moderate iridescence. Head, pronotum and elytra shiny, without microlines visible at 100×. Pronotum with posteriolateral angles rounded; posteriolateral impressions and laterally near the bead finely punctate, each puncture bearing a short, fine seta. Base of elytra, intervals 8 and 9 and apical portion of elytra with short, fine setae. Elytral striae impunctate, except the standard setigerous punctures in striae 2, 5 and 7. Elytral striae widened preapically, about as wide as adjacent interval, markedly wider than on elytral disc (Fig. 41A). Intervals with fine micro-punctures. Males with two terminal setae and females with four terminal setae near the posterior margin on sternum VII.

**Male genitalia.** Fig. 42A–C. Apical portion of phallic median lobe moderately long, narrowly tapered, symmetrically broadly rounded in dorsal/ventral aspect, extreme apex curved ventrad; endophallus with one darkened microtrichial field, about medial, in right lateral aspect; without lamina; ostium anopic. Ventral surface of distal 1/3 of shaft with two sharp ridges to apex.

**Ovipositor and female reproductive tract.** Very similar to those of *S. opalinus*, Fig. 44B. For details, see this topic for *S. opalinus*, below.

###### Geographical distribution.

Fig. 45. The known range of this species extends from Puerto Rico westward to Hispaniola, and then south-westward to Jamaica, the Caymans, the Swan Islands, and north-westward from Hispaniola to the Bahamas and the Key Islands off the coast of Florida.

###### Chorological affinities and relationships.

Within the *opalinus* species group, the range of this species is overlapped by the ranges of *S. flavilabris* (*sensu lato*), *S. integer*, *S. opalinus*, and *S. propinquus*. Relationships o*f S. fabricii* are not postulated beyond species group membership.

###### Material examined.

In addition to type material, we have seen a total of 1,633 specimens (843 males, 790 females). See Appendix for details.

**Figure 40.**
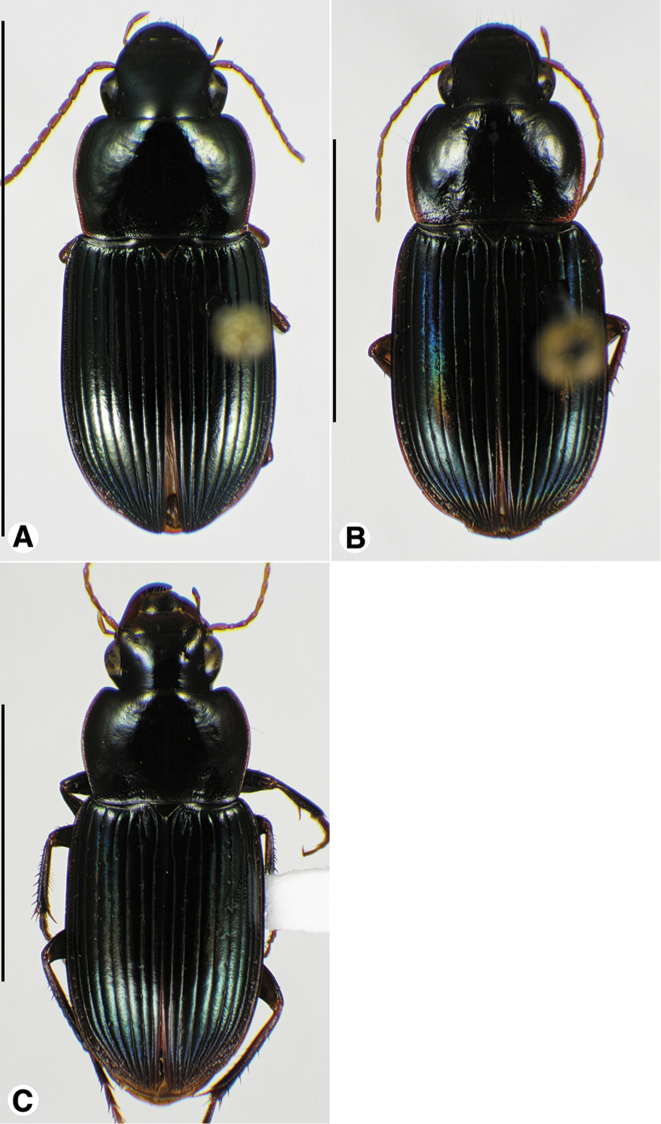
Habitus digital images of *Selenophorus opalinus* species group, in part, dorsal aspect. **A** *S. integer* (Fabricius) **B** *S. opalinus* LeConte **C** *S. propinquus* Putzeys. Scale bars: **A** 10 mm; **B, C** 5 mm.

**Figure 41.**
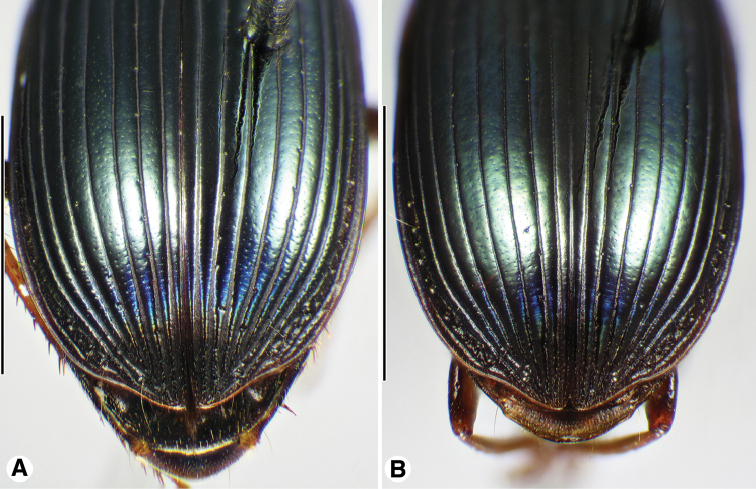
Digital images of apical portion of elytra of *Selenophorus* species, tilted dorsal aspect. **A** *S. fabricii* sp. n. **B** *S. integer* (Fabricius). Scale bars 3 mm.

**Figure 42.**
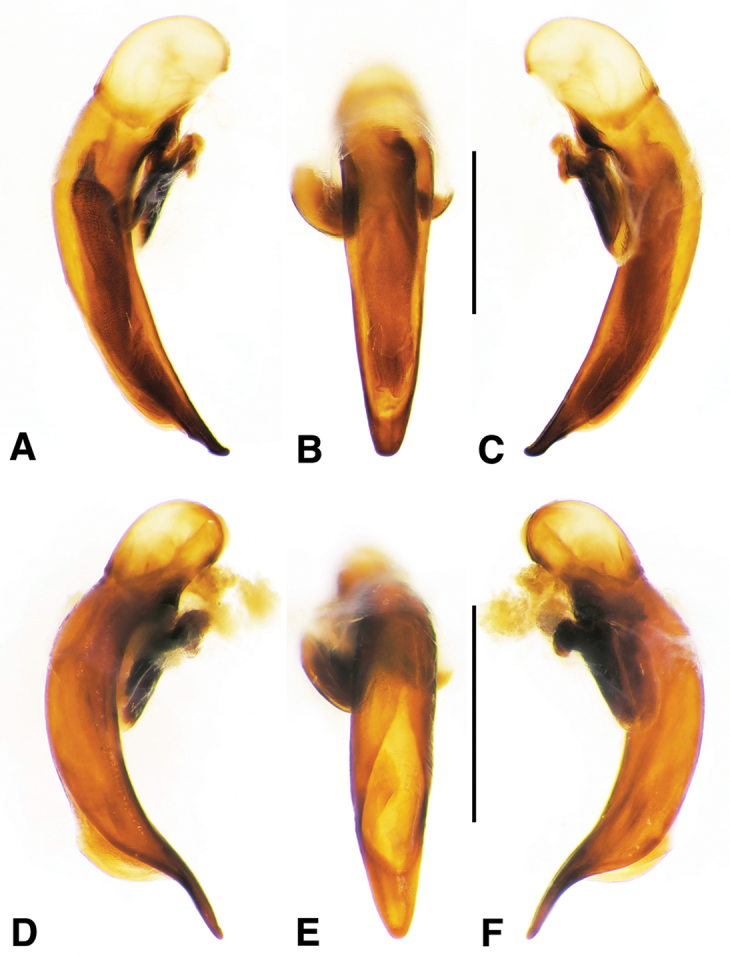
Digital images of male genitalia of *Selenophorus opalinus* species group, in part. **A, D** right lateral aspect **B, E** dorsal aspect **C, F** left lateral aspect. **A–C** *S fabricii* sp. n. **D–F** *S. flavilabris ubancus* Ball & Shpeley. Scale bars 1 mm.

**Figure 43.**
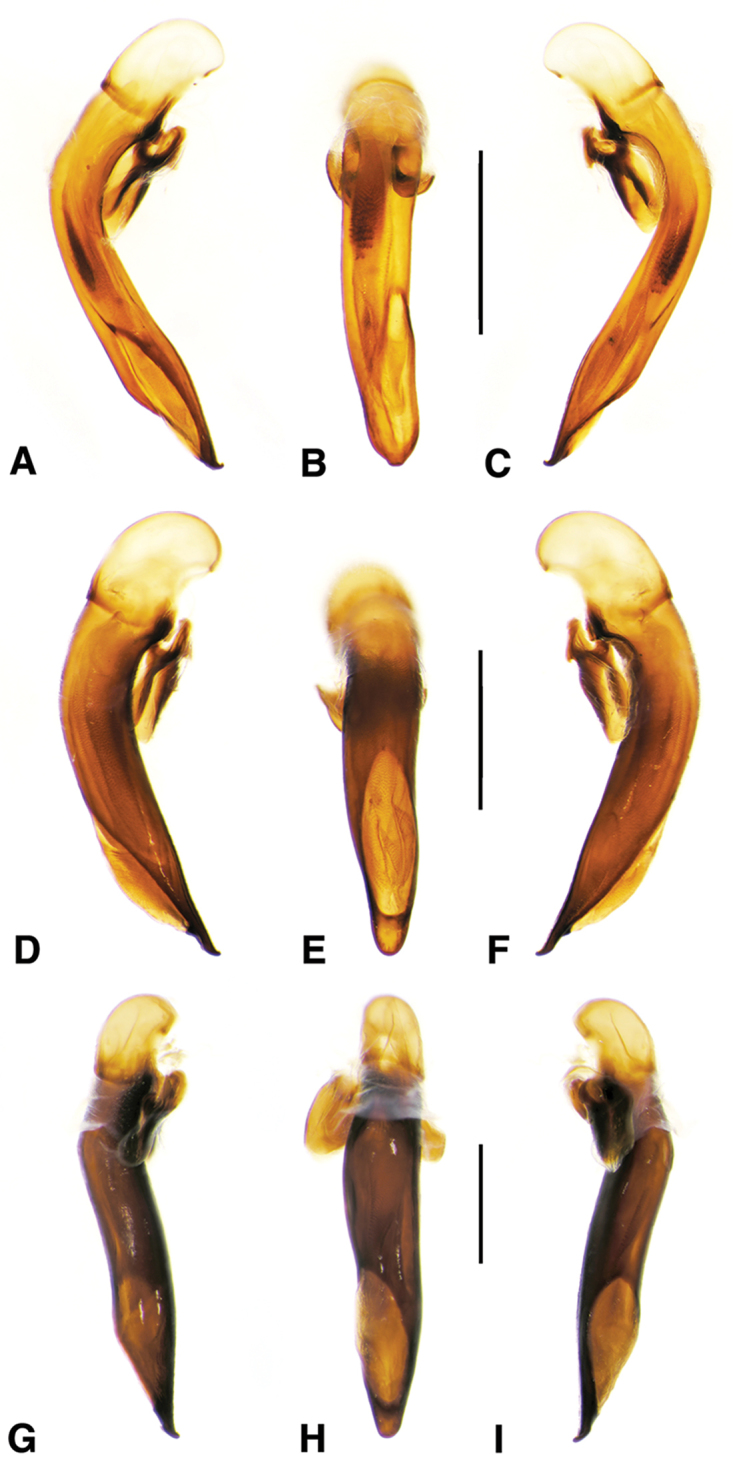
Digital images of male genitalia of *Selenophorus opalinus* species group, in part. **A, D, G** right lateral aspect **B, E, H** dorsal aspect **C, F, I** left lateral aspect. **A–C** *S. integer* (Fabricius) **D–F** *S. opalinus* LeConte **G–I** *S. propinquus* Putzeys. Scale bars 1 mm.

**Figure 44.**
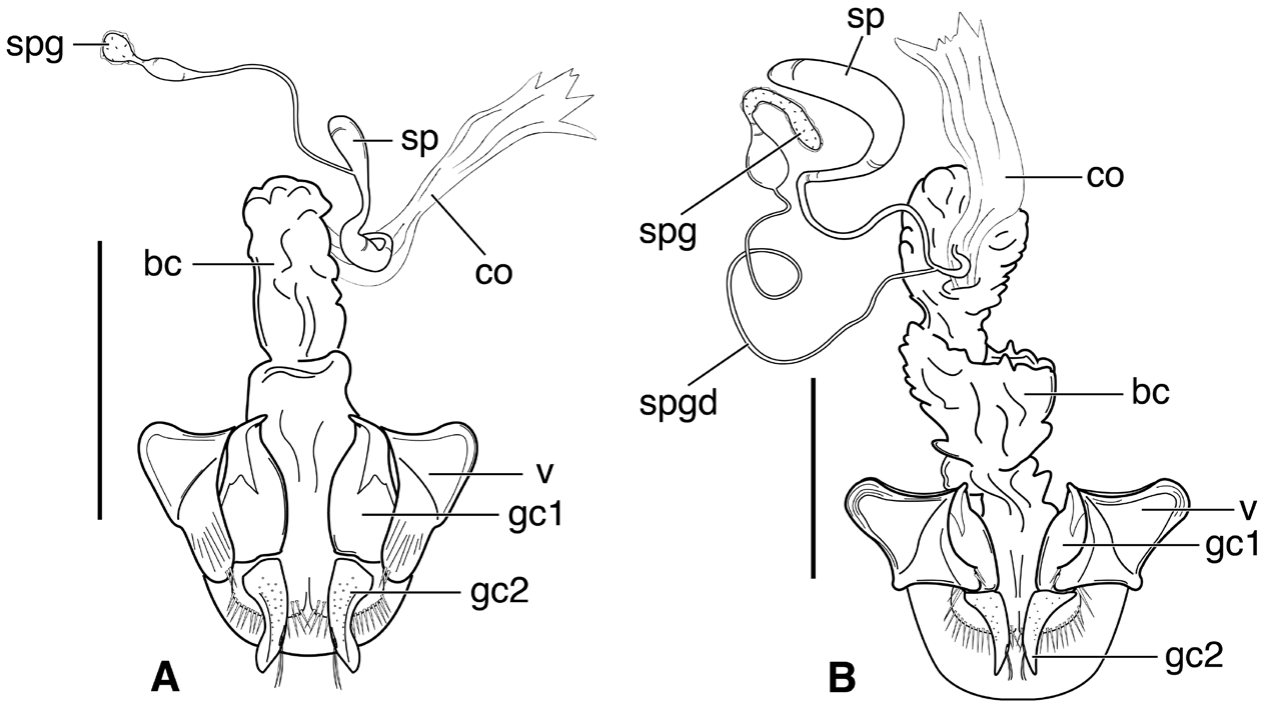
Line drawings of female reproductive tract of *Selenophorus opalinus* species group, in part, ventral aspect. **A** *S. flavilabris ubancus* Ball & Shpeley **B** *S. opalinus* LeConte. Legend: **bc** bursa copulatrix **co** common oviduct **gc1** gonocoxite 1 **gc2** gonocoxite 2 **sp** spermatheca **spg** spermathecal gland **spgd** spermathecal gland duct **v** valvifer. Scale bar 1 mm.

**Figure 45.**
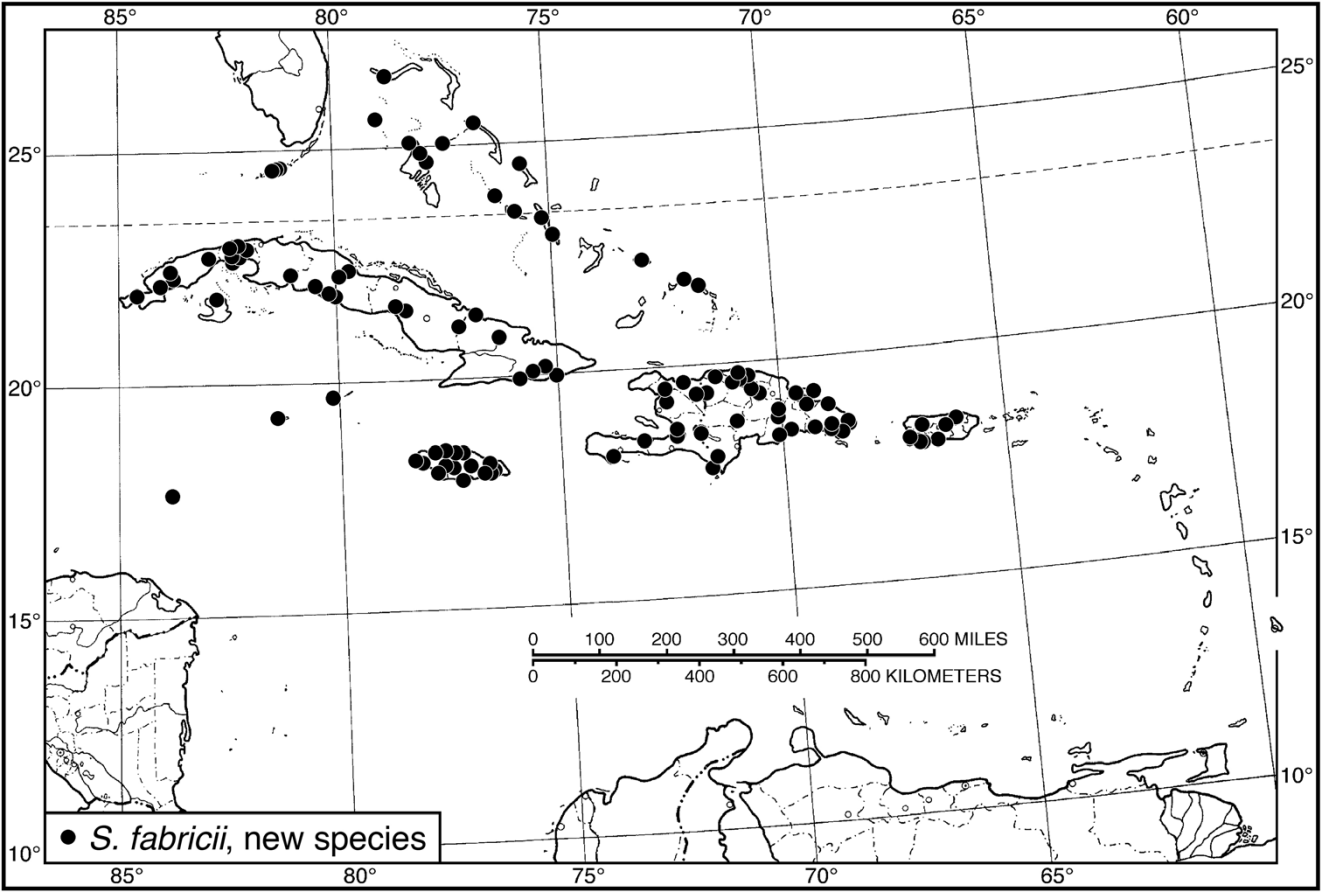
Map of West Indies showing known localities for species of *Selenophorus opalinus* species group, in part.

##### Selenophorus flavilabris

Taxon classificationAnimaliaColeopteraCarabidae

Dejean

###### Remarks.

This polytypic species is most conveniently treated by way of its subspecies. These are arranged below in alphabetical sequence by subspecific name.

##### Selenophorus flavilabris cubanus

Taxon classificationAnimaliaColeopteraCarabidae

Darlington

Figs 39B , 46

Selenophorus flavilabris cubanus Darlington, 1935b: 203. HOLOTYPE male and 35 PARATYPES: Soledad, near Cienfuegos, Cuba (various dates and collectors) (MCZC).— Blackwelder 1944: 49.— Erwin and Sims 1984: 440.— Ball 1992: 84, 85.— Ball and Shpeley 1992: 96.— Peck 2005: 32.
Selenophorus cubanus ; Ball 1992: 84, 85.— Ball and Shpeley 1992: 96.— Lorenz 1998: 355.— Lorenz 2005: 376.— Turnbow and Thomas 2008: 14.

###### Type locality.

Soledad, near Cienfuegos, Cienfuegos Province, Cuba.

###### Diagnosis.

This subspecies is readily separated from other species of the *opalinus* species group on a combination of: small size, entire dorsal surface with faint to moderate metallic reflection and legs unicolorous.

###### Descriptive notes.

Data for SBL in Table 1. Habitus as in Fig. 39B. Clypeus and labrum with anterior margin of each shallowly concave. Antennae, mouthparts and legs testaceous to rufo-testaceous. Dorsal and ventral surfaces rufo-brunneous to dark brunneous, not quite rufo-piceous. Dorsally with metallic blue and green reflections, not as bright as in *S. f. ubancus*, elytra additionally with faint iridescence; ventrally with very faint iridescence. Head, pronotum and elytra shiny, without microlines visible at 100×. Pronotum with posteriolateral angles rounded; posteriolateral impressions impunctate. Elytral striae impunctate, except the standard setigerous punctures in striae 2, 5 and 7. Intervals with fine micro-punctures. Males with two terminal setae and females with four terminal setae near the posterior margin on sternum VII.

**Male genitalia.** Very similar to *S. flavilabris ubancus*, Figs 42D–F. For details, see this topic for *S. flavilabris ubancus*, below.

**Ovipositor and female reproductive tract.** Very similar to *S. flavilabris ubancus*, Fig. 44A. For details, see this topic for *S. flavilabris ubancus*, below.

###### Geographical distribution.

**Figure 46.**
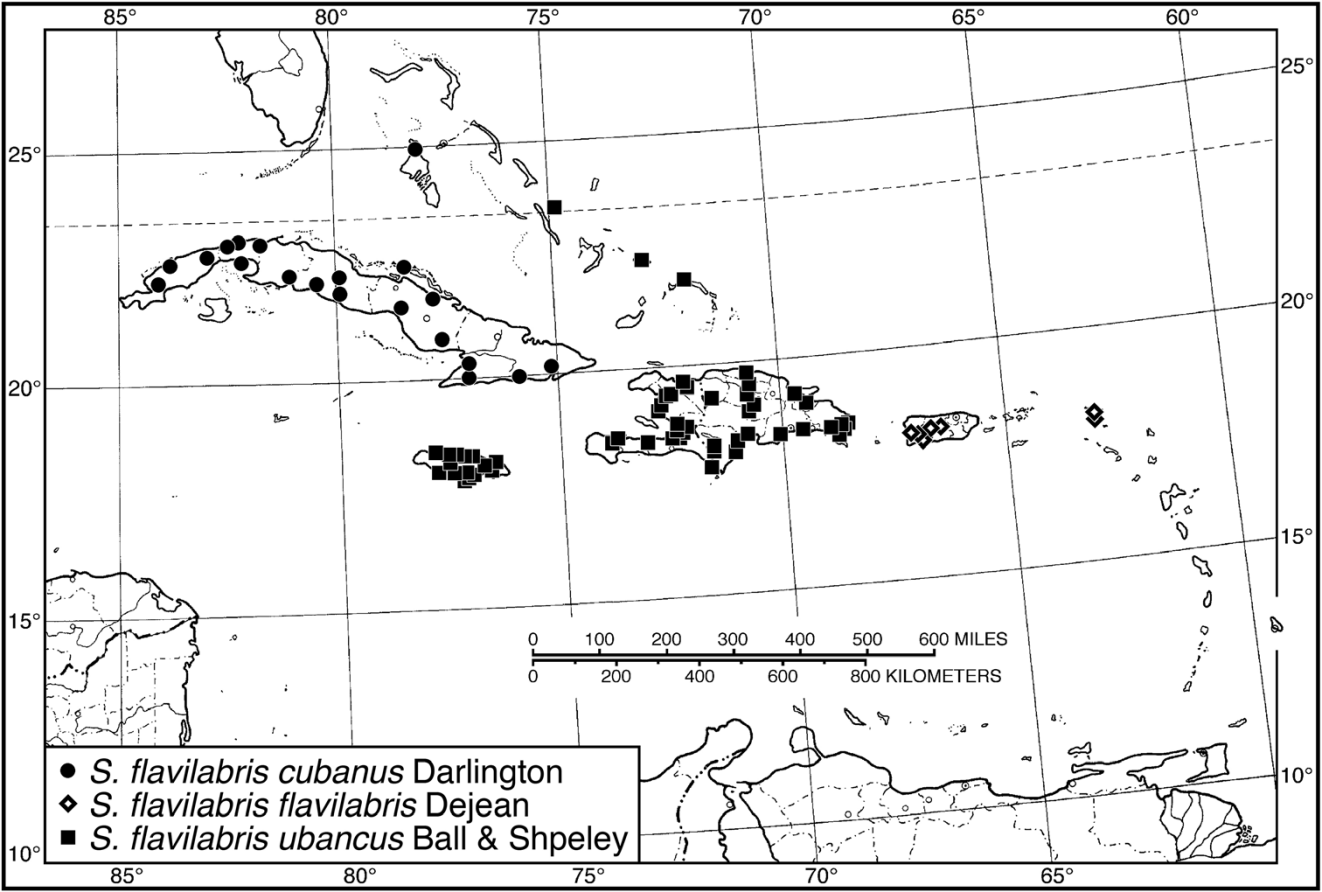
Map of West Indies showing known localities for species of *Selenophorus opalinus* species group, in part.

Fig. 46. This subspecies is known only from Greater Antillean Cuba and Andros Island in the Bahamas.

###### Chorological affinities and relationships.

The three subspecies of *S. flavilabris* are allopatric in distribution. The range of this subspecies is overlapped in the *opalinus* species group by the range of *S. fabricii*. Additionally, both this subspecies and *S. propinquus* are recorded from Andros Island in the Bahamas. Relationships of *S. flavilabris cubanus* are not postulated beyond species group membership.

###### Material examined.

In addition to type material, we have seen a total of 71 specimens (41 males, 30 females). See Appendix for details.

##### Selenophorus flavilabris flavilabris

Taxon classificationAnimaliaColeopteraCarabidae

Dejean

Figs 39C , 46

Selenophorus flavilabris Dejean, 1829: 79. Syntypes 3, in Chaudoir-Oberthür Collection; in front of following box label: //flavilabris/ Dej./ I. St. Barthelemy/C. Dejean// LECTOTYPE: specimen 1, male, labelled //[male]// //flavilabris/ m. in Ins. Barthelemy // //Schönherr//.— Gemminger and Harold 1868: 266.— Putzeys 1878a: 44.— Csiki 1932: 1198.— Blackwelder 1944: 49.— Erwin and Sims 1984: 440.— Ball 1992: 84, 85.— Ball and Shpeley 1992: 96.— Lorenz 1998: 355.— Lorenz 2005: 377.— Peck 2005: 32.— Turnbow and Thomas 2008: 14.
Selenophorus ramosi Darlington, 1939: 97. HOLOTYPE male, and 10 PARATYPES: Laguna Guánica, May 31, 1938 (MCZC).— Ball and Shpeley 1992: 96.

###### Type locality.

Saint Barthélemy, Leeward Islands, Lesser Antilles.

###### Diagnosis.

This subspecies is readily separated from other subspecies and species of the *opalinus* species group by the visible microlines on the dorsal surface.

###### Descriptive notes.

Data for SBL in Table 1. Habitus as in Fig. 39C. Clypeus and labrum with anterior margin of each shallowly concave. Antennae and mouthparts testaceous to rufo-testaceous. Legs bicolored, tibiae and tarsi testaceous to rufo-testaceous, femora rufo-brunneous to rufo-piceous. Dorsal and ventral surfaces rufo-brunneous to dark brunneous, not quite rufo-piceous, faintly iridescent. Head with mesh pattern isodiametric; pronotum with mesh pattern slightly transverse, sculpticells about 1.5–2× wide as long; elytra with mesh pattern transverse, sculpticells about 2–4× wide as long. Pronotum with posteriolateral angles rounded; posteriolateral impressions impunctate. Elytral striae impunctate, except the standard setigerous punctures in striae 2, 5 and 7. Intervals without fine micro-punctures. Males with two terminal setae and females with four terminal setae near the posterior margin on sternum VII.

**Male genitalia.** Very similar to *S. flavilabris ubancus*, Fig. 42D–F. For details, see this topic for *S. flavilabris ubancus*, below.

**Ovipositor and female reproductive tract.** Very similar to *S. flavilabris ubancus*, Fig. 44A. For details, see this topic for *S. flavilabris ubancus*, below.

###### Geographical distribution.

Fig. 46. This subspecies is known only from Puerto Rico and the two Lesser Antillean islands of Anguilla and St. Martin.

###### Chorological affinities and relationships.

The three subspecies of *S. flavilabris* are allopatric in distribution. Within the *opalinus* species group, the range of this subspecies is overlapped by the ranges of *S. fabricii*, *S. integer* and *S. propinquus*. Relationships of *S. flavilabris flavilabris* are not postulated beyond species group membership.

###### Material examined.

In addition to type material, we have seen a total of 74 specimens (25 males, 49 females). See Appendix for details.

##### Selenophorus flavilabris ubancus

Taxon classificationAnimaliaColeopteraCarabidae

Ball & Shpeley stat. n.

Figs 39D , 42D–F , 44A , 46

Selenophorus cubanus ubancus Ball & Shpeley, 1992: 103.— Ball 1992: 84, 85.— Lorenz 1998: 355.— Lorenz 2005: 376.— Perez-Gelabert 2008: 79.

###### Type material.

Complete label data for type material (holotype (MCZC), allotype and 231 paratypes) are provided in the original description.

###### Type locality.

Kenskoff, near Port-au-Prince, Ouest Department, Haiti, Hispaniola.

###### Diagnosis.

This subspecies is readily separated from other taxa of the *opalinus* species group on a combination of: small size, entire dorsal surface with bright metallic reflection and legs bicolored, femora darker than tibiae and tarsi.

###### Descriptive notes.

Data for SBL in Table 1. Habitus as in Fig. 39D. Clypeus and labrum with anterior margin of each shallowly concave. Antennae and mouthparts testaceous to rufo-testaceous. Legs bicolored, tibiae and tarsi testaceous to rufo-testaceous, femora infuscated, paler basally, remainder darker, rufo-brunneous to rufo-piceous. Dorsal and ventral surfaces rufo-brunneous to rufo-piceous, nearly piceous. Dorsally with metallic blue and green reflections, brighter than in *S. f. cubanus*, elytra additionally with iridescence; ventrally with iridescence. Head, pronotum and elytra shiny, without microlines visible at 100×. Pronotum with posteriolateral angles rounded; posteriolateral impression impunctate. Elytral striae impunctate, except the standard setigerous punctures in striae 2, 5 and 7. Intervals with fine micro-punctures. Males with two terminal setae and females with four terminal setae near the posterior margin on sternum VII.

**Male genitalia.** Fig. 42D–F. Apical portion of phallic median lobe long, narrowly triangular, symmetrically rounded in dorsal/ventral aspect, several minute subapical hooks on ventral surface; endophallus without darkened spine fields; without lamina; ostium anopic. Ventral surface of shaft smooth.

**Ovipositor and female reproductive tract.** Fig. 44A. Gonocoxite 2 (**gc2**) thick, moderately falcate. Bursa copulatrix (**bc**) moderately long; moderately long spermatheca (**sp**), with proximal swelling well above base, originating near base of common oviduct (**co**); long spermathecal gland duct originating about mid-length of the distal swelling of spermatheca. Spermathecal gland (**spg**) bulbous, with swelling of duct basad gland.

###### Geographical distribution.

Fig. 46. The range of this subspecies extends westward in the Greater Antilles from Hispaniola to Jamaica, and north-westward to North Caicos in the Turks and Caicos, and to Mayaguana Island and Rum Cay in the Bahamas.

###### Chorological affinities and relationships.

The three subspecies of *S. flavilabris* are allopatric in distribution. The range of this subspecies is overlapped by the ranges of *S. fabricii* and *S. propinquus*. Additionally, both this subspecies and *S. integer* are recorded from the eastern tip of Hispaniola. Relationships of *S. f. ubancus* are not postulated beyond species group membership.

###### Material examined.

In addition to type material, we have seen a total of 569 specimens (305 males, 264 females). See Appendix for details.

##### Selenophorus integer

Taxon classificationAnimaliaColeopteraCarabidae

Fabricius

**Figure 47.**
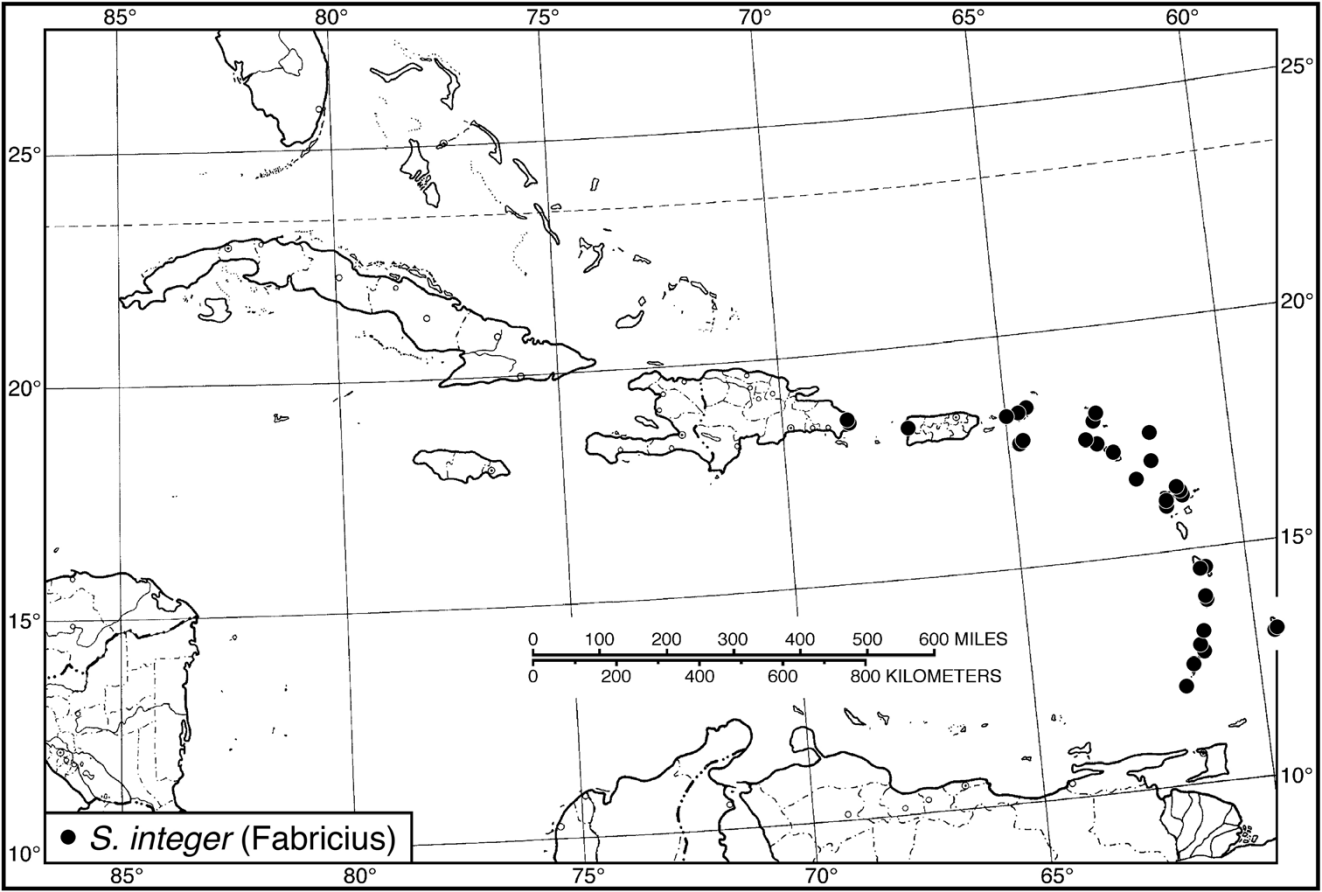
Map of West Indies showing known localities for species of *Selenophorus opalinus* species group, in part.

Figs 40A , 41B , 43A–C , 47

Carabus integer Fabricius, 1801: 196. TYPE MATERIAL: One syntype in ZMUC (Zimsen 1964: 57; Bousquet 2012: 1143).
Carabus grimmi Sturm, 1826: 148.
Selenophorus chalybeus Dejean, 1829: 110. 13 specimens in the Chaudoir-Oberthür Collection, in front of the following box label: //chalybeus/ Dej./ Petites Antilles/ C. Dejean//. LECTOTYPE (here selected), labelled: //[male// chalybeus Schönherr/ in Ins St. Barthelemy D [green paper; handwritten]// // Schönherr//.— Gemminger and Harold 1868: 265.— Putzeys 1878a: 47.— Csiki 1923: 1197.— Blackwelder 1944: 49.— Erwin and Sims 1984: 440.— Ball 1992: 85.— Lorenz 1998: 355.— Lorenz 2005: 376.— Peck 2005: 32.— Peck 2006: 176.— Ivie et al. 2008: 238.— Peck 2011: 13. **Syn. n.**
Harpalus integer ; Hope 1838: 41.— Gemminger and Harold 1868: 279.— Erwin and Sims 1984: 440.
Harpalus grimmi ; Gemminger and Harold 1868: 279 (junior synonym of Harpalus integer Fabricius).
Selenophorus integer ; Putzeys 1878a: 47.— Darlington 1934: 104.— Ball 1992: 85.— Lorenz 1998: 355.— Peck and Thomas 1998: 22.— Lorenz 2005: 377.— Peck 2005: 32.— Perez-Gelabert 2008: 79.— Turnbow and Thomas 2008: 14.— Bousquet 2012: 1143.

###### Type area.

*Americae insulis* (the Antilles). Here restricted to the Lesser Antillean island of St. Barthélemy, the type area for *S. chalybeus* Dejean, a junior synonym of *S. integer* Fabricius.

###### Notes.

Bennett and Alam (1985: 20) and Peck (2009: 12) included *Selenophorus affinis* in their list of the Barbados beetle fauna. However, Peck (2009: 12) also noted that this probably was a misidentification. *Selenophorus affinis*, a member of the subgenus Hemisopalus, is known to occur in Panama, Colombia and French Guiana. We believe that the correct species name is *Selenophorus integer*, as it is the only member of the *opalinus* species group currently known from the Barbados.

###### Diagnosis.

This species is readily separated from the other sympatric species in the *opalinus* species group by dorsal microsculpture, elytral stria width and leg color. Specimens of *Selenophorus f. flavilabris* have visible microlines on the dorsal surface; specimens of *S. fabricii* have the elytral striae much wider preapically relative to on the disc; and specimens of *S. propinquus* have the tibiae darkened apically. Specimens of *S. integer* have no visible microlines on the dorsal surface, elytral striae the same width preapically as on the disc and the tibiae are unicolorous, not darkened apically.

###### Descriptive notes.

Data for SBL in Table 1. Habitus as in Fig. 40A. Clypeus and labrum with anterior margin of each shallowly concave. Antennae and mouthparts testaceous to rufo-testaceous. Legs rufo-testaceous to rufous, femur slightly darker than remainder of leg. Dorsal and ventral surfaces rufo-brunneous to rufo-piceous. Elytra with faint to moderate iridescence, varying with angles to light source. Ventral surface with very faint iridescence. Head, pronotum and elytra shiny, without microlines visible at 100×. Pronotum with posteriolateral angles rounded; posteriolateral impressions and laterally near the bead finely punctate, each puncture bearing a short, fine seta. Base of elytra, intervals 8 and 9 and apical portion of elytra with short, fine pubescence. Elytral striae impunctate, except the standard setigerous punctures in striae 2, 5 and 7. Elytral striae very narrow from base to apex, not widened preapically (Fig. 41B). Intervals with fine micro-punctures. Males with two terminal setae and females with four terminal setae near the posterior margin on sternum VII.

**Male genitalia.** Fig. 43A–C. Apical portion of phallic median lobe short, broad, symmetrical, with short medial projection curved ventrad; endophallus with one darkened microtrichial field, about medial, in dorsal aspect; without lamina; ostium anopic-right pleuropic. Ventral surface of distal 1/3 of shaft with two sharp ridges to apex.

**Ovipositor and female reproductive tract.** Very similar to that of *S. opalinus*, Fig. 44B, but enlarged portion of spermatheca longer, and narrow portion shorter than in *S. opalinus*. For details, see this topic for *S. opalinus*, below.

###### Geographical distribution.

Fig. 47. The known range of this species extends from Greater Antillean eastern Hispaniola eastward to the Virgin Islands, and then southward through the Lesser Antilles as far south as Grenada.

###### Chorological affinities and relationships.

The range of this species is overlapped by the ranges of the following members of the *opalinus* species group: *S. fabricii*, *S. f. flavilabris*, *S. f. ubancus* and *S. propinquus* Relationships of *S. integer* are not postulated beyond species group membership.

###### Material examined.

In addition to type material, we have seen a total of 1,625 specimens (632 males, 992 females, 1 unknown). See Appendix for details.

##### Selenophorus opalinus

Taxon classificationAnimaliaColeopteraCarabidae

LeConte

**Figure 48.**
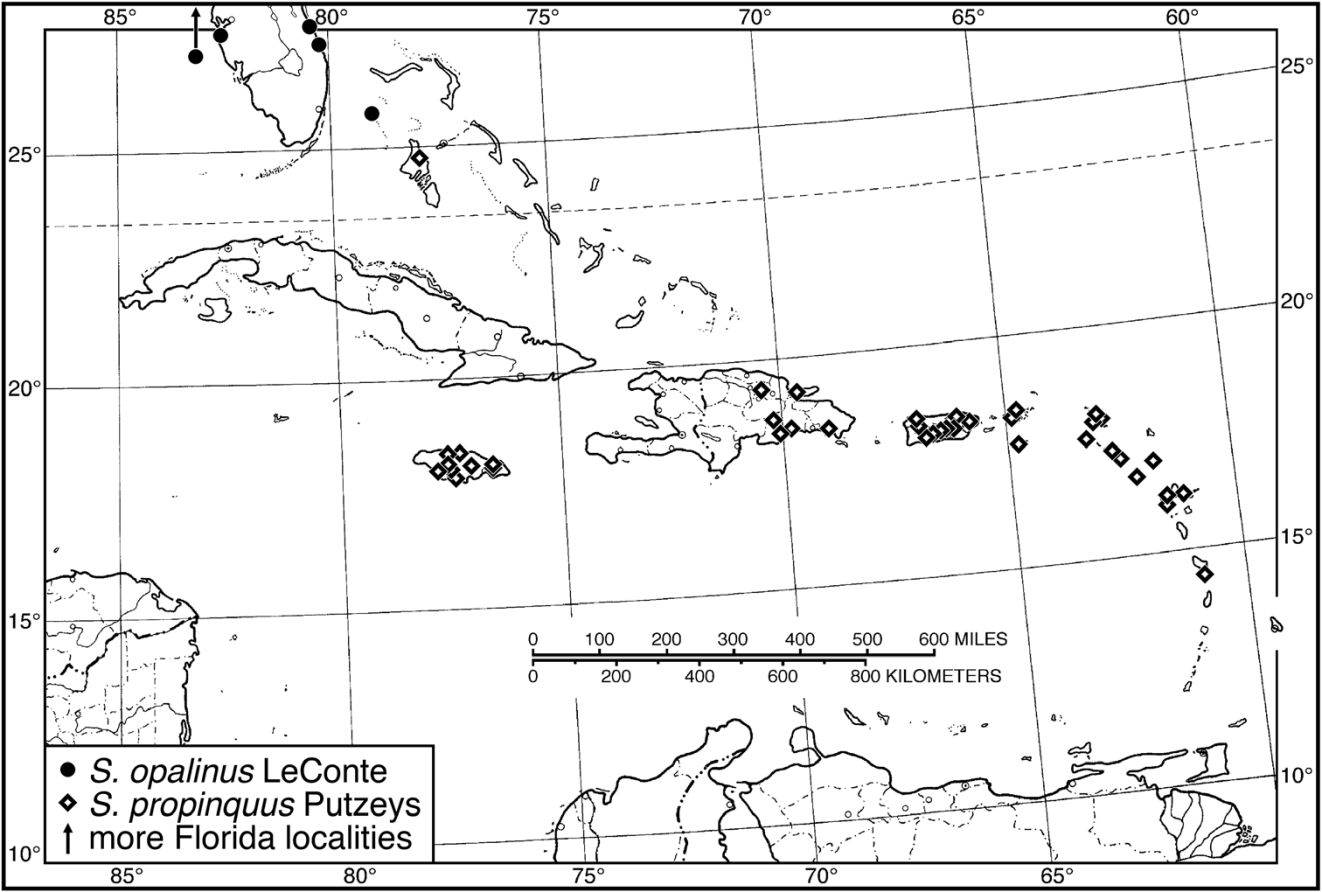
Map of West Indies showing known localities for species of *Selenophorus opalinus* species group, in part.

Figs 40B , 43D–F , 44B , 48

Selenophorus iripennis LeConte, 1848: 389 [not Say]. Secondary homonym of Selenophorus iripennis Say, 1823 = Amblygnathus iripennis (Say); see Ball and Maddison 1987: 206. TYPE MATERIAL: 8 syntypes in LeConte Collection (MCZC). LECTOTYPE, labelled: // orange disc]// //242// //Type/ 5922 [red paper]// //H. (S.) opalinus/ Lec/ iripennis Lec [handwritten]//.
Harpalus opalinus LeConte, 1863: 13. Replacement name for Harpalus iripennis (LeConte, 1848).
Selenophorus opalinus ; TYPE MATERIAL: see above.— Gemminger and Harold 1868: 266.— Putzeys 1878a: 62.— Csiki 1932: 1199.— Lindroth 1968: 824.— Ball 1992: 84, 85.— Peck and Thomas 1998: 22.— Lorenz 1998: 356.— Lorenz 2005: 377.— Bousquet 2012: 1144.

###### Type area.

Original citation “Carolina” and New York. Restricted to “Carolina” by Lindroth (1968: 824).

###### Diagnosis.

This species is readily separated from the only two members of the *opalinus* species group with which it may be sympatric. Specimens of *S. fabricii* have the elytral striae widened preapically, and specimens of *S. propinquus* have the tibiae darkened preapically. Specimens of *S. opalinus* have the striae the same width from the base of the elytron to the apex and the tibiae are unicolorous, not darkened apically.

###### Descriptive notes.

Data for SBL in Table 1. Habitus as in Fig. 40B. Clypeus and labrum with anterior margin of each shallowly concave. Antennae, mouthparts and legs testaceous to rufo-testaceous. Dorsal and ventral surfaces rufo-brunneous to piceous. Elytra with moderate to brilliant iridescence, varying with angles to light source. Ventral surface with moderate iridescence. Head, pronotum and elytra shiny, without microlines visible at 100×. Pronotum with posteriolateral angles rounded; posteriolateral impressions and laterally near the bead finely punctate, each puncture bearing a short, fine seta. Base of elytra, intervals 8 and 9 and apical portion of elytra with short, fine pubescence. Elytral striae impunctate, except the standard setigerous punctures in striae 2, 5 and 7. Intervals with fine micro-punctures. Males with two terminal setae and females with four terminal setae near the posterior margin on sternum VII.

**Male genitalia.** Fig. 43D–F. Apical portion of phallic median lobe symmetrically broadly rounded in dorsal/ventral aspect, extreme apex curved ventrad; endophallus without spines or darkened microtrichial fields; without lamina; ostium anopic. Ventral surface of distal 1/3 of shaft with two sharp ridges to apex.

**Ovipositor and female reproductive tract.** Fig. 44B. Gonocoxite 2 moderately thick, moderately falcate. Bursa copulatrix moderately long; moderately long spermatheca (**sp**), with basal swelling, originating near base of common oviduct; moderately long spermathecal gland duct (**spgd**) originating just above basal swelling of spermatheca. Spermathecal gland (**spg**) long, sausage-like, bulbous swelling of duct basad gland.

###### Geographical distribution.

Fig. 48. This mainland species is recorded in the West Indies only from South Bimini Island of the Bahamas.

###### Chorological affinities and relationships.

The range of this species is overlapped only by the range of *S. fabricii* within the *opalinus* species group. Relationships of *S. opalinus* are not postulated beyond species group membership.

###### Material examined.

In addition to type material, we have seen a total of 23 specimens (8 males, 15 females). See Appendix for details.

##### Selenophorus propinquus

Taxon classificationAnimaliaColeopteraCarabidae

Putzeys

Figs 40C , 43G–I , 48

Selenophorus propinquus Putzeys, 1874: 118. Species description evidently based on a specimen (or specimens) collected on the Lesser Antillean island of Antigua. In the Chaudoir-Oberthür Collection, are 3 specimens in front of the following box label: // Guadeloupe/ C. Dejean//. The first specimen is a female, labelled //Guad/ [small silver square]//, selected as Lectotype by Ball 1984. Because of their labels it seems doubtful that any of these three specimens are types, though judging from their small size, they all seem to fit the description of S. propinquus auctorum.— Putzeys 1878a: 49.— Csiki 1932: 1200.— Darlington 1934: 114.— Blackwelder 1944: 50.— Erwin and Sims 1984: 440.— Ball 1992: 85.— Lorenz 1998: 356.— Lorenz 2005: 377.— Peck 2006: 176.— Ivie et al. 2008: 238.— Turnbow and Thomas 2008: 14.— Peck 2011: 13.

###### Type locality.

The Lesser Antillean island of Antigua.

###### Diagnosis.

This species is readily separated from other members of the *opalinus* species group by the color of the tibiae, which are darkened apically.

###### Descriptive notes.

Data for SBL in Table 1. Habitus as in Fig. 40C. Clypeus and labrum with anterior margin of each shallowly concave. Antennae and mouthparts testaceous to rufo-testaceous. Legs dark testaceous to nearly brunneous, tibiae gradually darkened apically, to nearly piceous. Dorsal and ventral surfaces rufo-brunneous to piceous. Elytra with moderate to brilliant iridescence, varying with angles to light source. Ventral surface with moderate iridescence. Head, pronotum and elytra shiny, without microlines visible at 100×. Pronotum with posteriolateral angles rounded; posteriolateral impressions and laterally near the bead finely punctate, each puncture bearing a short, fine seta. Base of elytra, intervals 8 and 9 and apical portion of elytra with short, fine pubescence. Elytral striae impunctate, except the standard setigerous punctures in striae 2, 5 and 7. Intervals with coarser micro-punctures. Males with two terminal setae and females with four terminal setae near the posterior margin on sternum VII.

**Male genitalia.** Fig. 43G–I. Apical portion of phallic median lobe moderately long, narrowly tapered, symmetrically broadly rounded in dorsal/ventral aspect, extreme apex curved ventrad; shaft sinuous in lateral aspects rather than evenly curved; endophallus without spines or darkened microtrichial fields; without lamina; ostium somewhat anopic-left pleuropic. Ventral surface of distal 1/3 of shaft with two sharp ridges to apex.

**Ovipositor and female reproductive tract.** Very similar to that of *S. opalinus*, Fig. 44B. For details, see this topic for *S. opalinus*, above.

###### Geographical distribution.

Fig. 48. This species is recorded from Andros Island in the Bahamas, Greater Antillean Jamaica, to the Virgin Islands and St. Croix, and from Anguilla, Antigua, southward through the Lesser Antilles to Martinique.

###### Chorological affinities and relationships.

The range of this species is overlapped by the ranges of the following members of the *opalinus* species group: *S. fabricii*, *S. flavilabris* (*sensu lato*) and *S. integer*. Relationships of *S. propinquus* are not postulated beyond species group membership.

###### Material examined.

In addition to type material, we have seen a total of 693 specimens (339 males, 354 females). See Appendix for details.

##### Selenophorus palliatus

Taxon classificationAnimaliaColeopteraCarabidae

species group

###### Recognition.

Combination of the following characters: head, pronotum and elytra with mesh pattern isodiametric; serial punctures of striae 2, 5 and 7 foveate; and hind tarsus about 2/3 length of hind tibia.

**SBL.** Males, 6.12–8.60 mm; females, 6.28–9.12 mm.

**Color.** Antennae testaceous to rufo-testaceous, same color as legs or darker. Mouthparts and legs testaceous to rufo-testaceous. Dorsal and ventral surface rufo-brunneous to nearly piceous. Elytra distinctly bicolored or with apical margin diffusely paler or unicolorous. Elytral epipleuron pale, same color as the legs.

**Luster.** Dorsal surface with faint greenish to cupreous metallic luster

**Dorsal microsculpture**. Head, pronotum and elytra with mesh pattern isodiametric.

###### Male genitalia.

Apical portion of phallic median lobe short to moderately long, triangular, symmetrically rounded in dorsal/ventral aspect; endophallus with 4 microtrichial spine fields, spines thin and short or without spines or darkened microtrichial spine fields; without lamina.

**Ovipositor and female reproductive tract.** Gonocoxite 2 moderately long to long, thick, slightly falcate. Bursa copulatrix short to moderately long; large somewhat bulbous to sausage-like spermatheca originating near base of common oviduct; moderately long to long spermathecal gland duct originating near middle of bulb of spermatheca. Spermathecal gland small, bulbous, with or without small swelling of duct basad gland.

###### Included species.

The *palliatus* species group includes four species in the West Indies: *S. alternans* Dejean, *S. palliatus* (Fabricius), *S. pyritosus* Dejean and *S. woodruffi* Ball and Shpeley.

###### Geographical distribution.

The range of this species group in the West Indies extends throughout the Bahamas and Greater and Lesser Antilles.

##### Selenophorus alternans

Taxon classificationAnimaliaColeopteraCarabidae

Dejean

**Figure 49.**
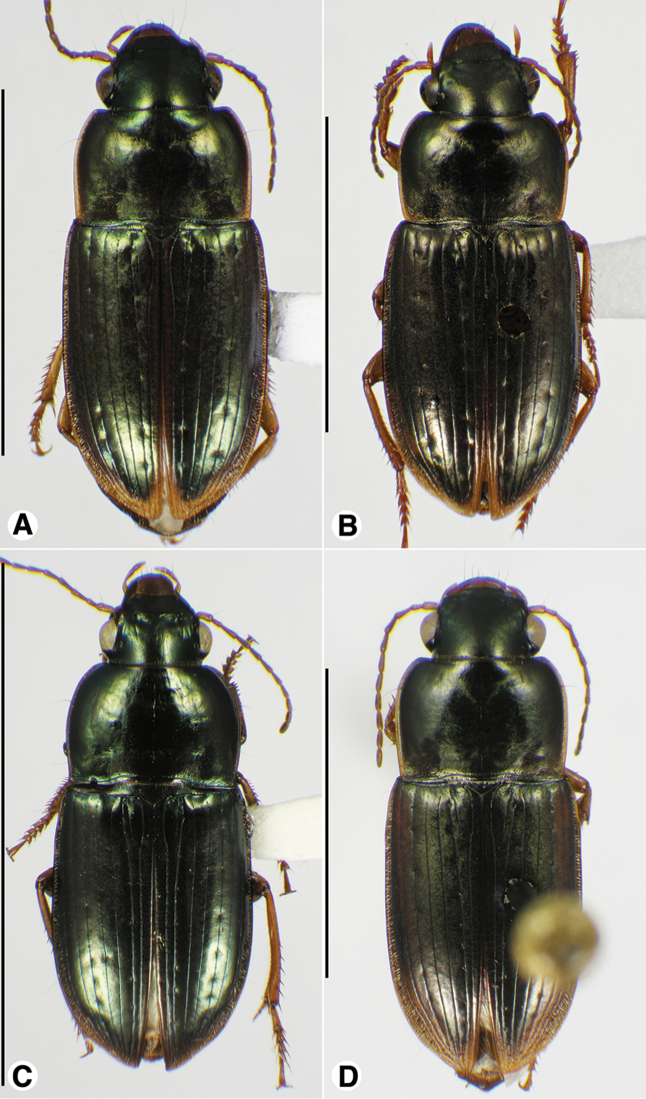
Habitus digital images of *Selenophorus palliatus* species group, dorsal aspect. **A** *S. alternans* Dejean **B** *S. palliatus* (Fabricius) **C** *S. pyritosus* Dejean **D** *S. woodruffi*, Ball & Shpeley. Scale bars: **A, B, D** 5 mm; **C** 10 mm.

**Figure 50.**
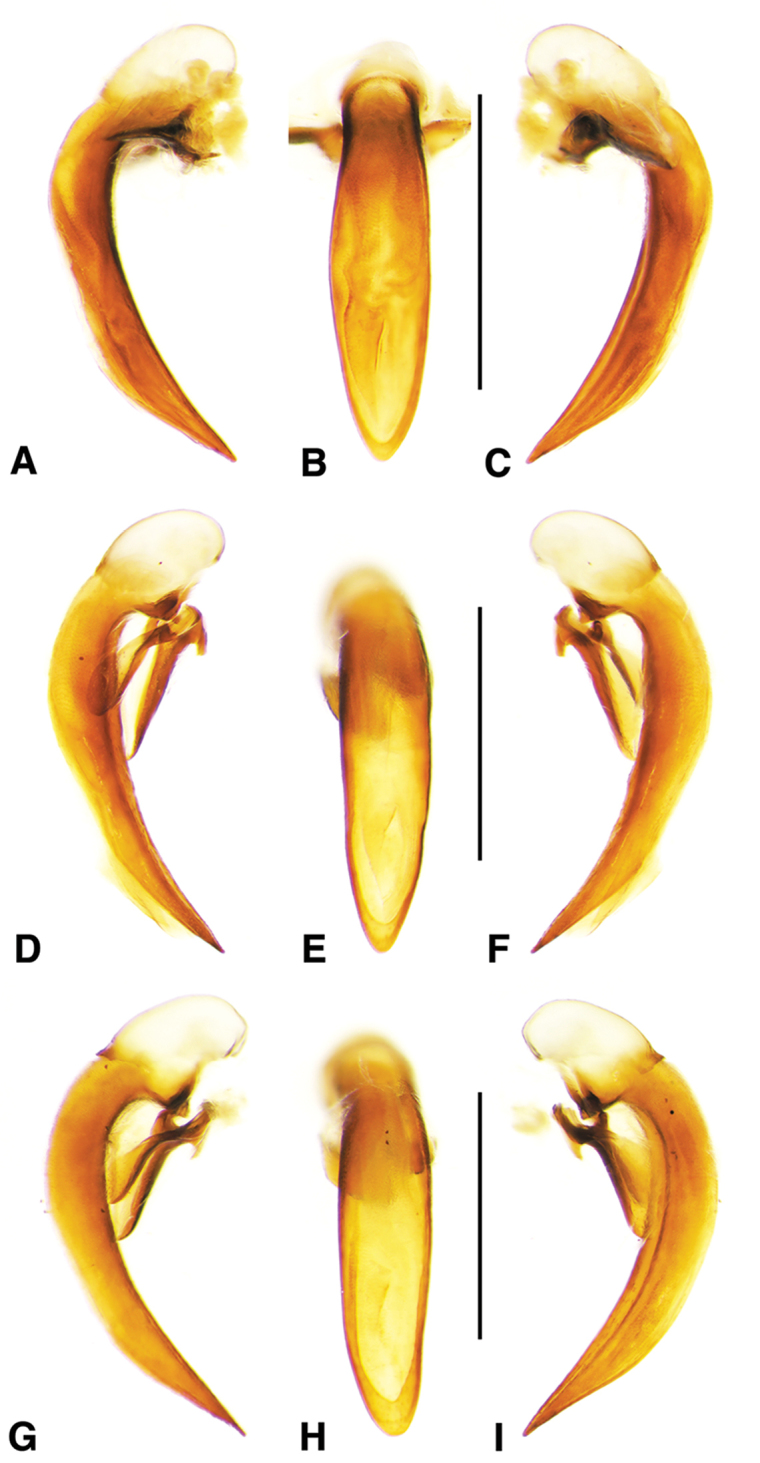
Digital images of male genitalia of *Selenophorus palliatus* species group, in part. **A, D, G** right lateral aspect **B, E, H** dorsal aspect **C, F, I** left lateral aspect. **A–C** *S. alternans* Dejean **D–F** *S. palliatus* (Fabricius) **G–I** *S. pyritosus* Dejean. Scale bars 1 mm.

Figs 49A , 50A–C , 53

Selenophorus alternans Dejean, 1829: 86. In Chaudoir-Oberthür Collection, 33 specimens in front of following box label: alternans/ Dej./ Bresil/ C. Dejean// LECTOTYPE (here selected), labelled: [male]// alternans m/ in Brasilia [green paper, handwritten]// [MNHP].— Gemminger and Harold 1868: 265.— Gundlach 1894: 294.— Csiki 1932: 1196.— Darlington 1934: 104.— Blackwelder 1944: 49.— Erwin and Sims 1984: 440.— Bennett and Alam 1985: 20.— Ball 1992: 85.— Lorenz 1998: 355; Lorenz 2005: 376.— Peck 2005: 32.— Ivie et al. 2008: 238.— Perez-Gelabert 2008: 79.— Turnbow and Thomas 2008: 14.— Peck 2009: 13.
Selenophorus lineatopunctatus Dejean, 1829: 86. TYPE MATERIAL: male, in front of the *alternans* box label (see above); LECTOTYPE (here selected), labelled: [male]// lineatopunctatus m./ Cayenne [green paper, handwritten]// [MNHP].— Gemminger and Harold 1868: 266.— Putzeys 1878a: 13.

###### Type locality.

Vicinity of Rio de Janeiro, State of Rio de Janeiro, Brazil.

###### Diagnosis.

This species is readily separated from the similarly colored member of the *palliatus* species group, *S. woodruffi*, by the impunctate intervals next to the basal ridge.

###### Descriptive notes.

Data for SBL in Table 1. Habitus as in Fig. 49A. Clypeus and labrum with anterior margin of each shallowly concave. Head, pronotum and elytra with mesh pattern isodiametric. Mouthparts and legs testaceous to slightly darker; antennae darker than legs. Dorsal and ventral surface rufo-brunneous to dark brunneous; dorsal surface with faint aeneous metallic luster. Elytron bicolored, with apical fascia testaceous to slightly darker, length of pale marking nearly the same in intervals 2–9, forming a diagonal pale fascia; pale marking of interval 1longer than that of interval 2; intervals 3–5 may be darker just in front of pale apical fascia. Elytral epipleuron pale, same color as the legs. Elytral striae impunctate, except the standard setigerous punctures in striae 2, 5 and 7. Punctures of striae 2, 5 and 7 markedly foveate. Elytron with intervals impunctate basally near basal ridge. Both males and females with four terminal setae near the posterior margin on sternum VII.

**Male genitalia.** Fig. 50A–C. Very similar to that of *S. pyritosus*. For details, see this topic for *S. pyritosus*, below.

**Ovipositor and female reproductive tract.** Very similar to that of *S. pyritosus*, Fig. 52A. For details, see this topic for *S. pyritosus*, below.

###### Geographical distribution.

Fig. 53. The known range of this species extends through the Lesser Antilles north-westward to the Virgin Islands, Puerto Rico, Hispaniola and to the islands of Andros, Mayaguana and New Providence in the Bahamas.

###### Chorological affinities and relationships.

The West Indian range of this species is overlapped by the ranges of *S. palliatus*, *S. pyritosus* and *S. woodruffi*, all members of the *palliatus* species group. Relationships of *S. alternans* are not postulated beyond species group membership.

###### Material examined.

In addition to type material, we have seen a total of 678 specimens (310 males, 365 females, 3 unknown). See Appendix for details.

**Figure 51.**
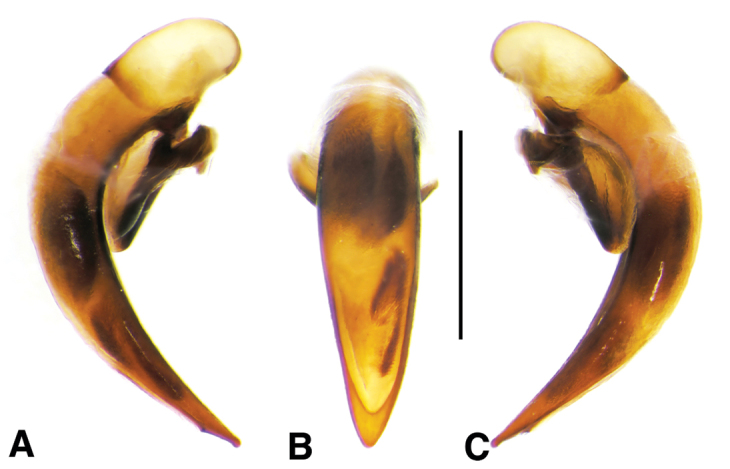
Digital images of male genitalia of *Selenophorus palliatus* species group, in part, *S. woodruffi* Ball & Shpeley. **A** right lateral aspect **B** dorsal aspect **C** left lateral aspect. Scale bar 1 mm.

**Figure 52.**
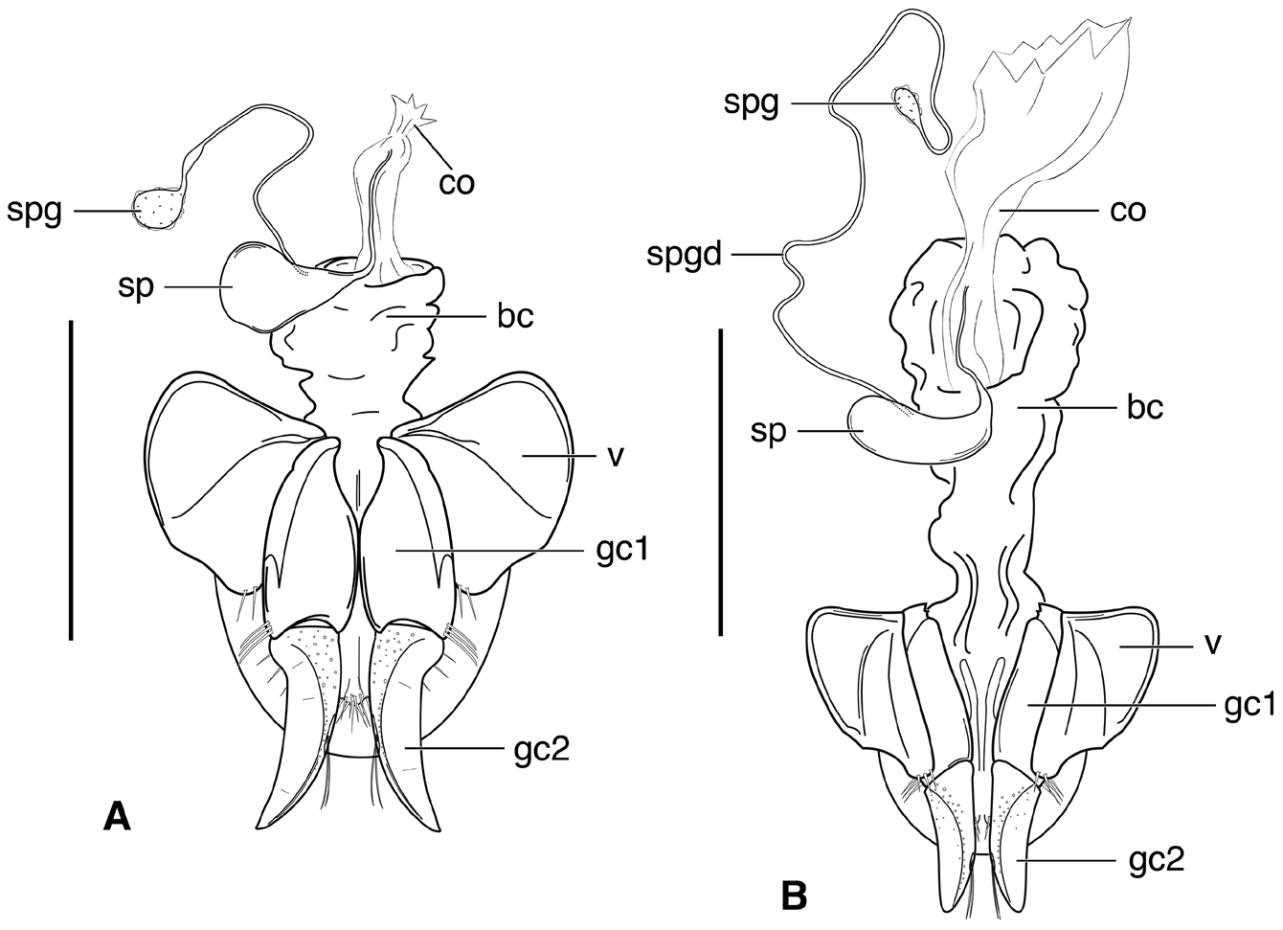
Line drawing of female reproductive tract of *Selenophorus palliatus* species group, in part, ventral aspect. **A** *S. pyritosus* Dejean **B** *S. woodruffi* Ball & Shpeley. Legend: **bc** bursa copulatrix; **co** common oviduct **gc1** gonocoxite 1 **gc2** gonocoxite 2 **sp** spermatheca **spg** spermathecal gland **spgd** spermathecal gland duct **v** valvifer. Scale bars 1 mm.

**Figure 53.**
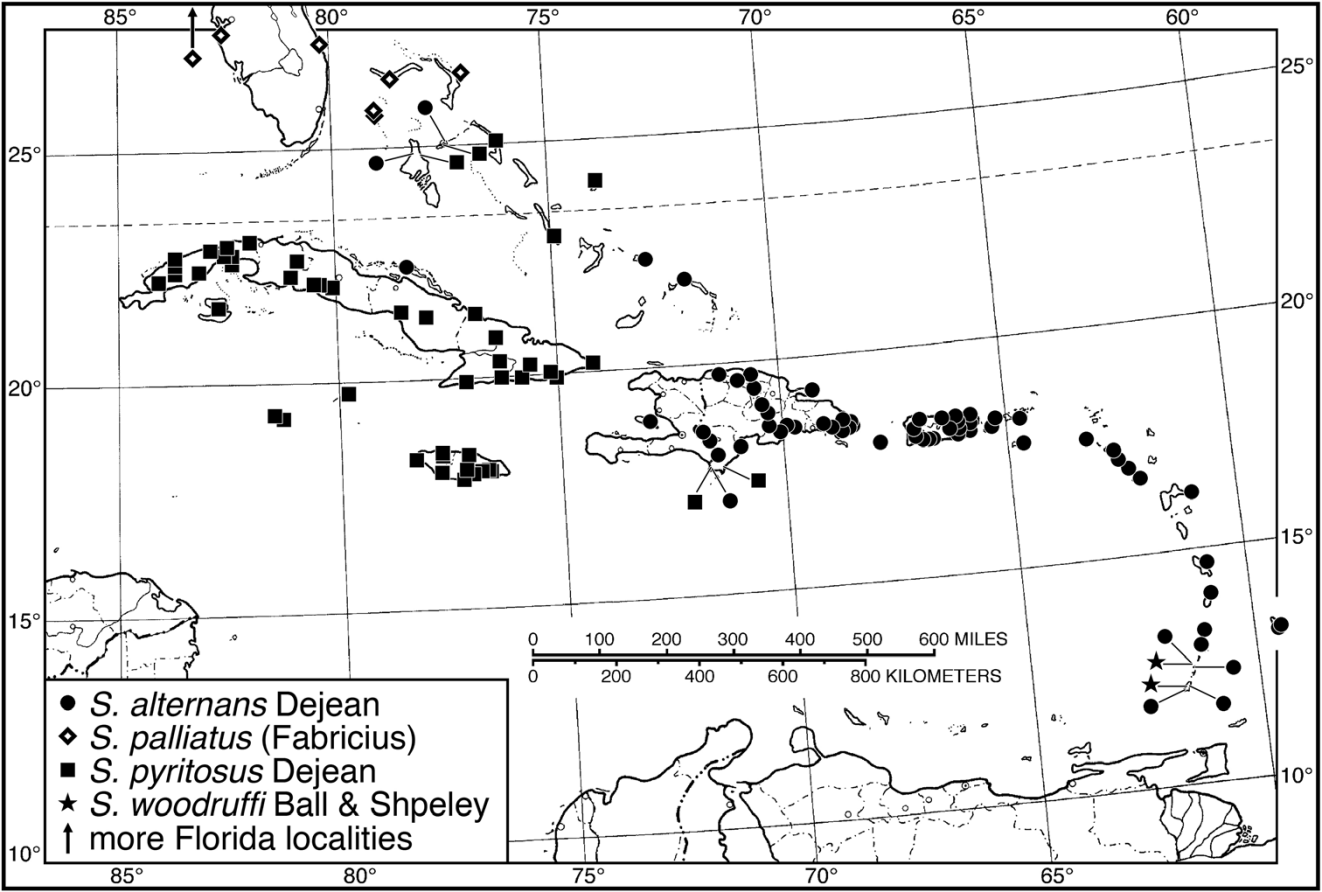
Map of West Indies showing known localities for species of *Selenophorus palliatus* species group.

##### Selenophorus palliatus

Taxon classificationAnimaliaColeopteraCarabidae

Fabricius

Figs 49B , 50D–F , 53

Carabus palliatus Fabricius, 1798: 58. TYPE MATERIAL: syntype [ZMUC]
Harpalus stigmosus Germar, 1824: 25. TYPE MATERIAL: syntypes probably lost (Bousquet 2012: 1144; synonymy established by Brullé 1835b: 290).—Putzeys 1878a: 12;
Selenophorus stigmosus ; Putzeys 1878a: 12.
Selenophorus impressus Dejean, 1829: 82. TYPE MATERIAL: one syntype in MNHP (Lindroth 1955: 28; Bousquet 2012: 1144);
Selenophorus palliatus ; Gemminger and Harold 1868: 265.— Csiki 1932: 1200.— Blackwelder 1944: 50.—Darlington 1953a: 9.— Ball 1992: 84, 85.— Peck and Thomas 1998: 22.— Lorenz 1998: 356.— Lorenz 2005: 377.— Turnbow and Thomas 2008: 14.— Bousquet 2012: 1144.

###### Type area.

“America boreali” (original citation). Here restricted to “Georgia”, the locality given for *H. stigmosus*, a junior synonym of *S. palliatus*.

###### Diagnosis.

This species is readily separated from the other members of the *palliatus* species group by the rounded posteriolateral angles of the pronotum, which are nearly rectangular in *S. alternans*, *S. pyritosus* and *S. woodruffi*.

###### Descriptive notes.

Data for SBL in Table 1. Habitus as in Fig. 49B. Clypeus and labrum with anterior margin of each shallowly concave. Head, pronotum and elytra with mesh pattern isodiametric. Mouthparts and legs testaceous to slightly darker; antennae darker than legs. Dorsal and ventral surface rufo-brunneous to brunneous; dorsal surface with faint cupeous metallic luster. Elytron bicolored, with apical fascia testaceous to slightly darker, length of pale marking nearly the same in intervals 2–9, forming a diagonal pale fascia; pale marking of interval 1 longer than that of interval 2. Elytral epipleuron pale, same color as the legs. Elytral striae impunctate, except the standard setigerous punctures in striae 2, 5 and 7. Punctures of striae 2, 5 and 7 foveate. Elytron with intervals impunctate basally near basal ridge. Males with two terminal setae and females with four terminal setae near the posterior margin on sternum VII.

**Male genitalia.** Fig. 50D–F. Very similar to those of *S. pyritosus*. For details, see this topic for *S. pyritosus*, below.

**Ovipositor and female reproductive tract.** Very similar to that of *S. pyritosus*, Fig. 52A. For details, see this topic for *S. pyritosus*, below.

###### Geographical distribution.

Fig. 53. This species is recorded only from Man-O-War Cay and North and South Bimini in the Bahamas in the West Indies.

###### Chorological affinities and relationships.

The West Indian range of this species is overlapped by the ranges of *S. alternans* and *S. pyritosus*, members of the *palliatus* species group. Relationships of *S. palliatus* are not postulated beyond species group membership.

###### Material examined.

In addition to type material, we have seen a total of 67 specimens (32 males, 35 females). See Appendix for details.

##### Selenophorus pyritosus

Taxon classificationAnimaliaColeopteraCarabidae

Dejean

Figs 49C , 50G–I , 52A , 53

Selenophorus pyritosus Dejean, 1829: 84. In the Chaudoir-Oberthür Collection, 27 specimens in front of the following box label: pyritosus/ Dej./ Antilles/ Col. Dejean// LECTOYPE: Specimen 1 labelled: //[male]// //pyritosus m./ in Ins. Cuba [handwritten, green paper// (here selected) [MNHP].— Gemminger and Harold 1868: 265.— Putzeys 1878a: 11.— Gundlach 1894: 293.— Csiki 1932: 1201.— Darlington 1934: 104.— Blackwelder 1944: 50.— Erwin and Sims 1984: 440.— Ball 1992: 84, 85.— Lorenz 1998: 356.— Lorenz 2005: 377.— Peck 2005: 32.— Perez-Gelabert 2008: 80.— Turnbow and Thomas 2008: 14.
Isopleurus macleayi Kirby, 1837: 50. TYPE MATERIAL: HOLOTYPE female, labelled: Type /HT [circular, ringed with red]// N. Amer/ [female]’’ Isopleurus Macleayi Kirby!/ I. multipunctatus Kirby Mss./ E. Indies 5751 Rev. Wm. Kirby [handwritten]// [BMNH].— LeConte 1873: 324. **Syn. n.**
Selenophorus alternans pyritosus Darlington, 1953a: 9.

###### Notes about synonymy.

Darlington 1953a: 9 proposed that *S. pyritosus* Dejean was a subspecies of *S. alternans* Dejean. However, we believe that *S. pyritosus* is a valid species.

###### Type area.

Cuba.

###### Diagnosis.

This species is readily separated from the other members of the *palliatus* species group by a combination of: posteriolateral angles of pronotum nearly rectangular and elytra without pale apical fascia, or with only narrow diffusely pale margin.

###### Descriptive notes.

Data for SBL in Table 1. Habitus as in Fig. 49C. Clypeus and labrum with anterior margin of each shallowly concave. Head, pronotum and elytra with mesh pattern isodiametric. Antennae, mouthparts and legs testaceous to rufo-testaceous. Dorsal and ventral surface rufo-brunneous to nearly piceous; dorsal surface with faint cupreous metallic luster. Elytra with apical margin diffusely paler or not. Elytral epipleuron pale, same color as the legs. Elytral striae impunctate, except the standard setigerous punctures in striae 2, 5 and 7. Punctures of striae 2, 5 and 7 foveate. Elytron with intervals impunctate basally near basal ridge. Males with two terminal setae and females with four terminal setae near the posterior margin on sternum VII.

**Male genitalia.** Fig. 50G–I. Apical portion of phallic median lobe short, triangular, symmetrically rounded in dorsal/ventral aspect; endophallus without spines or darkened microtrichial spine fields; without lamina.

**Ovipositor and female reproductive tract.** Fig. 52A. Gonocoxite 2 (**gc2**) long, thick, slightly falcate. Bursa copulatrix short (**bc**); large somewhat bulbous spermatheca (**sp**) originating from common oviduct (**co**), with proximal half attached to common oviduct; spermathecal gland duct originating near middle of bulb of spermatheca. Spermathecal gland (**spg**) small, bulbous, with small swelling of duct basad gland.

###### Geographical distribution.

Fig. 53. This species is known only from the Bahamas, Caymans and Greater Antillean islands of Cuba, Hispaniola and Jamaica.

###### Chorological affinities and relationships.

The West Indian range of this species is overlapped by the ranges of *S. alternans* and *S. palliatus*. Relationships of *S. pyritosus* are not postulated beyond species group membership.

###### Material examined.

In addition to type material, we have seen a total of 1,203 specimens (522 males, 681 females). See Appendix for details.

##### Selenophorus woodruffi

Taxon classificationAnimaliaColeopteraCarabidae

Ball & Shpeley

Figs 49D , 51A–C , 52B , 53

Selenophorus woodruffi Ball & Shpeley, 1992: 96.— Ball 1992: 85.— Lorenz 1998: 356.— Lorenz 2005: 378.

###### Type material.

Complete label data for type material (holotype (FSCA), allotype, and 63 paratypes) are provided in the original description.

###### Diagnosis.

This species is readily separated from similarly colored member of the *palliatus* species group, *S. alternans*, by the punctate elytral intervals next to the basal ridge. Additionally, some specimens have intervals 6–7 or 6–8 diffusely paler than the elytral disc but darker than the pale apical fascia.

###### Descriptive notes.

Data for SBL in Table 1. Habitus as in Fig. 49D. Clypeus and labrum with anterior margin of each shallowly concave. Head, pronotum and elytra with mesh pattern isodiametric Antennae, mouthparts and legs testaceous to slightly darker. Dorsal and ventral surface rufo-brunneous to nearly piceous; dorsal surface with faint aeneous/cupreous metallic luster. Elytron bicolored, with apical fascia testaceous to slightly darker, pale marking of 2^nd^–5^th^ intervals short, pale marking of 1^st^ and 6^th^–9^th^ intervals longer; intervals 6–7 or 6–8 may be diffusely paler than elytral disc but darker than apical fascia. Elytral epipleuron pale, same color as the legs. Elytral striae impunctate, except the standard setigerous punctures in striae 2, 5 and 7. Punctures of striae 2, 5 and 7 foveate. Elytron with intervals finely punctate basally near basal ridge. Males with two terminal setae and females with four terminal setae near the posterior margin on sternum VII.

**Male genitalia.** Fig. 51A–C. Apical portion of phallic median lobe moderately long, triangular, symmetrically rounded in dorsal/ventral aspect; endophallus with 4 microtrichial spine fields, spines thin and short; without lamina.

**Ovipositor and female reproductive tract.** Fig. 52B. Gonocoxite 2 moderately long, thick, slightly falcate. Bursa copulatrix moderately long; large sausage-like spermatheca (**sp**) originating near base of common oviduct, with proximal one third attached to common oviduct; long spermathecal gland duct (**spgd**) originating near middle of bulb of spermatheca; spermathecal gland (**spg**) small, bulbous.

###### Geographical distribution.

Fig. 53. This species is known only from the Lesser Antillean islands of Grenada and Mayreau in the West Indies.

###### Chorological affinities and relationships.

The range of this species is overlapped by the range of *S. alternans*. Relationships of *S. woodruffi* are not postulated beyond species group membership.

###### Material examined.

In addition to type material, we have seen a total of 70 specimens (49 males, 21 females). See Appendix for details.

##### Selenophorus parumpunctatus

Taxon classificationAnimaliaColeopteraCarabidae

species group

###### Recognition.

Externally, two species with elytron with pre apical notch on lateral margin. Internally, the endophallus of males with numerous short spines.

**SBL.** Males, 4.28–6.04 mm; females, 4.68–6.24 mm.

**Color.** Antennae with antennomeres 1–3 pale, antennomeres 4–11 darker. Mouthparts infuscated, testaceous to brunneous. Legs testaceous to dark rufo-testaceous. Dorsal surface brunneous to brunneo-piceous; ventral surface rufo-brunneous to brunneous. Elytral epipleuron paler than disc.

**Luster.** Dorsal surface dull to shiny, with or without very faint brassy luster, ventral surface dull.

**Dorsal microsculpture.** Head with mesh pattern isodiametric, microlines well impressed. Pronotum with slightly stretched transverse mesh pattern, sculpticells about 1.5–2× wide as long. Elytra with slightly to more stretched transverse mesh pattern, sculpticells 1.5–4× wide as long.

**Male genitalia.** Apical portion of phallic median lobe moderately long to long, narrowly tapered to triangular, symmetrical in dorsal/ventral aspect. Preapical orifice anopic; endophallus with 4–8 short spines with large bases; without lamina.

**Ovipositor and female reproductive tract.** Female of *S. obtusoides* is not known. Bursa copulatrix moderately long, recurved; long spermatheca originating near base of common oviduct, without distinctive narrowing basally; markedly long spermathecal gland duct originating above base of spermatheca. Spermathecal gland very small, bulbous, with moderately large swelling of duct basad gland.

###### Included species.

The *parumpunctatus* species group includes two species: *S. obtusoides* sp. n.and *S. parumpunctatus* Dejean.

###### Geographical distribution.

In the West Indies, the range of this species group is virtually co-extensive with the islands themselves.

##### Selenophorus obtusoides sp. n.

Taxon classificationAnimaliaColeopteraCarabidae

http://zoobank.org/8D37B226-134B-49B5-B828-8F99144272F4

**Figure 54.**
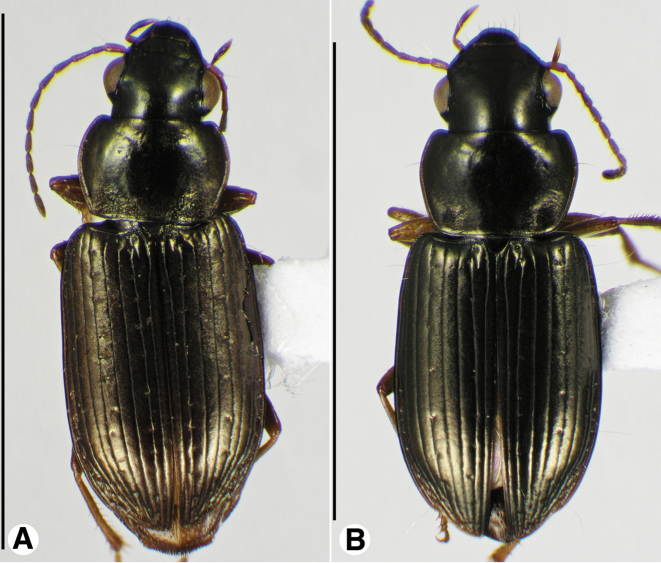
Habitus digital images of *Selenophorus parumpunctatus* species group, dorsal aspect. **A** *S. obtusoides* sp. n. **B** *S. parumpunctatus* Dejean. Scale bars 5 mm.

**Figure 55.**
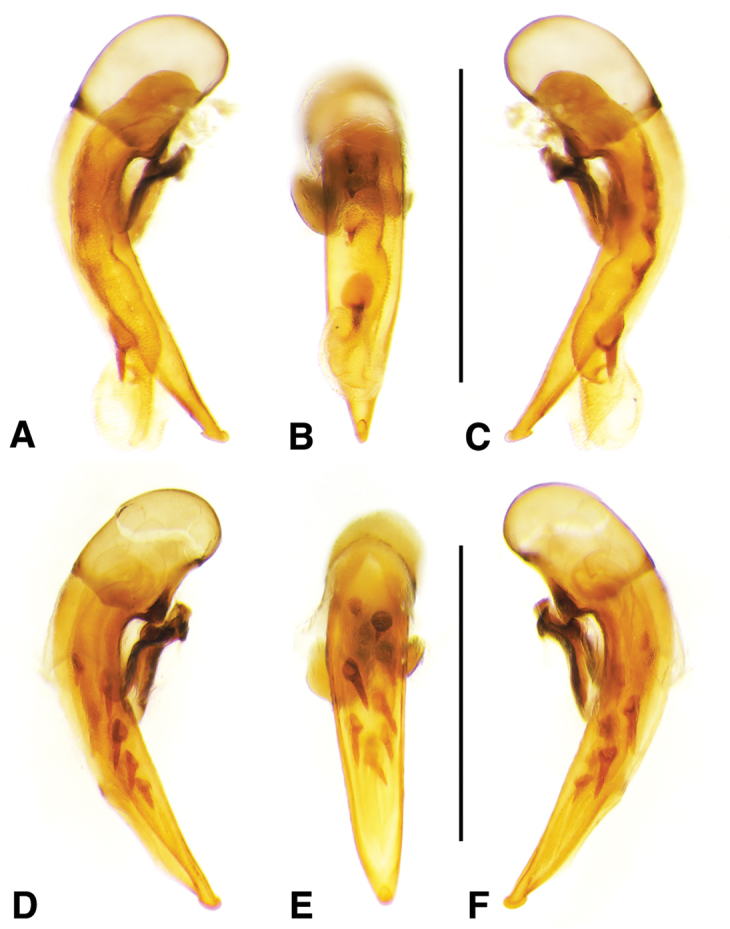
Digital images of male genitalia of *Selenophorus parumpunctatus* species group. **A, D** right lateral aspect **B, E** dorsal aspect **C, F** left lateral aspect. **A–C** *S. obtusoides* sp. n. **D–F** *S. parumpunctatus* Dejean. Scale bars 1 mm.

Figs 54A , 55A–C , 57

###### Specific epithet.

From Latin, “*obtusus*”, in reference to the obtuse posteriolateral angles of the pronotum, and Greek “*oides*”, having the form of.

###### Type material.

A single male, Holotype, labelled: “Lomas de Soroa/ 5.VI.1963/ Pinar del Rio. CUBA”; “CZ Acc/ 7.101501” (IZAC).

###### Type locality.

Near Soroa, Pinar del Rio province, Cuba.

###### Diagnosis.

Readily distinguished from *S. parumpunctatus* by a combination of: smaller size, the obtuse posteriolateral angles of the pronotum and setigerous punctures of striae 2, 5 and 7 more foveate.

###### Descriptive notes.

Data for SBL in Table 1. Habitus as in Fig. 54A. Clypeus and labrum with anterior margin of each very shallowly concave, nearly straight. Antennae with antennomeres1–3 testaceous, antennomeres 4–11 darker; palpi infuscated, rufous to rufo-brunneous, tips testaceous; legs rufo-testaceous. Dorsal surface brunneous; ventral surface rufo-brunneous; elytral epipleuron paler than disc. Head with mesh pattern isodiametric; pronotum with mesh pattern slightly transverse, sculpticells about 1.5× wide as long; elytra with mesh pattern slightly transverse, sculpticells about 1.5–2× wide as long. Pronotum with posteriolateral impressions punctate. Elytral striae impunctate, except the standard setigerous punctures in striae 2, 5 and 7; punctures of striae 2, 5 and 7 foveate. Male with two terminal setae near the posterior margin on sternum VII.

**Male Genitalia** Fig. 55A–C. Apical portion of phallic median lobe long, narrowly tapered, symmetrically rounded in dorsal/ventral aspect; endophallus with a row of six spines with large bases, medial in dorsal aspect; without lamina; ostium anopic.

**Ovipositor and female reproductive tract.** Female unknown.

###### Geographical distribution.

Fig. 57. This species is known only from the type locality of Lomas de Soroa in Pinar del Rio Province, Cuba.

###### Chorological affinities and relationships.

The range of this species is broadly overlapped by the range of *S. parumpunctatus*, the only other known member of the *parumpunctatus* species in the West Indies. Relationships of *S. obtusoides* are not postulated beyond species group membership.

###### Material examined.

Only the male holotype known; for details, see above.

**Figure 56.**
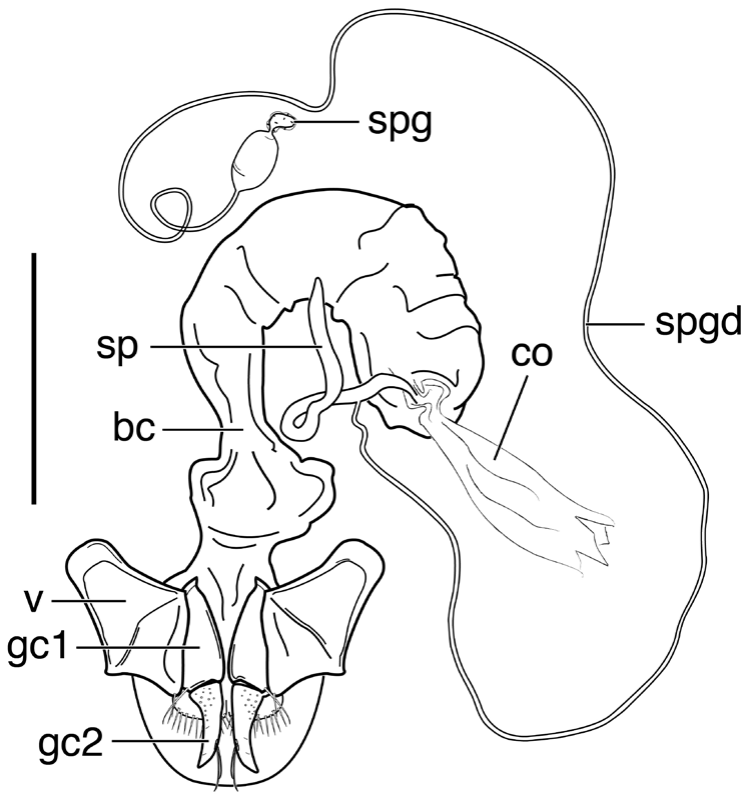
Line drawing of female reproductive tract of *Selenophorus parumpunctatus* species group, in part, ventral aspect, *S. parumpunctatus* Dejean. Legend: **bc** bursa copulatrix **co** common oviduct **gc1** gonocoxite 1 **gc2** gonocoxite 2 **sp** spermatheca **spg** spermathecal gland **spgd** spermathecal gland duct **v** valvifer. Scale bars 1 mm.

**Figure 57.**
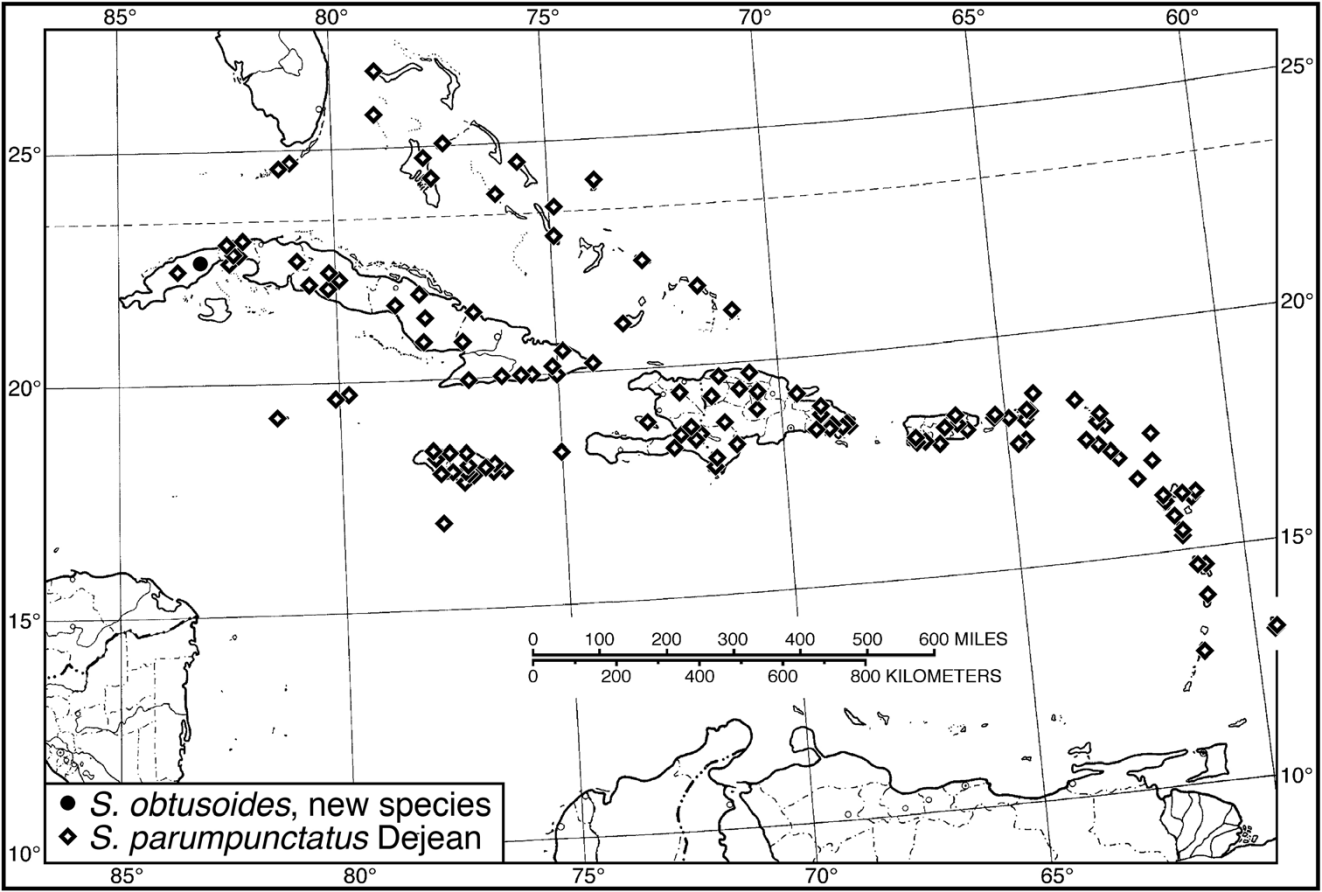
Map of West Indies showing known localities for species of *Selenophorus parumpunctatus* species group.

##### Selenophorus parumpunctatus

Taxon classificationAnimaliaColeopteraCarabidae

Dejean

Figs 54B , 55D–F , 56 , 57

Carabus sinuatus Gyllenhal [in Schönherr], 1806: 203 [primary junior homonym of Carabus sinuatus Gmelin, 1790].
Selenophorus sinuatus Dejean, 1829: 106.— Gemminger and Harold 1868: 266.— Putzeys 1878a: 27.— Csiki 1932: 1201.— Darlington 1934: 105.— Blackwelder 1944: 50.— Erwin and Sims 1984: 441.— Ball 1992: 85.— Lorenz 1998: 356.— Peck and Thomas 1998: 22.— Lorenz 2005: 377.— Peck 2005: 32.— Peck 2006: 17.— Ivie et al. 2008: 238.— Perez-Gelabert 2008: 80.— Turnbow and Thomas 2008: 15.— Peck 2011: 13.— Bousquet 2012: 1145.
Selenophorus parumpunctatus Dejean, 1829: 104. TYPE MATERIAL: 2 specimens, in Chaudoir-Oberthür Collection (MNHP), in front of the following box label: sinuatus/ Schonh/ Antilles/ C. Dejean// LECTOTYPE l (here selected) labelled [female]// parumpunctatus m [green paper]//.— Gemminger and Harold 1868: 266.— Gundlach 1894: 293.— Csiki 1932: 1200.— Blackwelder 1944: 50.— Erwin and Sims 1984: 440.— Lorenz 1998: 356.— Lorenz 2005: 377.— Bousquet 2012: 1145.
Selenophorus excisus LeConte, 1878: 377. [Primary junior homonym of S. excisus Putzeys, 1878a: 59]. LECTOTYPE female here selected, labelled: Fla// Type/ 5918 [red paper] // S.excisus/ LeC [handwritten]// [MCZC, LeConte Collection]. Synonymy established by Ball, in Bousquet 2012: 1145.— Csiki 1932: 1199. — Lorenz 1998: 355. — Lorenz 2005: 377. — Bousquet 2012: 1145.
Selenophorus mustus Casey, 1914: 152. LECTOTYPE [selected by Lindroth 1975: 141]: female, labelled Biscayne/ Fla// Casey/ bequest/ 1925// TYPE USNM/ 47889 [red paper]// mustus/ Csy [handwritten]// (USNM).— Casey 1918: 413 [junior synonym of S. excisusLeConte 1878].— Csiki 1932: 1199.— Lorenz 1998: 355.— Peck and Thomas 1998: 22.— Lorenz 2005: 377.— Bousquet 2012: 1145.

###### Type area.

Dejean was uncertain if his specimens of *S. parumpunctatus* were American or West Indian. The type area is restricted here to the island of Hispaniola.

###### Diagnosis.

Readily distinguished from *S. obtusoides* by a combination of: slightly larger size, the rounded posteriolateral angles of the pronotum and setigerous punctures of striae 2, 5 and 7 less foveate.

###### Descriptive notes.

Data for SBL in Table 1. Habitus as in Fig. 54B. Clypeus and labrum with anterior margin of each shallowly concave. Antennae with 1–3 basal antennomeres testaceous, antennomeres 4–11 darker; palpi infuscated, rufous to brunneous, tips testaceous; legs testaceous to rufous. Dorsal surface brunneous to brunneo-piceous with very faint brassy luster; ventral surface paler, rufous to brunneous; elytral epipleuron paler than disc. Head with mesh pattern isodiametric; pronotum with mesh pattern slightly transverse, sculpticells about 2× wide as long; elytra with mesh pattern transverse, sculpticells about 3–4× wide as long. Pronotum with posteriolateral impressions impunctate. Elytral striae impunctate, except the standard setigerous punctures in striae 2, 5 and 7. Males with two terminal setae and females with four terminal setae near the posterior margin on sternum VII.

**Male genitalia.** Fig. 55D–F. Apical portion of phallic median lobe moderately long, triangular, symmetrically rounded in dorsal/ventral aspect, with medial longitudinal bulge dorsally, slightly so ventrally; endophallus with four to eight spines with large bases; without lamina; ostium anopic.

**Ovipositor and female reproductive tract.** Fig. 56. Gonocoxite 2 (**gc2**) moderately thick, slightly falcate. Bursa copulatrix (**bc**) moderately long, recurved; long spermatheca (**sp**) originating near base of common oviduct (**co**), without distinctive narrowing basally; markedly long spermathecal gland duct (**spgd**) originating above base of spermatheca. Spermathecal gland (**spg**) very small, bulbous, with moderately large swelling of duct basad gland.

###### Geographical distribution.

Fig. 57. This wide-ranging species is found on most of the island groups in the West Indies.

###### Chorological affinities and relationships.

The West Indian range of this widely distributed species overlaps the range of *S. obtusoides*. Relationships of *S. parumpunctatus* are not postulated beyond species group membership.

###### Material examined.

In addition to type material, we have seen a total of 9,864 specimens (4,637 males, 5,222 females, 5 unknown). See Appendix for details.

##### Selenophorus striatopunctatus

Taxon classificationAnimaliaColeopteraCarabidae

species group

###### Recognition.

Striae 1–7 of elytra distinctly punctate.

**SBL.** Males, 5.20–6.04 mm, females 5.28–6.24 mm.

**Color.** Antennae with antennomere1 testaceous to rufo-testaceous, antennomeres 2–11 darker; mouthparts infuscated or not, testaceous to rufo-testaceous; legs testaceous to dark rufo-testaceous. Head and pronotum brunneous to brunneo-piceous; elytra brunneo-piceous to nearly piceous, suture and apical margin diffusely paler. Ventral surface rufo-brunneous to brunneo-piceous; elytral epipleuron paler than disc.

**Luster.** Pronotum with faint bluish metallic luster; elytra with faint to moderate iridescence; ventral surface faintly iridescent.

**Dorsal microsculpture.** Head shiny, with mesh pattern isodiametric, microlines very fine; pronotum shiny, with mesh pattern slightly transverse, sculpticells about 1.5–2× wide as long, microlines very fine; elytra very shiny, microlines not visible at 100×.

**Male genitalia.** Apical portion of phallic median lobe short, broad, apex symmetrically rounded in dorsal/ventral aspects; endophallus with 17 spines with large bases scattered throughout entire length; without lamina. Ventral surface of shaft smooth.

**Ovipositor and female reproductive tract.** Gonocoxite 2 moderately thick, slightly falcate. Bursa copulatrix markedly long; spermatheca moderately long, coiled, sausage-like, originating near base of common oviduct; markedly long spermathecal gland duct originating above base of spermatheca. Spermathecal gland somewhat dumbbell-like, narrowed in the middle.

###### Included species.

The *striatopunctatus* species group includes only one species in the West Indies: *S. striatopunctatus* Putzeys.

###### Geographic distribution.

In the West Indies, this species group is recorded from most of the islands.

##### Selenophorus striatopunctatus

Taxon classificationAnimaliaColeopteraCarabidae

Putzeys

**Figure 58.**
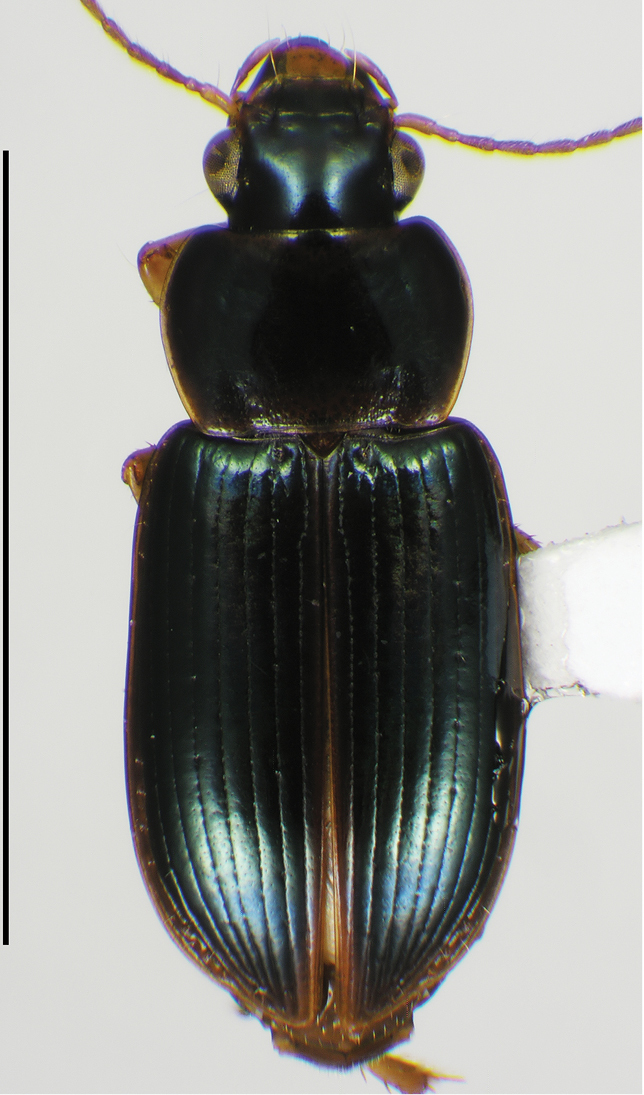
Habitus digital image of *Selenophorus striatopunctatus* species group, dorsal aspect, *S. striatopunctatus* Putzeys. Scale bar 5 mm.

**Figure 59.**
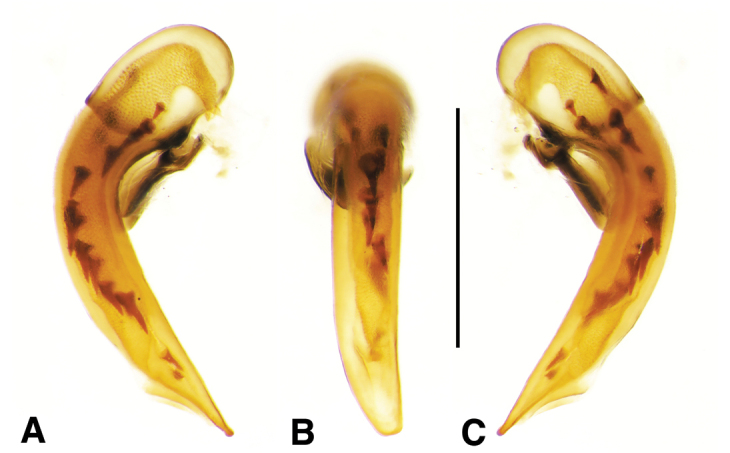
Digital images of male genitalia of *Selenophorus striatopunctatus* species group, *S. striatopunctatus* Putzeys. **A** right lateral aspect **B** dorsal aspect **C** left lateral aspect. Scale bar 1 mm.

**Figure 60.**
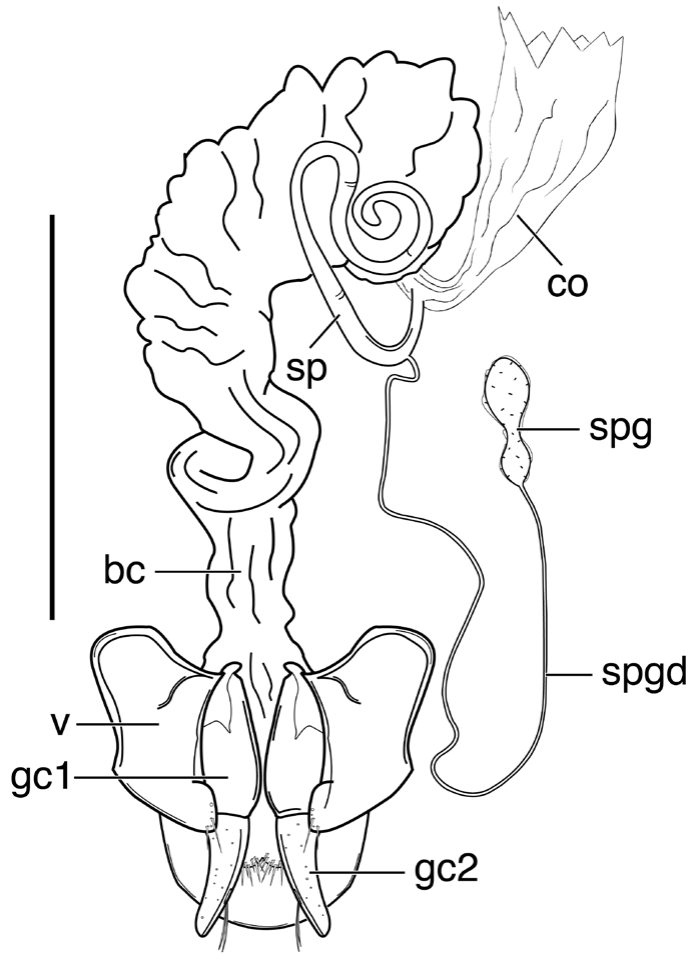
Line drawing of female reproductive tract of *Selenophorus striatopunctatus* species group, ventral aspect, *S. striatopunctatus* Putzeys. Legend: **bc** bursa copulatrix **co** common oviduct **gc1** gonocoxite 1 **gc2** gonocoxite 2 **sp** spermatheca **spg** spermathecal gland **spgd** spermathecal gland duct **v** valvifer. Scale bars 1 mm.

**Figure 61.**
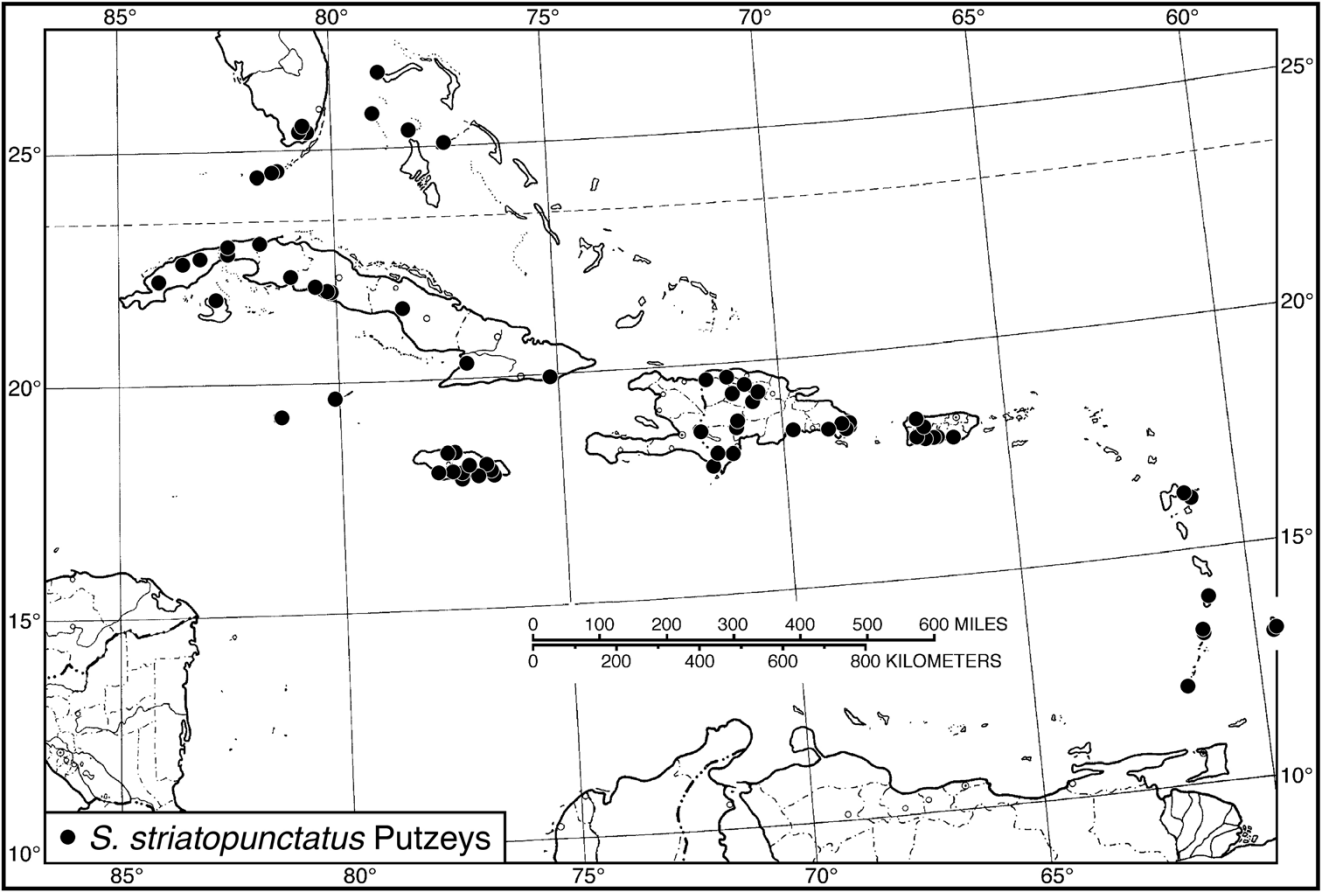
Map of West Indies showing known localities for species of *Selenophorus striatopunctatus* species group.

Figs 58 , 59A–C , 60 , 61

Selenophorus striatopunctatus Putzeys, 1878a: 33. SYNTYPES (5) in the Putzeys Collection (IRSB), and (5) in the Chaudoir-Oberthür Collection (MNHP). IRSB specimens as follows. 1, male [[indecipherable writing] VII.44 [green paper] // Putzeys Collection label// Type//; 2, male, Chiapas/ 5.7.58 Putzeys Collection label// Type// ; 3, Costarico [green paper]// Putzeys Collection label// Type//; 4, male, St. Doming [green paper]// Putzeys Collection label// Type//.— Amblygnathus puncticollis Putz./Emd. det, 1937//.— 5, male, Mex / 3.7.44// Putzeys Collection label// Type// [specimen a Pelmatellus sp.]; LECTOTYPE (here selected), specimen #2, above. MNHP specimens as follows. Box label striatopunctatus/ Chaud./ Antilles. 1, male, labelled Rep. Dominginie/ Sallé// 293// 2, 402//; 3, male, labelled Mexique// A. Deyrolle//; 4, female, unlabelled; 5, female, labelled Mexique/.— Csiki 1932: 1201.— Darlington 1934: 104.— Blackwelder 1944: 50.— Erwin and Sims 1984: 441.— Bennett and Alam 1985: 20.— Ball 1992: 85.— Lorenz 1998: 356.— Peck and Thomas 1998: 22.— Lorenz 2005: 377.— Peck 2005: 33.— Perez-Gelabert 2008: 80.— Turnbow and Thomas 2008: 15.— Peck 2009: 13.— Bousquet 2012: 1147.
Hemisopalus vigilans Casey, 1914: 137. LECTOTYPE (here selected) male, labelled Fla// CASEY/ bequest/ 1925// TYPE USNM/ 47869 [red paper]// vigilans/ Csy// (USNM).— Peck and Thomas (1998: 22).— Bousquet 2012: 1147.
Hemisopalus depressulus Casey, 1914: 137. LECTOTYPE selected by Lindroth (1975: 141) male, labelled Fla// Casey/ bequest/ 1925// TYPE USNM/ 47867 [red paper]// depressulus/ Csy [handwritten] (USNM); (synonymy established by Peck and Thomas (1998: 22).— Csiki 1932: 1199.— Ball and Maddsion 1987: 206.— Lorenz 1998: 355.— Lorenz 2005: 377.— Bousquet 2012: 1147.

###### Type area.

State of Chiapas, Mexico.

###### Diagnosis.

This species is readily separated from the other *Selenophorus* species by the punctate elytral striae.

###### Descriptive notes.

Data for SBL in Table 1. Habitus as in Fig. 58. Clypeus and labrum with anterior margin of each shallowly concave. Antennae with antennomere 1 testaceous to rufo-testaceous, antennomeres 2–11 darker; mouthparts infuscated or not, testaceous to rufo-testaceous; legs testaceous to dark rufo-testaceous. Head and pronotum brunneous to brunneo-piceous; elytra brunneo-piceous to nearly piceous, suture and apical margin diffusely paler. Ventral surface rufo-brunneous to brunneo-piceous; elytral epipleuron paler than disc. Pronotum with faint bluish metallic luster; elytra with faint to moderate iridescence; ventral surface faintly iridescent. Head shiny, with mesh pattern isodiametric, microlines very fine; pronotum shiny, with mesh pattern slightly transverse, sculpticells about 1.5–2× wide as long, microlines very fine; elytra very shiny, microlines not visible at 100×. Pronotum with posteriolateral impressions punctate; hind angles rounded. Elytral striae punctate, in addition to the standard setigerous punctures in striae 2, 5 and 7. Elytral intervals finely punctate. Males with two terminal setae and females with four terminal setae near the posterior margin on sternum VII.

**Male genitalia.** Fig. 59A–C. Apical portion of phallic median lobe short, broad, apex symmetrically rounded in dorsal and ventral aspects; endophallus with 17 spines with large bases scattered throughout entire length; without lamina. Ventral surface of shaft smooth.

**Ovipositor and female reproductive tract.** Fig. 60. Gonocoxite 2 (**gc2**) moderately thick, slightly falcate. Bursa copulatrix (**bc**) markedly long; spermatheca (**sp**) moderately long, coiled, sausage-like, originating near base of common oviduct (**co**) ; markedly long spermathecal gland duct (**spgd**) originating above base of spermatheca. Spermathecal gland (**spg**) somewhat dumbbell-like, narrowed in the middle.

###### Geographical distribution.

Fig. 61. This wide-ranging species is found on most of the island groups in the West Indies, with the exception of the islands located between the Greater Antillean Puerto Rico and Lesser Antillean Guadeloupe.

###### Chorological affinities and relationships.

The range of this species overlaps the ranges of most *Selenophorus* species. Relationships of *S. striatopunctatus* are not postulated beyond species group membership.

###### Material examined.

In addition to type material, we have seen a total of 803 specimens (398 males, 405 females). See Appendix for details.

##### Genus Stenomorphus

Taxon classificationAnimaliaColeopteraCarabidae

Dejean

Stenomorphus Dejean, 1831: 696. TYPE SPECIES: Stenomorphus angustatus Dejean (by monotypy).— Gemminger and Harold 1868: 385.— Csiki 1932: 1080.— Blackwelder 1944: 47.— Noonan 1976: 42.— Reichardt 1977: 429.— Erwin and Sims 1984: 441.— Noonan 1985a: 46.— Ball et al. 1991: 939.— Lorenz 1998: 357.— Lorenz 2005: 378.
Agaosoma Ménétries, 1843: 63. TYPE SPECIES: Stenomorphus californicum Ménétries (by monotypy).
Agaasoma Chenu, 1851: 134 (misspelling).

###### Recognition.

The very long, narrow, cylindrical body, and elongated pronotum, distinctly longer than wide (Pl/PW = 1.07–1.45) and serial punctures only in striae 2 and 5, readily distinguish members of this genus from other selenophorine genera. Males with biseriate adhesive vestiture only on fore-tarsi. Additionally, females have gonocoxite 2 bifurcate apically, and the basitarsus of the fore-tarsi expanded, about twice the width of tarsomere 2.

###### Included species.

Only two taxa of *Stenomorphus* are recorded from the West Indies: *S. californicus manni* Darlington and S. *cubanus* Darlington.

###### Chorological affinities and relationships.

See Ball et al. (1991: 981–982) for a discussion of these topics. The two Greater Antillean taxa of *Stenomorphus* being closely allopatric (*S. cubanus*, confined to Cuba, and *S. californicus manni*, confined to Hispaniola) would seem to suggest that they are adelphotaxa, but their relationships indicate a more complex situation, with each island being occupied independently and at a markedly different time.

###### Geographical distribution.

In the West Indies, this species group is recorded only from the Greater Antillean islands of Cuba and Hispaniola.

##### Stenomorphus californicus

Taxon classificationAnimaliaColeopteraCarabidae

Ménétriés

###### Remarks.

This species is wide-ranging in the Middle American and North American lowlands, where it is represented by three subspecies. Additionally, it is represented in the Greater Antilles by *S. c. manni* Darlington, that is treated below.

##### Stenomorphus californicus manni

Taxon classificationAnimaliaColeopteraCarabidae

Darlington

**Figure 62.**
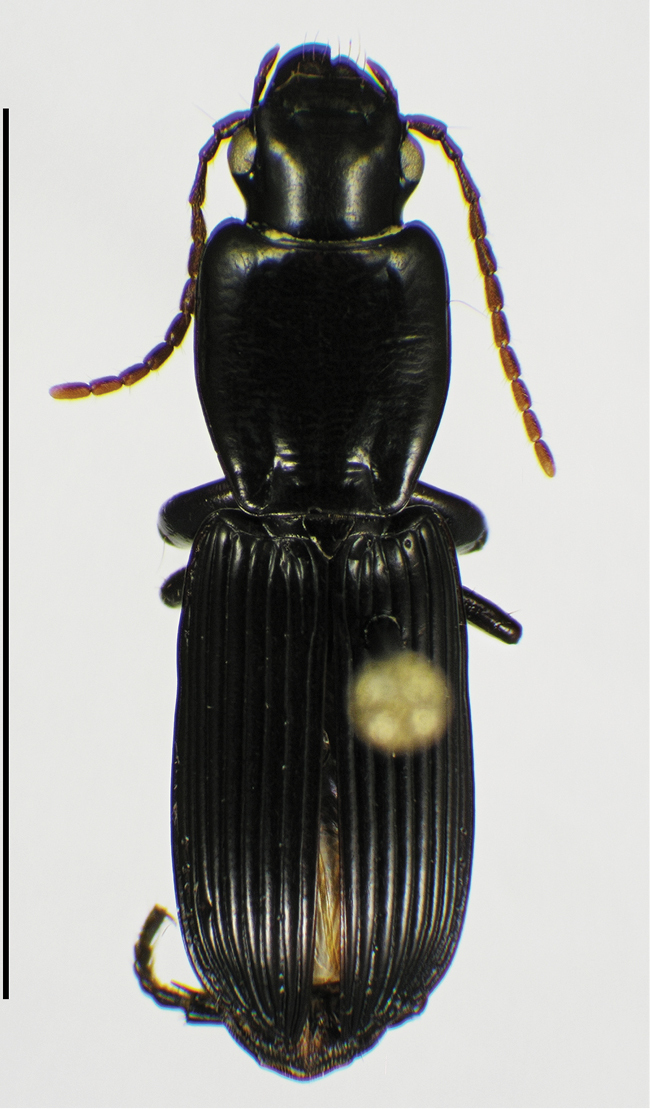
Habitus digital images of *Stenomorphus californicus manni* Darlington, dorsal aspect. Scale bar 10 mm.

**Figure 63.**
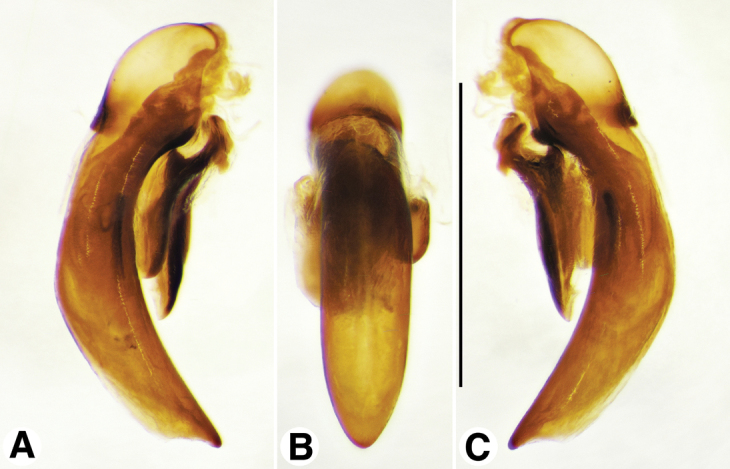
Digital images of male genitalia of *Stenomorphus californicus manni* Darlington. **A** right lateral aspect **B** dorsal aspect **C** left lateral aspect. Scale bar 1 mm.

Figs 62 , 63A–C , 65

Stenomorphus manni Darlington, 1934: 102. HOLOTYPE male, labeled: “Manneville/ Hayti Mann.”; “1925/ MCZ/ HoloType Stenomorphus/ manni Darl.” [name handwritten; red paper]; “Stenomorphus/ manni/ Drl.” [handwritten] (MCZC).— Blackwelder 1944: 48.— Erwin and Sims 1984: 441.— Ball et al. 1991: 961.— Ball 1992: 85.— Lorenz 1998: 357.— Lorenz 2005: 378.—Perez-Gelabert 2008: 80.

###### Type locality.

Manneville, Ouest Department, Haiti, Hispaniola.

###### Diagnosis.

With features in “Recognition” for the genus, and specimens found on the Greater Antillean island of Hispaniola.

###### Descriptive notes.

Data for SBL in Table 1. Habitus as in Fig. 62. The membranous hind wings are folded, not reduced in length. Both males and females with four terminal setae near the posterior margin on sternum VII.

**Male genitalia.** Fig. 63A–C. Apical portion of phallic median lobe short, broadly tapered in dorsal aspect; endophallus with small basal darkened microtrichial field, best viewed in ventral aspect (endophallus inverted), without spines; without lamina.

**Figure 64.**
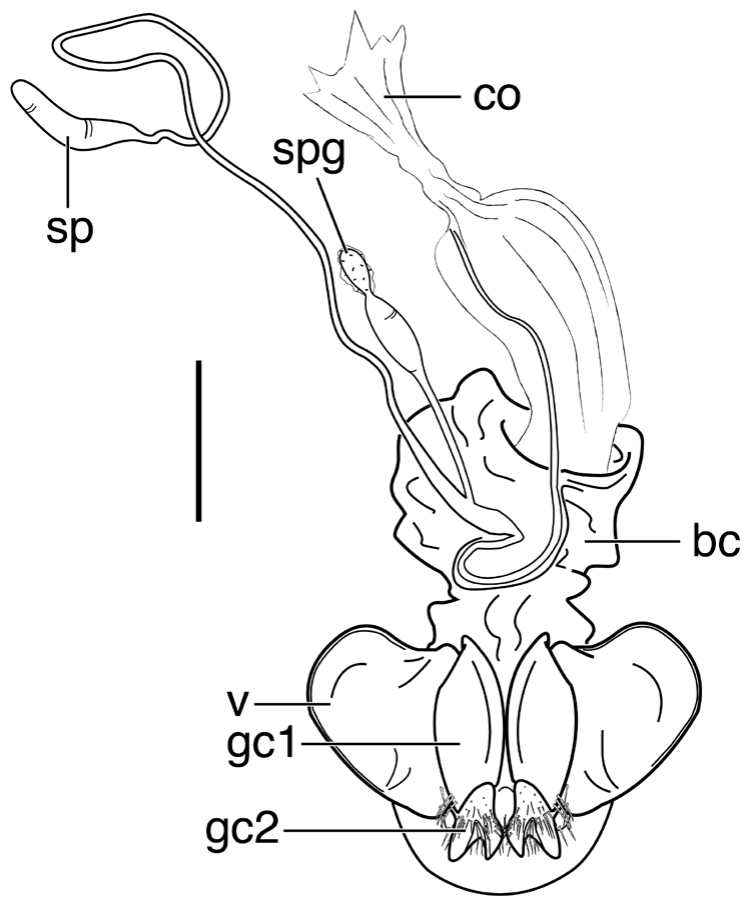
Line drawing of female reproductive tract of *Stenomorphus californicus rufipes* LeConte, ventral aspect. Legend: **bc** bursa copulatrix **co** common oviduct **gc1** gonocoxite 1 **gc2** gonocoxite 2 **sp** spermatheca **spg** spermathecal gland **v** valvifer. Scale bar 1 mm.

**Ovipositor and female reproductive tract.** Very similar to that of *S. californicus rufipes*, which is illustrated, Fig. 64. Gonocoxite 2 (**gc2**) short, broad, apically bifurcate, slightly falcate. Spermathecal duct originating from common oviduct (**co**), with proximal one sixth attached to common oviduct, branching to spermathecal gland duct (**spgd**). Spermathecal duct above this branching is about 2× as long as the spermathecal gland duct + spermathecal gland. Spermatheca (**sp**) sausage-like; spermathecal gland (**spg**) small, more or less bulbous, with swelling of duct basad gland.

###### Geographical distribution.

**Figure 65.**
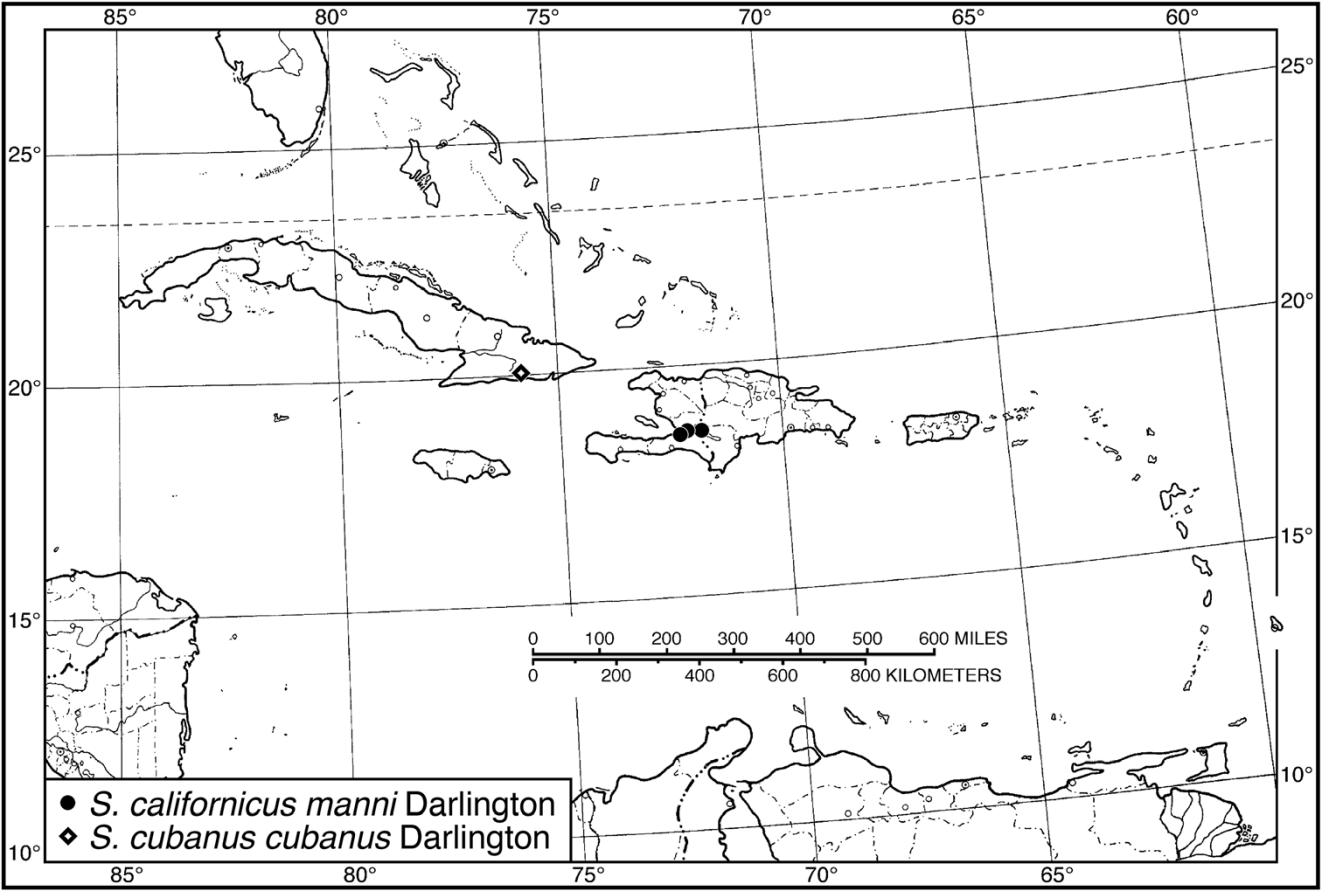
Map of West Indies showing known localities for species of *Stenomorphus* Dejean.

Fig. 65. This species is known only from Haiti, at Manneville and Port au Prince and vicinity, and western Dominican Republic, at Los Pinos.

###### Chorological affinities and relationships.

See this topic under genus *Stenomorphus*.

###### Material examined.

In addition to material reported in Ball et al., (1991: 961), we have seen 6 specimens (3 males, 3 females). See Appendix for details.

##### Stenomorphus cubanus

Taxon classificationAnimaliaColeopteraCarabidae

Darlington

Fig. 65

Stenomorphus cubanus Darlington, 1937: 135. HOLOTYPE male, labelled: “Cauto el Cristo/ (Cauto R.) Ote./ Aug. 16, 1936”; “Cuba 1936/ Darlington/ Collector”; “22488/ M.C.Z./ HoloType/ Stenomorphus/ cubanus D.” [name handwritten; on red paper]; “Stenomorphus cubanus Darl.” [handwritten] (MCZC).— Blackwelder 1944: 47.— Erwin and Sims 1984: 441.— Ball et al. 1991: 952.— Ball 1992: 85.— Lorenz 1998: 357.— Lorenz 2005: 378.—Peck 2005: 32.

###### Type locality.

Cauto el Cristo, Santiago de Cuba Province, Cuba. (Cauto el Cristo was previously in Oriente Province).

###### Diagnosis.

With features in “Recognition” for the genus, and found on the Greater Antillean island of Cuba.

###### Descriptive notes.

Data for SBL in Table 1. Habitus similar to that of *S. californicus manni* (Fig. 62). In the original description, Darlington stated that the membranous hind wings were vestigial, reaching slightly past the middle of the elytra. Both males and females with four terminal setae near the posterior margin on sternum VII.

**Male genitalia.** Phallic median lobe similar to that of *S. californicus manni*, Fig. 63A–C; differences expected in everted endophallus.

**Ovipositor and female reproductive tract.** Not Studied.

###### Geographical distribution.

Fig. 65. This species is known only from the type series collected in southeastern Cuba.

###### Chorological affinities and relationships.

See this topic under genus *Stenomorphus*.

###### Material examined.

We have not seen any material other than the specimens reported in Ball et al. (1991: 952).

##### Genus Discoderus

Taxon classificationAnimaliaColeopteraCarabidae

LeConte

Discoderus LeConte, (1853: 381). TYPE SPECIES: Selenophorus parallelus Haldeman (designation by Lindroth 1968: 830).— Gemminger and Harold 1868: 265.— Csiki 1932: 1039.— Blackwelder 1944: 46.— Noonan 1976: 41.— Reichardt 1977: 428.— Erwin and Sims 1984: 441.— Noonan 1985a: 47.— Lorenz 1998: 357.— Lorenz 2005: 378.
Selenalius Casey, 1914: 135, 153. TYPE SPECIES: Discoderus cordicollis Horn (by original designation).— Csiki 1932: 1196.— Blackwelder 1944: 49.— Noonan 1976: 41.— Reichardt 1977: 428.— Erwin and Sims 1984: 440.— Lorenz 1998: 357.— Lorenz 2005: 378.— Bousquet 2012: 1148.

###### Recognition.

Lindroth (1968: 830–831) noted the following features: Selenophori with pronotum discoid, posteriolateral angles evenly rounded, fore tibiae more widened apically than in *Selenophorus*, terminal spines stouter. Most species lack the elytral parascutellar stria. Males have the middle tibiae markedly arcuate, with series of small tubercles along the inner edge. Males of many species do not have the fore- and mid-tarsi dilated, and fore pair with only rudiments of adhesive vestiture. The male genitalia are characterized as follows: phallic median lobe slender, apex with tip narrowly obtuse in dorso/ventral aspect, acute in lateral aspect; endophallus without spines; without lamina. *Discoderus* females have a median enlarged plate-like area at the apex of abdominal sternum VII. Gonocoxite 2 of the ovipositor is short, thick, and lateral surface broad, concave, with transverse ridges. The internal reproductive tract of females are characterized as follows: spermathecal gland duct elongate; spermathecal gland elongate, in medial section markedly constricted.

###### Included species.

Only four species of *Discoderus* are recorded in the West Indies: *D. beauvoisii* (Dejean), *D. cinctus* (Putzeys), *D. cyaneopacus* (Darlington) and *D. thoracicus* (Putzeys).

###### Geographical distribution.

The West Indian range of this genus includes the islands of the Bahamas, Caymans, and Greater Antilles.

##### Discoderus beauvoisii

Taxon classificationAnimaliaColeopteraCarabidae

(Dejean)

**Figure 66.**
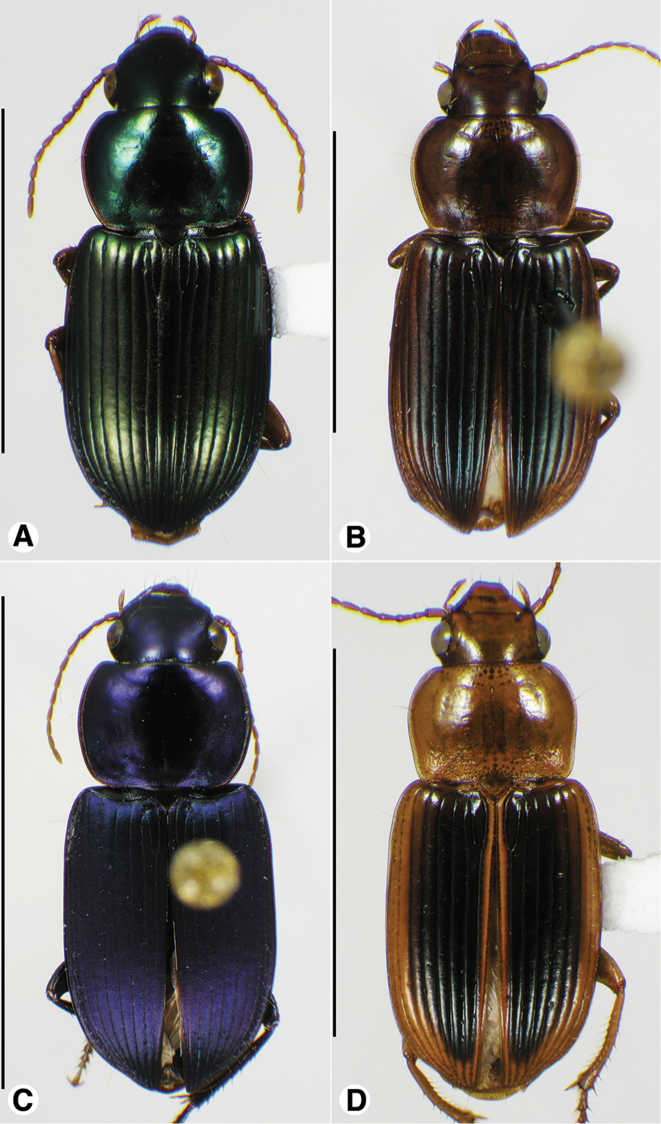
Habitus digital images of *Discoderus* species, dorsal aspect. **A** *D. beauvoisii* (Dejean) **B** *D. cinctus* (Putzeys) **C** *D. cyaneopacus* (Darlington) **D** *D. thoracicus* (Putzeys). Scale bars: **A, B, D** 5 mm; **C** 10 mm.

**Figure 67.**
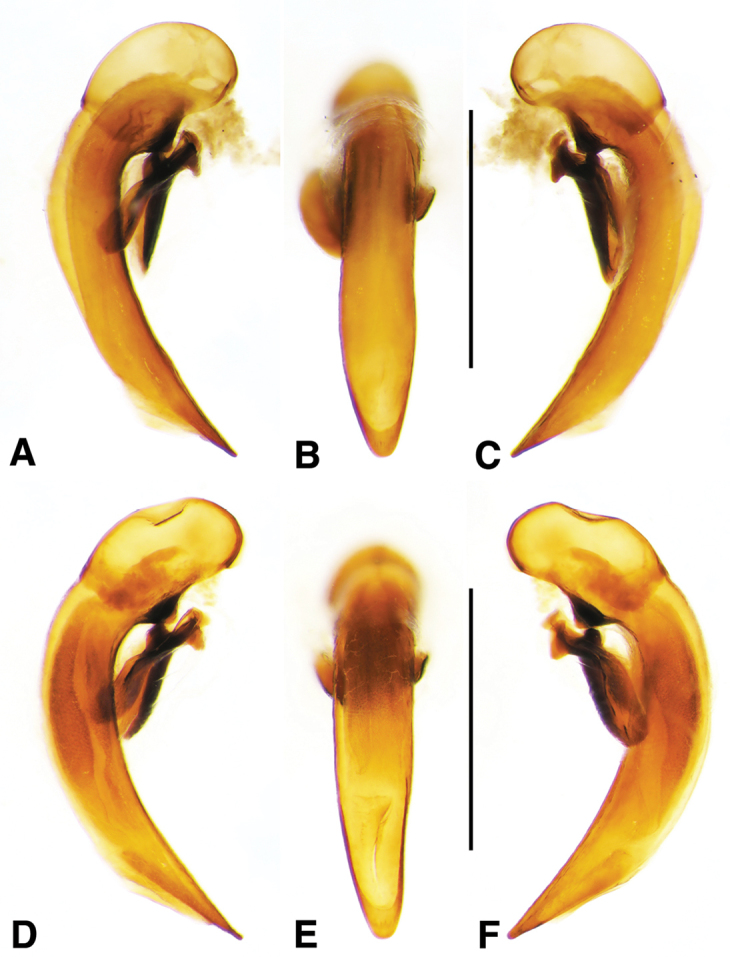
Digital images of male genitalia of *Discoderus* species. **A, D** right lateral aspect **B, E** dorsal aspect **C, F** left lateral aspect. **A–C** *D. beauvoisii* (Dejean) **D–F** *D. cinctus* (Putzeys). Scale bars 1 mm.

Figs 66A , 67A–C , 69 , 70

Selenophorus beauvoisii Dejean, (1829: 98) In the Chaudoir-Oberthür Collection, 25 specimens (4, of special note) in front of the following box label: Beauvoisii/ Dej./ Antilles/ C. Dejean//. LECTOTYPE (here selected) [male symbol] // beauvoisi mihi/ pensylvanicus mihi cat./ in Amer bor D. Beauvois//; also, a male, labelled aeneocupreus, in Jamaica (details below); also, a male, piciventris, S. Dominic, Mannerheim [Dejean Coll. label name, only – see Putzeys 1878a: 47]; also, a male, xanthoxcelis aeneocupreus, S. Dominic [Dejean Coll. label name, only – see Putzeys 1878a: 47].— Gemminger and Harold 1868: 265.— LeConte 1870: 403.— Putzeys 1878a: 46.— Csiki 1932: 1196.— Darlington 1934: 105.— Blackwelder 1944: 49.— Erwin and Sims 1984: 440.— Lorenz 1998: 355.— Lorenz 2005: 376.— Peck 2005: 32.— Bousquet 2012: 1622.
Selenophorus aeneocupreus Dejean, 1829: 99. LECTOTYPE: male, labeled aeneocupreus Schrank/ in Jamaica [green paper]// Schonherr [green paper]// Putzeys 1878a: 46 [as a junior synonym of S. beauvoisii]; Gemminger and Harold 1868: 265.— Csiki 1932: 1196.— Blackwelder 1944: 49.— Erwin and Sims 1984: 440.— Lorenz 1998: 355.— Lorenz 2005: 376.— Bousquet 2012: 1622.
Discoderus beauvoisi ; Noonan 1985a: 49.— Ball 1992: 84, 85.— Perez-Gelabert 2008: 79.

###### Type area.

Dejean incorrectly recorded that *S. beauvoisii* is from North America, and compared it to *S. aeneocupreus*, which he stated as being from Jamaica. LeConte (1870: 403) asserted that *S. beauvoisii* was not known from North America, but was common in the West Indies. In view of the above considerations, the type area of *D. beauvoisii* is here restricted to Jamaica, the locality specified for the lectotype of *S. aeneocupreus*, that name a junior synonym of *S. beauvoisii*.

###### Diagnosis.

The smaller size, greenish to bluish metallic luster of the dorsum and pale legs readily separates this species from the three other West Indian *Discoderus* species.

###### Descriptive notes.

Data for SBL in Table 1. Habitus as in Fig. 66A. Clypeus and labrum each with anterior margin moderately concave. Antennae, mouthparts and legs testaceous to slightly darker. Ventral surface rufous to dark rufo-brunneous. Pronotum and elytra with greenish to bluish metallic luster; head with less metallic luster. Antennae and mouthparts rufo-testaceous to dark rufous. Ventral surface and legs piceous. Pronotum and elytra violaceous to bluish, with hints of green; head with less metallic reflection. Head, posteriolateral surface of pronotum and elytra with mesh pattern isodiametric; pronotal disc with mesh pattern slightly transverse, sculpticells about 2× wide as long. Elytral striae impunctate, except the standard setigerous punctures in striae 2, 5 and 7. Both males and females with four terminal setae near the posterior margin on sternum VII.

**Male genitalia.** Fig. 67A–C. Apical portion of phallic median lobe moderately long, narrowly triangular, symmetrically rounded in ventral/dorsal aspects; endophallus without spines or darkened microtrichial fields; without lamina.

**Ovipositor and female reproductive tract.** Fig. 69A. Gonocoxite 2 (**gc2**) of the ovipositor is short, thick, and lateral surface broad, concave, with transverse ridges. Bursa copulatrix (**bc**) moderately short; spermatheca (**sp**) originating near base of common oviduct (**co**), with proximal one third attached to common oviduct; spermathecal gland duct (**spgd**) originating below inflated portion of spermatheca. Spermathecal gland (**spg**) with moderately long duct, gland sausage-like, with slight swelling of duct basad gland.

###### Geographical distribution.

Fig. 70. This species ranges throughout the Greater Antilles islands (Cuba to the Virgin Islands) and on Mayaguana Island of the Bahamas

###### Chorological affinities and relationships.

The range of this species overlaps the ranges of the three other West Indian *Discoderus* species. The bright metallic luster of the dorsal surface of the body shared by members of *D. beauvoisii* and *D. cyaneopacus* may indicate close relationship between these two species.

###### Material examined.

In addition to type material, we have seen a total of 1,875 specimens (877 males, 998 females). See Appendix for details.

**Figure 68.**
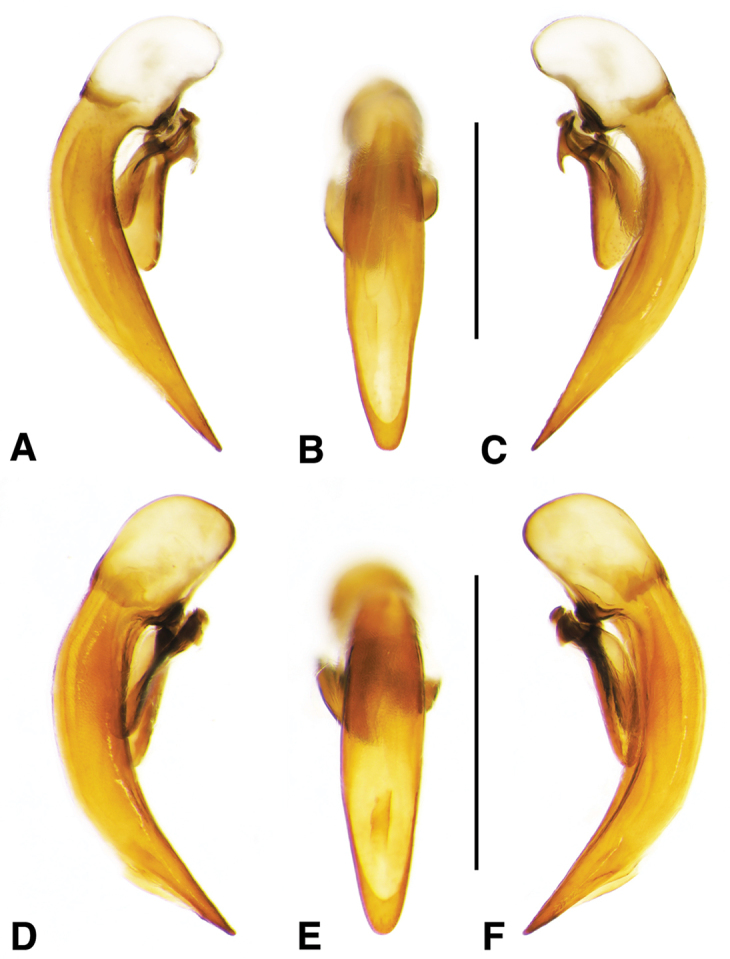
Digital images of male genitalia of *Discoderus* species, **A** and **D** right lateral aspect **B, E** dorsal aspect **C** and **F** left lateral aspect. **A–C** *D. cyaneopacus* (Darlington) **D–F** *D. thoracicus* (Putzeys). Scale bars 1 mm.

**Figure 69.**
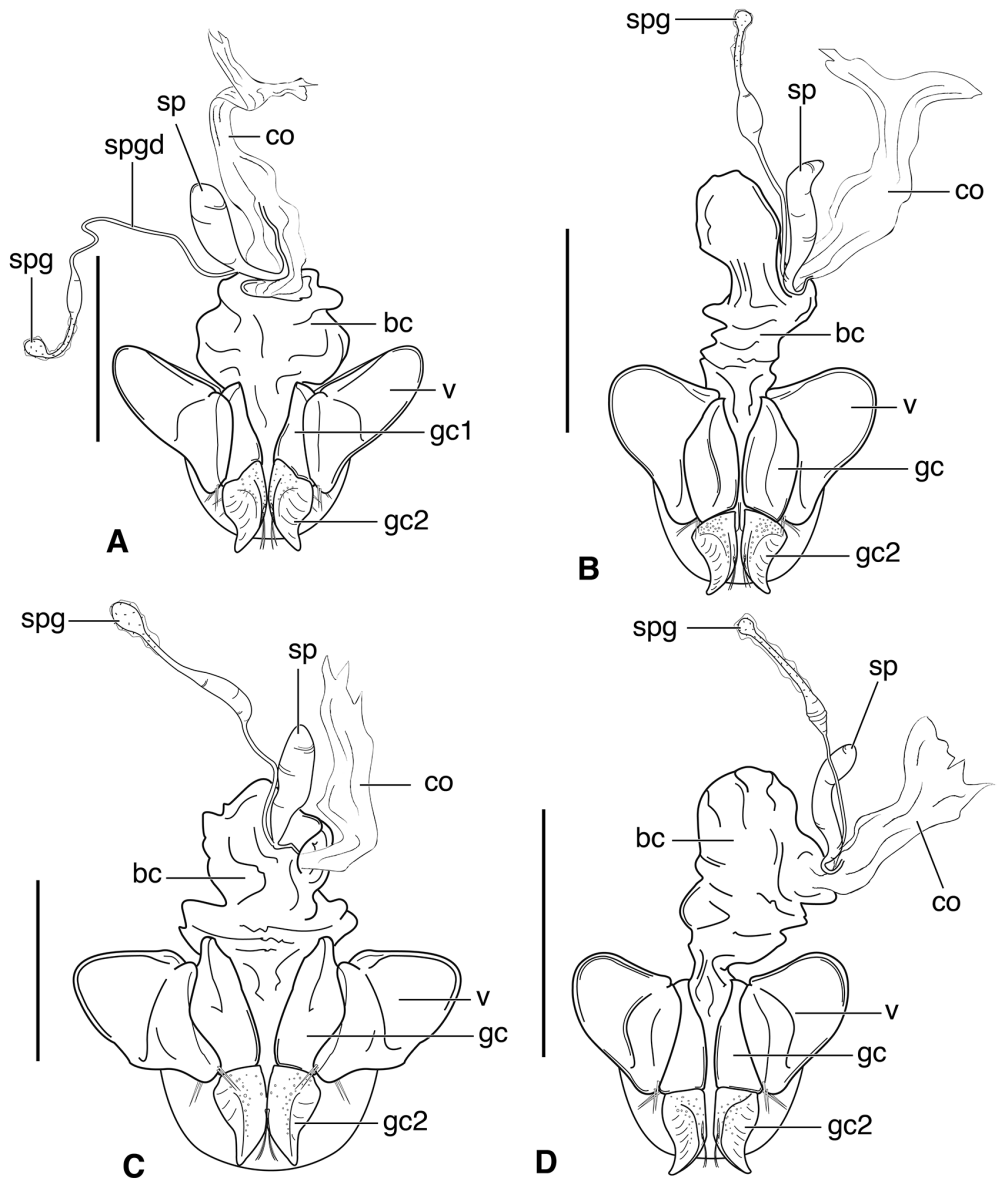
Line drawings of female reproductive tract of *Discoderus* species, ventral aspect. **A** *D. beauvoisii* (Dejean) **B** *D. cinctus* (Putzeys) **C** *D. cyaneopacus* (Darlington) **D** *D. thoracicus* (Putzeys). Legend: **bc** bursa copulatrix **co** common oviduct **gc1** gonocoxite 1 **gc2** gonocoxite 2 **sp** spermatheca **spg** spermathecal gland **spgd** spermathecal gland duct **v** valvifer. Scale bars 1 mm.

**Figure 70.**
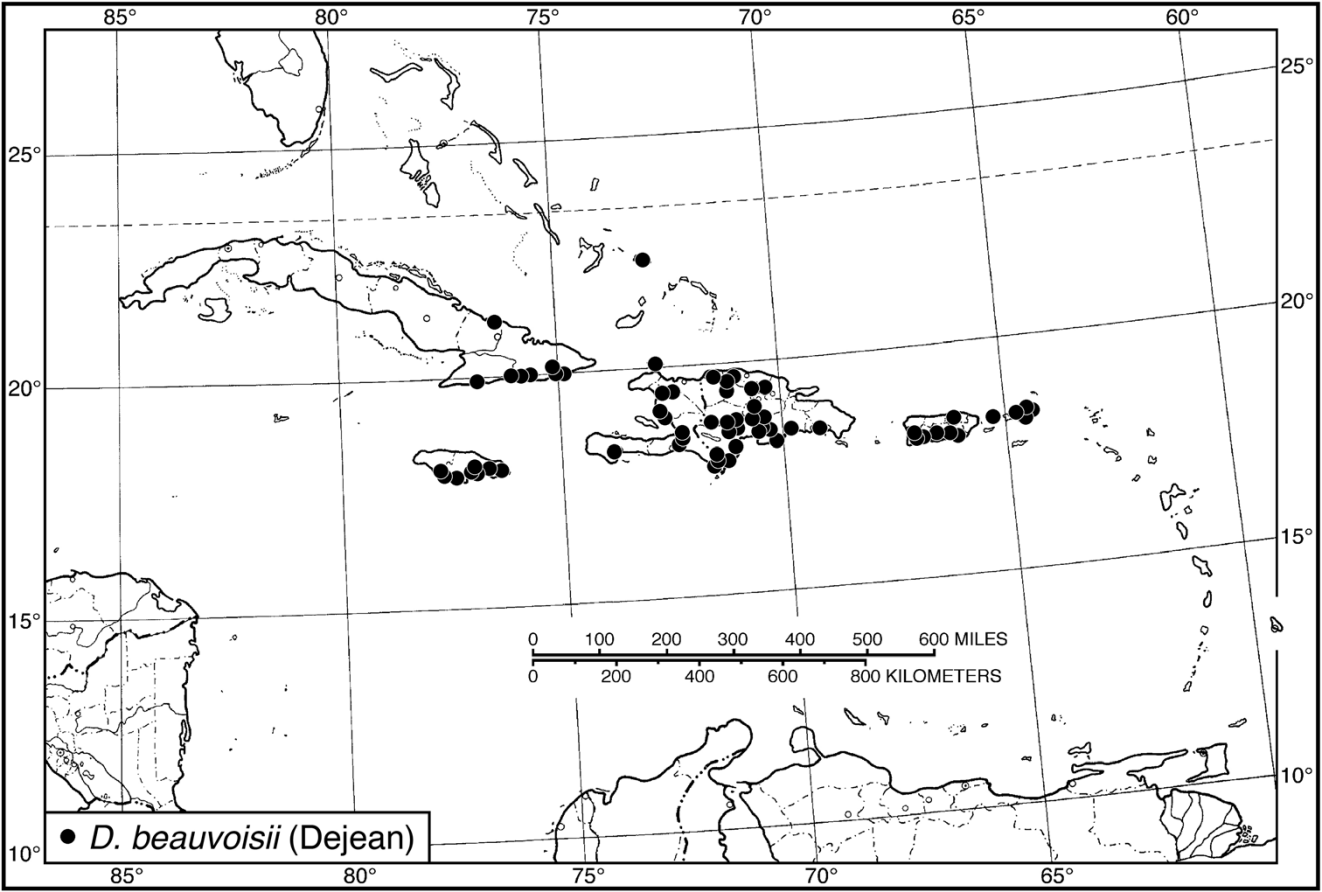
Map of West Indies showing known localities for species of *Discoderus* LeConte, in part.

##### Discoderus cinctus

Taxon classificationAnimaliaColeopteraCarabidae

(Putzeys)

**Figure 71.**
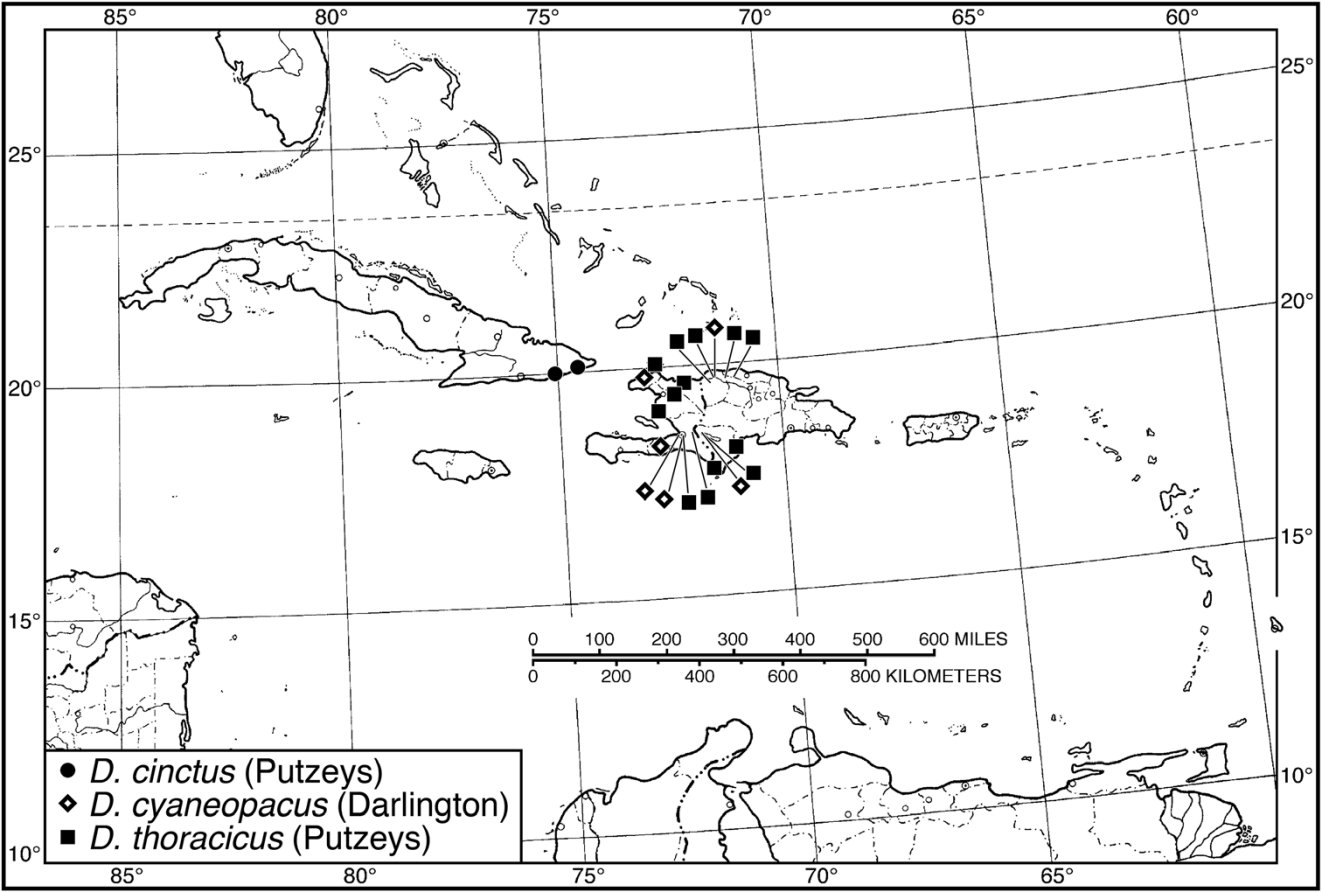
Map of West Indies showing known localities for species of *Discoderus* LeConte, in part.

Figs 66B , 67D–F , 69B , 71

Selenophorus cinctus Putzeys, 1878a: 45. In the Chaudoir-Oberthür Coll., one specimen in front of following box label: //cinctus/ Chaud/ Cuba/ A. Deyrolle.// LECTOTYPE (here selected) male, labelled: //Cuba//; //Ex Museo/ Chaudoir// (MNHP).— Csiki 1932: 1197.— Darlington 1934: 104.— Blackwelder 1944: 49.— Erwin and Sims 1984: 440.— Lorenz 1998: 355.— Lorenz 2005: 376.— Peck 2005: 32.
Discoderus cinctus ; Ball 1992: 84, 85.

###### Type area.

Cuba.

###### Diagnosis.

More robust habitus and matte surfaces of head and pronotum with easily visible microsculpture readily separates *D. cinctus* from the similarly colored, but paler, and allopatric *D. thoracicus* (Fig. 66B; cf. Fig. 66D). The posteriolateral angles of the pronotum are more broadly rounded than those of *D. thoracicus*. Although range of SBL overlaps broadly for these two species, there is a distinct average size difference as well, with members of *D. cinctus* the larger (SBL, Table 1). The pale, non-metallic dorsal color pattern distinguishes this species pair from the other two West Indian species of *Discoderus*.

###### Descriptive notes.

Data for SBL in Table 1. Habitus as in Fig. 66B. Labrum with anterior margin moderately concave; clypeus with anterior margin shallowly concave. Antennae, mouthparts and legs testaceous to slightly darker. Head, pronotum and ventral surface rufo-testaceus to rufo-brunneous. Elytra rufo-testaceus to rufo-brunneous, with darker median cloud in intervals 2–6; cloud with faint greenish to bluish metallic luster. Head and posteriolateral surface of pronotum with mesh pattern isodiametric; pronotal disc with mesh pattern slightly transverse, sculpticells about 2× wide as long; elytra with mesh pattern slightly transverse, sculpticells about 1.5× wide as long. Elytral striae impunctate, except the standard setigerous punctures in striae 2, 5 and 7. Males with two terminal setae and females with four terminal setae near the posterior margin on sternum VII.

**Male genitalia.** Fig. 67D–F. Apical portion of phallic median lobe moderately long, narrowly triangular, symmetrically rounded in ventral/dorsal aspects; endophallus with darkened microtrichial field visible in right lateral aspect; without lamina.

**Ovipositor and female reproductive tract.** Fig. 69B. Very similar to that of *D. beauvoisii*. For details, see this topic for *D. beauvoisii*, above.

###### Geographical distribution.

Fig. 71. This species is known only from the southeastern tip of Greater Antillean Cuba.

###### Chorological affinities and relationships.

The range of this species is overlapped by the range of *D. beauvoisii*, but is geographically isolated from what would seem to be its closest relative, the Hispaniolan *D. thoracicus*.

###### Material examined.

In addition to type material, we have seen a total of 82 specimens (30 males, 52 females). See Appendix for details.

##### Discoderus cyaneopacus

Taxon classificationAnimaliaColeopteraCarabidae

(Darlington)

Figs 66C , 68A–C , 69C , 71

Selenophorus cyaneopacus Darlington, 1934: 107. HOLOTYPE male: Cap Haitien, W.M. Mann (MCZC). One male, two female PARATYPES: Jean Rabel, E.C. & G.M. Leonard (USNM). One female PARATYPE: Port-au-Prince, Aug., G.N. Wolcott (USNM).— Blackwelder 1944: 49.— Erwin and Sims 1984: 440.— Lorenz 1998: 355.— Lorenz 2005: 377.— Perez-Gelabert 2008: 79.
Discoderus cyaneopacus ; Ball 1992: 85.

###### Type locality.

Cap Haitien, Nord Department, Haiti, Hispaniola.

###### Diagnosis.

The larger size, violaceous to bluish metallic luster of the dorsum and piceous legs readily separates the members of this species from those of the three other *Discoderus* species.

###### Descriptive notes.

Data for SBL in Table 1. Habitus as in Fig. 66C. Clypeus with anterior margin markedly concave, basal membrane of labrum visible in most specimens. Anterior margin of labrum with markedly deep V-shaped notch. Antennae and mouthparts rufo-testaceous to dark rufous. Ventral surface and legs piceous. Pronotum and elytra violaceous to bluish, with hints of green; head with less metallic reflection. Head, posteriolateral surface of pronotum and elytra with mesh pattern isodiametric; pronotal disc with mesh pattern slightly transverse, sculpticells about 2× wide as long. Elytral striae impunctate, except the standard setigerous punctures in striae 2, 5 and 7. Males with two terminal setae and females with four terminal setae near the posterior margin on sternum VII.

**Male genitalia.** Fig. 68A–C. Apical portion of phallic median lobe moderately long, narrowly triangular, symmetrically rounded in ventral/dorsal aspects; endophallus without spines or darkened microtrichial fields; without lamina.

**Ovipositor and female reproductive tract.** Fig. 69C. Very similar to that of *D. beauvoisii*. For details, see this topic for *D. beauvoisii*, above.

###### Geographical distribution.

Fig. 71. This species is only known from the Greater Antillean island of Hispaniola.

###### Chorological affinities and relationships.

The range of this species is overlapped by the ranges of *D. beauvoisii* and *D. thoracicus*. The bright metallic luster of the dorsal surface of the body shared by members of *D. cyaneopacus* and *D. beauvoisii* may indicate close relationship between these two species.

###### Material examined.

In addition to type material, we have seen a total of 14 specimens (3 males, 11 females). See Appendix for details.

##### Discoderus thoracicus

Taxon classificationAnimaliaColeopteraCarabidae

(Putzeys)

Figs 66D , 68D–F , 69D , 71

Selenophorus thoracicus Putzeys, 1878a: 59. TYPE MATERIAL: two specimens in Chaudoir-Oberthür Collection (MNHP), in front of following box label: “Haiti/ Mannerh”, LECTOTYPE, first specimen, labelled: [female]; thoracicus Mann S. Dominique D [both labels on green paper, handwritten].— Csiki 1932: 1202.— Darlington 1934: 104, 105.— Blackwelder 1944: 50.— Erwin and Sims 1984: 441.— Lorenz 1998: 356.— Lorenz 2005: 377.— Perez-Gelabert 2008: 80.
Selenophorus excisus Putzeys, 1878a: 59. TYPE MATERIAL: in Chaudoir-Oberthür Collection (MNHP), in front of following box label, 3 specimens (and one empty pin hole) //nigriventris/ Chaud/ Rep. Dominicaine/ Sallé//. To right of series, hand-printed on yellow paper, // excisus//. LECTOTYPE male (first of three specimens, noted above).— Csiki 1932: 1198.— Darlington 1934: 104.— Blackwelder 1944: 50.— Erwin and Sims 1984: 441.— Lorenz 1998: 356.— Lorenz 2005: 377.
Discoderus thoracicus ; Ball 1992: 84, 85.

###### Note about synonymy.

Darlington (1934: 105) established the name *S. excisus* Putzeys, 1878a, as a junior synonym of *S. thoracicus* Putzeys, 1878a.

###### Type area.

Hispaniola, Dominican Republic, as recorded by Putzeys in the original description.

###### Diagnosis.

More slender habitus and shiny head and pronotum with few microlines visible readily separates this species from the similarly colored, but darker, *D. cinctus*. Males have the posteriolateral angles of the pronotum emarginate basally, such that the posteriolateral angle appears obtuse, whereas the females lack the basal emargination and the posteriolateral angles are rounded.

###### Descriptive notes.

Data for SBL in Table 1. Habitus as in Fig. 66D. Clypeus and labrum each with anterior margin moderately concave. Antennae, mouthparts and legs testaceous to slightly darker. Head, pronotum and ventral surface testaceous to rufo-testaceus. Elytra testaceous to rufo-testaceus, with darker median cloud in intervals 2–6. Head and disc of pronotum shiny, at 100× no visible microlines in males, only few microlines visible in females; posteriolateral surface of pronotum with mesh pattern isodiametric; elytral surface with mesh pattern slightly transverse, sculpticells about 1.5× wide as long. Elytral striae impunctate, except the standard setigerous punctures in striae 2, 5 and 7. Males with two terminal setae and females with four terminal setae near the posterior margin on sternum VII.

**Male genitalia.** Fig. 68D–F. Apical portion of phallic median lobe moderately long, narrowly triangular, symmetrically rounded in ventral/dorsal aspects; endophallus without spines or darkened microtrichial fields; without lamina.

**Ovipositor and female reproductive tract.** Fig. 69D. Very similar to that of *D. beauvoisii*. For details, see this topic for *D. beauvoisii*, above.

###### Geographical distribution.

Fig. 71. This species appears to be confined to the Gearter Antillean island of Hispaniola, other than the single specimen labelled simply “Cuba”, to which Philip Darlington attached a label that reads “loc. doubtful”. We believe that this species does not occur on Cuba.

###### Chorological affinities and relationships.

The range of this species is overlapped by the ranges of *D. beauvoisii* and *D. cyaneopacus*. It is geographically isolated from what would seem to be its closest relative, the Cuban *D. cinctus*.

###### Material examined.

In addition to type material, we have seen a total of 222 specimens (113 males, 109 females). See Appendix for details.

## Biogeography

### Island distribution of Selenophorine species in the West Indies

The West Indies comprises thousands of islands, many of which are very small and not inhabited by humans. A total of forty-four species and subspecies of Selenophori are recorded from 76 islands in this paper. The equilibrium theory of island biogeography proposed by MacArthur and Wilson (1967) states that as the island size increases, the number of species living on those islands will increase as well. To apply this theory, we calculated species-area relationships using the least-squares linear regression method. Data for land area were available from published sources for most islands; when not available, the land area was estimated by laying a virtual grid over the image of an island in Google Earth maps. Number of species for each island was determined from the study material on hand.

**Figure 72.**
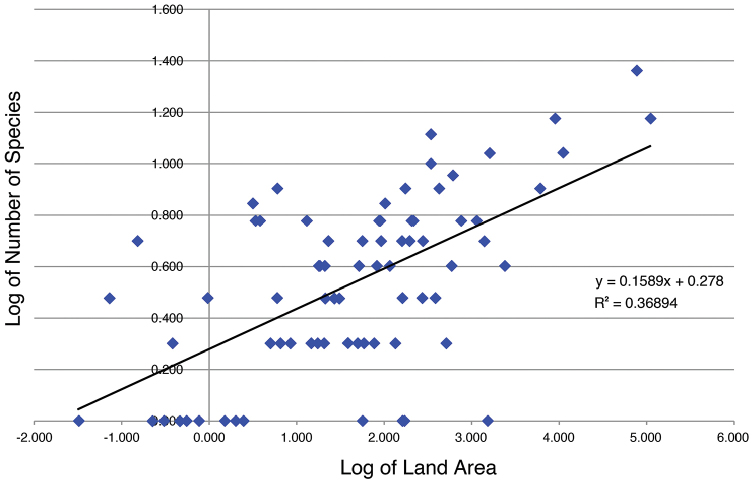
Data analysis of log of number of Selenophori species plotted against log of island area.

Data analysis of log of species number against log of island area (Table 4 run using Excel (Microsoft Office 2010) with the Data Analysis package, produced a least-squares linear regression line with equation y = 0.1589x + 0.278 and R^2^ = 0.36894 (Fig. 72). The *p* value is <0.00001, indicating a highly significant relationship between island size and the number of selenophorine species collected on those islands. However, the R^2^ value of 0.36894 is low indicating that more than half of the plots, some of which can be regarded as outliers, are removed some distance from the regression line. Rather than exclude outlier data as had previously been done by Browne et al (1993), we included all data in our analysis.

Outlier data can be the result of at least two factors. First, islands can be over- or under-collected. When working with museum specimens, one can say with reasonable certainty that the collection effort for each island would not have been the same. For example, eight selenophorine species were recorded on Andros Island with a land area of 5,957 km^2^; eight selenophorine species were also recorded from Mustique with a land area of only 6 km^2^. The largest island with a single recorded selenophorine species is Great Inagua, with a land area of 1,544 km^2^. The smallest island with a single recorded selenophorine species is Marina Cay, with a land area of 0.032 km^2^.

Second, is distance from a source area. Nearly all of the West Indian selenophorine species have functional flight wings, resulting in specimens flying distances for dispersion and therefore being attracted to light traps. Five selenophorine species are recorded from Little Camanoe (Greater Antilles) with a land area of 0.15 km^2^, whereas 7 selenophorine species are recorded from Great Camanoe with a land area of 3.05 km^2^. Little Camanoe is within 230 meters of Great Camanoe. All five of the species recorded from Little Camanoe are recorded from Great Camanoe. The single selenophorine species recorded from Marina Cay, land area of 0.032 km^2^, is also recorded from Great Camanoe, with only 300 meters separating the two islands. Three selenophorine species are recorded from Isla Magueyes, with a land area of only 0.072 km^2^, which is within 70 meters of Puerto Rico, with 15 recorded selenophorine species, and a land area of 8,868 km^2^. The three species found on Isla Magueyes are also recorded from Puerto Rico.

**Table 4.** Data for island area and number of Selenophori species collected on each of those islands (76 in total), plus log of land area and log of number of species which were used to generate Figure 72.

Island	Area (km^2^)	Log Area	# of Species	Log Species
Cuba	109,884	5.040	15	1.176
Hispaniola	76,192	4.881	23	1.362
Jamaica	10,991	4.041	11	1.041
Puerto Rico	8,868	3.948	15	1.176
Andros	5,957	3.775	8	0.903
Isla de Pinos	2,419	3.384	4	0.602
Guadeloupe	1,630	3.212	11	1.041
Great Inagua	1,544	3.189	1	0.000
Grand Bahama	1,373	3.138	5	0.699
Martinique	1,128	3.052	10	1.000
Dominica	751	2.876	6	0.778
St. Lucia	617	2.790	9	0.954
Long	596	2.775	4	0.602
Eleuthera	518	2.714	2	0.301
Barbados	430	2.633	8	0.903
Cat	389	2.590	3	0.477
St. Vincent	345	2.538	10	1.000
Grenada	344	2.537	13	1.114
Antigua	280	2.447	5	0.699
Mayaguana	280	2.447	5	0.699
Middle Caicos	273.9	2.438	3	0.477
St. Croix	218	2.338	6	0.778
New Providence	207	2.316	6	0.778
Grand Cayman	196	2.292	5	0.699
St. Kitts	176	2.246	8	0.903
Marie-Galante	170.5	2.232	1	0.000
Great Exuma	163	2.212	1	0.000
San Slavador	163	2.212	3	0.477
Barbuda	161	2.207	5	0.699
Vieques	135	2.130	2	0.301
North Caicos	116.4	2.066	4	0.602
Montserrat	103	2.013	7	0.845
Nevis	93	1.968	5	0.699
Anguilla	91	1.958	6	0.778
St. Martin	88	1.944	6	0.778
St. Thomas	83	1.919	4	0.602
Rum Cay	77.2	1.888	2	0.301
North Bimini	59	1.771	2	0.301
Mona	55.82	1.747	1	0.000
Tortola	55.7	1.746	5	0.699
St. John	50.8	1.706	4	0.602
Man-O-War Cay	50	1.700	2	0.301
Anegada	38	1.580	2	0.301
Culebra	30.1	1.479	3	0.477
Little Cayman	26.2	1.418	3	0.477
South Bimini	23	1.362	5	0.699
St. Barthelemy	21	1.322	4	0.602
St. Eustatius	21	1.322	4	0.602
Virgin Gorda	21	1.322	3	0.477
Desirade	20.64	1.315	2	0.301
Buck	18.43	1.266	4	0.602
Bequia	18	1.255	4	0.602
Grand Turk	17.4	1.241	2	0.301
Cayman Brac	14.7	1.167	2	0.301
Saba	13	1.114	6	0.778
Union	8.2	0.929	2	0.301
Les Saintes	6.52	0.814	2	0.301
Mustique	6	0.778	8	0.903
Canouan	5.96	0.775	3	0.477
Navassa	5	0.700	2	0.301
Mayreau	3.8	0.580	6	0.778
Guana	3.4	0.531	6	0.778
Great Camanoe	3.05	0.498	7	0.845
Norman	2.428	0.385	1	0.000
Great Swan	2	0.301	1	0.000
Desecheo	1.52	0.182	1	0.000
Darby	0.96	-0.018	3	0.477
Salt	0.76	-0.119	1	0.000
Redonda	0.548	-0.261	1	0.000
Cayo Norte	0.46	-0.337	1	0.000
Sombrero	0.38	-0.420	2	0.301
Little St. James	0.304	-0.517	1	0.000
Little Tobago ^1^	0.233	-0.652	1	0.000
Little Camanoe	0.15	-0.823	5	0.699
Isla Magueyes	0.072	-1.143	3	0.477
Marina Cay	0.032	-1.495	1	0.000

^1^ British Virgin Islands

### Taxonomic aspects of West Indian biogeography

Over a time span of some 33 million years, the West Indies were invaded and occupied by members of eight selenophorine genera and 10 species groups of *Selenophorus* Dejean (Table 5). Most of these assemblages are believed to represent a single occupation, but some species groups are represented by two or three invaders from the mainland. In Table 6 the species are classified as “Immigrant”, meaning representation both in the islands and on the mainland, or “precinctive”, meaning that they originated in the islands, where they are living now, with the implication that they are descended from an immigrant ancestor. See Ball and Shpeley (2009: 180–182) for a brief account of the geological events relevant to the populating of the West Indies.

To analyze the geographical distributions of the selenophorine taxa, we plot them on the following units: Bahamas, Greater Antilles and Lesser Antilles. To generalize further, we indicate occurrences on major portions of the mainland: “Middle America and Florida”; and “South America”. Species are indicated by the generic name with letter “X” and a number 1 to 7. See Tables 7 and 8.

The genera are distributed as in Table 7. Three genera are known only from the Greater Antilles, two from the Lesser Antilles, one is shared between the Bahamas and Greater Antilles and two are shared between the Greater and Lesser Antilles. Six of these genera are immigrant. We postulate that the two precinctive genera (*Paraulacoryssus* and *Neodiachipteryx*) are very early invaders, their ancestors having reached the proto-Antilles by way of the relatively short-lived Greater Antilles-Aves Ridge (GAARlandia) Iturralde-Vinent and MacPhee (1999). Heinicke, Duellman and Hedges (2007) postulated that the proto-Antilles was not connected with a land bridge to South America.

The remaining West Indian genera (*Stenomorphus*, *Discoderus*, and *Amblygnathus*) and species groups of *Selenophorus* reached the islands by waif dispersal (Lazell 2005: 116) or by way of the Nicaraguan Rise (Graham 2010: 74), an island or island chain, now totally submerged, that extended between the mainland and northern Hispaniola (during Neogene time?). We postulate that the 11 immigrant taxa are relatively young in the islands, no older than the Pleistocene, and the 33 precinctive taxa are older, their origins extending at various times through the Neogene Period. This extended interval gives the time that may be required for the differentiation that occurred among the West Indian genera and species groups of *Selenophorus* (Table 7).

In their treatment of the species of *Stenomorphus*, Ball, Shpeley and Currie (1991: 982) noted the geographical proximity of the two Antillean taxa of *Stenomorphus* (*S. cubanus* in eastern Cuba and *S. californicus manni* in western Hispaniola), suggesting an adelphotaxon relationship, but their structural characters suggest that they are more distantly related and the authors postulated that *S. cubanus* was an earlier arrival in the Antilles (early Tertiary?) and possibly by way of Cuba.

All four species of *Discoderus* occupy Hispaniola, to which *D. cyaneopacus* is confined. *Discoderus beauvoisii* ranges from westernmost Cuba throughout the Greater Antilles, and one Bahaman island. *Discoderus cinctus* and *D. thoracicus* are markedly similar to one another and are allopatric in distribution, with *D. cinctus* on easternmost Cuba and *D. thoracicus* along the northern and southern coasts of Hispaniola. This pair of species is adelphotaxic.

Ball and Maddison (1987: 173, Fig. 70B) recognized that *Amblygnathus puncticollis* was a Greater Antillean precinctive member of the *puncticollis* subgroup that included five mainland species, collectively ranging from Panama northward to California and Arizona in the USA.

The West Indian species groups of *Selenophorus* (*sensu lato*), 10 in number, exhibit a pattern as in Table 8. The island/island assemblages contain each between five and 10 species groups. Five species groups are represented throughout the West Indies as well as on the American mainland. Two groups (*latior* and *nonseriatus*) are represented throughout except for the island of Jamaica. One group (*seriatoporus*) is known only by one species in the Lesser Antilles. One group (*mundus*) is known only from two islands (Hispaniola, two species, and Jamaica, single species) in the Greater Antilles.

In more detail, the *discopunctatus*, *parumpunctatus* and *striatopunctatus* species groups are represented each by a single widespread immigrant species that ranges throughout the Antilles. The *latior*, *hylacis* and *nonseriatus* species groups are each represented by different species in the Greater and Lesser Antilles.

Compared to the Antilles, the Bahamas house relatively few taxa (Table 9), with most of them being shared with Cuba, etc. Two immigrant occupants are *S. palliatus* and *S. opalinus*, both only from a single Bahaman island. This indicates markedly recent arrival in the islands. The Greater Antilles have a few more taxa than the Lesser Antilles.

In summary, both the selenophorine genera and species groups of *Selenophorus* show a similar pattern in the Greater Antilles: decrease in number of taxa from Hispaniola eastward to the Puerto Rico Bank, and to Cuba and the Bahamas. In terms of numbers and kinds, the residents of the Greater and Lesser Antilles are nearly equal. Both are markedly more numerous than those of the Bahamas.

**Table 5.** Minimum number of postulated invasions of the West Indies by the Selenophori.

Genera	
*Paraulacoryssus*	1
*Neodiachipteryx*	1
*Neoaulacoryssus*	1
*Athrostictus*	1
*Amblygnathus*	3
*Stenomorphus*	2
*Discoderus*	1
*Selenophorus*	see below
**Species groups of *Selenophorus* (*sensu lato*)**	
*striatopunctatus*	1
*discopunctatus*	2
*parumpunctatus*	2
*palliatus*	3
*opalinus*	2
*latior*	1
*hylacis*	1
*nonseriatus*	1
*seriatoporus*	1
*mundus*	2
**Total invasions**	26

**Table 6.** West Indian Selenophorine taxa: immigrants and precinctives.

Immigrant	Precinctive
*N. cupripennis*	*P. puertoricensis*
*A. paganus*	*N. cariniger*
*A. g. gilvipes*	*N. davidsoni*
*A. cephalotes*	*S. cubanus*
*S. discopunctatus* sp. group	*S. c. manni*
*S. discopunctatus*	*D. beauvoisi*
*S. yucatanus*	*D. cinctus*
*S. palliatus* sp. group	*D. cyaneopacus*
*S. palliatus*	*D. thoracicus*
*S. parumpunctatus* sp. group	*A. puncticollis*
*S. parumpunctatus*	*S. palliatus* sp. group
*S. striatopunctatus* sp. group	*S. alternans*
*S. striatopunctatus*	*S. pyritosus*
*S. opalinus* sp. group	*S. woodruffi*
*S. opalinus*	*S. parumpunctatus* sp. group
*S. seriatoporus* sp. group	*S. obtusoides*
*S. spinosus*	*S. opalinus* sp. group
**TOTAL SPP/ SUBSP: 11**	*S. fabricii*
	*S. flavilabris flavilabris*
	*S. flavilabris cubanus*
	*S. flavilabris ubancus*
	*Selen. integer*
	*S. propinquus*
	*S. latior* sp. group
	*S. latior*
	*S. barbadensis*
	*S. solitarius*
	*S. hylacis* sp. group
	*S. clypealis*
	*S. dessalinesi*
	*S. parvus*
	*S. subquadratus*
	*S. nonseriatus* sp. group
	*S. nonseriatus*
	*S. irec*
	*S. iviei*
	*S. mundus* sp. group
	*S. mundus*
	*S. paramundus*
	*S. pseudomundus*
	**TOTAL SPP/ SUBSP: 33**

**Table 7.** Geographical distribution of the Selenophorine genera and species in the West Indies, excluding *Selenophorus* (*sensu lato*).

Antillean genera & species	Total Antillean taxa	Middle America & Florida	West Indies			South America
			Bahamas	Greater Antilles	Lesser Antilles	
*Paraulacoryssus*	1			X1		
*Neodiachipteryx*	2			X1, X2		
Neoaulacoryssus	1				X1	X
*Athrostictus*	1	X			X1	X
*Stenomorphus*	2	X		X2		X
*Discoderus*	4	X	X1	X1, X2, X3, X4		
*Amblygnathus*	3	X		X1	X2, X3	X
Total W..Ind. Genera/ (spp.)	7 (14)	4	1 (1)	5 (9)	3 (4)	4

**Table:** Symbols for species of West Indian selenophorine genera excluding *Selenophorus* (*sensu lato*) used in Table 7.

*Paraulacoryssus*		*Discoderus*	
*P. puertoricensis*	X1	*D. beauvoisii*	X1
*Neodiachipteryx*		*D. cinctus*	X2
*N. cariniger*	X1	*D. cyaneopacus*	X3
*N. davidsoni*	X2	*D. thoracicus*	X4
*Neoaulacorryssus*		*Amblygnathus*	
*N. cupripennis*	X1	*A. puncticollis*	X1
*Athrostictus*		*A. g. gilvipes*	X2
*A. paganus*	X1	*A. cephalotes*	X3
*Stenomorphus*			
*S. cubanus*	X1		
*S. calif. manni*	X2		

**Table 8.** Geographical distribution of the species groups and species of *Selenophorus* (*sensu lato*) in the West Indies.

*Seleophorus* species groups & species	Total *Selenophorus* (*s. l.*) taxa	Middle America & Florida	West Indies			South America
			Bahamas	Greater Antilles	Lesser Antilles	
*discopunctatus*	2	X	X1	X1	X1, X2	X
*palliatus*	4	X	X1, X2, X3	X1, X3	X4	X
*parumpunctatus*	2	X	X1	X1, X2	X1	X
*striatopunctatus*	1	X	X1	X1	X1	X
*opalinus*	7	X	X1, X3, X4, X6, X7	X1, X2, X3, X4, X5, X7	X2, X5, X7	X
*latior*	3			X1, X3	X1, X2	
*hylacis*	4	X		X1, X2, X3	X1, X3, X4	X
*nonseriatus*	3	X		X1	X1, X2, X3	X
*seriatoporus*	1	X			X1	X
*mundus*	3			X1, X2, X3		
Total Antillean taxa (spp.)	11 (30)	8	6 (11)	8 (20)	9 (17)	8

**Table:** Symbols for species of *Selenophorus* used in Table 8.

*Selen. discopunctatus* species group		*Selen. latior* species group	
*S. discopunctatus*	X1	*S. barbadensis*	X1
*S. yucatanus*	X2	*S. latior*	X2
*Selen. palliatus* species group		*S. solatarius*	X3
*S. alternans*	X1	*Selen. hylacis* species group	
*S. palliatus*	X2	*S. clypealis*	X1
*S. pyritosus*	X3	*S. desslalinesi*	X2
*S. woodruffi*	X4	*S. parvus*	X3
*Selen. parumpunctatus* species group		*S. subquadratus*	X4
*S. parumpunctatus*	X1	*Selen. nonseriatus* species group	
*S. obtusoides*	X2	*S. irec*	X1
*S. striatopunctatus* species group		*S. iviei*	X2
S. *striatopunctatus*	X1	*S. nonseriatus*	X3
*Selen. opalinus* species group		*Selen. seriatoporus* species group	
*S. fabricii*	X1	*S. spinosus*	X1
*S. f. flavilabris*	X2	*Selen. mundus* species group	
*S. f. cubanus*	X3	*S. mundus*	X1
*S. f. ubancus*	X4	*S. paramundus*	X2
*S. integer*	X5	*S. pseudomundus*	X3
*S. opalinus*	X6		
*S. propinquus*	X7		

**Table 9.** Distribution of the species and subspecies of Selenophori group in the West Indies.

	Middle America & Florida	West Indies								South America	No. Isl.
		Bah.	Greater					Lesser			
			Cuba	Cay.	Jam.	Hisp.	P. R.	Leew.	Wind.		
*S. pyritosus* ^1^	X	X	X	X	X	X					11
*S. palliatus*	X	X									4
*S. discopunctatus*	X	X	X	X	X	X	X	X	X	X	56
*S. yucatanus*	X							X	X		4
*S. parumpunctatus*	X	X	X	X	X	X	X	X	X		53
*S. striatopunctatus*	X	X	X	X	X	X	X	X	X		17
*S. fabricii*	X	X	X	X	X	X	X				21
*S. opalinus*	X	X									1
*D. beauvoisii* ^2^		X	X		X	X	X				14
*S. alternans* ^1^		X	X			X	X	X	X		29
*S. flavi. cubanus*		X	X								2
*S. flavi. ubancus*		X			X	X					5
*S. propinquus*		X			X	X	X	X	X		25
*S. cubanus* ^3^			X								1
*D. cinctus* ^2^			X								1
*A. puncticollis* ^4^			X		X	X	X				4
*S. obtusoides* ^1^			X								1
*S. solitarius*			X								1
*S. subquadratus*			X			X	X	X	X		9
*S. nonseriatus*			X		X	X		X	X		6
*S. paramundus*					X						1
*N. davidsoni* ^5^						X					1
*N. cariniger*						X					1
*S. calif. manni* ^3^						X					1
*D. cyaneopacus* ^2^						X					1
*D. thoracicus*						X					1
*S. pseudomundus* ^1^						X					1
*S. mundus*						X					1
*S. latior*						X	X	X	X		12
*S. dessalinesi*						X					1
*S. clypealis*						X	X				3
*S. integer*						X	X	X	X		25
*S. parvus*							X	X	X		10
*S. flavi. flavilabris*							X	X			3
*P. puertoricensis* ^6^							X				1
*A. paganus* ^7^								X	X	X	18
*A. cephalotes* ^4^								X		X	1
*S. woodruffi* ^1^								X	X		2
*S. iviei*								X	X		5
*S. irec*								X			1
*N. cupripennis* ^8^									X	X	3
*A. g. gilvipes* ^4^									X	X	2
*S. spinosus* ^1^									X	X	1
*S. barbadensis*									X		2
*S. dubius*											?
TOTAL	8	12	15	5	11	23	15	17	18	6	

^1^ *Selenophorus* ^2^ *Discoderus* ^3^ *Stenomorphus* ^4^ *Amblygnathus* ^5^ *Neodiachypteryx* ^6^ *Paraulacoryssus* ^7^ *Athrostictus* ^8^ *Neoaulacoryssus*

## Supplementary Material

XML Treatment for Selenophori

XML Treatment for Neoaulacoryssus

XML Treatment for Neoaulacoryssus cupripennis

XML Treatment for Paraulacoryssus

XML Treatment for Paraulacoryssus puertoricensis

XML Treatment for Athrostictus

XML Treatment for Athrostictus paganus

XML Treatment for Amblygnathus

XML Treatment for cephalotes

XML Treatment for Amblygnathus cephalotes

XML Treatment for iripennis

XML Treatment for Amblygnathus puncticollis

XML Treatment for suturalis

XML Treatment for Amblygnathus gilvipes gilvipes

XML Treatment for Neodiachipteryx

XML Treatment for Neodiachipteryx cariniger

XML Treatment for Neodiachipteryx davidsoni

XML Treatment for Selenophorus

XML Treatment for Subgenus Celiamorphus

XML Treatment for Selenophorus discopunctatus

XML Treatment for Selenophorus discopunctatus

XML Treatment for Selenophorus yucatanus

XML Treatment for Selenophorus latior

XML Treatment for Selenophorus barbadensis

XML Treatment for Selenophorus latior

XML Treatment for Selenophorus solitarius

XML Treatment for Selenophorus seriatoporus

XML Treatment for Selenophorus spinosus

XML Treatment for Subgenus Selenophorus

XML Treatment for Selenophorus hylacis

XML Treatment for Selenophorus clypealis

XML Treatment for Selenophorus dessalinesi

XML Treatment for Selenophorus dubius

XML Treatment for Selenophorus parvus

XML Treatment for Selenophorus subquadratus

XML Treatment for Selenophorus mundus

XML Treatment for Selenophorus mundus

XML Treatment for Selenophorus paramundus

XML Treatment for Selenophorus pseudomundus

XML Treatment for Selenophorus nonseriatus

XML Treatment for Selenophorus irec

XML Treatment for Selenophorus iviei

XML Treatment for Selenophorus nonseriatus

XML Treatment for Selenophorus opalinus

XML Treatment for Selenophorus fabricii

XML Treatment for Selenophorus flavilabris

XML Treatment for Selenophorus flavilabris cubanus

XML Treatment for Selenophorus flavilabris flavilabris

XML Treatment for Selenophorus flavilabris ubancus

XML Treatment for Selenophorus integer

XML Treatment for Selenophorus opalinus

XML Treatment for Selenophorus propinquus

XML Treatment for Selenophorus palliatus

XML Treatment for Selenophorus alternans

XML Treatment for Selenophorus palliatus

XML Treatment for Selenophorus pyritosus

XML Treatment for Selenophorus woodruffi

XML Treatment for Selenophorus parumpunctatus

XML Treatment for Selenophorus obtusoides

XML Treatment for Selenophorus parumpunctatus

XML Treatment for Selenophorus striatopunctatus

XML Treatment for Selenophorus striatopunctatus

XML Treatment for Stenomorphus

XML Treatment for Stenomorphus californicus

XML Treatment for Stenomorphus californicus manni

XML Treatment for Stenomorphus cubanus

XML Treatment for Discoderus

XML Treatment for Discoderus beauvoisii

XML Treatment for Discoderus cinctus

XML Treatment for Discoderus cyaneopacus

XML Treatment for Discoderus thoracicus

## Appendix (label data)

This section contains label data for specimens other than type material that were examined for this study. All abbreviations of collectors’ proper names are followed by a period whether or not the period(s) were present on the locality label. The island of St. Croix is included in the Greater Antilles island grouping (Peck et al. 2014).

### *Neoaulacoryssus cupripennis* (Gory)

“WEST INDIES”, 1920 (BMNH). **LESSER ANTILLES. Grenada. St. George Parish.** Mount Gay Estuary, H.H. Smith (BMNH). **Mustique.** 1920, H.H. Smith (BMNH). **St. Lucia.** Anse La Raye, Anse Galet, 1 km SSW Anse La Raye, 50 m, VI.21-30.1991, J.E. Rawlins, S.A. Thompson (CMNH).

### *Paraulacoryssus puertoricensis* (Mutchler)

**GREATER ANTILLES. Puerto Rico.** Aibonito, II.20.1932, S.T. Danforth (MCZC). Ensenada, VI.14-19.1915 (AMNH). L. Guanica, V.31.1938, Darlington (MCZC). Lajas, XII.13.1935, H.L. Dozier (USNM).

### *Athrostictus paganus* (Dejean)

**GREATER ANTILLES. St. Croix.** XI.26.1925 (AMNH). **LESSER ANTILLES. Antigua.** stn. # 282, VIII.28.1936, Chapin, Blackwelder (USNM). Christian Valley, bl trap, VII.17.1991, FAO Insect Survey (CMNC). **Barbados.** V.16, D. & L. Stoner (AMNH); V.16-18, D. Stoner (AMNH); VI, D. & L. Stoner (AMNH); IX.1900, Uyttenboogaart (ZMAN). Marine Hotel, at light, IX.14.1918, H. Morrison (USNM). **St. Michael.** Cavehill, uv light, VIII.1972, M.M. Alam (UASM). **Barbuda.** Codrington, 0-20 m, VII.1976, N.L.H. Krauss (AMNH). **Canouan.** IV.1937, S.T. Danforth (MCZC). **Grenada. St. George Parish.** Mount Gay Estuary (Leeward Side), H.H. Smith (BMNH). **Guadeloupe.** Pte Grande Vigie, XII.12.1971, Chalumeau (IREC). **Marie Galante.** Pte Pisiou, II.5.1978, Chalumeau (IREC). **Martinique.** H. Stehle (USNM). Anse a 21Ave, X.27.1981, Roguet, (IREC). Caravelle, VIII.5.1973, Camb. (IREC). Caravalle peninsula, uv light trap, IX.21.2014, N. Moulin, J. Braud (JMLC). Fort de France, 180 m, VI.1944, H. Stehle (USNM). **MonTserrat.** Cassava Ghaut, Beattie House, 16°45.91'N, 62°12.95'W, 192.6 m, light trap, VI.14-21.2002, A. Krakower (WIBF). Delvine Village, V.28.1982, Chalumeau (IREC). stn. #270, VII.24.1936, Blackwelder (USNM). stn. #271, VII.25.1936, Blackwelder (USNM). Woodlands, Riverside House, 16°45.99'N, 62°13.344'W, uv light, VII.31.2005, I.A. Foley (WIBF). **Mustique.** 1920, H.H. Smith (BMNH). **NEVIS.** VI.29.1935, S.T. Danforth (MCZC). H.A. Beatty (MCZC), (WIBF), (USNM). **St. Eustatius.** I.23.1937, S.T. Danforth (UASM). **St. Kitts.** VI.193, S.T. Danforth (MCZC). **St. Lucia.** Castries, uv trap, VI.19.1977, R.E. Woodruff (FSCA), (UASM). Escap Comm., 13.8324°N 60.8986°W, merc vapor light, 30 m, V.1.2009, I.A. Foley (WIBF). Escap Comm-Fond Bay, 13.83242°N 60.89864°W, V.14-16.2009, 1-46 m, R.C. Winton, E.A. Ivie (WIBF). **St. Martin.** Great Bay, XI.21.1927, S.T. Danforth (MCZC). Marigot, V.26.1973, Chalumeau (IREC). **St. Vincent. St. George Parish.** Kingstown, at light, “10-2-31”, M. Kisliuk, C.E. Cooley (USNM). **not located.** South End: VII.5.1908, M. Cameron (BMNH); 1920, H.H. Smith (BMNH); H.H. Smith (BMNH). **Trinidad & Tobago.** VIII, Busk (USNM). Tobago, S of airport, stn. #582, I.17.1955, P. Wagenaar Hummelinck (ZMAN).

### *Amblygnathus puncticollis* (Putzeys)

**GREATER ANTILLES. CUBA. Camaguey.** Baragua, at light, VI.11.1932, Christenson (UASM). **Habana.** El Lucero, VI.1945 (MNHC). Santiago de las Vegas, VI.6.1960, M. Barro (MNHC). **Pinar del Rio.** San Vicente, VI.24.1957 (FSCA). **HISPANIOLA: DOMINICAN REPUBLIC. Dajabon.** 9 km S Loma de Cabrera, 620 m, 19°21'N, 71°37'W, disturbed pastures in mesic woodland, VII.12.1992, C. Young, S. Thompson et al (CMNH). **Independcia.** 4 km S Los Pinos, Loma de Vientos, 455 m, 18°35'N, 71°46'W, semiarid deciduous forest with pastures, VII.23.1992, C. Young, S. Thompson et al (CMNH). **La Altagracia.** 2 km N Bayahibe, 10 m, 18°23'N, 6851'W, Dry seasonal forest on limestone, VII.3.1992, C. Young, S. Thompson et al (CMNH). Nisibon Papagallo, Lake area, blacklight trap, VI.24.1998, R.E. Woodruff (FSCA). **La Vega.** La Cienega de Manabao, Park Hdqt., 914 m., blacklight, VII.3-5.1999, R.E. Woodruff (FSCA). **Pedernales.** km 24 N Cabo Rojo, 914 m., blacklight trap, VI.23.1999, Woodruff & Baranowski (FSCA). along Rio Mulito, 13 km N Pedernales, 230 m, 18°09'N, 71°46'W, riparian woodland, VII.17.1992, C. Young, S. Thompson et al (CMNH). Las Mercedes, 21 km N Cabo Rojo, 490 m, VII.10.1987, J. Rawlins, R. Davidson (CMNH). **Puerto Plata.** Puerto Plata, VII.20.1937, Clench (MCZC). **HISPANIOLA: HAITI. Sud.** Etang Lachaux, SW peninsula, under 304.8 m, X.26-27.1923, Darlington (UASM). **JAMAICA. Manchester.** Mandeville, uv light trap, J.H. Frank (UASM): V.22-26.1970; VI.18-20.1970. Mandeville, X.3-5.1969, J.H. Frank (UASM); uv trap; uv light. **St. Andrew Parish.** Swallowfield, X.1950, C.B. Lewis (UASM). **St. Catherine Parish.** Hellshire Hills, Spearwood Valley, uv light trap, VIII.1-IX.1.1970, J.H. Frank (UASM). **St. Thomas.** Golden Cove (Grove), uv trap, IX.9-10.1969, J.H. Frank (UASM). **PUERTO RICO.** Hacienda Paraiso, Real Anon, black light, “12-10-04”, O.H. Garrido, A.P. Asso (WIBF). Mercedita, at lights, V.12.1972, J. Micheli (JMPR). Ponce, at lights, VIII.1.1971, J. Micheli (JMPR).

### *Amblygnathus gilvipes gilvipes* Ball & Maddison

**LESSER ANTILLES. MARTINIQUE.** Caravelle peninsula, uv light trap, IX.167.2014, N. Moulin, J. Braud (JMLC). **ST. VINCENT. St. Andrew Parish.** E. Layou, Emerald Valley Hotel, 13°12.0'N, 61°14.8'W, forest edge, uv, 20 m, 06-123, VIII.27-29.2006, S. & J. Peck (CMNC).

### *Neodiachipteryx cariniger* (Putzeys)

**GREATER ANTILLES. HISPANIOLA. HAITI. Ouest.** Furcy, W.M. Mann (MCZC); W.M. Mann (UASM). La Visite & vic., LaSelle Range, 1524-2133.6 m, IX.16-23.1934, Darlington (MCZC), (UASM).

### *Selenophorus discopunctatus* Dejean

**UNITED STATES. Florida.** Collier County. Chokoloskee (type locality of *S. chokoloskei* Leng). Pinellas County. Dunedin (record from Darlington, 1935). St. Lucie County. Lakewood Park, uv light trap, VIII.25.1972, J.H. Frank (UASM). **BAHAMAS. Andros Island.** 8.8 km N “T” junction, 8 km E old lumber road, hidden coppice, coast, coppice sweeping, VII.30.1987, J. Browne (CMNC). Stafford Crk., Doc Woodside’s Place, high coast coppice, black light, VII.26.1987, J. Browne (CMNC). **Cat Island.** WJ Clench (MCZC): VII.17.1935; VII.23.1935. **Darby Island.** I.18.1953, E.B. Hayden, L. Giovannoli (AMNH). **Grand Bahama Island.** 8 Mile Rock, IV.23.1936 (MCZC). **Long Island.** Clarence Town, III.13.1953, E.B. Hayden, L. Giovannoli (AMNH). **New Providence Island.** Nassau, UV trap, VI.24.1972, F.D. Bennett (FSCA). **San Salvador.** Gerace Research Centre, II.20.2004, W.E. Steiner, J.M. Swearingen (USNM). Gerace Research Centre, scrub forest edge, at open catchment, uv light, W.E. Steiner, J.M. Swearingen (USNM): VI.19.2003; VI.20.2003; VI.22.2003. **TURKS & CAICOS. Grand Turk Island.** Waterloo, C.B. Lewis (IJSM): IV.22.1954; IV.26.1954. **Middle Caicos.** Zambarra, uv, XII.4-11.1993, B.M. Riggs (FSCA). **North Caicos.** uv light, VII.20.1993, A. Swann (FSCA). **GREATER ANTILLES. CAYMANS. Grand Cayman Island.** uv light, P. Fitzgerald (FSCA): VII.9.1987; VI.14.1991. Boatswain Pointe, XII.20.1975, E.J. Gerberg (FSCA). Mastic Trailhead S, bl trap, V.21.2009, Thomas, Turnbow & Ball (FSCA). 3.2 km N Georgetown, flooded frehwater pond, V.26.1975, P.J. Spangler (USNM). 4.8 km N Georgetown, V.26.1975, A.B. Gurney (USNM). **CUBA.** Palmer & Riley (USNM). **Camaguey.** Baragua, at light, L.C. Scaramuzza: IV.24.1928 (USNM); VII. 27.1929 (MCZC); VII. 30.1929 (MCZC). Baragua, at light, Christenson (USNM): V.24.1932; V.29.1932; VI.3.1932; VI.11.1932. Baragua, grasses, XII.13.1929, L.D. Christenson (MCZC). Camaguey, IV.21-V.5.1933, H.J. MacGillavry (ZMAN). **Cienfuegos.** Cayamas, EA Schwarz (USNM): I.1; II.6; III.12; V.30; VI.11; XII.29. San Antonio, IV.1905, G. Dimmock (USNM). Soledad, Darlington (MCZC): X.15.1926; X.22.1926; XI.11.1926; VI.1929; VIII.2-12.1934; IV.1936; V.1936; VIII.1936. Soledad (MCZC): B.B. Leavitt; IX.2.1932, B.B. Leavitt; V-VI.1939, Parsons. Soledad, big flood, X.21.1926, Darlington (MCZC). Soledad, 21.12682 -80.33289, mv light, 71 m, V.21.2013, 2013-029X, R.S. Anderson (CMNC). Trinidad Mts., 182.9-609.6 m, VI.1929, Darlington (MCZC). **Havana.** Almendares, V.24.1932, M. Barro (IZAC). Guanajay, IV.25, Palmer & Riley (USNM). Guantanamo Bay, Center Bargo, uv, S. Calhoun (FSCA): VIII.3.1972; IX.7.1972. Guantanamo U.S. Navy Base, uv, IX.3.1964, T.S. Josey (FSCA). Guantanamo Bay Navy Base, Caravella Point, uv, S. Calhoun (FSCA): IX.6.1972; IX.11.1972. Guantanamo Bay Navy Base, Kittery Housing Area, uv, V.15-18.1972, S. Calhoun (FSCA). Havana, Baker (AMNH), (CMNH), (MCZC), (UASM), (USNM). Jibacoa, Litoral Costa Norte, V.1962 (MNHC). Santa Maria del Mar, I.7.1967, R. Bielawski, A. Riedel (IZWP). Stgo.-Vegas, IV.1905, G. Dimmock (USNM). **Holguin.** Calabezas, Aguayo, I.1932 (MCZC). **Isla de la Juventud.** Nueva Gerona, G. Link (CMNH). **Matanzas.** Matanzas, X.3.1973, E. & Z. Meszaros (HNHM). Playa Larga, Cienaga Zapate, luz, P. & A. (PVRC): VI.18.1996; VI.20.1996; IV.1997. **Pinar del Rio.** Cabanas, Palmer & Riley (USNM): V.21; VI.2. Estacion El Taburete, Sierra del Rosario, X.1998, P. Valdez (PVRC). Pinar del Rio, V.16-29.1933, H.J. MacGillavry (ZMAN). S. del Rangel, V.1934, M. Barro (MCZC). 7 km N Vinales, IX.16-22.1913 (AMNH). **Sancti Spiritus.** Topes de Collantea, VII.17.1956, C. & P. Vaurie (MCZC). **Santiago de Cuba.** Aguadores, VI.6.1936, Darlington (MCZC). Sierra Maestro, Ocujal del Turquino, II.9.1967, R. Bielawski, A. Riedel (IZWP). coast below Pico Turquino, VI.26-30.1936, Darlington (MCZC). **Villa Clara.** north end of Lago del Hanabanilla, VII.1.1990, J. Rawlins, S. Thompson (CMNH). **not located.** Verrasco, boundary of oak-fir forest and plains, VI.5.1933, H.J. MacGillavry (ZMAN). **HISPANIOLA: DOMINICAN REPUBLIC. Azua.** 8 km NE Padre Los Casas, Rio Las Cuevas, 580 m, 18°46'N, 70°53'W, riparian growth in arid thornscrub, X.3-4.1991, C.Young, S. Thompson et al (CMNH). 8 km NE Padre Los Casas, Rio Las Cuevas, 580 m, 18°46'N, 70°53'W, VIII.7.1990, J. Rawlins, S. Thompson (CMNH). **Barahona.** Barahona, X.1938, Darlington (MCZC). vic. Filipinas, 518.2 m, V.5-6.1985, E. Giesbert (FSCA).**Dajabon.** 9 km S Loma de Cabrera, 620 m, 19°21'N, 71°37'W, disturbed pastures in mesic woodland, VII.12.1992, C. Young, S. Thompson et al (CMNH). Rio Massacre Balneario Don Miguel, 7 km SW Dajabon, 40 m, V.26.1973, D. & M. Davis (USNM). **Distrito Nacional.** VIII.12.1967, L.H. Rolston (TAMU). Cachon de la Rubia, nr Central Ozama, VI.10.1969, Flint & Gomez (USNM). Santo Domingo, VIII. 5.1967, L.H. Rolston (TAMU). Sosua, Clench (MCZC). **El Seibo.** Miches, UV trap, VI.9.1976, R.E. Woodruff (FSCA). **Espaillat.** Moca, 1926, Ciferri (MCSN). **Independcia.** S Lago Enriquillo, 18°24'N, 71°42'W, uv light, VII.12.1993, D.S. Sikes, R.P. Rosenfeld (WIBF). 3 km up road from La Descubierta to Los Pinos, blacklighting, VII.15.2005, S.W. Lingafelter (USNM). 4 km S Los Pinos, Loma de Vientos, 455 m, 18°35'N, 71°46'W, semiarid deciduous forest with pastures, C. Young, S. Thompson et al (CMNH): VII.21.1992; VII.23.1992. **La Altagracia.** Nisibon, uv, IX.24.1985, Stange, Woodruff & House (FSCA). Nisibon Finca Papagallo, blacklight, VI.16-19.1999, R.E. Woodruff & R.M. Baranowski (FSCA). vic. Oyo Clara, 18°33'48"N, 68°26'50"W, uv light, VII.24-VIII.5.2002, K.W. Will, C. Chaboo (EMEC). P.N. del Este, Boca de Yuma, 18°21.904'N, 68°37.094'W, at light, 2 m, VIII.5.1999, M.A. Ivie, K.A. Guerrero (WIBF). Punta Cana Resort, 18°30'16"N, 68°22'37"W, at light, VII.24-VIII.5.2002, K.W. Will, C. Chaboo (EMEC). **La Romana.** La Romana, uv, IX.17.1976, R.E. Woodruff (FSCA). 8 km W Romana, under rocks, XI.16.1984, P. Spangler, R. Faitoute (USNM). **Maria Trinidad Sanchez.** Nagua, IV.15.1977, R.S. Rominger (UCDC). **Monte Cristi.** 5 km NNE Bontoncillo, 50 mm 19°45'N, 71°42'W, arid thornscrub, XI.29-30.1992, Davidson, Klingler (CMNH). Monte Cristi, VI.21.1967, L.H. Rolston (TAMU). 3 km N Villa Elisa, uv, X.1.1985, Woodruff & Stange (FSCA). 5 km N Villa Elisa, V.10-18.1985, E. Giesbert (FSCA). **Pedernales.** Cabo Rojo, 35 m 18°02'N, 71°39'W, VII.19.1990, Rawlins, Young Thompson (CMNH). Cabo Rojo, at light, 0-10 m, IX.10.1988, M. Ivie, Philips, Johnson (WIBF). Cabo Rojo, in pool & at lights, M. Ivie, Philips, Johnson (WIBF): VIII.18-23.1988; VIII.28-29.1988. Cabo Rojo, Alcoa Headquarters, blacklight trap, VI.20-24.1999, Woodruff & Baranowski (FSCA). 10 km E Cabo Rojo, under dry cow dung in desert, VI.23.1999, R.E. Woodruff (FSCA). 14 km N Cabo Rojo, 150 m., thorn scrub trop. dry forest, VIII.19.1988, M. Ivie, Philips, Johnson (WIBF). 14.5 km N Cabo Rojo, 165 m, 18°03'N, 71°39'W, arid thornscrub, IX.26-271991, C. Young, S. Thompson et al (CMNH). 17 km N Cabo Rojo, 255 m, 18°04'N, 71°39'W, dry deciduous forest, XI.21.1991, C. Young, S. Thompson et al (CMNH). 21 km N Cabo Rojo, uv, VI.19.1976, R.E. Woodruff (FSCA). 27 km N Cabo Rojo, uv trap, VI.19.1976, R.E. Woodruff (UASM). **Peravia.** 12.4 km E Rio Ocoa, VII.3.1992, M.A. & R.O. Ivie (WIBF). **Puerto Plata.** 8 km W Bani, uv, V.25.1985, Woodruff & Stange (FSCA). Los Hidalgos, VI.4-5.1969, Flint & Gomez (USNM). Puerto Plata (MCZC): VII.20.1937, Clench; VIII.29-IX.2.1938, Darlington. **San Cristobal.** San Cristobal, VI.8-9.1969, Flint & Gomez (USNM). Villa Altagracia, VII.1938, Darlington (MCZC). **San Juan.** 11 km WNW Hato Nuevo, 1 km SE Ingenito, Presa de Sabaneta, 19°02'N, 71°18'W, near shoreline, Rawlins, Davidson, Onore (CMNH). Rio Mijo, uv, V.20.1985, Woodruff & Stange (FSCA). **Santo Domingo.** Ca Duarte, K. 29, VII.3.1978, Chalumeau & Abud (IREC). Haina, 1920, G.N. Wolcott (AMNH). **HISPANIOLA: HAITI. Artibonite.** Ennery, nr 304.8 m, IX.6-11.1934, Darlington (MCZM). **Centre.** Poste Terre Rouge, 61 m, X.5.1934, Darlington (MCZC). **Ouest.** Camp Perrin, South Peninsula, II.27.1983, M.K. Langworthy (UASM). Mannville, XI.16-17.1934, Darlington (MCZC). 5 km S Montrouis, under dry grass, V.2.1977, J.H. Frank (UASM). Port-au-Prince, III.1925, G.N. Wolcott (AMNH), (MCZC), (USNM). Port-au-Prince, Thor (a suburb), X.10-12.1970, J.E. Porter (FSCA): UV trap; P. Daniels res., uv. **JAMAICA. Clarendon Parish.** Milk River Bath, XI.19.1968, R.E. Woodruff (FSCA). Milk River Bath, uv, XI.19.1968, R.E. Woodruff (FSCA). Portland Ridge, N side, VII. 23.1958, T.H. Farr (IJSM). Portland Ridge, PWD Fishing Club, uv, VIII.20.1969, Woodruff (FSCA). **Manchester Parish.** Mandeville, uv trap J.H. Frank (UASM): VIII.22-25.1969; X.3-5.1969. Mandeville, uv light trap J.H. Frank (UASM): V.22-24.1970; VI.4-5.1970; VIII.14-16.1970. nr Oxford Caves, VII.19.1970, J. Farradane (BMNH). **St. Andrew Parish. **Beverley Hills, XI.3.1953, R.P. Bengry (IJSM). Constant Spring, IV.2-11.1931 (AMNH). Crossroads (IJSM): VI.1941, C.B. Lewis; I.8.1950, R.P. Bengry. Guava Ridge, VII.5.1950, R.P. Bengry (IJSM). Half-Way Tree, R.P. Bengry (IJSM): IX.3.1950; IX.10.1951. Half-Way Tree, I.28.1937, Chapin & Blackwelder (USNM). Half-Way Tree, at light, II.6-12.1937, Chapin & Blackwelder (USNM). Kingston, Darlington (MCZC): VIII.27.1934; VIII.28.1934. Kingston: VII.1.1966, H. Howden (CNCI); I.31, Chapin & Blackwelder (USNM). Kingston, Mona Hotel, uv, X.16.1971, R.M. Baranowski (FSCA). Kingston Botanic Gardens, under log, III.15.1969, J.H. Frank (UASM). Liguanea Plain, XI-XII.1911, C.T. Brues (MCZC). Liguanea, nr Kingston, VI.10.1931, M. Kisliuk (USNM). Mona, IX.23.1950, M. Parry (IJSM). Swallowfield, C.B. Lewis (IJSM): VI.9.1951; X.27.1951. Swallowfield, X.12.1951, R.P. Bengry (IJSM). Upper Mountain View, C.B. Lewis (IJSM): VI.6-10.1942; III.9.1947; IX.13.1947; XI.4.1947; VI.6.1948. Upper Mountain View, G.B. Thompson (IJSM): X.1.1946, X.8.1946; X.16.1946; X.16.1946; X.20.1946. St. Elizabeth. Black River, at licght, VI.7.1969, J.H. Frank (UASM). **St. James Parish.** Greenwood, II.12.1947, C.B. Lewis (IJSM). Montego Bay, X.25.1950, H.B. Southby (IJSM). **St. Mary Parish.** Highgate, uv, VI.1.1970, E.G. Farnsworth (FSCA), (UASM). Port Maria, VII. 12.1953, R.P. Bengry (IJSM). **Trelawny Parish.** Duncans, Howden & Becker (CNCI): VIII. 4.1966; VIII. 6.1966; VIII.14.1966; VIII.15.1966; VIII.19.1966; VIII.21.1966. 14.5 km W Falmouth, VIII.5.1967, L. & C.W. O’Brien. Vale Royal, X.1-3.1969, R. Arscott (UASM). **NAVASSA ISLAND.** near lighthouse, 18°23.82'N, 75°00.74'W, 80 m, VII.30.1998, W.E. Steiner, J.M. Swearingen (USNM). bluff of SW rim, 18°23.75'N, 75°00.65'W, VII.30.1998, W.E. Steiner, J.M. Swearingen (USNM). **PUERTO RICO.** Aguirre, in soil around cane-stool, V.1.1925, H.E. Box (USNM). Aibonito, S.T. Danforth (MCZC): II.20.1932; II.21.1932. Bayamon, at light, XII.26.1932, (USNM). Bosque de Guanica, 17°58'50"N, 66°52'37"W, 15W BL, sheet in dry coastal forest at Cobanus Rd., VI.9.2005, R. Brown, S. Lee (MEMU), (UASM). Bosque de Guanica, 17°58'31"N, 66°52'45"W, 15W BL, box trap with ammonia, dry coastal forest at Ojo de Agua, VI.9.2005, R. Brown, S. Lee (MEMU). Coamo Springs (AMNH): VI.5-7.1915; VIII.25.1919. Rio Grande, El Verde Stn., 3.1 km WNW Pico El Yunque, Sierra de Luquillo, 355 m, VI.3-6.1996, Young et al (CMNH). El Yunque Stn., Luquillo Forest, VII.10-16.1969, H. & A. Howden (UASM). Ensenada, VI.14-19.1915A (MNHM). Ensenada, under rock, nr sea level, II.24.1924, J. Micheli (USNM). Guanica, rd #116, under rocks, xerophytic area, VIII.17.1974, J. Micheli (USNM). Guanica Forest, H. & A. Howden (UASM): VII.25-26.1969; VII.27.1969; VII.29.1969. Guanica Forest Res., beating at beach, IX.24.1987, M.A. Ivie (WIBF). Isabela Expt. Stn., UV trap, VII.2.1972, R.E. Woodruff, A.E. Agostini (FSCA). L. Guanica, V.31.1938, Darlington (MCZC). L. Tortuguero, VIII.2.1962, O. & R. Flint, Matthews (USNM). La Parguera, H. & A. Howden (UASM): VII.28.1969; VII.30.1969. La Plata, X.15.1927, S.T. Danforth (MCZC). Luquillo National Forest, El Yunque Rd., UV trap, VII.5.1977, R.E. Woodruff (FSCA). Mayaquez: II.1968, Lenczy (CUIC); VII.3.1975, S.J. Ramos (JMPR). Mayaquez, night sweep, VIII.8.1961, Flint & Spangler (USNM). Mercedita, V.3.1975, J. Micheli (JMPR). Mercedita, at lights after rain, X.12.1976, J. Micheli (UASM). Ponce, VII.1975, J. Micheli (USNM). Ponce, in black light trap, J. Micheli (JMPR): IX.17-23.1977; IX.24-30.1977. Ponce Playa, La Guancha, mud flat, VII.13.1978, J. Micheli (JMPR). Ponce Rd. 132, km 22, under stones, IX.24.1974, J. Micheli (USNM). Ponce C.CP.R., I.8.1979, J. Micheli (JMPR). Ponce, Valle Real, II.4.1979, J. Micheli (JMPR). Rd. 10, Km. 24, in black light trap, IX.23-29.1977, J. Micheli (JMPR). Rio Guanajibo at Sabana Grande, 100 m, V.4.1985, Hoebeke, Liebherr, Nichols (CUIC). Salinas, under stone, VII.6.1975, J. Micheli (JMPR). San German, Reserva Forest Maricao, Km 16.2 on Rte 120, uv light, VIII.8.1999, P.W. Kovarik (WIBF). 4.1 km SSE San Pedro, 18°22'35"N, 66°41'01"W, fields near Rio Grande de Arecibo, 300 m, VI.16.1996,Young, Klinglas, Davidson, Rawlins, Zarol, Thompson (CMNH). San Vicente, VII.25-28.1956, C. & P. Vaurie (MCZC). Santa Isabel, I.1975, J. Micheli (USNM). **Culebra Island.** III.14.1941, C. Parsons (MCZC). **Desecheo Island.** V.19-22.1975, S.J. Ramos (JMPR). **Isla Maguey.** Parguera, XII.18.1962, P. & P. Spangler (USNM). **Vieques Island.** XII.24-31.1935, S.T. Danforth (MCZC). **St. Croix.** (AMNH): III.1.1925; III.6.1925; III.10.1925; IV.9.1925. Blackwelder (USNM): XI.12.1936; XI.20.1936; XII.1.1936. 1937, H.A. Beatty (USNM); H.A. Beatty (MCZC). 4 km NW Christiansted, 17°45'N, 64°34'W, under leaf litter and wood debris on sandy soil, low dry scrub on exposed hilltop, II.12.1996, W.E. Steiner, J.M. Swearingen (USNM). Golden Grove, UV light, V.21.1980, D.F. Keaveny (UASM). Miller’s Bay, Frederiksted, III.3.1941 (USNM). Point Udall, crest, 17°46'N, 64°34'W, under leaf litter on sand at edge of ground cover vegetation on dry zone of upper beach, II.12.1996, W.E. Steiner, J.M. Swearingen (USNM). **VIRGIN ISLANDS. Anegada.** airport, VII.19.1985, S. & P. Miller (WIBF). **Camanoe.** at uv light, X.16.2005, B.D. Valentine family (BDVC). uv, X.16.2005, B.D.V. family (BDVC). **Great Camanoe.** (Upper) uv, B.D.V. family (BDVC): X.15.2006; X.21.2006; X.16.2006; X.21.2006; X.30.2006. uv, B.D. & S.C. Valentine (BDVC): X.16.2008; X.18.2008; X.19.2008; X.27.2008. uv, X.6.2008, B. Valentine, D. Dennis (BDVC). **Guana Island.** VII.1-14.1984, S.E. & P.M. Miller (WIBF). X.23-30.2002, B. & B. Valentine (BDVC). 0-80 m: VII.13-26.1986, S.E. Miller, M.G. Pogue (WIBF); VII.9-23.1987, S.E. Miller, V.O. Becker (USNM). at uv light, B. & B. Valentine (BDVC): X.20-22.2000; X.23-30.2002; X.2-7.2003; X.9-15.2003; X.12.2003. at generator, uv, IX.27.2006, J. Cokendolpher (BDVC). at uv light, B.D. Valentine, S.C. Valentine-Cooper (BDVC): X.22.2003; X.5.2004; X.8.2004; X.8-14.2004; X.13.2004; X.16.2004; X.19.2004; X.25.2004. at uv light, B.D. Valentine, S.C. Valentine-C (BDVC): IX.22.2005; IX.23.2005; IX.30.2005; X.1.2005. at uv light, B.D. & B.S. Valentine (BDVC): X.7.2005; X.13.2005. laundry area, uv, J. Ckendolpher (BDVC): IX.25.2006; IX.26.2006; X.4.2006; X.4-6.2006. sand pit, malaise, X.22-28.2001, B. & B. Valentine (BDVC). tool shed, uv, J. Cokendolpher (BDVC): IX.29.2006; X.1-2.2006. uv, B.D.V. (BDVC): IX.7.2006; X.9.2006; X.13.2006; X.17.2006; X.18.2006. The Flat, uv, S.C. Valentine-Cooper (BDVC): X.13.2008; X.28.2008. **St. John.** Caneel Bay, Lind Pt., R.T. Bell (RTBC): I.11.1966; XII.30.1965; I.9.1966; I.7.1966. Lind Point, V.30.1962, J.A. Muller (USNM). **St. Thomas.** (AMNH): III.12.1925; III.13.1925. Charlotte Amalie, VI.26, 1976, R.H. Pine (USNM). S central coast, XII.17.1975, R.D. Ward (CMNH). **Tortola.** XII.1928, S.T. Danforth (MCZC). **Virgin Gorda.** Pond Bay, ca. 5 m, VII.10.1984, D. Ford et al (WIBF). Virgin Peak, IV.15.1956, J.F.G. Clarke (USNM). **LESSER ANTILLES. ANGUILLA.** Low Ground, Black garden Rd., 18°13.795'N, 63°03.959'W, in/under rot. loblolly, M.A. Ivie (WIBF): V.16.2004; V.17.2004. Low Ground, Black garden Rd., 18°13.8'N, 63°03.96'W, in/under rot. loblolly, 16.5 m, V.17.2004, M.A. Ivie (WIBF). Low Ground, Black garden Rd., 18°13.795'N, 63°03.959'W, under rot. loblolly, 16.5, V.17.2004, M.A. Ivie (WIBF). Lower Shoal Bay (1.5 km SW), 18°15'10"N, 63°02'20"W, W.E. Steiner, J.M Swearingen (USNM): III.25.1992; III.28.1992. Meads Bay, 18°10.904'N, 63°08.186'W, dune behind beach, in/under rot. loblolly, 3 m, V.17.2004, M.A. Ivie (WIBF). Old Ta to Sandy Ground, 18°12.394'N, 63°03.969'W, 0-61 m, XI.16-20.1999, M.A. Ivie, J.B. Runyon (WIBF). Rendevous Bay, uv light, VII.11-16.1993, R.M. & H.V. Baranowski (FSCA). Sandy Ground to N. Shannon Hill and road to Salt Pond, 0-61 m, XI.8.1999, M.A. Ivie, J.B. Runyon (WIBF). West End, Altamir Hotel, sign light, V.16.2004, M.A. Ivie (WIBF). **Antigua.** (MCZC). D. & L. Stoner (AMNH): VI; VI.1918; VI.24.1918; VII. VI.21, L. Stoner (AMNH). “2-4-13”, P.G. Russel (USNM). Christian Hill, V.27.1978, Chalumeau (IREC). St. Johns, 0-100 m, VIII.1979, N.L.H. Krauss (AMNH). Stn. #278: VIII.18.1936, Chapin & Blackwelder (USNM); VI.27.1918 (AMNH). UV trap, IX.2.1969, H.A. Wright (FSCA). Christian Valley, bl trap, FAO Insect Survey (CMNC): VI.28-30.1991; VII.17.1991; VII.26.1991; X.8.1991. Christian Valley, blacklight trap, FAO Insect Survey (CMNC): IX.23.1991; X.12-13.1991. Christian Valley Agr. Station, uv light trap, FAO Insect Survey, R.E. Woodruff (CMNC): VIII.14.1991; IX.11.1991; X.10.1991. **Barbados.** VI, D. Stoner (AMNH). uv, VIII.23-25.1972, M.M. Alam (FSCA), (CMNC). **St. George.** Groves, uv, IX.20.1972, M.M. Alam (FSCA). **St. Michael.** Cavehill, uv, M.M. Alam: VII.10-30.1972 (FSCA), (UASM); VII.31-VIII.2.1972 (FSCA), (UASM); VIII.14.1972 (FSCA); VIII.16-17.1972 (FSCA), (UASM); VIII.17-18.1972 (UASM); VIII.18-20.1972 (FSCA), (UASM); VIII.21-22.1972 (FSCA); X.21-22.1972 (UASM). **St. Thomas.** Edgehill, uv, M.M. Alam (FSCA): X.2-3.1972; X.3-4.1972; X.5-6.1972; X.6-7.1972; X.7-8.1972; X.8-9.1972; X.12-13.1972; X.13-14.1972; X.15-16.1972; X.17-18.1972; X.21-22.1972; X.22-23.1972; XI.1-2.1972; XI.5-6.1972; XI.25-26.1972. Edgehill, uv light trap, X.21-22.1972, M.M. Alam (CMNC). **Barbuda.** Codrington, 0-20 m, VII.1976, N.L.H. Krauss (AMNH). **BEQUIA ISLAND.** Cinnamon Garden, 13°00'50"N, 61°14'W, dry scrub woodland, uv trap, 80 m, 08-77, VIII.3.2008, S. Peck, M. de Silva (CMNC). Spring Estate, 13°01'10"N, 61°13'45"W, dry scrub woodland, uv trap, 80 m, 08-74, VII.31.2008, S. Peck, M. de Silva (CMNC). **Buck Island.** B.I. Reef Nat. mon., Dedricks Shack, uv light, VIII.23.1996, A. Paponi (WIBF). **Canouan.** IV.1937, S.T. Danforth (MCZC). **Desirade.** IV.7.1973, Camb. (IREC). VI.9.1937 S.T. Danforth (MCZC). **Dominica.** Clarke Hall, O.S. Flint Jr. (USNM): IV.20.1964; IV.30.1964; V.8.1964; VI.15-30.1964. Clarke Hall (USNM): VI.1-10.1964, J.F.C.G. & T.M. Clarke; VII.7.1964, T.J. Spilman. **Grenada. St. George Parish.** Point Salina, VIII.2.1935, S.T. Danforth (MCZC). Point Salinas, l. trap, VI.27.1987, R. Woodruff (CMNC). St. Georges, VIII.1910, Allen & Brues (MCZC). **Guadeloupe.** Anse Lagourde (IREC): IX.10.1972, Chalumeau; IX.24.1972, F. Chalumeau. Basse-Terre, Saint-Claude, Ducharmony, IV.5.2002, J. Touroult (WIBF). Clugny, Chalumeau (IREC): I.22.1978; VIII.2.1970; IX.5.1971. Domaine Duclos, VI.24-28.1960, P. & C. Vaurie (AMNH). Dulcos (Htrs), IV.12.1979, Chalumeau (IREC). Gran-Terre, Anse de Tarare, 16°15.242'N, 61°11.901'W, coastal scrub, 0-20 m, VIII.20.2005, M.A. Ivie (WIBF). Portland (Mle), II.11.1979, Chalumeau (IREC). Pte Gr. Vigie, I.12.1975, Chalumeau (IREC). Pte Grde Vigie, XII.12.1971, Chalumeau (IREC). Viard (Pt-Bourg), IV.20.1973, Chalumeau (IREC). Viard (P. Bourg), IV.20.1973, F. Chalumeau (CMNH). **Les Saintes.** IV.14.1973, Chalumeau (IREC). TDH, IX.16.1978, Chalumeau (IREC). **Martinique.** 1 km E Diamant, 14°28.7'N, 61°00.6'W, thorn forest, uv trap, 10 m, 10-54, VII.7.2010, S. Peck (CMNC). St. Pierre, V.1.1975, Chalumeau (IREC). Diamant VI.17.1960, P. & C. Vaurie (AMNH). **MAYREAU.** 12°38.42'N, 61°23.56'W, cemetary, old field, uv trap, 72 m, 09-51, VIII.11.2009, S. Peck (CMNC). 12°38.56'N, 61°23.59'W, NW side of island, thorn scrub forest, uv trap, 50 m, 09-75, VIII.23.2009, S. Peck (CMNC). Saltwhistle Bay, 12°38.68'N, 61°03.95'W, S. Peck (CMNC): thorn scrub at pond, uv trap, 1 m, 09-53, VIII.12.2009; thorn scrub, 2 m, 09-79, VIII.24.2009. Station Hill, 12°38'N, 61°23'45"W, disturbed woodland, lights and gen. colln., 30 m, 08-85, VIII.11-15.2008, S. Peck, M. de Silva (CMNC). **Montserrat**. VI.30.1936, S.T. Danforth (MCZC); IV.21.1966, C.J. Edwards (CNCI). Blake’s, between dump & Jack Boy Hill, I.11.2002, K.A. Marske (WIBF). Cassa Ghaut, Beattie House, uv light, V.20.2003, K.A. Marske (WIBF). Cassa Ghaut, Beattie House, 16°45.91'N, 62°12.95'W, uv light, 192.6 m, III.23-IV.3.2002, A. Krakower (WIBF). Cassava Ghaut, Beattie House, uv light, V.20.2003, K.A. Marske (WIBF). Cassava Ghaut, Beattie House, 16°45.91'N, 62°12.95'W, light trap, 192.6 m, A. Krakower (WIBF): III.11-23.2002; VI.14-21.2002. Cedar Ghaut, 16°46.55'N, 62°12.34'W, uv light, VIII.6.2005, WIBF grp (WIBF). Delvine Village, V.28.1982, Chalumeau (IREC). E side, N of airport, nr dump, VI.26.2000, K.A. Guerrero (WIBF). East side, nr coast, 16°46.57'N, 62°10.27'W, *Acacia* scrub, dung bait pitfall, 82.3 m, V.27-VI.24.2002, K.A. Marske (WIBF). East Side Road, N of airport, 16°46.283'N, 62°10.254'W, under rocks, 190.5 m, VI.21.2000, M.A. Ivie, K.A. Guerrero (WIBF). Big River, 16°45.719'N, 62°11.34'W, uv light, 374.9 m, VIII.7.2005, WIBF grp (WIBF). Hope Ghaut, Beattie House, 16°45.91'N, 62°12.95'W, uv light, VI.21-30.2002, M.A. ivie (WIBF). Jack Boy Hill, 16°45.77'N, 62°10.98'W, overnight, VII.10.2005, WIBF grp (WIBF). Little Bay to Rendezvous Bay trail, I.7.2002, M.A. Ivie, K.A. Marske (WIBF). Olveston school, tennis courts, at light, VII.7.2002, K.A. Marske (WIBF). Plymouth, J. Cooter (BMNH): VIII.31.1975; IX.10.1975. Rendevous Bay, 16°48.496'N, 62°12.298'W, uv light, sea level, VII.26-31.2005, WIBF (WIBF). Rendezvous Bay, 16°48.58'N, 62°12.30'W (WIBF): uv light trap, VII.26-31.2005, WIBF grp; uv light, VIII.8.2005, I.A. Foley. Richmond Hill, nr Plymouth, IX.10.1975, J. Cooter (BMNH). Soufriere Galway’s. V.30.1982, Chalumeau (IREC). Spanish Point, VIII.31.1975, J. Cooter (BMNH). v. Statue Rock, III.23.1984, Chalumeau (IREC). stn #264, VII.22.1966, Blackwelder (USNM). stn #267, VII.22.1966, Blackwelder (USNM). Woodlands, Riverse House, 16°45.99'N, 62°13.34'W, WIBF grp (WIBF): uv light, 42.7 m, VII.25.2003; VIII.20-23.2005. Woodlands, Riverside House, 16°45.99'N, 62°13.34'W, I.A. Foley (WIBF): VI.21.2005; uv light, VII.31.2005. **Mustique.** H.H. Smith (BMNH). Rutland Bay, 12°53'N, 61°11'W, dry woodland, forest edge, uv trap, 5 m, 08-73, VII.29.2008, S. Peck, M. de Silva (CMNC). **Nevis.** VI.29.1935, S.T. Danforth (MCZC). beach on NW shore, I.20.2004, M.A. & L.L. Ivie (WIBF). Hurricane Hill, blacklight trap, VIII.14.1992, H.V. & R.M. Baranowski (CMNC). **REDONDA.** 16°56.36'N, 62°20.75'W, 152.4-274.3 m, VIII.6.2005, V.G. Martinson (WIBF). **SABA.** The Bottom, attr. to lights, XI.22.2010, M. Gillet (WIBF). Spring Bay Trail, 17.63334°N 63.22100°W, xeric scrub, under rocks, V.20.2008, D.S. Sikes (WIBF). **SOMBRERO.** At light, XI.14.1999, M.A. Ivie, J.B. Runyon (WIBF). **St. BarthÉlemy.** Anse Gr. Saline, V.30.1973, Chalumeau (IREC). Lorient, stn #448, VI.3.1949, P. Wagenaar Hummelinck (ZMAN). **St. Eustatius.** VIII.26.1917 (AMNH); VI.21.1937, S.T. Danforth (MCZC). stn #297, III.18.1937, P. Wagenaar Humelinck (ZMAN). **St. Kitts.** Basseterre, VIII.5.1931, S.T. Danforth (MCZC). stn #296, III.19.1937, P Wag. Hummelinck (ZMAN). stn #297, III.18.1937, P. Wag. Hummelinck (ZMAN). SE peninsula, Majors Bay, light trap, IX.3.1991, R.E. Woodruff (CMNC). Needsmost (Needsmust), light trap, IX.12.1987, T. Blanchette (CMNC). **St. Lucia.** Anse La Raye, Anse Galet, 1 km SSW Anse La Raye, 50 m, VI.21-30.1991, J. Rawlins, S. Thompson (CMNH). Bouton, 13.8795°N 61.0707°W, hand collecting, VII.10.2009, C.A. Maier (WIBF). Escap Comm., 13.8324°N 60.8986°W, merc vapor light, 30 m, V.1.2009, I.A. Foley (WIBF). Escap Community, 13.8324°N 60.8986°W, 46 m, VI.15-22.2009, WIBF group (WIBF). Escap Community, 13.83242°N 60.89864°W, at house, 46 m, I.A. Foley, R.C. Winton (WIBF): IV.26-30.2009; V.1-10.2009. Escap Community, 13.83242°N 60.89864°W, ridge, uv light, V.1.2009, I.A. Foley (WIBF). Escap Community, 13.83242°N 60.89864°W, merc vapor, VI.16-19.2009, R.C. Winton, E.A. Ivie (WIBF). Escap Comm-Fond Bay, 13.83242°N 60.89864°W, IV.27.2009, I.A. Foley, R.C. Winton (WIBF): 1-46 m; in rotten log, 1-46 m. Escap Comm-Fond Bay, 13.83242°N 60.89864°W, 1-46 m, V.14-16.2009, R.C. Winton, E.A. Ivie (WIBF). Gros Ilet, IV.11.1978, Chalumeau (IREC). SE of La Pointe, 13.8398°N 60.8887°W, 73 m, VI.03.2009, I.A. Foley (WIBF). Marigot Bay, blacklight trap, VIII.22.1988, M. Paul (CMNC). Mon Repos, Fox Grove Inn, 13°51.8'N, 60°54.4'W, uv light into trees, 90 m, S. & J. Peck (CMNC): 07-50, VII.8-18.2007; 07-75, VII.20-28.2007. near Mon Repos, 13°51.87'N, 60°54.45'W, VI.13.2009, S.M. Clark et al (WIBF). summit of Pigeon Island N.P., under leaves of surface crawling plants, 91.4 m, VII.30.1989, W.M. Graham, R. Freitag (UASM). R. Galet, S of Dennery, VIII.1.1963, Flint & Cadet (USNM). Vergallier R. nr Marquis, VII.31.1963, Flint & Cadet (USNM). **St. Martin.** Baie de Friar, V.26.1973, Chalumeau (IREC). Galion, V.3.1981, Chalumeau (IREC). Great Bay, ST Danforth: XI.21.1927 (AMNH); XI.21.1927 (MCZC); XII. 24.1927 (MCZC). Marigot, V.26.1973, Chalumeau (IREC). **ST. VINCENT. St. Andrew Parish.** Buccament, Emerald Valley, 13°12.0'N, 61°13.8'W, dry forest remnant, uv light, 30 m, 07-20, VI.12-19.2007, S. & J. Peck (CMNC). E. Layou, Emerald Valley Hotel, 13°12.0'N, 61°14.8'W, 20 m, VIII.27-29.2006, S. & J. Peck (CMNC): streamside, uv trap, 06-122; forest edge, uv, 06-123. **St. George Parish.** Brighton Bay Village, 13°07.97'N, 61°10.06'W, streamside, uv traps, 1 m, 07-10, VI.8.2007, S. & J. Peck (CMNC). Stubbs, 13°08.9'N, 61°10.121'W, pasture-fallow field, uv light, 75 m, 07-11, VI.9.2007, S. & J. Peck (CMNC). **St. Patrick Parish.** Wallilabou Bay, 13°14.9'N, 61°16.2'W, uv trap, 2 m, S. & J. Peck (CMNC): hotel grounds, coastal grove, 06-118, VIII.23.2006; nr. mouth, coastal, 06-121, VIII.26.2006.

### *Selenophorus yucatanus* Putzeys

**LESSER ANTILLES. GRENADA. St. George Parish.** Lance aux Epines, Coral Cove, 11°59.570'N, 61°45.277'W, sand beach, uv trap, 1 m, 10-78, VIII.28.2010, S. Peck (CMNC). Point Salinas, l. trap, VI.27.1987, R. Woodruff (CMNC). **MAYREAU.** east beach, manchineel forest, uv trap, 1 m, VIII.13.2008, S. Peck, M. de Silva (CMNC). Station Hill, 12°38'N, 61°23'45"W, disturbed woodland, lights and gen. colln., 30 m, 08-85, VIII.11-15.2008, S. Peck, M. de Silva (CMNC). **Mustique.** II.29.1937, S.T. Danforth (MCZC). **Union Island.** IV.28.1937, S.T. Danforth (MCZC). Campbell, Miss Irene Reserve, 12°35.44'N, 61°27.34'W, high canopy thorn forest, 85 m, 09-66, VIII.18.2009, S. Peck (CMNC). Clifton, top of Fort Hill, 12°36.12'N, 61°24.93'W, thorn scrub, 2 uv traps, 125 m, 09-59, VIII.15.2009, S. Peck (CMNC).

### *Selenophorus barbadensis* Ball & Shpeley

**LESSER ANTILLES. Barbados.** Uv, VIII.23-25, M.M. Alam (FSCA). **St. Michael.** Cavehill, UV light, IX.20.1972, M.M. Alam (UASM). Cavehill, uv, M.M. Alam (FSCA): VII.10-30.1972; VIII.8-10.1972; VIII.21-22.1972. **St. Thomas.** Edgehill, uv, M.M. Alam (FSCA): X.15-16.1972; X.21-22.1972; XI.25-26.1972. **ST. VINCENT. St. Andrew Parish.** E. Layou, Emerald Valley Hotel, 13°12.0'N, 61°14.8'W, forest edge, uv., 20 m, 06-123, VIII.27-29.2006, S. & J. Peck (CMNC).

### *Selenophorus latior* Darlington

**GREATER ANTILLES. Hispaniola: Dominican RepUBLIC. La Altagracia.** vic. Oyo Clara, 18°33'48"N, 68°26'50"W, uv light, VII.24-VIII.5.2002, K.W. Will, C. Chaboo (EMEC). **Puerto Rico.** Bosque de Guanica, VI.9.2005, R. Brown, S. Lee (MEMU): 17°58'50"N, 66°52'37"W, 15W BL, sheet in dry coastal forest at Cobanus Rd.; 17°58'31"N, 66°52'45"W, 15W BL, box trap with ammonia, dry coastal forest at Ojo de Agua. Cabo Rojo S of Corozo, V.2.1985, Liebherr et al (CUIC). Mayaguez, IV.7.1935, Cedo (MCZC). Pt. Cangrejos, G.N. Wolcott: II.8.1920, (USNM); V.28.1920 (UASM). Rio Piedras, at light, VI.14.1934, AS Mills (USNM). **Virgin IsLANDS. Great Camanoe.** (Upper) uv, B.D.V. family (BDVC): X.9.2007; X.27.2007. uv, X.9.2007, B.D. Valentine family (UASM). uv, B.D. & S.C. Valentine (BDVC): X.7.2008; X.16.2008; X.17.2008; X.18.2008; X.19.2008. uv, B.D. & S.C. Valentine (UASM): X.16.2008; X.27.2008. uv, X.6.2008, B. valentine, D. Dennis (UASM). **Guana Island.** 0-80 m, S.E. Miller, V.O. Becker: VII.9-23.1987 (WBIF); VII.9-23.1987 (UASM); VII.9-23.1987 (USNM); VII.9-23.1987 (BPBM). 0-80 m, VII.13-26.1986, S.E. Miller, M.G. Pogue (WIBF). laundry area, J. Cokendolpher (BDVC). uv, X.10.2005, BDV & SCV-C (BDVC). uv, X.12.2005, B.D. & S.C. Valentine (BDVC). uv light, X.24.2004, B.D. Valentine, S.C. Valentine-Cooper (BDVC). The Flat, malaise, X.2008, S.C. Valentine-Cooper (BDVC). **Tortola.** Road Town, VIII.3.1976, R.H. Pine (USNM). **LESSER ANTILLES. Grenada. St. Andrew Parish.** Balthazar Estate, blacklight trap, VI.12.1990, J.H. Frank, M.C. Thomas (FSCA). Balthasar R., 1.6 km E Grenville, UV light, VI.13.1977, R.E. Woodruff, E.E. Grissell (FSCA). **Guadeloupe.** Viard (P. Bourg), F. Chalumeau: IV.20.1973 (CMNH); IV.20.1973 (IREC).Viard (Pt-Bourg), IV.20.1973, Chalumeau (IREC). Viard (P. Brg), IX.7.1974, Chalumeau (IREC). Viard (P. Bourg), IX.8.1974, F. Chalumeau (IREC).Viard (P. Brg), IX.8.1974, Chalumeau (IREC). **MAYREAU.** Saltwhistle Bay, 12°38.68'N, 61°03.95'W, S. Peck (CMNC): thorn scrub at pond, uv trap, 1 m, 09-53, VIII.12.2009; salt flat, thorn scrub, uv trap, 0.5 m, 09-54, VIII.12.2009. **MUSTIQUE.** l’Ancecoy Pont, 12°53'N, 61°11'W, dry scrub woodland, uv trap, 1 m, 08-72, VII.30.2008, S. Peck, M. de Silva (CMNC). Rutland Bay, 12°53'N, 61°11'W, dry woodland, forest edge, uv trap, 5 m, 08-73, VII.29.2008, S. Peck, M. de Silva (CMNC). **ST. KITTS.** Needsmost (Needsmust), light trap, IX.12.1987, T. Blanchette (CMNC). **St. Lucia.** Escap Community, 13.83242°N 60.89864°W, merc vapor, VI.14.2009, R. Winton, E.A. Ivie (WIBF). Gros Ilet, IV.11.1978, Chalumeau (IREC). Mon Repos, Fox Grove Inn, 13°51.8'N, 60°54.4'W, uv light into trees, 90 m, 07-75, VII.20-28.2007, S. & J. Peck (CMNC). Praslin, 13°52.7'N, 60°53.7'W, beach, mangrove, uv traps, 0.5 m, 07-68, S. & J. Peck (CMNC). **ST. VINCENT. St. Andrew Parish.** Buccament, Emerald Valley, 13°12.0'N, 61°13.8'W, dry forest remnant, uv light, 30 m, 07-20, VI.12-19.2007, S. & J. Peck (CMNC). Buccament, Emerald Valley Hotel, 13°12.0'N, 61°13.8'W, uv light trap, 20 m, 07-12, VI.10-20.2007, S. & J. Peck (CMNC). Buccament Bay, 13°11.5'N, 61°00'W (should read 61°16.0'W), estuary, uv light, 1 m, 07-22, VI.20.2007, S. & J. Peck (CMNC). E Layou, Emerald Valley Hotel, 13°12.0'N, 61°14.8'W, forest edge, uv., 20 m, 06-123, VIII.27-29.2006, S. & J. Peck (CMNC). **St. George Parish.** Brighton Bay Village, 13°07.97'N, 61°10.06'W, streamside, uv traps, 1 m, 07-10, VI.8.2007, S. & J. Peck (CMNC). Stubbs, 13°08.9'N, 61°10.121'W, pasture-fallow field, uv light, 75 m, 07-11, VI.9.2007, S. & J. Peck (CMNC). **St. Patrick Parish.** Wallilabou Bay, 13°14.9'N, 61°16.2'W, river mouth, coastal, uv trap, 2 m, 06-121, VIII.26.2006, S. & J. Peck (CMNC).

### *Selenophorus solitarius* Darlington

**GREATER ANTILLES. CUBA. Cienfuegos.** Cayamas, I.14, E.A. Schwarz (USNM).

### *Selenophorus spinosus* sp. n.

**LESSER ANTILLES. GRENADA. St. Andrew Parish.** Mirabeau, Agr. Lab, light trap, VI.28.1990, J. Telesford (CMNC).

### *Selenophorus clypealis* Ball & Shpeley

**GREATER ANTILLES. Hispaniola: Dominican RepUBLIC. Independcia.** 4 km S Los Pinos, Loma de Vientos, 455 m, 18°35'N, 71°46'W, semiarid deciduous forest with pastures, VII.23.1992, C. Young, S. Thompson et al (CMNH). **Pedernales.** Cabo Rojo, at light, 0-10 m, IX.10.1988, M. Ivie, Philips, Johnson (WIBF). Cabo Rojo, sea level, 17°55n 71°39'W, edge of salt marsh, X.21.1991, Davidson, Rawlins et al (CMNH). **Hispaniola: Haiti. Artibonite.** source Matelas, IX.5.1934, Darlington (MCZC). **Virgin IsLANDS. Little St. James.** X.9-X.11.1999, W. Lu (BDVC).

### *Selenophorus dessalinesi* Ball & Shpeley

**GREATER ANTILLES. Hispaniola: Dominican Republic. Hato Mayor.** Parque Los Haitises, E of Trepada Alta, 12 km W El Valle, 18°59'N, 69°30'W, mesic forest on limestone, 145 m, VII.6.1992, J. Rawlins, S. Thompson, C. Young, R. Davidson (CMNH). **Monte Cristi.** 5 km NNE Botoncillo, 19°46'N, 71°24'W, arid thorn scrub, 50 m, XI.29-30.1992, R. Davidson, M. Klingler, S. Thompson, J. Rawlins (CMNH). Monte Cristi, VI.21.1967, L.H. Rolston (TAMU). **Hispaniola: Haiti. Artibonite.** swamps N of Dessalines, IX.11.1934, Darlington (MCZC).

### *Selenophorus parvus* Darlington

**GREATER ANTILLES. Puerto Rico.** La Parguera, H. & A. Howden (UASM): VII.28.1969; VII.30.1969. **not located.** Smythe, Wolcott (UASM). **LESSER ANTILLES. Barbados.** uv, M.M. Alam: VIII.23-25.1972 (FSCA), (UASM); VIII.26-29.1972 (FSCA); VIII.31-IX.2.1972 (FSCA). Graeme Hall, Entomology Compound, blacklight trap, IX.6.1988, R. Adams (CMNC). **St. George.** Groves, uv, M.M. Alam (FSCA): IX.5.1972; IX.7.1972; IX.9.1972; IX.10.1972; IX.11.1972; IX.12.1972; IX.13.1972; IX.14.1972; IX.16.1972; IX.18.1972; IX.23.1972; IX.24.1972; IX.25.1972; IX.26.1972; IX.27.1972; IX.29.1972; XII.5.1972. Groves, uv light, IX.26.1972, M.M. Alam (UASM). **St. Michael.** Coast 9.7 km N Bridgetown, VII.25-30.1972, D. Miller (AMNH). Cavehill, uv, VII.1-9.1972, M.M. Alam (FSCA). Cavehill, uv, M.M. Alam (FSCA), (UASM): VII.10-30.1972; VII.31-VIII.2.1972; VIII.3.1972; VIII.5-7.1972; VIII.8-10.1972; VIII.11-13.1972; VIII.14.1972; VIII.16-17.1972; VIII.18-20.1972; VIII.21-22.1972. Cavehill, M.M. Alam (CMNC): blacklight trap, VII.31-VIII.2.1992; uv light, VII.10-30.1972. **St. Thomas.** Edgehill, uv, M.M. Alam (FSCA): X.1-2.1972; X.2-3.1972; X.3-4.1972; X.4-5.1972; X.5-6.1972; X.6-7.1972; X.7-8.1972; X.8-9.1972; X.9-10.1972; X.10-11.1972; X.11-12.1972; X.12-13.1972; X.13-14.1972; X.14-15.1972; X.15-16.1972; X.16-17.1972; X.17-18.1972; X.18-19.1972; X.19-20.1972; X.20-21.1972; X.21-22.1972; X.22-23.1972; X.26-27.1972; X.29-30.1972; X.30-31.1972; X.31-XI.1.1972; XI.1-2.1972; XI.5-6.1972; XI.6-7.1972; XI.14-15.1972; XI.15-16.1972; XI.17-18.1972; XI.18-19.1972; XI.20-21.1972; XI.21-22.1972; XI.23-24.1972; XI.25-26.1972. Edgehill, uv light, M.M. Alam (UASM): X.7-8.1972; X.15-16.1972; X.21-22.1972; X.29-30.1972XI.23-24.1972; XI.25-26.1972. Edgehill, uv light, X.22-23.1972, M.M. Alam (CMNC). Welchman Hall, 13°11.74'N, 59°34.60'W, gully forest, uv light trap, 270 m, 06-59, V.26.2006, S. & J. Peck (CMNC). **Barbuda.** Codrington, at light, E.J. Pearce (BMNH): VI.30.1962; VII.1.1962. Holetown, at light, VIII.5-6.1978, G.E. Ball (UASM). **BEQUIA ISLAND.** Hamilton, 13°00'30"N, 61°14'W, woodland, forest edge, uv trap, 25 m, 08-75, VIII.1.2008, S. Peck, M. de Silva (CMNC). Hope Estate, 13°00'30"N, 61°13'30"W, dry scrub woodland, uv trap, 100 m, 08-78, VIII.4.2008, S. Peck, M. de Silva (CMNC). Spring Estate, 13°01'10"N, 61°13'45"W, dry scrub woodland, uv trap, 80 m, 08-74, VII.31.2008, S. Peck, M. de Silva (CMNC). Spring Estate, 13°01'10"N, 61°14'W, dry scrub woodland, uv trap, 100 m, 08-76, VIII.2.2008, S. Peck, M. de Silva (CMNC). **CANOUAN.** Mahault Bay, 12°43'45"N, 61°19'15"W, dry scrub woodland, uv trap, 30 m, S. Peck, M. de Silva (CMNC): 08-80, VIII.6.2008; 08-84, VIII.9.2008. **GRENADA. St. Andrew Parish.** Mirabeau, Agr. Lab., l. trap (CMNC): IX.10.1990, A. Thomas; IX.12.1990, J. Telesford. **MARTINIQUE**. ZNIEFF 35, Les Anses-d’Arlet, 14.46017N 61.06340W, light trap, X.15.2015, E. Poirier (JMLC). **MUSTIQUE.** Rutland Bay, 12°53'N, 61°11'W, dry woodland, forest edge, uv trap, 5 m, 08-73, VII.29.2008, S. Peck, M. de Silva (CMNC). **St. Lucia.** Fond d’Or Bay, XII.21.1975, Chalumeau (IREC). Mon Repos, Fox Grove Inn, 13°51.8'N, 60°54.4'W, uv light into trees, 90 m, S. & J. Peck (CMNC): 07-50, VII.8-18.2007; 07-75, VII.20-28.2007. Praslin, 13°52.7'N, 60°53.7'W, beach, mangrove, uv traps, 0.5 m, 07-68, S. & J. Peck (CMNC). Thomazo, XII.20.1975, Chalumeau (IREC).

### *Selenophorus subquadratus* (Putzeys)

**GREATER ANTILLES. CUBA.** F.C. Bowditch (MCZC). **Cienfuegos.** Soledad, Darlington (MCZC): VI.1929; IV.1936. **Habana.** Laguna Somorrostro, VI.1956 (MNHC). **Pinar del Rio.** Estacion El Taburete, Sierra del Rosario, X.1998, P. Valdez (PVRC). S. del Rosario, X.12.1996, P. & A. (PVRC). **Hispaniola: Dominican RepUBLIC. La Vega.** 2 km E Manabao, ultraviolet & mercury vapor light, VII.18.1996, M.C. Thomas, R. Turnbow (FSCA). **San Cristobal.** Villa Altagracia, VII.1938, Darlington (MCZC). **Puerto Rico.** Carolina, VII.1978, J. Micheli (JMPR). Humacao, at light, III.18.1940, J.A. Ramos (MCZC). Luquillo, VII.8.1932, J. Blanch (MCZC). Rd. 10, in black light trap, IX.23-29.1977, J. Micheli (JMPR). Rd. 365, Km. 7.4, in forest litter, X.1978, J. Micheli (JMPR). **LESSER ANTILLES. Dominica.** Bellevue-Chopin, V.22.1973, Chalumeau (IREC). **Guadeloupe.** Basse-Terre, Matouba, St. Claude, VIII.8.2003, J. Touroult (WIBF). FWI, Basse-Terre, Sainte-Rose, Sofia, XI.22.1999, M.A. Ivie (WIBF). Prise d’Eau, IX.25.1970, Chalumeau (IREC). Verou, I.2.1973,Chalumeau (IREC). **Martinique.** Le Precheur,VIII.16.1983, Chalumeau (IREC). MONTSERRAT. Woodlands, Riverside House, 16°45.99'N, 62°13.34'W, VIII.7-16, M.A. & L.L. Ivie (WIBF). **SABA.** Mountain Road, at light, IX.7.2010, M. Gillet (WIBF). **St. BarthÉlemy.** Quat./Flamands, V.29.1973, Chalumeau (IREC). **St. Kitts.** Philips P.L., XII.6.1983, Lalanne-Cassou (IREC). Phillips Level P.L., XII.9.1983, Lalanne-Cassou (IREC). Molineux (Philips), IV.5.1980, Chalumeau (IREC).

### *Selenophorus mundus* Putzeys

**GREATER ANTILLES. Hispaniola: Dominican Rep. Azua.** 8 km NE Padre Los Casas, Rio Las Cuevas, 580 m, 18°46'N, 70°53'W, VIII.7.1990, J. Rawlins, S. Thompson (CMNH). **Barahona.** Barahona, IX.1938, Darlington (MCZC). **Independencia.** ESE Jimani, S Lago Limon, 18°24'N, 71°44'W, uv light, 20 m, VII.3.1992, M.A. & R.O. Ivie (WIBF). S of Lago Enriquillo, 18°24N 71°42W, uv light, 20 m, VII.18.1993, D.S. Sikes, R. Rosenfeld (WIBF). 4 km S Los Pinos, Loma de Vientos, 455 m, 18°35'N, 71°46'W, semiarid deciduous forest with pastures, VII.23.1992, C. Young, S. Thompson et al (CMNH). **Monte Cristi.** 5 km NNE Bontoncillo, 50 m, 19°45'N, 71°42'W, arid thornscrub, XI.29-301992, Davidson, Klingler et al (CMNH). 3 km N Villa Elisa, uv, X.1.1985, Woodruff & Stange (FSCA). 8.2 km N Villa Elisa, mv & bl l, VI.1.1994, R. Turnbow (UASM). **Pedernales.** Cabo Rojo,V.18.1994, R. Turnbow (UASM). Cabo Rojo, dead in light fixture, IX.10.1988, Ivie, Philips, Johnson (WIBF). Cabo Rojo, in pool & at light, 0-10 m, VIII.18-23.1988, Ivie, Philips, Johnson (WIBF). 10 km E Cabo Rojo, under dry cow dung in desert, VI.23.1999, R.E. Woodruff (FSCA). ca. 35 km NNW Cabo Rojo, El Aceitillan, 1430 m., dry pine forest, pine needles & rot. wood, VIII.20.1988, Ivie, Philips, Johnson (WIBF). Sa. de Baoruco, N Cabo Rojo, km 33, 1500 m, pine forest on limestone, II.10.1975, W.L. & D.E. Brown (MCZC). **Puerto Plata.** Los Hidalgos, VI.4-5.1969, Flint & Gomez (USNM). **San Juan.** San Juan de Maguana, “5-10-1928”, Ciferri (MCSN). **Hispaniola: Haiti. Artibonite.** source Matelas, IX.5.1934, Darlington (MCZC). **Ouest.** Kenskoff, (nr P. au P.), 1219-1829 m, IX.2.1934, Darlington (MCZM). Pont Beudet, 30.5 m, II.3-III.4.1922, (AMNH). Port au Prince (MCZC): 1931, E. Ducasse; 1934, Darlington. Port au Prince, and vic., X.5.1934, Darlington (MCZC).

### *Selenophorus pseudomundus* Ball & Shpeley

**GREATER ANTILLES. Hispaniola: Dominican Republic. Barahona.** 16 km W Barahona, VIII.29.1997, P.W. Kovarik (WIBF). **Independencia.** ese Jimani, S Lago Limon, 18°24'N, 71°44'W, uv light, 20 m, VII.3.1992, M.A. & R.O. Ivie (WIBF). **Pedernales.** Cabo Rojo, at light, 0-10 m, VIII.26.1988, M.A. Ivie, T.K. Philips, K.A. Johnson (WIBF). 9.5 km N Cabo Rojo, 18°00.042'N, 71°38.793'W, light, beating, 33 m, VIII.8.1999, M.A. Ivie, K.A. Guerrero (WIBF). 10.2 km N Cabo Rojo, VII.9.1996, M.C. Thomas (FSCA). Las Mercedes, 21 km N Cabo Rojo, 490 m, VII.10.1987, J. Rawlins, R. Davidson (CMNH). **Peravia.** 12.4 km E Rio Ocoa, VII.3.1992, M.A. & R.O. Ivie (WIBF).

### *Selenophorus nonseriatus* Darlington

**GREATER ANTILLES. Cuba. Habana.** Jaruco, Jaula, Cuevo el Indio, entrada, III.28.1997, P. & A. (PVRC). **Pinar del Rio.** Las Animas, Sierra Rangel, 457.2 m, IX.3-5.1934, S.C. Bruner, A.R. Otero (MNHC). Rangel Mts., 457.2 m, VIII.24.1936, Darlington (MCZC). Sierra del Rangel, VI.1950, M. Barro (MNHC). **Villa Clara.** north end of Lago del Hanabanilla, VII.1.1990, J.E. Rawlins & S. Thompson (CMNH). **Hispaniola: Dominican RepUBLIC. Barahona.** Filipinas, 18°07.339'N, 71°07.152' W, blacklight/night beating, 625 m, VII.7.2004, S.W. Lingafelter (USNM). nr Filipinas, Larimar Mine, VI.26-VII.7.1992, Woodruff & Skelley (FSCA): at light; night beating. nr Filipinas, Larimar Mine, R.E. Woodruff, P.E. Skelley (FSCA): at light, VI.20-26.1992; at night, VI.20-26.1992; flight trap, VI.26-VII.7.1992. 9.2 km NW Paraiso, confluence of Rio Nizao and Rio Cottico, 230 m, 18°03'N, 71°12'W, VIII.9-10.1990, Thompson, Rawlins et al (CMNH). 2 km E Payoso, mv & bl, VII.13.1996, R. Turnbow (RHTC). 2 km E Payoso, mercury vapor & ultraviolet light, VII.13.1996, M.C. Thomas (FSCA). **Dajabon.** 9 km S Loma de Cabrera, 620 m, 19°21'N, 71°37'W, disturbed pastures in mesic woodland, VII.12.1992, C. Young, S. Thompson et al (CMNH). **El Seibo.** Loma Cocuyo, 6 km N Pedro Sanchez, 475 m, 18°55N 69°07W, disturbed fields and woodland, VII.4.1992, Davidson, Rawlins et al (CMNH). 11.3 km N Pedro Sanchez, Loma de Chivo, 1524 m., blacklight trap, VI.20.1998, R.E. Woodruff, P.H. Freytag (FSCA). **Elias Pina.** 4 km SE Rio Limpio, 760 m, V.24-25.1973, D. & M. Davis (USNM). San Francisco Mts., IX.5, A. Busck (USNM). **Hato Mayor.** Par. Nac. Los Haitises, F.I.T. #1, bosquehumido, IV.16-VI.1.1992, Ivie, Sikes, Lanier (WIBF). Parc. Nac. Los Haitises, 19°05'N, 69°29'W, leaf litter, s. l., VII.19.1993, D. Sikes, R. Rosenfeld (WIBF). Parc. Nac. Los Haitises, W of Sabana de la Mar, Bosque Humido, litter in buttresses, IV.1.1992, M.A. Ivie, D.S. Sikes (WIBF). Parc. Nac. Los Haitises, W of Sabana de la Mar, FIT, 10 m, IV.1-16.1992, M.A. Ivie (WIBF). Parc. Nac. Los Haitises, W of Sabana de la Mar, Bosque Humido, FIT, IV.16-VI.1.1992, M.A. Ivie (WIBF). Parc. Nac. Los Haitises, D. Sikes, R. Rosenfeld (WIBF): FIT, VII.18-21.1993; berlese leaf litter, VII.20.1993; FIT, VII.22-31.1993. Parque Los Haitises, E of Trepada Alta, 12 km W El Valle, 145 m, 18°59'N, 69°30'W, mesic forest on limestone, VII.6.1992, Davidson, Rawlins et al (CMNH). Parque Los Haitises, 3 km W Cueva de Arena, 19°04'N, 69°29'W, mesic lowland forest, VII.7-9.1992, Davidson, Rawlins et al (CMNH). P.N. Los Haitises, FIT, VII.1992-VII.16.1993, D.S. Sikes (WIBF). **La Vega.** Constanza to Jarabacoa, 609.6-1219.2 m, VIII.1938, Darlington (MCZC). **Monte Cristi.** 5 km NNE Bontoncillo, 50 m, 19°46'N, 71°42'W, arid thornscrub, XI.29-30.1992, Davidson & Klingler (CMNH). **Pedernales.** 9 km N Cabo Rojo, 35 m, 18°02'N, 71°39'W, desert scrub, intercept trap, VII.13-19.1990, Masner, Rawlins & Young (CMNH). 23.5 km N Cabo Rojo, 540 m, 18°06'N, 71°38'W, deciduous forest, intercept trap, VII.19-25.1990, Masner, Rawlins & Young (CMNH). 26 km N Cabo Rojo, 730 m, 18°06'N, 71°38'W, mesic deciduous forest with scattered pines, VII.16.1992, Davidson, Rawlins et al (CMNH). 30 km N Cabo Rojo, 18°27.282'N, 71°35.943'W, uv & merc. vap. light, 1002.8 m, VII.21.1999, M.A. Ivie, G.O. Dominici (WIBF). La Aguita, N of Pedernales, 18°09.172'N, 71°44.786'W, VII.21.1999, M.A. Ivie, Guerrero (WIBF). along Rio Mulito, 13 km N Pedernales, 230 m, 18°09'N, 71°46'W, riparian woodland, VII.17.1992, C. Young, S. Thompson et al (CMNH). **Puerto Plata.** Pico El Murazo, north slope near summit, 910 m, 19°41'N, 70°57'W, mesic deciduous forest, XI.28.1992, Davidson & Klingler et al (CMNH). **Samana.** Sanchez, VII.1938, Darlington (MCZC). **San Cristobal.** Villa Altagracia, VII.1938, Darlington (MCZC). **Santiago.** foothills Cordillera Central, S of Santiago, VI.1938, Darlington (MCZC). Mt. Diego de Ocampo, 914.4-1219.2 m, VII.1938, Darlington (MCZC). Par. Nac. A Bermudez, Los Tablones, on Rio Izquiera, 19°03'N, 70°50'W, flight intercept trap, 1290 m, IV.9-VII.7.1992, M.A. & R.O. Ivie (WIBF). **Hispaniola: Haiti. Grand’Anse.** N.E. Foothills, La Hotte, 609.6-1219.2 m, X.10-24.1934, Darlington (MCZC). **Ouest.** La Visite & vic., LaSelle Range, 1524-2133.6 m, IX.16-23.1934, Darlington (MCZC). **Jamaica. St. Ann Parish.** Claremont, III.14.1921 (AMNH). **LESSER ANTILLES. Dominica.** Morne Macaque, XI.11.1976, Chalumeau (IREC). **Grenada. St. Andrew Parish.** Balthazar, windward side, H.H. Smith (BMNH). **St. Vincent.** H.H. Smith (BMNH).

### *Selenophorus fabricii* sp. n.

**UNITED STATES. Florida.** Monroe County. Sugarloaf Key, Sec. 25 SE ¼, Kitchings Hammock, uv trap 92-314, VIII.10-19.1992, S. & J. Peck (CMNC). Middle Torch Key, Sec. 17, Lazele Pl., uv light trap 92-315, VIII.10-19.1992, S. & J. Peck (CMNC). Big Pine Key, N end, mangrove hw. transition, uv light, VI.1990, E. Peck (CMNC). **BAHAMAS. Andros Island.** VIII.1-10.1904, Barber (MCZC). Forfar Field Stn., nr. Stafford Creek, uv trap in coastal coppice, VII.22028.2006, M.C. Thomas, T.R. Smith (UASM). Fresh Crk, Andros Town Androsia, high interior coppice, black light, VIII.5-6.1987, J. Browne (CMNC). Mennenite’s Farm, crop, black light, VII.30.1987, J. Browne (CMNC). Red Bays, Lewis Coppit, Shadrock Russel’s Place, high interior coppice, black light, VIII.17.1987, J. Browne (CMNC). **Cat Island.** Arthurs Town, W.J. Clench (MCZC): VII.7.1935; VIII.4.1935; VII-VIII.1935. **Darby Island.** I.18.1953, L. Giovannoli (AMNH). **Eleuthera Island.** The Bluff, W.M. Mann (USNM). **Grand Bahama.** 8 Mile Rock, IV.16.1936, Clench (MCZC). **Great Exuma.** Simons Pt., 23 31 50, 75 47 30, I.12.1980, S.A. Teale (SEMC). **Long Island.** Clarence Town, III.13.1953, L. Giovannoli (AMNH). Simm, VIII.8.1936 (MCZC). **Mayaguana Island.** UV trap, C.M. Murvosh (FSCA): VIII.3.1963; VIII.10.1963; VIII.24.1963; VIII.25.1963; VIII.28.1963. **New Providence Island.** VII.1904, Barber (MCZC). Nassau: XII.25.1898, A. Busck (USNM); IV.16.1953, E.B. Hayden (AMNH). Nassau, UV trap, IV.24.1972, F.D. Bennett (FSCA). **South Bimini Island.** VII.1951, C. & P. Vaurie (AMNH). **TURKS & CAICOS. Middle Caicos.** uv light, VII.20.1993, A. Swann (FSCA). **North Caicos.** Zambarra, XII.4-11.1993, B.M. Riggs (FSCA). **GREATER ANTILLES. Caymans. Grand Cayman.** uv light, P. Fitzgerald (FSCA): VII.9.1987; VII.11.1987; VI.1992. uv light trap, P. Fitzgerald (FSCA): VII.26.1982; 16232, XI.29.1992. Boatswain Point, XII.16.1985, E.J. Gerberg (FSCA). Boatswain Point, Lime Tree Estate, E.J. Gerberg (FSCA): II.16.1985; II.27.1987. Boatswain Point, Lime Tree Estate, E.J. Gerberg (UASM): IX.20.1983; XII.16.1985. Botanic Garden, uv light, VI.4.2008, M.C. Thoams, R.H. Turnbow, B.K. Dozier (FSCA). Georgetown, IX.16.1973, E.J. Gerberg (FSCA). **Little Cayman.** Pirates’ Point, MV light, VII.18.1975, R.R. Askew (BMNH). **Cuba**. (AMNH), (DEFW), (HNHM). **Camaguey.** Baragua, at light, C.F. Stahl: X.5.1925 (MCZC); X.6.1925 (USNM); X.6.1925 (MCZC); X.23.1925 (USNM). Baragua, at light, Christenson (USNM): V.24.1932; V.26.1932; V.29.1932; VI.1.1932; VI.4.1932; VI.5.1932; VI.11.1932; VI.14.1932. Baragua, in soil, X.28.1930, L.D. Christenson (MCZC). Galbis, V.8.1920 (CUIC). **Cienfuegos.** Cayamas, E.A. Schwarz (USNM): I.4; II.26; V.6; V.31; XII.29. Central Constancia, V.1914, J.F. Merrill (USNM). San Antonio, IV.9.1905 G. Dimmock (USNM). San Ant. de los Banos, VI.1973, L.F. Armas (IZAC). Soledad, G. Salt (MCZC): VI.3.1925; VI.9.1925. Soledad, VI-VII.1947, W.L. Nutting (MCZC). Central Soledad, VII.1.1932, B.B. Leavitt (MCZC). Soledad, nr. Cienfuegos, Darlington (MCZC): X.18.1926; X.25.1926; X.26.1926; X.27.1926; XI.4.1926; XI.13.1926; VI.1929; VIII.2-12.1934; IV.1936; V.1936. Soledad, nr. Cienfuegos (MCZC): V-VI.1939, Parsons; VII.20.1956, C. & P. Vaurie; VIII.6-20, N.A. Weber. Soledad, nr. Cienfuegos, big flood, X.21.1926, Darlington (MCZC). Soledad, nr. Cienfuegos, swampy pond by S. Anton, XI.13.1926, Darlington (MCZC). Soledad, Bates & Fairchild: VI.27.1932 (MCZC); VII.23.1932 (UASM). Soledad, 21.12682 -80.33289, mv light, 71 m, V.21.2013, 2013-029X, R.S. Anderson (CMNC). Trinidad Mtns., Mina Carlota, VII.1939, Parsons (MCZC). Trinidad Mtns., 182.9-609.6 m, VI.1929, Darlington (MCZC). **Guantanamo.** Guantanamo, X.15.1934, M.M. Saylor (CASC). Guantanamo Bay, mosquito light trap, IV.3.1970, J.E. Tisdale (FSCA). Guantanamo Bay, Beach Housing Area, UV trap, S. Calhoun (FSCA): III.24-27.1972; VIII.21.1972. Guantanamo Bay, Center Bargo, uv, S. Calhoun (FSCA): VI.19-22.1972; VIII.3.1972. Guantanamo Bay, Center Bargo, New Jersey trap, VIII.3.1972, S. Calhoun (FSCA). Guantanamo Bay Navy Base, Kittery Housing Area, uv, S. Calhoun (FSCA): V.8-11.1972; V.15-18.1972; VII.24-25.1972. Guantanamo U.S. Navy Base, uv, IX.3.1964, T.S. Josey (FSCA). Guantanamo Bay, Navy Base, Caravella Pt., UV trap, S. Calhoun (FSCA): VI.24-25.1972; IX.12.1972; IX.20.1972; IX.26.1972; IX.27.1972; IX.28.1972; X.2.1972; X.10.1972; X.17.1972; X.19.1972; X.23.1972; X.27.1972; X.30.1972; X.31.1972; XI.6.1972; XI.13.1972; XI.14.1972; XI.16.1972; XI.21.1972; XII.14.1972; III.29.1973. Guantanamo U.S. Navy Base, uv, IX.3.1964, T.S. Josey (FSCA). **Havana.** Almendares, V.7.1932 (IZAC). Almendares (MCZC): VI.14.1932, Jaume; VI.24.1932; VII.10.1932. Bocade Haruco, VI.7.1958, J. Schwartz (FSCA). Cojimar, 1913, T. Barbour, L.A. Shaw (MCZC). Guanajay, Palmer & Riley (USNM): IV.25; V.1. La Habana, V.1974, E. & Z. Meszaros (UASM). Havana (MCZC); XI.5-XI.6.1915 (AMNH); V.1974, E. & Z. Meszaros (HNHM); VII.5.1974, E. & Z. Meszaros (HNHM); Baker (CMNH); Baker (USNM); F.C. Bowditch (MCZC); vic. of Havana, M.T. Cook (AMNH): V.24.1905; V.27.1905; V.31.1905; VI.2.1905. vic. of Havana,T. Barbour (MCZC). Havana, University Hill, XI.9.1915 (AMNH). Jamaica ad San Hose de las Lajas, II.22.1967, R. Bielawski, A. Riedel (IZWP). La Chapa, Quivicon, IX.11.1996, F. Fonseca (PVRC). Laguna Somorrostro: VII.8.1956 (IZAC); VIII.1964 (MNHC). Litoral de Marianao: VII.1950 (IZAC); VI.1932, M. Barro (MNHC). Melena del Sur, Campo Boniato, VII.16.1997, E. Fonseca (PVRC). Playa Baracoa (IZAC): X.1953, M.L. Jaume; VII.3.1954. Santa Maria del Mar ad Habana, I.7.1967, R. Bielawski, A. Riedel (IZWP). Santiago-Vegas, IV.15.1905, G. Dimmock (USNM). **Holguin.** Coletones, Gibara, luz, II.16.1998, P. & A. (PVRC). Holguin, XII.1929, Aguayo (MCZC). **Isla de la Juventud.** XI.15, Oertel [?spelling] (USNM). Nueva Gerona, G. Link (CMNH): V.31.1912; III.13.1913; III.1913. **Matanzas.** Playa Larga, Cienaga Zapate, luz, P. & A. (PVRC): VI.18.1996; VI.20.1996; VI.23.1996. Playa Larga, Cienaga Zapate, luz, IV.1997, P. Valdez R. (PVRC). **Pinar del Rio.** Guanahacabibes Pen., VII.3-4.1956, C. & P. Vaurie (MCZC). Guane: VII.6.1933, S.T. Danforth (MCZC); VII.6-10.1933, H.J. MacGillavry (ZMAN). Pinar del Rio (MCZC). Pinar del Rio, at light, V.16-29.1933, H.J. MacGillavry (ZMAN). Lomas de Soroa, V.1965 (IZAC). San Vicente, C. & P. Vaurie: VII.6-10.1956 (AMNH); VII.6-VII.10.1956 (MCZC); VII.25-28.1956 (MCZC). San Vicente, Vinales, VI.1963, Alayo & Garcia (IZAC). Soroa, III.9163, Alayo & Garcia (IZAC). **Santiago de Cuba.** Ponupo ad la Maya, II.11.1967, R. Bielawski, A. Riedel (IZWP). Puerto Boniato ad Santiago de Cuba, II.10.1967, R. Bielawski, A. Riedel (IZWP). Santiago de Cuba, II.11.1967, R. Bielawski, A. Riedel (IZWP). **Villa Clara.** north end of Lago del Hanabanilla, VII.1.1990, J. Rawlins & S. Thompson (CMNH). Marimon, V.1942, P. Alayo (IZAC). San Blas (MCZC): V-VI.1932; (1932) Vueltas, VII.10.1963, L.V. (IZAC). **Hispaniola: Dominican Republic.** 1963, T. Morales, C. Rodrigues (USNM). **Barahona.** Barahona, IX.1938, Darlington (MCZC). 4.5 km S & 5 km W Barahona, mv & bl l, V.17.1992, R. Turnbow (UASM). nr. Filipinas, Larimar Mine, R.E. Woodruff, P.E. Skelley (FSCA): at light, VI.20-26.1992; at light, VI.26-VII.7.1992; hand catch, VI.20-26.1992. 6 km NW Paraiso, Rio Nizao, 170 m, VII.25-26.1990, Rawlins, Young & Thompson (CMNH). 6 km NWParaiso, Rio Nizao, 170 m, 18°02'N, 71°12'W, VII.25-26.1990, Thompson, Young, Rawlins (CMNH). 9.2 km NW Paraiso, confluence of Rio Nizao and Rio Coltico, 230 m, VIII.9-10.1990, J. Rawlins, S. Thompson (CMNH). **Dajabon.** 9 km S Loma de Cabrera, 620 m, 19°21'N, 71°37'W, disturbed pastures in mesic woodland, VII.12.1992, C. Young, S. Thompson et al (CMNH). **Distrito Nacional.** Sosua, Clench (MCZC). **Elias Pina.** SFrncsco Mts., IX.1905, A. Busck (USNM). **Espaillat.** Moca, Ciferri (MCSN): 1926; V.1927; VI.1927; VIII.1927; X.1927; 1928; V.1929. **Hato Mayor.** W Sabana de la Mar, Par. Nac. Los Haitises, 19°03'N, 69°24'W, uv light, 5 m, VII.17.1993, D.S. Sikes (WIBF). **Independcia.** 4 km S Los Pinos, Loma de Vientos, 18°35'N, 71°46'W, semiarid deciduous forest with pastures, C. Young, S. Thompson et al (CMNH): 455 m, VII.23.1992; 475 m, X.12.1991. **La Altagracia.** 2 km N Bayahibe, 10 m, 18°23'N, 6851'W, dry seasonal forest on limestone, VII.3.1992, C. Young, S. Thompson et al (CMNH). Nisibon, uv, IX.24.1985, Stange, Woodruff & House (FSCA). Nisibon Papagallo, blacklight trap (FSCA): VI.16-19.1998, R.E. Woodruff, P.H. Freytag; VI.16-19.1999, R.E. Woodruff, R.M. Baranowski; VI.24.1998, R.E. Woodruff. vic. Oyo Clara, 18°33'48"N, 68°26'50"W, uv light, VII.24-VIII.5.2002, K.W. Will, C. Chaboo (EMEC). P.N. del Este, Boca Yuma, 18°21.904'N, 68°37.087'W, entrance, at light, beating vegetation, VIII.25.1999, M.A. Ivie (WIBF). Parque Nacional del Este, Guaraguao, light trap, V.30.1992, K. Guerrero (WIBF). Punta Cana Resort, 18°30'16"N, 68°22'37"W, at light, VII.24-VIII.5.2002, K.W. Will, C. Chaboo (EMEC). **La Romana.** Higueral, uv, Folch & Woodruff (FSCA): VIII.14.1977; VIII.15.1977; VIII.16.1977; VIII.18.1977; VIII.19.1977. Higueral, UV trap, VIII.17.1977, R.E. Woodruff (FSCA). La Romana, uv, R.E. Woodruff: VIII.22.1977 (FSCA), (UASM); IX.1976 (FSCA); IX.17.1976 (FSCA), (UASM); IX.18.1976 (FSCA); IX.21.1976 (FSCA), (UASM); IX.22.1976 (FSCA), (UASM); IX.29.1976 (FSCA), (UASM). La Romana, uv, IX.1976, R.E. Woodruff (UASM). La Romana, uv light, VIII.16.1977, Folch & Woodruff (UASM). La Romana, UV trap, E. Folch: IX.13.1976 (FSCA); IX.16.1976 (FSCA), (UASM). **La Vega.** Constanza, blacklight, XI.8.1984, P. Spangler, R. Faitoute (USNM). La Vega, 3 km W Manabao, VII.18.1996, M.C. Thomas (FSCA). **Monsenor Nouel.** Bonao, VI.1927, Ciferri (MCSN). **Monte Cristi.** 5 km NNE Bontoncillo, 50 m, 19°45'N, 71°42'W, arid thornscrub, XI.29-X30.1992, Davidson & Klingler (CMNH). **Pedernales.** Cabo Rojo, D.S. Sikes, R.P. Rosenfeld (WIBF): uv light, VII.8.1993; sweeping pool, VII.9.1993. Cabo Rojo, 17°55'N, 71°39'W, 10 m, VII.27.1990, Young, Rawlins & Thompson (CMNH). Cabo Rojo, 10 m, VII.27.1990, Rawlins, Young & Thompson (CMNH). Cabo Rojo, 10 m, 17°55'N, 71°39'W, coastal desert, C. Young, S. Thompson et al (CMNH): X.1.1991; X.19-23.1991; and brackish tidal flats, VII.15-VII.18.1992. Cabo Rojo, at light, 0-10 m, M. Ivie, Philips, Johnson (WIBF): VIII.24.1988; IX.8-9.1988. Cabo Rojo, at light, V.20.1992, R. Turnbow (UASM). Cabo Rojo, in pool & at light, 0-10 m, M. Ivie, Philips, Johnson (WIBF): VIII.18-23.1988; VIII.24-28.1988. Cabo Rojo, hotel, VIII.21.1992, D. Sikes, J. Brodzinsky (WIBF). Cabo Rojo, coast thorn scrub, swim pool surface, 10 m, XI.28-XII.2.1991, 91-362, Masner & Peck (CMNC). Cabo Rojo, Alcoa, blacklight trap, VII.11998, R.E. Woodruff, R.M. Baranowski (FSCA). Cabo Rojo, Alcoa Headquarters, blacklight trap (FSCA): VI.10.1998, R.E. Woodruff, R.M. Baranowski; VI.20-24.1999, Woodruff & Baranowski. 14.5 km N Cabo Rojo, 165 m, 18°03'N, 71°39'W, arid thornscrub, IX.26-27.1991, Davidson, Rawlins et al (CMNH). 14.5 km N Cabo Rojo, 165 m, 18°03'N, 71°39'W, VII.19.1990, Davidson, Rawlins, Thompson (CMNH). 17 km N Cabo Rojo, 255 m, 18°04'N, 71°39'W, dry deciduous forest, Davidson, Rawlins et al (CMNH): X.211991; X.211991. 21 km N Cabo Rojo, uv, VI.19.1976, R.E. Woodruff (FSCA). 23.5 km N Cabo Rojo, 540 m, 18°06'N, 71°38'W, wet deciduous forest, IX.26-27.1991, Davidson, Rawlins et al (CMNH). 23.5 km N Cabo Rojo, 540 m, VII.20.1990, Rawlins, Young & Thompson (CMNH). 23.5 km N Cabo Rojo, 18°06'N, 71°38'W, 540 m, VII.20.1990, Young, Rawlins & Thompson (CMNH). 2 km SE Cabo Rojo Rd toward Oviedo, in bat cave, VIII.24.1988, M. Ivie, Philips, Johnson (WIBF). along Rio Mulito, 13km N Pedernales, 230 m, 18°09'N, 71°46'W, riparian woodland, VII.17.1992, C. Young, S. Thompson et al (CMNH). **Puerto Plata.** Top of Pico Isabel de Torres nr Teleferrico, at light, VII.31.1999, K.A. Guerrero (WIBF). Puerto Plata, Hurst (MCZC). **Samana.** Samana Peninsula, 8 km S Las Galeras, Purta Balandra, 35 m, 19°11'N, 69°14'W, semiarid scrub for. on limestone, X.10.1991, Davidson, Rawlins et al (CMNH). Sanchez (AMNH): V.28-31.1915; VI.7-12.1915. **San Juan.** Rio Mijo, uv, V.20.1985, Woodruff & Stange (FSCA). **San Pedro de Macoris.** San P. de Macoris, VI.1941, J. Serralles (USNM). **Santiago.** Santiago: III.26.1956, S. del Rosario (MCZC); 1930, Ciferri (MCSN). **Santo Domingo.** Santo Domingo (MCZC); VIII.5.1967, L.H. Rolston (TAMU). Ca Duarte, K. 29, VII.3.1978, Chalumeau & Abud (IREC). **Hispaniola, Haiti. Artibonite.** Swamps N of Dessalines, IX.11.1934, Darlington (MCZC). Ennery, nr. 304.8 m, IX.6-11.1934, Darlington (MCZC). Plaine de l’Artibonite, IX.6.1934, Darlington (MCZC).**Centre.** Mt. Trou d’Eau, XI.19.1934, Darlington (MCZC). Poste Terre Rouge, 609.6 m, X.5.1934, Darlington (MCZC). **Nippes.** Grande Riviere, W.M. Mann (MCZC). Miragoane, X.30-XI.2.1934, Darlington (MCZC). **Nord-Est.** Le Trow [sic], X.2.1925, V.A. Hoffman (USNM). **Ouest.** Cape Haitien, W.M. Mann (MCZC). Port-au-Prince: V.1925, G.N. Wolcott (USNM); V.17.1934, M.M. Saylor (CASC), (USNM). vic. Port-au-Prince, E.M. Ducasse (MCZC). Port-au-Prince, Thor, UV trap, X.10-12.1970, J.E. Porter (FSCA). Port-au-Prince, Thor (suburb), P. Daniels res., uv, X.10-12.1970, J.E. Porter (FSCA). **Sud.** Etang Lachaux, S.W. Peninsula, under 304.8 m, X.26-27.1934, Darlington (MCZC). Petionville, 304.8 m, VII.5.1956, B. & B. Valentine (UASM). **Jamaica. Clarendon Parish.** Jackson Bay, XII.14.1974, G.R. Proctor (IJSM). Milk River Bath, UV trap, VI.19.1970, E.G. Farnsorth (FSCA). Milk River Bath, uv light, R.E. Woodruff (UASM): XI.19.1968; V.14.1969. **Manchester Parish.** Mandeville, in house, VI.16.1969 (UASM). Mandeville, uv trap, J.H. Frank (UASM): X.3-5.1969; III.26-27.1970; XI.24-26.1970. Mandeville, uv light trap, J.H. Frank (UASM): VI.4-5.1970; VII.16-18.1970. **St. Ann Parish.** Runaway Bay, UV trap, VII.12.1970, E.G. Farnsworth (FSCA). **Portland Parish.** Green Hills, X.1950, I.K. Sibley (IJSM). Hardwar Gap, 1219.2 m, VII.2.1966, Howden & Becker (CNCI). Milk River Bath, uv, R.E. Woodruff (FSCA): XI.19.1968; V.14.1969. Pt. Antonio, III.20, F.C. Bowditch (MCZC). **St. Andrew Parish.** Bamboo Lodge, nr. Irish Town, 91.4 m, UV trap, VII.23.1972, R.M. Baranowski (FSCA). Gordon Town, A. V.d.Porten (IJSM): X.15.1949; VI.10.1950; VII.3.1950. Gordon Town, VIII.1968, D. Bruce (IJSM). Half-Way Tree, I.28.1937, Chapman & Blackwelder (USNM). Half-Way Tree, R.P. Bengry (IJSM): IX.3.1950; IX.23.1950; X.10.1951. Half-Way Tree, at light, IX.14.1950, R.P. Bengry (IJSM). Irish Town (IJSM): XI.1949, G.L. Thynne; X.22-X.24.1951, H.K. Henry. Kingston: VIII.27.1934, Darlington (MCZC); XI.1952, G.R. Proctor (IJSM). Kingston, at light (CMNH). Kingston Botanic Gardens, under log, III.15.1969, J.H. Frank (UASM). Kingston, Mona Hotel, uv, X.19.1971, R.M. Baranowski (FSCA), (UASM). Kingston, Raetown (CMNH). Liguanea, at light, I.2.1931, M. Kisliuk (USNM). Liguanea, nr Kingston, VI.9.1931, M. Kisliuk (USNM). Mona (IJSM): XII.18.1946, G.B. Thompson; IX.23.1950; I.11.1951. Palisadoes, VII.4.1948, D.E. Miller (UMMZ). Swallowfield, C.B. Lewis (IJSM): X.1950; X.27.1951. Swallowfield, R.P. Bengry (IJSM): XI.3.1950; VII.13.1953. Upper Mountain View, IX.28.1947, C.B. Lewis (MCZC), (IJSM). St. AnN's Bay, Windsor Hotel, III.20.1955, T.H. Farr (SMIJ). **St. Catherine Parish.** Bushy Park, Amity Hall, II.9.1947, G.B. Thompson (IJSM). Port Henderson, uv, VII.12.1970, E.G. Farnsworth (FSCA), (UASM). Rio Cobre, 5 mi. above Spanishtown, VIII.29.1934, Darlington (MCZC). Spanish Town, XII. 21.1984, Vaicher (UASM). Spanish Town, uv, E.G. Farnsworth (FSCA), (UASM): VII.10.1970; VII.15.1970; VIII.5.1970. Spanish Town, Jamaica School Agr., uv, E.G. Farnsworth: V.3.1970 (FSCA); VI.14.1970 (FSCA), (UASM). Twickenham Park (UASM): uv light trap, V.8.1970, E.G. Farnworth; uv trap, III.24-25.1970, J.H. Frank. Worthy Park, UV trap, R.E. Woodruff (FSCA): XI.21.1968; V.11.1969. **St. Elizabeth Parish.** Balaclava, X.1892 (USNM). Black R., UV trap, VII.14.1970, E.G. Farnsworth (FSCA). Black River, at light, VI.7.1969, J.H. Frank (UASM). **St. James Parish.** Montego Bay, H.B. Southby (IJSM): IX.23.1950; X.25.1950; X.30.1950; V-VI.1951; II.15.1951; VII.15.1951; XII.15.1951. **St. Thomas Parish.** Yallahs, uv, VI.25.1970, E.G. Farnsworth (FSCA). **Trelawney Parish.** Duncans, Howden & Becker (CNCI): VIII.1.1966; VIII.10.1966; VIII.14.1966; VIII.16.1966; VIII.19.1966; VIII.21.1966; VIII.22.1966; VIII.23.1966. Vale Royal, uv trap, X.1-3.1969, R. Arscott (UASM). **Westmoreland Parish.** 0.8 km N Negril, nr. beach, uv, XII.10.1969, E.G. Farnsworth (FSCA), (UASM). Whitehouse Inn: VII. 18.1954, M.L. Farr (IJSM); II.9.1962, B. Heineman (AMNH). **Puerto Rico.** Desengano, XII.1923 (AMNH). Ensenada: VI.14-19.1915 (AMNH); III.21.1936, Darlington (MCZC). Guajataca, BSA Camp, at light, IV.1971, J. Maldonado (OSUC). Guanica, X.2.1913, E.J.S. (AMNH). L. Guanica, V.31.1938, Darlington (MCZC). La Plata, XII.18.1926, S.T. Danforth (AMNH), (MCZC). Mayaguez: X.1909, H. Aquebibes (MCZC); I.1916, R.T. Cotton (CUIC); II.1916, R.T. Cotton (CUIC); V.11.1917 (AMNH). Mercedita, X.10.1976, J. Micheli (UASM). Paguera, II.18 (AMNH). Ponce, VII.20-22.1914 (AMNH). Ponce, J. Micheli (USNM): III.4,1971; VIII.26.1971. Ponce, ‘By-Pass Hwy.’, IV.20.1971, J. Micheli (USNM). Ponce Rd. 132, km 20, under stones at night, IX.17.1976, J. Micheli (UASM). Quebradillas, 6.3 km SSE La Casada Piedra, E Side of Lage de Guajataca, 200 m, 18°22'24"N, 66°54'22"W, VI.151996, Davidson & Zano et al (CMNH). Rd. 10, Km. 24, in black light trap, IX.16-22.1977, J. Micheli (JMPR), (UASM). **Isla Maguey.** Parguera, XII.20.1962, P. & P. Spangler (USNM). **Swan Islands. Great Swan Island.** S. Nelson (MCZC). VIII.1971, G.R. Proctor (IJSM).

### *Selenophorus flavilabris cubanus* Darlington

**BAHAMAS. Andros Island.** black light, V-VIII.1987, J. Browne (CMNC). Mennenite’s Farm, crop, black light, VII.31.1987, J. Browne (CMNC). **GREATER ANTILLES. Cuba.** (HNHM). **Camaguey.** Baragua, at light, VII.27.1929, L.C. Scaramuzza (MCZC). Est. Exp. Agronomica, Camaguey, VI.1961, R. Hernandez (IZAC). Ciego de Avila. Cayo Coco, La Jaula, Bajo Cortez, X.14.1988, R. Regalado (MNHC). **Cienfuegos.** Cayamas, E.A. Schwarz (USNM). San Antonio, IV.7.1905, G. Dimmock (USNM). Soledad, 1925, G. Salt (MCZC). Central Soledad, VII.1.1932, B.B. Leavitt (MCZC). Soledad, Darlington (MCZC): X.21.1926; X.24.1926; VI.1929; VIII.2-12.1934; V.1936; VIII.1936. Soledad (MCZC): I.1927; I-II.1927, C.T. & B.B. Brues; V-VI.1939, Parsons; VIII.6-20, N. Banks. Soledad, berlese funnel, VIII.6-20, N.A. Weber (MCZC). Soledad, big flood, X.21.1926, Darlington (MCZC). Soledad, 21.12682 -80.33289, mv light, 71 m, V.21.2013, 2013-029X, R.S. Anderson (CMNC). Trinidad Mts., Buenos Aires, 762-1066.8 m, V.8-14.1936, Darlington (MCZC). **Granma.** Veguitas, W.M. Mann (USNM). **Guantanamo.** Guantanamo, III.29.1914 (AMNH). **Havana.** Ariguanabo, II.1962 (IZAC). Cojimarad, II.14.1967, R. Bielawski, A. Riedel (IZWP). Santa Maria del Mar ad Habana, I.7.1967, R. Bielawski, A. Riedel (IZWP). **Matanzas.** Playa Larga, Cienaga Zapate, luz, VI.18.1996, P. & A. (PVRC). Versalles, VII.2.1944, A.T. (IZAC). **Pinar del Rio.** Estacion El Taburete, Sierra Rosario, X.1998, P. Valdez (PVRC). Guane, VII.6.1933, S.T. Danforth (MCZC). San Vicente, VII.25-28.1956, C. & P. Vaurie (MCZC). Soroa, at light, VI.1964 (IZAC). **Santiago de Cuba.** Cuabitas, Stgo. de Cuba, VIII.1948, P. Alayo (IZAC). Santiago, J.M. Espin (USNM). coast below Pico Turquino, VI.26-30.1936, Darlington (MCZC). **Villa Clara.** Sabanas de Placetas, VIII.15.1939, J.P. Carabia (MCZC). Zapata, Playa Larga, I.28-29.1967, R. Bielawski, A. Riedel (IZWP).

### *Selenophorus flavilabris flavilabris* Dejean

**GREATER ANTILLES. Puerto Rico.** Castaner Finca, Adjuntas, II.5.1934, R.G. Oakler (USNM). Ensenada: VI.14-19.1915 (AMNH); III.21.1936, J.A. Ramos (MCZC). Guanica, X.2.1913, E.G.S. (MCZC). Jayuya, Bosque Estal del Toyo Negro, 0.7 km SE Cerro de Punta Cordillera Central, 1195 m, 18°10'09"N, 66°35'16"W, VI.9.1996, Rawlins, Young et al (CMNH). L. Guanica, V.31.1938, Darlington (MCZC). Lajas, XI.28.1929 (MCZC). Mandios, III.17.1906, Wheeler (AMNH). Mayaguez (MCZC): IX.15.1930, A. Suro; IX.19.1930, L. Martorell. Rd. 10, Km. 24, in black light trap, IX.23-29.1977, J. Micheli (JMPR). Rd. 365, Km. 7.4, in forest litter, X.1978, J. Micheli (JMPR). Toro Negro St. For., Cerro de Punta, N.E. side, 1000-1300 m, V.6.1985, Liebherr et al (CUIC). **LESSER ANTILLES. Anguilla.** West end, stn #052, VII.2.1973, P. Wagenaar Hummelinck (ZMAN). **St. Martin.** Cul de sac, II.3.1978, Chalumeau (IREC). Old Battery Hill, E of Great Ba, stn #461, V.18.1949, P. Wagonaar Hummelinck (ZMAN). Pic Paradis, V.2.1981, Chalumeau (IREC). SimpsoN's Bay: XII.23.1927 (AMNH); XII.23.1927 (MCZC).

### *Selenophorus flavilabris ubancus* Ball & Shpeley, stat. n.

**Not located.** Prickly Pear I., VIII.1908, M. Cameron (BMNH) (see Ball and Shpeley 1992: 105). **BAHAMAS. Mayaguana Island.** UV trap, C.M. Murvosh (FSCA): VIII.3.1963; VIII.24.1963; VIII.25.1963; VIII.28.1963. uv light trap, VIII.1963, C.M. Murvosh (UASM). **Rum Cay.** nr Port Nelson, III.16.1963, E.B. Hayden, L. Giovannoli (AMNH). **TURKS & CAICOS. North Caicos.** Kew, VI.29-30.1954, G.R. Proctor (CASC), (IJSM). **GREATER ANTILLES. Hispaniola: Dominican RepUBLIC. Azua.** east side of crest, Sierra Martin Garcia, 7 km WNW Barrero, 860 m, 18°21'N, 70°58'W, cloud forest nr. disturbed forest, VII.25-VII.261992, C. Young, S. Thompson et al (CMNH). **Barahona.** Barahona, IX.1938, Darlington (MCZC). nr. Filipinas, Larimar Mine, at light, VI.26-VII.7.1992, R.E. Woodruff, P.E. Skelley (FSCA). 2 km E Payoso, mv & bl, VII.13.1996, R. Turnbow (RHTC). **Dajabon.** 9 km S Loma de Cabrera, 620 m, 19°21'N, 71°37'W, disturbed pastures in mesic woodland, VII.12.1992, C. Young, S. Thompson et al (CMNH). **Hato Mayor.** W Sabana de la Mar, Par. Nac. Los Haiteses, 19°03'N, 69°24'W, uv light, 5 m, VII.17.1993, D.S. Sikes (WIBF). **Independcia.** 4 km S Los Pinos, Loma de Vientos, 455 m, 18°35'N, 71°46'W, semiarid deciduous forest with pastures, VII.23.1992, C. Young, S. Thompson et al (CMNH). ESE Jimani, S Lago Limon, 18°24'N, 71°44'W, uv light, 20 m, VII.3.1992, M.A. & R.O. Ivie (WIBF). **La Altagracia.** 2 km N Bayahibe, 10 m, 18°23'N, 68°51'W, dry seasonal forest on limestone, VII.3.1992, C. Young, S. Thompson et al (CMNH). vic. Oyo Clara, 18°33'48"N, 68°26'50"W, uv light, VII.24-VIII.5.2002, K.W. Will, C. Chaboo (EMEC). P.N. de Este, Boca de Yuma, 18°21.904'N, 68°37.094'W, at light, 2m, VIII.1999, M.A. Ivie, K.A. Guerrero (WIBF). Punta Cana Resort, 18°30'16"N, 68°22'37"W, at light, VII.24.VIII.5.2002, K.W. Will, C. Chaboo (EMEC). **La Romana.** La Romana, UV trap, IX.13.1976, E. Folch (FSCA). **La Vega.** Bamboo Hole Canyon, Rio Baiquare, 5 km SE Jarabacoa, 580 m, VII.22.1987, Davidson & Rawlins (CMNH). Constanza, 914.4-1219.2 m, VIII.1938, Darlington (MCZC). Constanza, 1524 m, VIII.1922, (CMNH). Constanza, Hotel Nueva Suiza, 1300 m, pine-hw. ravine, under rock, II.5.1975, W.L. & D.E. Brown (MCZC). Constanza to Jarabacoa, 609.6 m-1219.2 m, VIII.1938, Darlington (MCZC). Jarabacoa, 457.2-1219.2 m, VIII.1938, Darlington (MCZC). 2 km E Manabao, mv & bl, VII.18.1996, R. Turnbow (RHTC). 2.5 km SW Pinar Bonito, 1430 m, 1851'N, 70434'W, riparian vegetation near stream in pine woodland, XI.26.1992, C. Young, S. Thompson et al (CMNH). **Monte Cristi.** Monte Cristi, VI.21.1967, L.H. Rolston (TAMU). **Pedernales.** 1 km S Arroyos, 1125 m, 18°14'N, 71°45'W, second growth forest, X.181991, Davidson, Rawlins et al (CMNH). Cabo Rojo, in pool & at lights, 0-10 m, VIII.18-23.1988, M. Ivie, Philips, Johnson (WIBF). Cabo Rojo, 10 m, 17°55'N, 71°39'W, coastal desert, IX.26-271991, Davidson, Rawlins et al (CMNH). Km 24 N Cabo Rojo, 914 m., blacklight trap, VII.2.1998, R.E. Woodruff, R.M. Baranowski FSCA. Km 24 N Cabo Rojo, 914 m., blacklight trap, VI.23.1999, Woodruff & Baranowski (FSCA). 25 km N Cabo Rojo, mv & bl, VII.10.1996, R. Turnbow (RHTC). 25 km N Cabo Rojo, mercury vapor & ultraviolet light, 700 m, VII.10.1996, M.C. Thomas (FSCA). 26 km N Cabo Rojo, 7340 m, 18°06'N, 71°38'W, wet deciduous forest, IX.26-27.1991, Davidson, Rawlins et al (CMNH). 30 km N Cabo Rojo, 18°27.282'N, 71°35.483'W, uv & merc. vap. light, 1002.8 m, VII.21.1999, M.A. Ivie, G.O. Dominici (WIBF). ca. 35 km N Cabo Rojo, Las Abejas, 1250 m, FIT, VIII.26-IX.9.1988, M. Ivie, Philips, Johnson (WIBF). along Rio Mulito, 13km N Pedernales, 230 m, 18°09'N, 71°46'W, riparian woodland, VII.17.1992, C. Young, S. Thompson et al (CMNH). **Puerto Plata.** Puerto Plata (MCZC): Hurst; VII.20.1937, W.J. Clench. **Samana.** Sanchez, V.7.1927, A. Wetmore (USNM). **San Pedro de Macoris.** San Pedro, 13 km E Boca Chica, mv & bl l, V.27.1992, R. Turnbow (UASM). **Santiago.** Mt. Diego de Ocampo, 914.4-1219.2 m, VII.1938, Darlington (MCZC). San Jose de las Matas, 304.8-609.6 m, VI.1938, Darlington (MCZC). **Santo Domingo.** nr. Rio Haina, stn #031, V.3.1975, P. Wagenaar Hummelinck (ZMAN). **Hispaniola: Haiti.** (CUIC). **Artibonite.** swamps N of Dessalines, IX.11.1934, Darlington (MCZC). Ennery, nr 304.8 m, IX.6-11.1934, Darlington (MCZC). source Matelas, IX.5.1934, Darlington (MCZC). Mt. Basil, N. Haiti, 1432.6 m, IX.9.1934, Darlington (MCZC). St. Marc, WM Mann (MCZC). **Centre.** Mt. Trou d’Eau, XI.19.1934, Darlington (MCZC). Poste Terre Rouge, 609.6 m, X.5.1934, Darlington (MCZC). **Grand’Anse.** N.E. foothills, La Hotte, 609.6-1219.2 m, X.10-24.1934, Darlington (MCZC). **Nippes.** Grande Riviere, W.M. Mann (MCZC). LeBrun, nr Miragoane, in rotten log, I.2.1974, R.T.B.C. & Sette (RTBC). nr Miragoane, R.T. R.T.B.C. (RTBC): XII.30.1973; XII.31.1973. P. Riviere/Nippes, VII.13.1978, Chalumeau (IREC). Salagnac, Miragoane, VII.14.1978, Chalumeau (IREC). **Ouest.** Cape Haitien, W.M. Mann (MCZC). Carrefour, V.1-V.3.1908, M. Cameron (BMNH). Diquini, W.M. Mann (MCZC). Furcy, W.M. Mann (MCZC). Kenskoff, III.20.1940 (MCZC). Kenskoff, nr. P.-au-P., 1219.2-1828.8 m, Darlington (MCZC): IX.16.1934; IX.23.1934; XI.9.1934; XI.10.1934; XI.12.1934. 6.4 km N Kenscoff, 1066.8 m, VII.5.1956, B. & B. Valentine (UASM). **Sud.** Duchity, open rocky field, XII.20.1976, M. Langworthy (RLDC). Etang Lachaux, SW Peninsula, under 304.8 m, X.26-27.1934, Darlington (MCZC). Manneville, W.M. Mann (MCZC). Ravine du Sud, on ground, 1485 m, II.6.1984, S.R. Yocom (FSCA). Petion, W.M. Mann (MCZC). Petionville, W.M. Mann (MCZC). Port-au-Prince: V.17.1934, M.M. Saylor (CASC); V.28.1929, E.M. Ducasse (MCZC); VII.18-21.1955, A.F. Archer (MCZC); M. Cameron (BMNH). Port-au-Prince, dry ravine, XII.12.1976, M. Langworthy (RLDC). vic. Port-au-Prince, E.M. Ducasse (MCZC). **Sud-Est.** mts. N of Jacmel, W.M. Mann (MCZC). **Jamaica.** L.G. Perkins (MCZC), (BMNH). **Clarendon Parish.** Portland Cottage (label reads St. Catherine Parish), uv, VII.4.1970, E.G. Farnsworth (FSCA). Portland Ridge, PWD Fishing Club, uv, VIII.20.1969, Woodruff (FSCA). **Manchester Parish.** Mandeville, in house, V.16.1969 (UASM). Mandeville, uv trap, J.H. Frank (UASM): IX.19-21.1969; X.3-5.1969; X.18-18.1969. Mandeville, uv light trap; VI.4-5.1970, J.H. Frank (UASM). **Portland Parish.** Port Antonio (label reads Clarendon Parish), Bonnieview, at light, VII.16.1952, A.M. Laessle (FSCA). **St. Andrew Parish.** Constant Spring, IV.12-16.1931 (AMNH). Crossroads, VI.8.1950, R.P. Bengry (IJSM). Half-Way Tree, R.P. Bengry (IJSM): II.17.1947; IX.3.1950; X.10.1951. Half-Way Tree, I.28.1937, Chapin & Blackwelder (USNM). Kingston (MCZC). Kingston Botanic Gardens, under log, III.15.1969, J.H. Frank (UASM). Mona (IJSM): XII.18.1946, G.B. Thompson; IX.23.1950, M. Parry; I.11.1951, T. Parry. Mona, VIII.1953, E. Williams (MCZC). Swallowfield, C.B. Lewis (IJSM): X.1950; X.27.1951. Swallowfield, R.P. Bengry (IJSM): X.12.1951; VII.13.1953. Upper Mountain View, G.B. Thompson: X.1.1946 (MCZC); XII.15.1946 (IJSM). **St. Ann Parish.** Ocho Rio, VIII.20-24.1934, Darlington (MCZC). **St. Catherine Parish.** Above Rocks, VIII.8.1956, B. & B. Valentine (UASM). Bushy Park, Amity Hall, II.9.1947, G.B. Thompson (IJSM). Old Harbour, K. (CMNH). Rio Cobre, 8 km above Spanish Town, VIII.29.1934, Darlington (MCZC). Port Henderson, uv, VII.12.1970, E.G. Farnsworth (FSCA), (UASM). Spanish Town, uv, E.G. Farnsworth: VII.10.1970 (FSCA), (UASM); VII.15.1970 (FSCA); VIII.5.1970 (FSCA), (UASM). Spanish Town Jamaica School Agr., uv, VI.14.1970, E.G. Farnsworth (FSCA). Worthy Park, uv, VI.3.1970, E.G. Farnsworth (FSCA). Worthy Park, uv trap, R.E. Woodruff (FSCA): V.11.1969; VIII.28.1969. Worthy Park, VIII.21.1969, R.E. Woodruff (UASM). **St. Elizabeth Parish.** Balaclava, II.13.1937, Chapin & Blackwelder (USNM). Black River, at light, VI.7.1969, J.H. Frank (UASM). Quickstep, Forestry Hut, under stone, IV.11-12.1949, R.P. Bengry (IJSM). **St. James Parish.** Greenwood, II.12.1947, G.B. Thompson (MCZC), (IJSM). Montego Bay, X.30.1950, H.B. Southby (IJSM). **St. Thomas Parish.** Whitfield Hall, IV.13.1950, R.P. Bengry (MCZC). **Trelawny Parish.** Duncans, Howden & Becker (CNCI): VIII.1.1966; VIII.15.1966; VIII.19.1966; VIII.21.1966. Duncans, VIII.19.1966, Howden & Becker (UASM). 1.4 km W Duncans, 30 m, IX.3.1976, R.I. Crombie, F.I. McCullough (USNM). 5.6 km E Duncans, IX.3.1976, R.I. Crombie, F.I. McCullough (USNM). Falmouth, VII.19.1960, C. & P. Vaurie (AMNH). Good Hope, VIII.11.1966, H.F. Howden (CNCI). Nr. Troy, Cockpit Country, uv trap, VIII.26.1969, J.H. Frank (UASM). **not located.** nr 12 Mile Stone between Kingston & Cedar Valley, VI.25.1928, C.R. Orcutt (USNM).

### *Selenophorus integer* (Fabricius)

**GREATER ANTILLES. Hispaniola: Dominican Republic. La Altagracia.** Punta Cana Resort, 18°30'16"N, 68°22'37"W, at light, VII.24-VIII.5.2002, K.W. Will, C. Chaboo (EMEC). **Puerto Rico.** Bosque de Guanica, VI.9.2005, R. Brown, S. Lee (MEMU): 17°58'50"N, 66°52'37"W, 15W BL, sheet in dry coastal forest at Cobanus Rd.; 17°58'31"N, 66°52'45"W, 15W BL, box trap with ammonia, dry coastal forest at Ojo de Agua. Mayagues, Hacienda la Juanita, 2.8 km ENE Las Vegas, 18°11'45"N, 67°00'22"W, disturbed habitat, VI.10-11.1996, Young, Klingler, Zano (CMNH). Ponce Rd. 132, Km 20, under stones at night, IX.17.1976, J. Micheli (UASM). **St. Croix.** II.26.1925 (AMNH); III.4.1925 (AMNH); X.20.1979, D.F. Keaveny (WIBF); H.A. Beatty (WIBF), (MCZC), (USNM). Canaan, stn #617, at light, VI.22.1955, P. Wagenaar Hummelinck (ZMAN). Est. Betsy’s Jewel, IV.1993, Z. Hillis (WIBF). Estate Cotton garden, nr. S.E.T.I. Station, grass/xeric litter, I.11.1993, D.S. Sikes (WIBF). Est. Figtree, nr. Hess Refinery, at night, VIII-X.1993, B. Wilhelm (WIBF). Estate North Hall, Crege Gut, 30.5 m, XI.10.1992, M.A. Ivie (WIBF). Estate Sprat Hall, Cregue Dam Rd., 24.4 m, XI.7.1992, M.A. Ivie (WIBF). Frederiksted, Est. Sprat Hall, IV.1974, V. Yntema (WIBF). Judith’s Fancy, W.I. Knausenberger (WIBF): XII.2.1985; XII.2.1996. St. George Botanical Gardens, 17°43'N, 64°50'W, II.9.1996, W.E. Steiner, J.M. Swearingen (USNM). Two Williams, II.21.1966, M.B. Peace (USNM). **Virgin Islands. Great Camanoe**. (Upper) uv, X.7.2008, B.D. & S.C. Valentine (BDVC). **St. Thomas.** X.15.1978, M.A. Ivie (WIBF). A. Staercke (ZMAN). Est. St. Peter, 3-H-4 North Star, uv light, ca. 426.7 m, I.4-VI.30.1993, C. Mayes (WIBF). French Bay Estate, 228.6 m, VI.11.1979, M.A. Ivie (WIBF). south central coast, XII.17.1978, R.B. Nard (CMNH). **Tortola Island.** Chalwell, at light, C. Petrovic (BDVC): III.2003; IX.2003 IX.2007. **LESSER ANTILLES. ANGUILLA.** The Valley, 18°13'00"N, 63°03'20"W, III.23.1992, W.E. Steiner, J.M. Swearingen (USNM). **ANTIGUA.** Christian Valley, bl trap, VIII.29.1991, FAO Insect Survey (CMNC). **Barbados.** D. & L. Stoner (AMNH): VI; VI.7. uv, M.M. Alam (FSCA), (UASM): VIII.23-25.1972; VIII.26-29.1972; VIII.31-IX.2.1972. **St. George.** Groves, uv, IX.5.1972, M.M. Alam (FSCA), (UASM). **St. Michael.** Cavehill, uv, M.M. Alam (FSCA), (UASM): VII.10-30.1972; VII.31-VIII.2.1972; VIII.3.1972; VIII.5-7.1972; VIII.8-10.1972; VIII.11-13.1972; VIII.14.1972; VIII.16-17.1972; VIII.18-20.1972; VIII.21-22.1972. Cavehill, uv, VIII.22-23.1972, M.M. Alam (UASM). Cavehill, uv light, VIII.11-13.1972, M.M. Alam (CMNC). **St. Thomas.** Edgehill, uv, M.M. Alam (FSCA): VII.10-30.1972; X.5-6.1972; X.8-9.1972; X.11-12.1972; X.22-23.1972; X.31-XI.1.1972; XI.1-2.1972; XI.5-6.1972; XI.17-18.1972. Edgehill, uv, M.M. Alam (FSCA), (UASM): X.1-2.1972; X.3-4.1972; X.12-13.1972; X.13-14.1972; X.14-15.1972; X.15-16.1972; X.16-17.1972; X.17-18.1972; X.20-21.1972; X.21-22.1972; X.30-31.1972. **Barbuda.** Codrington, 0-20 m, VII.1976, N.L.H. Krauss (AMNH). NW of Codrington Village, stn #603, VII.5.1955, P. Wagonaar Hummelinck (ZMAN). **Bequia Island.** H.H. Smith (BMNH). **Buck Island.** B.I. Reef Nat. Mon., A.C. Poponi (WIBF): uv light, X.2.1996; FIT, XI.22.1996. B.I. Reef Nat. Mon., Detrick’s Shack, uv light, VIII.23.1996, A.C. Poponi (WIBF). **Dominica.** Clarke Hall, J.F.G.C. & T.M. Clarke (USNM): VI.1-10.1964; I.15.1965. Clarke Hall, T.J. Spilman (USNM): VII.9.1964; IX.29.1964. Clarke Hall, under cow dung, V.24.1964, J.F.G.C. & T.M. Clarke (USNM). Clarke Hall Est., UV light, VIII.5.1965, D.M. Anderson (USNM). 4.8 km E Pont Casse, X.13-16.1966, A.B. Gurney (USNM). **Grenada.** Chantilly Est., windward side, H.H. Smith (BMNH). Mount Gay Est., leeward side, H.H. Smith (BMNH). **St. Andrew Parish.** Coubal, XII.16.1989, J. Telesford (CMNC). Mirabeau, Agr. Lab., light trap (CMNC): VI.18.1990, J. Telesford; VI.28.1990, J. Telesford; IX.11.1990, H. Thomas. Mirabeau Agr. Lab., uv light, R.E. Woodruff (FSCA): IX.24.1990; X.3.1990; X.6.1990; X.7.1990; X.13.1990. Mirabeau Agr. Lab., blacklight trap, IX.29.1990, R.E. Woodruff (FSCA). Pearls airport area, 457 m behind beach, III.8.1990, R.E. Woodruff (CMNC). Point Salinas, blacklight trap, VI.2.1987, R.E. Woodruff (CMNC). **St. John Parish.** Black Bay Agr. Sta., malaise trap, IX.21-27.1990, R.E Woodruff, A. Thomas, J. Telesford (FSCA). **Guadeloupe.** (HNHM). Basse Terre, 3.2 km W of St. Saurew, under rotten banana stems, VII.11.1970, J.H. Frank (USNM). Basse Terre, Trois Rivieres, Grande Anse, V.29.2002, J. Touroult (WIBF). Domaine Duclos, VI.24-28.1960, P. & C. Vaurie (AMNH). Portland (Moule), V.11.1975, F. Chalumeau (CMNH), (IREC). Pte Grde Vigie, I.12.1975, Chalumeau (IREC). St. Francois, XII.9.1982, Chalumeau (IREC). Vernou, Chalumeau (IREC): IV.23.1971; V.16.1972; VII.18.1971; VII.181972; VIII.10.1971. **Marie Galante.** Habitation Murat, near Grand-Bourg, under old oak lumber, IX.22.2013, J.-M. Lemaire (JMLC). **Martinique.** Anse a 11Ave, X.27.1981, Roguet (IREC). Caravelle, VIII.15.1973, Camb. (IREC). Fort de France, 180 m, VI.1944, H. Stehle (USNM). **MAYREAU.** 12°38.42'N, 61°23.56'W, cemetery, old field, uv trap, 72 m, 09-51, VIII.11.2009, S. Peck (CMNC). Saltwhistle Bay, 12°38.68'N, 61°03.95'W, salt flat, thorn scrub, uv trap, 0.5 m, 09-54, VIII.12.2009, S. Peck (CMNC). Station Hill, 12°38'N, 61°23'45"W, disturbed woodland, lights and gen. colln., 30 m, 08-85, VIII.11-15.2008, S. Peck, M. de Silva (CMNC). **Montserrat.** Cassava Ghaut, Beattie House, 16°45.908'N, 62°12.953'W, uv light, 192.6 m, VI.21-30.2002, M.A. Ivie (WIBF). Cassava Ghaut, 16°45.91'N, 62°12.95'W, light trap, 192.6 m, VI.14-21.2002, A. Krakower (WIBF). Riley’s Estate, Chalumeau (IREC): IV.21.1984; V.29.1982. Salem, IX.4.1975, J. Cooter (BMNH). **MUSTIQUE.** Rutland Bay, 12°53'N, 61°11'W, dry woodland, forest edge, uv trap, 5 m, 08-73, VII.29.2008, S. Peck, M. de Silva (CMNC). **SABA.** windward side, III.24.1986, R.S. Miller (WIBF). Mountain Road, at light, M. Gillet (WIBF): X.4.2010; X.19.2010. St. JohN's, floating in toilet bowl, XII.9.2011, M. Boken (MBCN). **St. Eustatius.** stn #297, III.18.1937, P. Wagenaar Hummelinck (ZMAN). **St. Kitts.** VI.1935, S.T. Danforth (MCZC). W Basseterre, Bottom Mattingly Heights, blacklight trap, IX.5-6.1991 (CMNC). W Basseterre, Bottom Mattingly Heights, blacklight trap, IX.14.1991, R.E. Woodruff (CMNC). W Basseterre, Bottom Mattingly Hts., blacklight trap, R.E. Woodruff (FSCA): VIII.21-25.1991; VIII.27-VIII.31.1991. Brimstone Hill, V.30.1937, Roys (MCZC). East side, V.26.1937, Roys (MCZC). **St. Lucia.** VII.15.1931, S.T. Danforth (MCZC). Anse La Raye, Anse Galet, 1 km SSW Anse La Raye, 13°56'N, 61°03'W, 50 m, VI.21-30.1991, J.E. Rawlins, S.A. Thompson (CMNH). Castries, uv, VI.19.1977, R.E. Woodruff (FSCA). Chassin, 13.9965°N 60.9195°W, trap site, uv light, 94 m (WIBF): V.17-23.2009, R.C. Winton, E.A. Ivie; V.31-VI.4.2009, R.C. Winton, C.A. Maier. Escap Community, 13.83242°N 60.89864°W, ridge, uv light, 46 m, V.1.2009, I.A. Foley (WIBF). Escap Community, 13.8324°N 60.8986°W, 46 m (WIBF): merc vapor, V.21.2009, R.C. Winton; at house, VI.1-16.2009, R.C. Winton et al; VI.15-22.2009, WIBF group. Ford d’Or Bay, XII.21.1975, Chalumeau (IREC). Gros Piton, 13.81026°N 60.06525°W, uv light trap, VI.9-15.2009, E.A. Ivie & C.A. Maier (WIBF). Quilles For. Res., La Porte, 13.8404°N 60.9741°W, trap site, uv light, 272 m, V.5-7.2009, I.A. Foley, R.C. Winton (WIBF). Thomazo, XII.20.1975, Chalumeau (IREC). **St. Martin.** Great Bay, XII.24.1927, S.T. Danforth (MCZC). St. Peter, Cul-de-Sac, stn #467, V.24.1949, P. Wagenaar Hummelinck (ZMAN). SimpsoN's Bay, XII.23.1927, S.T. Danforth (MCZC). **St. Vincent.** 1965, E. Kirby (RTBC); H.H. Smith (BMNH). South end, H.H. Smith (BMNH). **St. Andrew Parish.** Buccament, Emerald Valley Hotel, 13°12.0'N, 61°13.8'W, uv light trap, 20 m, 07-12, VI.10-20.2007, S. & J. Peck (CMNC). Buccament Bay, 13°11.5'N, 61°00'W (should read 61°16.0'W), estuary, uv light, 1 m, 07-22, VI.20.2007, S. & J. Peck (CMNC). E Layou, Emerald Valley Hotel, 13°12.0'N, 61°14.8'W, 20 m, VIII.27-29.2006, S. & J. Peck (CMNC): streamside, uv trap, 06-122; forest edge, uv, 06-123. **St. George Parish.** Cane Hall, Rick’s Apt., blacklight trap, R.E. Woodruff (FSCA): IX.15-16.1991; IX.26.1991. **St. Patrick Parish.** Monteiths, Vermont Valley, IX.28.1991, R.E. Woodruff (FSCA). Wallilabou Bay, 13°14.9'N, 61°16.2'W, hotel grounds, coastal grove, uv trap, 2 m, 06-113, VIII.18.2006, S. & J. Peck (CMNC).

### *Selenophorus opalinus* LeConte

**UNITED STATES. Florida.** Manatee County. Oneco (UASM): III.27.1954, G.E. Ball; IV.4.1954. St. Lucie County. Fort Pierce, IV.2.1954, H.E. Evans (UASM). Lakewood Park, uv light trap, VIII.25.1972, J.H. Frank (UASM). [**not mapped, north of map used**: Okaloosa County. Eglin Air Force Base, VI.10-VI.11.1998, D.J. Printiss (UASM).] **BAHAMAS. South Bimini Island.** VIII.17.1951, C. & P. Vaurie (AMNH).

### *Selenophorus propinquus* Putzeys

**BAHAMAS. Andros Island.** Maidenhair Coppice, BLT, VI.11.2004, M.C. Thomas (UASM). **GREATER ANTILLES. Hispaniola: Dominican Republic. Azua.** 8 km NE Padre Las Casas, Rio Las Cuevas, VIII.7.1990, J Rawlins & S Thompson (CMNH). **Baoruco.** Sierra de Neiba, Los Guineos on upper Rio Colorado, mesic riparian woodland, 630 m, VIII.11-12.1990, J. Rawlins, S. Thompson (CMNH). **Barahona.** nr. Filipinas, Larimar Mine, at light, VI.26-VII.7.1992, R.E. Woodruff, P.E. Skelley (FSCA). **Espaillat.** Moca, Ciferri (MCSN): 1926; VI.1927; X.1927; II.1928. **Independcia.** 4 km S Los Pinos, Loma de Vientos, 455 m, 18°35'N, 71°46'W, semiarid deciduous forest with pastures, VII.23.1992, C. Young, S. Thompson et al (CMNH). **La Altagracia.** Nisibon Finca Papagallo, blacklight, VI.16-19.1999, R.E. Woodruff, R.M. Baranowski (FSCA). Nisibon Papagallo, blacklight (FSCA): VI.16-19.1998, R.E. Woodruff, P.H. Freytag; VI.24.1998, R.E. Woodruff. **La Romana.** Higueral, UV trap, VIII.17.1977, R.E. Woodruff (FSCA). La Romana, uv, IX.16.1976, E. Folch (FSCA), (UASM). La Romana, uv, R.E. Woodruff: IX.1976 (FSCA); IX.17.1976 (FSCA), (UASM); IX.22.1976 (FSCA), (UASM). **La Vega.** ca. 5 km S Constanza, 1250 m, at light, pine-guava forest, VIII.31.1988, M. Ivie, Philips, Johnson (WIBF). 2 km E Manabao, VII.18.1996: mv & bl, R. Turnbow (RHTC); ultraviolet & mercury vapor light, M.C. Thomas, R. Turnbow (FSCA). 3 km W Manabao, VII.18.1996, M.C. Thomas (FSCA). near mouth Arroyo Los Dajaos, 5 km E Manabao, 140 m, 19°04'N, 70°45'W, riparian woodland, X.9.1991, C. Young, S. Thompson et al (CMNH). 2.5 km SW Pinar Bonito, 1430 m, 18-51N 70-43W, riparian vegetation near stream in pine woodland, XI.26.1992, C. Young, S. Thompson et al (CMNH). **Samana.** Sanchez, VI.7-12.1915 (AMNH). **San Cristobal.** Villa Altagracia, VII.1938, Darlington (MCZC). **Santo Domingo.** S. Domingo, IX.30.1966, L.H. Rolston (TAMU). nr. Rio Haina, stn #031, V.3.1973, P. Wagenaar Hummelinck (ZMAN). **Jamaica. Manchester Parish.** Mandeville, in house (UASM): VI.21.1969; VII.15.1969. Mandeville, uv trap, J.H. Frank (UASM): IX.24-26.1970; X.3-5.1969; X.18-19.1969; X.24-26.1970. Mandeville, UV light, VII.2.1970, J. Farradane (BMNH). Mandeville, uv light trap, J.H. Frank (UASM): IV.10-12.1970; V.22-24.1970; VI.4-5.1970; VI.18-20.1970; VII.16-18.1970; VIII.14-16.1970; VIII.28-29.1970; XI.19-21.1970. Newport, under stone, I.10.1969 (UASM): J.H.F.; J.H. Frank. **Portland Parish.** Springhill, Cedar Hunt Farm, at light, V.24.1969 (UASM). **St. Andrew Parish.** Bamboo Lodge, nr, Irish Town, 914.4 m, uv trap, R.M. Baranowski: VII.22.1972 (FSCA); VII.23.1972 (FSCA), (UASM). Gordon Town, VIII.1968, D. Bruce (IJSM). Irish Town, Howden & Becker (CNCI): VIII.24.1966; VIII.26.1966; VIII.27.1966; VIII.28.1966. Irish Town, VIII.25.1966, Howden & Becker (UASM). Kingston, Darlington (MCZC): VIII.27.1934; VIII.28.1934. Kingston: (AMNH); VII.25.1960, P. & C. Vaurie (AMNH). Kingston Botanic Gardens, under log, III.15.1969, J.H. Frank (UASM). Mona, Univ. College of W.I., XII.12.1949, C.B. Lewis (MCZC). **St. Ann Parish.** Discovery Bay, uv light trap, XII.13-17.1969, J.H. Frank (UASM). **St. Catherine Parish.** Linstead, uv, IV.4.1971, R.M. Baranowski (FSCA). **St. Elizabeth Parish.** Black River, at light, VI.7.1969, J.H. Frank (UASM). **Trelawny Parish.** Nr. Troy, Cockpit Couontry, V.16.1969 (UASM). Vale Ryal, uv trap, X.1-3.1969, R. Arscott (UASM). **not located.** 12 Mile Stone betw. Kingston & Cedar Valley, VI.25.1928, C.R. Orcutt (USNM). **Puerto Rico.** Arecibo, 4.1 km SSE San Pedro fields nr. Rio Grande de Arecibo 18°22'35"N, 66°41'01"W, VI.161996, Young, Klinger, Davidson et al (CMNH). Bosque de Guanica, VI.9.2005, R. Brown, S. Lee (MEMU): 17°58'50"N, 66°52'37"W, 15W BL, sheet in dry coastal forest at Cobanus Rd.; 17°58'31"N, 66°52'45"W, 15W BL, box trap with ammonia, dry coastal forest at Ojo de Agua. Cayey, II.24.1968, F. Fisk (OSUC). Coamo Springs (AMNH): XII.27.1914; I.10.1915. Coamo Springs, IV.28.1929, S.T. Danforth (MCZC). El Yunque Stn., Luquillo Forest, VII.10-16.1969, H. & A. Howden (UASM). Ensenada, III.31.1936, J.A. Ramos (MCZC). Ft. Buchanan, uv trap, IX.22.1977, A. Gillogty, H. Harlan (KSUO). Fortuna AES, F. Fisk (OSUC): I.5.1968; I.1968; III.1968. Guajataca, BSA Camp, at light, IV.1971, J. Maldonado (OSUC). Guanica, X.2.1913, E.A. Schwarz (AMNH). L. Guanica, V.31.1938, Darlington (MCZC). Isabella Expt. Stn., UV trap, VII.2.1972, R.E. Woodruff, A.E. Agostini (FSCA). Isabella, Bosque Estal de Guajataca Montanzas Aymamon 18°25'06"N, 66°37'55"W, forest, VI.14-15.1996, Rawlins, Zanol, Davidson et al (CMNH). La Parguera, VII.28.1969, H. & A. Howden (UASM). Mercedita, X.10.1976, J. Micheli (UASM). Mercedita, at lights, VI.12.1972, J. Micheli (USNM). Orocovis, Basque Estal del Torro Negro 1.2 km N Don Juana Cordillera Central 18°10'30"N, 66°29'33"W, VI.81996, Davidson, Rawlins, Young et al (CMNH). Ponce: VII.20-22.1914 (AMNH); VIII.23.1971, J. Micheli (USNM). Ponce, “By-Pass Hwy.”, at lights, J. Micheli (USNM): I.20.1972; IX.26.1974. Ponce Rd. 132, Km 20, under stones at night, IX.17.1976, J. Micheli (UASM). Quadradillas, I.2.1915 (AMNH). Rd. 10, Km 24, VIII. 6.1977, J. Micheli (JMPR). Rd. 10, Km 24, in black light trap, IX.16-22.1977, J. Micheli (JMPR), (UASM). Rio Piedras, at light, II.15.1934, C.G. Anderson (USNM). Rt. 132, Km 20, at lights, V.26.1972, J. Micheli (JMPR). San German, T.E. Rogers (FSCA): XI.1.1967; I.21.1968. Viegnes Island (AMNH): IX.25.1921; IV.28.1930. Villalba, VI.20.1930, C.G. Salazar (MCZC). **Isla Maguey.** Parguera, XII.20.1962, P. & P. Spangler (USNM). **St. Croix.** H.A. Beatty (WIBF), (MCZC). X.20.1965, M.B. Peace (USNM). Christiansted, Spring Gut, under trash, II.15.1983, J.A. Ynema (WIBF). slope above Christiansted, VII.28.1976, R.H. Pine (USNM). Creque Gut, above dam, from stream debris, IX.30.1987, M. Ivie (WIBF). East Hill, uv trap, II.21.1966, M.B. Peace (USNM). Est. Stoney Ground, Sandy Point, beating at night, I.10.1993, R.S. Miller (WIBF). Golden Grove, uv light, V.16.1980, D.F. Keaveny (UASM). Golden Grove, at u-v light, M. Ivie (WIBF): III.12.1980; V.2.1980; V.12.1980; V.16.1980; V.21.1980; VI.9.1980; VII.14.1980; X.1980; (1979) Golden Grove, at u-v light, IV.24.1980, D.F. Keaveny (WIBF). Judith’s Fancy, XII.2.1985, W.I. Knausenberger (WIBF). Kingshill, uv light, II.11-17.1966, P.J. Spangler (USNM). St. George Botanical Gardens, 17°43'N, 64°50'W, II.9.1996, W.E. Steiner, J.M. Searingen (USNM). stn #338, XI.29.1936, Blackwelder (USNM). **Virgin IsLANDS. Camanoe.** uv, B.D.V. family (BDVC): X.15.2006; X.19.2006. **Great Camanoe.** (Upper) uv, B.D.V. family (BDVC): X.8.2007; X.16.2006; X.6.2007; X.21.2007. uv, (BDVC): X.27.2008, B.D. & S.C. Valentine; X.5.2008, B. Valentine, D. Dennis. uv, B.D. & S.C. Valentine (BDVC): X.9.2008; X.11.2008; X.16.2008; X.21.2008. uv, X.17.2008, B.D. & S.C. Valentine (UASM). Cam Bay, leaf litter, 30.5 m, VII.11.1994, S. Bucklin, M. Becker (WIBF). **Guana Island.** VII.1-14.1984, S.E. & P.M. Miller (WIBF). 0-80 m, VII.5-23.1985, S.E. & P.M. Miller (WIBF). 0-80 m, VII.9-23.1987, S.E. Miller, V.O. Becker (USNM). at uv light, B.D. Valentine, S.C. Valentine-Cooper (BDVC): X.22.2003; X.4.2004; X.8.2004; X.13.2004; X.23.2004; X.24.2004. at uv light, X.13.2003, B. & B. Valentine (BDVC). uv, X.13.2004, B.D.V. & S.C. Valentine-Cooper (BDVC). The Flat, malaise, X.2008, S.C. Valentine-Cooper (BDVC). The Flat, malaise trap, X.12.2007, B.D.V. family (BDVC). **St. John.** Lameshur Bay-VIERS, at u-v light, III.8.1984, M. Ivie (WIBF). **St. Thomas.** FrenchmaN's Bay, I.19.1981, R.S. Miller (WIBF). South Central Coast, XII.171975, R.D. Ward (CMNH). **Tortola Island.** Chalwell, at light, XI.2003, C. Petrovic (BDVC). **LESSER ANTILLES. Anguilla.** The Farrington, IX.26-X.1.1986, E. Censky (CMNH). Sandy Ground to N. Shannon Hill and road to Salt Pond, 0-61 m, XI.8.1999, M.A. Ivie, J.B. Runyon (WIBF). **Antigua.** Christian Valley, bl trap, FAO Insect Survey (CMNC): V.9.1991; V.10-12.1991; V.13.1991; V.16.1991; V.23.1991; V.24-27.1991; VI.5.1991; VI.11-12.1991; VI.17.1991; VI.18.1991; VI.24-25.1991; VI.28-30.1991; VII.2.1991; VII.5-7.1991; VII.8.1991; VII.9.1991; VII.12-14.1991; VII.17.1991; VII.22-24.1991; VII.26.1991; VIII.19.1991; VIII.27.1991; VIII.29.1991; IX.10.1991; IX.11.1991; IX.13.1991; IX.17.1991; IX.19.1991; IX.23.1991; IX.24.1991; X.8.1991; X.29.1991; XI.14.1991. Christian Valley Agr. Stn., blacklight trap, R.E. Woodruff (CMNC): VIII.14.1991; IX.11.1991. Christian Valley Agr. Stn., malaise trap, IX.9-19.1991, R.E. Woodruff (CMNC). Christian Valley Agr. Stn., FAO Insect Survey, R.E. Woodruff (CMNC): uv light trap, IX.25.1991; blacklight trap, X.10.1991; uv light, X.18-21.1991. Christian Valley, Agr. Exp. Sta., blacklight trap, R.E. Woodruff (FSCA): IV.29.1991; V.23.1991; VI.14-16.1991; VII.18.1991. Friars Hill, stn #594, VII.16.1955, P. Wagenaar Hummelinck (ZMAN). St. JohN's, VIII.1967, N.L.H. Krauss (USNM). St. JohN's, 0-150 m, VII.1966, N.L.H. Krauss (AMNH). **Buck Island.** 17°48'N, 64°37'W, under leaf litter on sand at edge of ground cover vegetation on dry zone of upper beach, II.15.1996, W.E. Steiner, Z. Hillis, J. Swearingen, et al (USNM). B.I. Reef Nat. Mon., uv light, A.C. Poponi (WIBF): VIII.23.1996; IX.13.1996; X.2.1996. B.I. Reef Nat. Mon., Dedrick’s Shack, uv light, VIII.23.1996, A.C. Poponi (WIBF). **Dominica.** Antrim, 304.8 m, III.12.1956, J.F.G. Clarke (USNM). Clarke Hall, at light, III.10.1964, D.F. Bray (USNM). **Guadeloupe.** Anse Lagourde, IX.24.1972, Chalumeau (IREC). Domaine Duclos, VI.24-VI.28.1960, P. & C. Vaurie (AMNH). Basse-Terre, J. Touroult (WIBF): Mane à Vaches, at light, III.22.2001; Saint-Claude, Ducharmony, IV.5.2002; Matouba, St. Claude, VIII.8.2003. Grand E [?spelling] VII. 14.1970, Chalumeau (IREC). Matouba, 579.1 m, VI.29.1960, P. & C. Vaurie (AMNH). Portland (Moule), V.11.1975, F. Chalumeau (IREC). Prise d’Eau, VIII.22.1970, Chalumeau (IREC). St. Felix Gosia [?spelling], II.4.1974, Gysin (IREC). St. Francois, XII.9.1982, Chalumeau (IREC). Vernou, Chalumeau (IREC): III.15.1971; IV.23.1971; VI.12.1971; VII.22.1970; VIII.10.1971; IX.6.1970. Viard (P.B.), VII.14.1970, F. Chalumeau (IREC). **MARTINIQUE.** 1 km E Diamant, 14°28.7'N, 61°00.6'W, thorn forest, flight intercept, 10 m, 10-51, VII.7-28.2010, Peck (CMNC). **Monserrat.** Cassava Ghaut, Beattie House, 16°45.908'N, 62°12.953'W, 192.6 m, VI.21-30.2002, M.A. Ivie (WIBF). Cassava Ghaut, Beattie House, 16°45.91'N, 62°12.95'W, 192.6 m, VI.10-17.2003, K.A. Marske (WIBF). Cassava Ghaut, Beattie House, 16°45.91'N, 62°12.95'W, 192.6 m, A. Krakower (WIBF): uv light, II.5-16.2002; light trap, VI.14-21.2002. Cassava Ghaut, Beattie House, 16°45.91'N, 62°12.95'W, uv light, 192.6 m (WIBF): III.11-23.2002, A. Krakower; III.23-IV.3.2002, A. Krakower; V.30-VI.6.2002, A. Krakower; VI.21-30.2002, M.A. Ivie. Cassava Ghaut, Beattie House (WIBF): uv light, V.20.2003, K.A. Marske; uv light trap, VI.21-30.2002, M.A. Ivie. N of Cedar Ghaut, Furlong, 16°46.546'N, 62°10.338'W, 51.8 m, VIII.6-9.2005, WIBF grp (WIBF). Galway’s Soufriere P.L., XII.12.1983, Lalanne-Cassou (IREC). Old Towne, Palm Court, at house lights, V.4-VI.2.2002, K. Marske (WIBF). Olveston, 16°45'01"N, 62°13'52"W, malaise trap in garden, II.18-27.2001, M. Stevens (WIBF). Olveston, school tennis courts, at light, VII.7.2002, K.A. Marske (WIBF). Rendevouz Bay, 16°48.496'N, 62°12.298'W, uv light, sea level, VII.26-31.2005, WIBF grp (WIBF). Rendevouz Bay, 16°48.50'N, 62°12.30'W, uv light trap, WIBF grp (WIBF): VII.26-31.2005; VIII.8-9.2005. Rendevouz Bay, 16°48.50'N, 62°12.30'W, uv light, VIII.8.2005, I.A. Foley (WIBF). Woodands, 76.2 m, II.1959, G.R. Proctor (IJSM). Woodlands, 16°45.44'N, 62°13.13'W, malaise trap, III-IV.2001, M. Stevens, I. Ripuano (WIBF). Woodlands, Riverside House, 16°45.99'N, 62°13.34'W (WIBF): light, I.1-4.2002, M.A. Ivie, K. Marske, K. Puliafico; uv light, VII.25.2003, WIBF grp; uv light, VII.25.2005, I.A. Foley. Woodlands, Riverside House, 16°45.99'N, 62°13.34'W, M.A. Ivie, K. Marske, K. Puliafico (WIBF): I.1-4.2002; I.10-13.2002. Woodlands, Riverside House, 16°45.99'N, 62°13.344'W, uv light, VII.31.2005, I.A. Foley (WIBF). Woodlands, Riverside House, VII.25-29.2005, M.A. & L.L. Ivie (WIBF). **NEVIS.** Cotton Ground Village, blacklight trap, III.24-IV.2.1993, B. Brandy (CMNC). **SABA.** Dancing Place Trail,at light, V.25.2008, M.A. Ivie (WIBF). Dancing Place Trailhead, 17.6245°N 63.2371°W, roadside, headlamp, 21: 00-22: 00, 291 m, III.13.2008, J.A. Slowik (WIBF). **St. BarthÉlemy.** Anse Saline, IV.25.1974, Chalumeau (IREC). **St. Kitts.** Frigate Bay, IV.9.1953, L. Pastuel [?spelling] (WIBF). W Basseterre, Bottom Mattingly Hts., blacklight trap, VIII.21-25.1991, R.E. Woodruff (FSCA). W Basseterre, Bottom Mattingly Heights, blacklight, IX.5.1991, R.E. Woodruff (CMNC). W Basseterre, Flamenco Disco, blacklight trap, VIII.27.1991, R.E. Woodruff (CMNC). W Basseterre, Flamenco Disco, uv trap, VIII.30.1991, R.E. Woodruff (CMNC). Philips P.L., XII.6.1983, Lalanne-Cassou (IREC). St. Cristophe Molineux (Philips), IV.5.1980, Chalumeau (IREC). St. Cristophe Mt. Misery (Ouest), IV.4.1980, Chalumeau (IREC). **St. Martin.** Pic Paradis, V.2.1981, Chalumeau (IREC).

### *Selenophorus alternans* Dejean

**BAHAMAS.** V.-VIII.1987, J. Browne (CMNC). **Andros Island.** 8 km S on old logging rd, Gobi Leke, freshwater marsh community, black light, VII.9.1987, J. Browne (CMNC). **Mayaguana Island.** uv, C.M. Murvosh (FSCA): VIII.3.1963; VIII.10.1963; VIII.24.1963; VIII.25.1963; VIII.27.1963; VIII.28.1963. uv, C.M. Murvosh (UASM). Atlantic Missile Range, uv, VIII.30.1963, C.M. Murvosh (FSCA), (UASM). **New Providence Island.** Nassau (USNM). Nassau, at light, IV.16.1963, E.B. Hayden (AMNH). Nassau, uv trap, VI.24.1972, F.D. Bennett (FSCA). **TURKS & CAICOS. North Caicos Island.** uv light, VII.20.1993, A. Swann (FSCA). **GREATER ANTILLES. CUBA.** (MCZC). **Ciego de Avila.** Cayo Coco, La Juala, lux fria, IV.21.1988, E. Gutierrez (MNHC). **Pinal del Rio.** Guane, Danforth (MCZC): VII.5.1933; VII.6.1933. **HISPANIOLA: DOMINICAN REPUBLIC. Barahona.** Barahona,  IX.1938, Darlington (MCZC). **Distrito Nacional.** VIII.12.1967, L.H. Rolston (TAMU). S. Domingo, VIII.2.1966, L.H. Rolston (TAMU). **Independcia.** S of Lago Enriquillo, 18°24'N, 71°42'W, uv light, 20 m, VII.12.1993, D. Sikes, R. Rosenfeld (WBIF). 4 km S Los Pinos, Loma de Vientos, 18°35'N, 71°46'W, 455 m, semiarid forest with pastures, VII.251992, Davidson et al (CMNH). **La Altagracia.** vic. Oyo Clara, 18°33'48"N, 68°26'50"W, uv light, VII.24-VIII.5.2002, K.W. Will, C. Chaboo (EMEC). Parque Nacional del Este, Caseta de Boca de Yuma, 50 m, V.1992, K.A. Guerrero, F. del Monte (WBIF). Punta Cana Resort, 18°30'16"N, 68°22'37"W, at light, VII.24-VIII.5.2002, K.W. Will, C. Chaboo (EMEC). **La Romana.** Higueral, uv trap, VIII.17.1977, R.E. Woodruff (FSCA). La Romana, uv trap, IX.13.1976, E. Folch (FSCA). La Romana, uv trap in sugarcane field, IX.18.1976, E. Folch (FSCA). La Romana, uv, R.E. Woodruff (FSCA): IX.1976; IX.17.1976; IX.22.1976. uv, R.E. Woodruff (UASM): IX.22.1976; IX.29.1976. La Romana, uv, IX.16.1976, E. Folch (FSCA). **La Vega.** 4 km SE Jarabacoa, Rio Baiquate, 560 m., VII. 24.1987, R. Davidson (CMNH). **Monsenor Nouel.** Bonao, Ciferri (MCSN): V.1927; VI.1927. **Monte Cristi.** 5 km NNE Bontoncillo, 50 m, 19°45'N, 71°42'W, arid thornscrub, XI.29-30.1992, Davidson, Klingler (CMNH). 3 km N Villa Elisa, uv, Woodruff & Stange: X.1.1983 (FSCA), (UASM); X.1.1985 (FSCA). 5 km N Villa Elisa, V.10-18.1985, E. Giesbert (FSCA). **Pedernales.** Cabo Rojo, at light, 0-10 m, IX.10.1988, M. Ivie, Philips, Johnson (WIBF). Cabo Rojo, 17-55N 71-39W, 10 m, VII.27.1990, Rawlins, Young, Thompson (CMNH). Cabo Rojo, in pool & at lights, 0-10 m, M. Ivie, Philips, Johnson (WIBF): VIII.24-28.1988; VIII.28-29.1988; IX.8-9.1988. Cabo Rojo, in swimming pool, 10 m, VII.19.1987, J. Rawlins, R. Davidson (CMNH). Cabo Rojo, 10 m, 19°55'N, 71°39'W, coastal desert, IX.26-27.1991, C. Young, S. Thompson et al (CMNH). Cabo Rojo, sea level, 19°55'N, 71°39'W, edge of salt marsh, X.21.1992, C. Young, S. Thompson et al (CMNH). 1 km E Cabo Rojo, 17-55N 71-39W, 10 m, VII.30.1990, Rawlins, Young, Thompson (CMNH). 14.5 km N Cabo Rojo, 165 m, 18°03'N, 71°39"W, arid thornscrub, IX.26-27.1991, Davidson, Klingler et al (CMNH). 25 km N Cabo Rojo, mv & bl, VII.10.1996, R. Turnbow (RHTC). 25 km N Cabo Rojo, mv & bl, 700 m., VII.10.1996, M.C. Thomas (FSCA). Las Mercedes, 21 km N Cabo Rojo, 490 m, VII.10.1987, J. Rawlins, R. Davidson (CMNH). **Peravia.** S. Jose de Ocoa, VII.4.1978, Chalumeau & Abud (IREC). **Puerto Plata.** Los Hidalgos, VI.4-VI.5.1969, Flint & Gomez (USNM). Puerto Plata, VII. 20.1937, W.J. Clench (MCZC). **Samana.** Samana Pen., 8 km S Las Galeras, Punta Balandro, 35 m, 19°11'N, 69°14'W, semiarid scrub for. on limestone bluffs, X.101991, Davidson, Young et al (CMNH). **San Cristobal.** IX.3.1967, L.H. Rolston (TAMU). San Cristobal, VI.8-9.1969, Flint & Gomez (USNM). **Santiago.** Santiago, 1930, Ciferri (MCSN). **Santo Domingo.** Ca Duarte, Km 29, VII.3.1978, Chalumeau & Abud (IREC). Santo Domingo I., Sosua, Clench (MCZC). Santo Domingo I. (MCZC). **HISPANIOLA: HAITI. Ouest.** Gonave I., VII.18.1937, S.T. Danforth (AMNH), (MCZC). **PUERTO RICO.** Anasco, IX.3.1930, J. Landron (MCZC). Banos de Coamo, under cow dung, VII.30.1976, J. Micheli (JMPR). Caquas, “12-4-1912”, S.S. Crossman (AMNH), (MCZC). Bosque de Guanica, VI.9.2005, R. Brown, S. Lee (MEMU): 17°58'50"N, 66°52'37"W, 15W BL, sheet in dry coastal forest at Cobanus Rd.; 17°58'31"N, 66°52'45"W, 15W BL, box trap with ammonia, dry coastal forest at Ojo de Agua. Coamo Springs, IX.28.1929, S.T. Danforth (MCZC). El Yunque Stn., Luquillo Forest, VII.10-16.1969, H. & A. Howden (UASM). Ensenada, VI.14-19.1915 (AMNH). Ensenada, cowdung, IV.20.1936, H.L. Dozier (USNM). Florida, “VI-31”, Alsina (MCZC). Florida, VI.1931, S. Danforth (MNHC). Guanica, VII.26.1913, E.J.S. (AMNH), (MCZC). Guanica, I.1916 (CUIC). Humacao, M.A. Diaz (MCZC). Isabela, light trap, V.1948 (MCZC). Isabela Expt. Stn., UV trap, VII.2.1972, R.E. Woodruff, A.E. Agostini (FSCA). Isabela Expt. Stn., uv, VII.2.1972, Woodruff & Agostini (FSCA). Mameys, VI.27.1969, O.S. Flint Jr. (USNM). Manati, VII.10.1912 (USNM). Mayaguez, VIII.1920, G.N. Wolcott (USNM). Mayaguez (MCZC): XI.22.1930, M.A. Diaz; IV.1932, Cedo; VI.20.1932, Figarella; XI.1937, L. Morera. Mercedita, J. Micheli: V.3.1975 (JMPR); at lights, X.10.1976 (UASM); fluorescent light, VIII.26.1977 (UASM); fluorescent light, XI.6.1977 (JMPR). Ponce, at light, J. Micheli: IX.27.1974 (USNM); IV.22.1972 (JMPR); V.24.1977 (JMPR). Ponce, in black light trap, J. Micheli (JMPR): IX.17-23.1977; IX.24-30.1977. Ponce, Bypass Hwy., at light, J. Micheli (USNM): VII. 14.1971; IX.26.1974. Ponce, La Yuca, IV.15.1975, J. Micheli (UASM). Rio Piedra, IX.8.1931, Alsina (MCZC). Rio Piedras, at light, A.S. Mills (USNM): VI.14.1934; IX.12.1934. Rte. #132, Km 20, at light, VI.9.1971, J. Micheli (USNM). Sabanallono, XII.1930, Davila (MCZC). San Juan, at light, VI.23.1935, Anderson (USNM). San German, Reserva For. Maricao, Km 16.2 on Rte 120, at uv light, VIII.8.1999, P. Kovarik (WIBF). San Juan, Morro Castle, XII.28.1971, G.F. & S. Hevel (USNM). **Culebra Island.** III.14.1941, C. Parsons (MCZC). **Isla Mona.** VIII.11-31.1944, HA Beatty (MCZC). **Vieques Island.** IV.28.1930 (AMNH). **ST. CROIX.** H.A. Beatty (MCZC). Golden Grove, D.V. Keaveny: V.19.1982 (CASC); uv light, V.16.1980 (UASM); uv light, V.16.1980 (USNM). **VIRGIN ISLANDS. Camanoe.** uv, X.13.2006, B.D.V. family (BDVC). **Great Camanoe.** (Upper) uv, X.9.2007, B.D.V. family (BDVC). **Guana Island.** 0-80 m, VII.9-23.1987, S.E. Miller, V.O. Becker: (WIBF), (USNM). The Flat, uv, IX.30.2006, B.D.V. family (BDVC). **St. John.** Estate Carolina, NW of Coral Bay, at uv light, 76.2 m, VI.2.1982, W.B. Muchmore (WIBF). **LESSER ANTILLES. ANGUILLA.** Rendevous Bay, uv light, VIII.11-18.1993, R.M. & H.V. Baranowski (FSCA). **Barbados.** uv, VIII.23-25.1972, M.M. Alam (FSCA). **St. Michael.** Cavehill, uv, M.M. Alam: VII.1-30.1972 (FSCA); VII.31-VIII.2.1972 (FSCA); VIII.5-7.1972 (FSCA); VIII.8-10.1972 (FSCA); VIII.14.1972 (FSCA), (UASM); VIII.21-22.1972 (FSCA), (UASM); VIII.23-25.1972 (UASM); VIII.26-29 (UASM). **St. Thomas.** Edgehill, uv, M.M. Alam (FSCA): X.14-15.1972; X.16-17.1972; X.21-22.1972. **BEQUIA.** VI.1967, Badger (USNM). **GRENADA. St. Andrew Parish.** Mirabeau, Agr. Lab., light trap, III.27.1990, J. Telesford (CMNC). **St. George Parish.** Lance aux Epines, Coral Cove, 11°59.570'N, 61°45.277'W, sand beach, uv trap, 1 m, 10-73, VIII.16.2010, S. Peck (CMNC). Lance aux Epines, Coral Cove, 11°59.721'N, 61°45.480'W, thorn scrub, uv trap, 10 m, 10-75, VIII.18.2010, S. Peck (CMNC). Lance aux Epines, Coral Cove, 11°59.721'N, 61°45.480'W, thorn scrub, uv trap, 10 m, 10-77, VIII.27.2010, S. Peck (CMNC). Lance aux Epines, Coral Cove, 11°59.570'N, 61°45.277'W, sand beach, uv trap, 1 m, 10-78, VIII.28.2010, S. Peck (CMNC). Point Salinas, l. trap, VI.27.1987, R. Woodruff (CMNC). Point Salinas, at night, VI.27.1987, R. Woodruff (CMNC). **GUADELOUPE.** Deux-Mamelles, IV.17.1973, Chalumeau (IREC). St. Francois, V.28.1983, L. Cassou (IREC). **MARTINIQUE.** Le Prêcheur, X.20.2012, D. Romé (JMLC). **MAYREAU.** Saltwhistle Bay, 12°38'N, 61°23'45"W, Canash Pond, woodland, uv trap, 2 m, 08-86, VIII.11.2008, S. Peck, M. de Silva (CMNC). Station Hill, 12°38'N, 61°23'45"W, disturbed woodland, lights and gen. colln., 30 m, 08-85, VIII.11-15.2008, S. Peck, M. de Silva (CMNC). **MONTSERRAT.** St. Anthony, Plymouth, blacklight trap, VIII.8.1992, H.V. & R.M. Baranowski (CMNC). Woodlands Beach, at uv light, VIII.13.2005, I.A. Foley (WIBF). Woodlands, Riverside House, 16°45.99'N, 62°13.34'W, uv light, I.A. Fley (WIBF): VII.25.2005; VII.31.2005. Woodlands, Riverside House, 16°45.99'N, 62°13.34'W, VIII.20-23.2005, WIBF (WIBF). 1.6 km W Plymouth, jct. rd. to Fox’s Bay, uv trap, VI.25.1977, R.E. Woodruff (FSCA). **NEVIS.** VIII.22.1978, L.E. Syda (WIBF). **REDONDA.** 16°56.36'N, 62°20.75'W, 152.4-274.3 m, VIII.6.2005, M. Ivie, V. Martinson (WIBF). **SABA.** Mountain Road, light, X.15.2010, M. Gillet (WIBF). **ST. KITTS.** Basseterre, VIII.5.1931, S.T. Danforth (MCZC). Basseterre, at light, V.23.1937, Rays (MCZC). W Basseterre, Bottom Mattingly Hts., blacklight trap, VIII.21-25.1991, R.E. Woodruff (FSCA). W Basseterre, Bottom Mattingly Heights, blacklight, IX.14.1991, R.E. Woodruff (CMNC). W Basseterre, Flamenco Disco, blacklight trap, VIII.27.1991, R.E. Woodruff (CMNC). Needsmost (Needsmust), light trap, IX.12.1987, T. Blanchette (CMNC). **ST. LUCIA.** Castries, UV trap, VI.19.1977, R.E. Woodruff (FSCA), (UASM). Gros Islet, VI.25.1931, S.T. Danforth (MCZC). Union Agricultural Sta., IX.11.1986, Crop Protection Unit (CMNC). **ST. VINCENT. St. Andrew Parish.** Buccament Bay, 13°11.5'N, 61°00'W (should read 61°16.0'W), estuary, uv light, 1 m, 07-22, VI.20.2007, S. & J. Peck (CMNC). **St. George Parish.** Brighton Bay Village, 13°07.97'N, 61°10.06'W, streamside, uv traps, 1 m, 07-10, VI.8.2007, S. & J. Peck (CMNC). Cane Hall, Ricks Apt., light trap, X.1-5.1991, R.E. Woodruff (CMNC). Mesopotamia, on Cocos nucifera, “82-08-07” (FSCA). Montreal, V.3.1978, Porion (IREC). **St. Patrick Parish.** Wallilabou Bay, 13°14.9'N, 61°16.2'W, hotel grounds, coastal grove, uv trap, 2 m, S. & J. Peck (CMNC): 06-113, 18.VIII.2006; 06-118, 23.VIII.2006. **UNION ISLAND.** Chatham Bay, Water Rock Res., 12°36.18'N, 61°26.59'W, tall forest, 2 uv traps, 125 m, 09-64, VIII.16.2009, S. Peck (CMNC).

### *Selenophorus palliatus* (Fabricius)

**UNITED STATES. Florida.** Manatee County, Oneco (UASM): IV.3.1954, G.E. Ball; IV.4.1954. St. Lucie County. Fort Pierce, IV.2.1954, H.E. Evans (UASM). [**not mapped, north of map used**: Hernando County. Weeki Wachee (UASM): III.2.1955; V.19.1955.] [**not mapped, north of map used**: Wakulla County. Panacea, 11 km 2 on Rte 30, V.21.1986, H. & A. Howden (UASM).] **BAHAMAS. Grand Bahama. **Freeport, VI.20-27.1987, W.E. Steiner, M.J. & R. Molineaus (USNM). **Man-O-War Cay.** VIII.21.1971, H. & A. Howden (CNCI). **North Bimini Island.** P. & C. Vaurie (AMNH): VII. 24.1951; VII. 26.1951; VII. 29.1951; VIII.16.1951. Lerner Marine Lab (AMNH): IX.7.1947; X.6.1947, J.A. Oliver. **South Bimini Island.** V.1951, Cazier & Gertsch (AMNH), (MCZC). C. & P. Vaurie (MCZC): VIII.9.1951; VIII.15.1951.

### *Selenophorus pyritosus* Dejean

**BAHAMAS. Andros Island.** Mennenite’s Farm, crop, black light, VII.30.1987, J. Browne (CMNC). Stafford Crk, Doc Woodside’s Place, high coast coppice, black light, VII.26.1987, J. Browne (CMNC). **Eleuthera.** Governor’s Harbour, V.2.1936, Clench (MCZC). **Long Island.** Clarence Town, III.13.1953, L. Giovanioli (AMNH). **New Providence Island.** Clifton Bluff, 25°01'N, 77°33'W, IV.17.2007, W.E. Steiner, J.M. Swearingen (USNM). Nassau (MCZC), (USNM). Nassau, VIII.6.1936 (MCZC). Paradise Island, Cabbage Beach, 25°05'30"N, 77°19'W, on sand, under edge of leaf litter layer, shrub zone on dunes behind beach, II.24.2005, W.E. Steiner, J.M. Swearingen (USNM). **San Salvador.** Gerace Research Centre, 24°07'N, 74°26'W, coastal sandy scrub forest, uv light, VI.25.2005, W.E. Steiner, J.M. Swearingen (USNM). **GREATER ANTILLES. CAYMANS. Grand Cayman.** Boatswain Point, VI.21.1976, E.J. Gerberg (FSCA). Botanic Garden, VI.4.2008, M.C. Thomas, R.H. Turnbow, B.K. Dozier (FSCA). **Cayman Brac.** Major Donald Dr., 0.4 km E jct. Ashton Reid Dr., bl trap, V.25.2009, Thomas, Turnbow, Ball (FSCA). **CUBA.** (DEFW); III.4.1930 (USNM). **Camaguey.** Camaguey, VIII-IX.1933, J.M. Osorio (IZAC). Baragua, at light, Christenson (USNM): VI.11.1932; V.23.1932; V.24.1932; V.26.1932. **Cienfuegos.** Cayamas, E.A. Schwarz (USNM): II.28; V.7; V.20; V.25; V.27. Central Constancia, V.1914, J.F. Merrill (USNM). San Antonio, IV.1905, G. Dimmock (USNM). San Antonio de los Banos, J.H. Pazos (USNM). Soledad (MCZC): VI.7.1925, G. Salt; VIII.2-12.1934, Darlington; V-VI.1939, Parsons. Soledad, big flood, X.21.1926, Darlington (MCZC). Trinidad Mtns., Hanabanillo Falls, IV.30.1936, Darlington (MCZC). Trinidad Mtns., Mina Carlota (MCZC): III.20.1925, J.G. Myers; VII.1939, Parsons. **Granma.** Jiguani, 1913 (MCZC). **Guantanamo.** Guantanamo, 1918, W.M. Mann (USNM). Guantanamo U.S. Navy Base, uv, IX.3.1964, T.S. Josey (FSCA). Guantanamo Bay, mosquito trap, III.26.1970, J.E. Tisdale (FSCA). Guantanamo Bay, mosquito light trap, J.E. Tisdale (FSCA): III.24.1970; IV.3.1970. Guantanamo Bay, Beach Housing Area, uv trap, III.24-27.1972, S. Calhoun (FSCA). Guantanamo Bay, Center Bargo, uv, S. Calhoun (FSCA): VI.19-22.1972; VIII.3.1972. Guantanamo Bay Navy Base, Kittery Beach Housing Area, uv trap, VIII.21.1972, S. Calhoun (FSCA). Guantanamo Bay Navy Base, Kittery Beach Housing Area, uv, S. Calhoun (FSCA): V.8-11.1972; V.15-18.1972; VII.24-25.1972. Guantanamo Bay Navy Base, Kittery Housing Area, uv light, S. Calhoun (UASM). Guantanamo Bay Navy Base, Caravella Point, uv, S. Calhoun (FSCA): VII.24-25.1972; IX.6.1972; IX.18.1972; IX.26.1972; IX.27.1972; X.2.1972; X.10.1972; X.11.1972; X.17.1972; X.19.1972; X.23.1972; X.27.1972; X.30.1972; XI.6.1972; XI.8.1972; XI.13.1972; XI.16.1972; XII.14.1972. Maisi, Boca de Jauco, VI.29.1990, E. Gutierrez (MNHC). San Carlos Est., C.T. Ramsden (AMNH): V.6.1914; at light, XII.9.1914. **Havana.** Almendares: V.24.1932, Jaume (MCZC); III.1949, M.L. Jaume (IZAC). Bocade Haruco, VI.7.1958, J. Schwartz (FSCA). Guanajay, IV.25, Palmer & Riley (USNM). Havana: VII.18.1900, Palmer & Riley (USNM); XI.5-6.1915 (AMNH); XI.13.1926, Darlington (MCZC); XI.1955, N.L. Krauss (USNM). Havana, Baker (AMNH), (MCZC), (USNM). Havana (MCZC). Havana, E. & Z. Meszaros (HNHM): IV.1974; V.1974; VII.5.1974. Havana, stones, dry pasture, XI.13.1926, Darlington (MCZC). vic. Havana, V.27, M.T. Cook (AMNH). Havana, University Hall, XI.9.1915 (AMNH). La Habana, V.1974, E. & Z. Meszaros (UASM). Jibacoa, V.1962, F. Zayas (IZAC). Jibacoa, at light VI.1962, Zayas (IZAC). Melena del Sur, Campo Boniata, VII.16.1997, E. Fonzeca (PVRC). Santiago-Vegas, G. Dimmock (USNM): III.28.1905; IV.1905. Est. Exp. Agronomica, Stgo. de las Vegas, V.6.1969, I. Garcia (IZAC). **Holguin.** Calabezas, Aguayo, I.1932 (MCZC). Calatones, Gibara, luz, (PVRC): II.5.1998, A. Lozada, P. Valdez R.; II.16.1998, P&A; II.17.1998, P. Valdez R. **Isla de la Juventud.** III.10.1939, S.C.B. (IZAC). 20 km E Cocodrijo, IX.1976, L.F. Armas (IZAC). Cayo Piedras, XI.5.1955, G.E. Watson III (PMNH). Sierra de Casas ad Nueva Gerona, I.19.1967. R. Bielawski, A. Riedel (IZWP). **Matanzas.** Los Arabos, V.8.1953, M.L. Jaume (INHS). Playa Larga, Cienaga Zapate, luz (PVRC): VI.23.1996, P. & A.; IV.1997, P. Valdez R. Versalles, IX.2.1949, A.T. (IZAC). **Pinar del Rio.** Cabanas, V.17, Palmer & Riley (USNM). Cabanas, IX.5-8.1913 (AMNH). Guane, IX.24-26.1913 (AMNH). Guane, VII.6.1933, S.T. Danforth (MCZC). Pinar del Rio, V.16-29.1933, H.J. MacGillavry (ZMAN). Pinar del Rio, at light, V.16-29.1933, H.J. MacGillavry (ZMAN). El Veral, Guanacobibes, XI.1967, G. Agueros (IZAC). Guanabacabives, Veral, VII.1971, D. Alayo (IZAC). Lomas de Soroa, VI.1963, Alayo & Garcia (IZAC). Pica Pica, Sumidero, VIII.1966, Garrido & Aguam (IZAC). Puerto Esperanza, VI.29-VII.6.1933, H.J. MacGillavry (ZMAN).San Vicente, Vinales, VI.1964, I. Garcia (IZAC). Taco Taco, IV.1963, Alayo & Garcia (IZAC). 7 km N Vinales, IX.16-22.1913 (AMNH). **Santiago de Cuba.** Aguadores, VI.6.1936, Darlington (MCZC). Cerro Guayabal, III.15.1936, S.C. Bruner (MNHC). Sierra Maestra, Ocujal del Turquino, XI.8-9.1967, R. Bielawski, A. Riedel (IZWP). coast below Pico Turquino, VI.26-30.1936, Darlington (MCZC). **Villa Clara.** Marimon, V.1941, P. Alayo (IZAC) (label reads “Ote.”, most likely for Oriente). north end of Lago del Hanabanilla, VII.1.1990, J. Rawlins, S. Thompson (CMNH). **HISPANIOLA: DOMINICAN REPUBLIC. Pedernales.** Cabo Rojo, Alcoa Hdqtrs., blacklight trap, VI.20-24.1999, Woodruff & Baranowski (FSCA). 4 km W Oviedo, arid thorn forest, intercept traps, 10 m, XI.28-XII.4.1991, 91-344, Masner & Peck (CMNC). **JAMAICA. Clarendon Parish.** Milk River Bath, uv, XI.19.1968, R.E. Woodruff (FSCA). Mocho, beneath leaves, XI.15.1978, J. Simpson (UASM). Portland Cottage (label reads St. Catherine Parish), uv, VII.4.1970, E.G. Farnsworth (FSCA). **St. Andrew Parish.** Constant Spring, IV.12-16.1931 (AMNH). Cross Roads (IJSM): VI.8.1950, R.P. Bengry; VI.1941, C.B. Lewis. Gordon Town, VI.10.1950, A.V. Porten (IJSM). Halfway Tree, R.P. Bengry (IJSM): VII.3.1950; IX.14.1950. Hope River, V.26.1908, M. Cameron (BMNH). Hope Road, X.11.1983, D. East (IJSM). Kingston, Mona Hotel, uv, X.16.1971, R.M. Baranowski (FSCA). NE end of Kingston race course, VI.20.1955, G.R. Proctor (IJSM). Palisadoes, VII.4.1948, D.E. Miller (UMMZ). Upper Mtn. View, VI.6-10.1942, C.B. Lewis: (IJSM), (MCZC). **St. Ann Parish.** Runaway Bay, VII.12.1970, E.G. Farnsworth: blacklight trap (FSCA); uv (FSCA); uv light (UASM). **St. Catherine Parish.** Caymanas Estate, uv light trap, V.10.1970, J.H. Frank (UASM). Old Harbour, IX.4.1908, M. Cameron (BMNH). Port Henderson, uv, VII.12.1970, E.G. Farnsworth (FSCA). Rio Cobre, 8 km above Spanishtown, 1934, Darlington (MCZC). Spanish Town, uv, E.G. Farnsworth (FSCA): VII.10.1970; VIII.5.1970. Spanish Town, Jamaica School Agr., uv, VI.14.1970, EG Farnsworth (FSCA). Spanish Town, Jamaica School Agr., mosquito lite trap, VI.3.1970, E.G. Farnsworth (FSCA). Worthy Park, uv, VI.3.1970, E.G. Farnsworth (FSCA). **St. Elizabeth Parish.** Santa Cruz, IV.15.1937, Roys (MCZC). **St. Thomas Parish.** Yallahs, uv, VI.25.1970, E.G. Farnsworth (FSCA), (UASM). **Trelawny Parish.** 14.5 km W Falmouth, VIII.5.1969, J.H. Frank (UASM). Holland Estate, IX.2.1969, J.H. Frank (UASM). **Westmoreland Parish.** 0.8 km W Negril, nr. Beach, uv, XII.10.1969, E.G. Farnsworth: (FSCA), (UASM).

### *Selenophorus woodruffi* Ball & Shpeley

**LESSER ANTILLES. GRENADA. St. Andrew Parish.** Balthasar R., 1.6 km E Grenville, uv light, VI.13.1977, R.E. Woodruff, E.E. Grissell (FSCA). Mirabeau, Agr. Lab., l. trap, IX.14.1990, J. Telesford (CMNC). **St. George Parish.** Point Salinas, l. trap, VI.27.1987, R. Woodruff (CMNC). Point Salinas, at night, VI.27.1987, R.E. Woodruff (CMNC). Point Salinas, blacklight trap, VI.2.1987, R.E. Woodruff (CMNC). True Blue, at night, X.1.1990, R.E. Woodruff, A. Thomas (FSCA). **MAYREAU.** 12°38.55'N, 61°23.58'W, thorn scrub forest, malaise, 50 m, 09-55, VIII.13-27.2008, S. Peck (CMNC). 12°38.42'N, 61°23.56'W, cemetery, old field, uv trap, 72 m, 09-51, VIII.11.2009, S. Peck (CMNC). Saltwhistle Bay, 12°38.68'N, 61°03.95'W (should read 61°23.95'W), salt flat, thorn scrub, uv trap, 0.5 m, 09-54, VIII.12.2009, S. Peck (CMNC).

### *Selenophorus parumpunctatus* Dejean

**Not located.** “Barbican” [possibly in Kingston, Jamaica] XII. 28.1978, P. Johnson (UASM). **UNITED STATES. Florida.** Monroe County. Big Pine Key, WatsoN's Hammock, hardwood hammock edge, shrub beating, VI-1-7.1986, J. Browne (UASM). Big Pine Key, SW¼ S4, mangrove-hardwood transition, malaise, VI.1-30, 1986, S. & J. Peck (UASM). Big Pine Key, hardwood-mangrove, u-v light, VIII.26-IX.5.1986, S. & J. Peck (UASM). Big Pine Key, N end, mangrove-hardwood transition, u-v light, VII.1990, E. Peck (UASM). Long Key, VII.23.1948, L.D. Beamer (SEMC). **BAHAMAS. Andros Island.** Atala coppice, high interior coppice, black light, VII.25.1987, J. Browne (CMNC). Forfar Field Station, Stafford Creek, uv light in coastal coppice, M.C. Thomas (FSCA): VI.2.2001; VI.6.2001. Fresh Creek, V-VI.1917, W.M. Mann (AMNH). Fresh Crk, Andros Twn Androsia, high interior coppice, black light, VIII.5-6.1987, J. Browne (CMNC). Mangrove Cay (AMNH): V.25.1904, W.M. Wheeler; V-VI.1917, W.M. Mann. 11.3 km E Three Crks Point, wet pineland/savannah junction, black light, VII.26.1987, J. Browne (CMNC). **Cat Island.** Arthurs Town, VII-VIII.1935, W.J. Clench (MCZC). **Darby Island.** I.18.1953, E.B. Hayden, L. Giovannoli (AMNH). **Grand Bahama.** 8-Mile Rock, IV.16.1936, Clench (MCZC). Freeport, VI.20-27.1987, W.E. Steiner, M.J. & R. Molineaux (USNM). Pine Ridge, at light, V.13.1953, E.B. Hayden, G.B. Rabb (AMNH). **Great Inagua.** 19.3 km N Matthew Town, I.29.1953, L. Giovannoli (AMNH). **Long Island.** Clarence Town, III.13.1953, L. Giovannoli (AMNH). Hard Bargain, uv trap, VI.27.1972, F.D. Bennett (FSCA). **Mayaguana Island.** uv, C.M. Murvosh (FSCA): VIII.10.1963; VIII.24.1963; VIII.26.1963; VIII.27.1963; VIII.28.1963. **New Providence Island**. Coral Harbour, 24°59'N, 77°29'W, under leaf litter on sandy soil near beach, IV.15.2007, W.E. Steiner, J.M. Swearingen (USNM). Nassau, UV trap, VI.24.1972, F.D. Bennett (FSCA). **North Bimini Island.** VIII.16.1951, P. & C. Vaurie (AMNH). Lerner Marine Lab, X.6.1947, J.A. Oliver (AMNH). **Rum Cay.** nr Port Nelson, III.16.1953, L. Giovannoli (AMNH). **San Salvador.** Gerace Research Centre, 24°07'N, 74°26'W, II.20.2004, W.E. Steiner, J.M. Swearingen (USNM). Gerace Research Centre, 24°07'N, 74°26'W, scrub forest edge, at open catchment, at uv light, W.E. Steiner, J.M. Swearingen (USNM): VI.20.2003; II.16.2004. **South Bimini Island.** C. & P. Vaurie (AMNH): VII.11.1951; VII.20.1951; VII.21.1951; VII.25.1951; VII.28.1951; VII.30.1951; VII.31.1951; VII.1951; VIII.2.1951; VIII.3.1951; VIII.4.1951; VIII.7.1951; VIII.8.1951; VIII.10.1951; VIII.15.1951; VIII.21.1951; VIII.22.1951. VIII.4.1951, C. & P. Vaurie (MCZC). **TURKS & CAICOS. Grand Turk.** Hawke’s Nest area, 21°26'30"N, 71°07'35"W, under rocks among leaf litter on sandy soil in mixed scrub, II.9.2001, W.E. Steiner, J.M. Swearingen (USNM). North East Point, 21°30'50"N, 71°07'45"W, under rocks and wood debris among low spreading plants on sandy soil near concrete building, II.4.2001, W.E. Steiner, J.M. Swearingen (USNM). North Wells, 21°29'50"N, 71°08'28"W, under wood debris and rocks among leaf ltter in mixed sandy scrub, near salt pond, II.10.2001, W.E. Steiner, J.M. Swearingen (USNM). SE area nr. airport and South Creek, 21°27'15"N, 71°07'45"W, under wood debris and rocks among leaf litter near salt pond, II.6.2001, W.E. Steiner, J.M. Swearingen (USNM). SE side of North Creek, 21°29'25"N, 71°08'W, under rocks and debris on sandy soil, open weedy edges of turf and garden areas, W.E. Steiner, J.M. Swearingen (USNM): II.3.2001; II.5.2001. **Middle Caicos.** Zambarra, uv light, XII.4-11.1993, B.M. Riggs (FSCA). **GREATER ANTILLES. Caymans. Cayman Brac.** 19°43.158'N, 79°47.579'W, uv light, VI.7.2008, Thomas & Turnbow (FSCA). Bight Rd., Parrot Pres., bl trap, V.25.2009, Thomas, Turnbow, Ball (FSCA). Hemmington Road, 19°42.634'N, 79°48.907'W, uv light, VI.8.2008, M.C. Thomas, R.H. Turnbow, B.K. Dozier (FSCA). Hemmington Road at Songbird Drive, bl trap, V.24.2009, Thomas, Turnbow, Ball (FSCA). Major Donald Dr., 0.4 km E jct. Ashton Reid Dr., bl trap: V.24.2009, R. Turnbow (FSCA); V.25.2009, Thomas, Turnbow, Ball (FSCA). Major Donald Dr., 4 km E jct. Bluff Rd., 19.71848N 79.79504W, bl trap, V.23.2009, Thomas, Turnbow, Ball (FSCA). **Grand Cayman.** VIII.25.1908, M. Cameron (BMNH). bl trap, VII.19.1990, P. Fitzgerald (FSCA). uv trap, P. Fitzgerald (FSCA): VII.9.1987; VII.11.1987; IV.1.1990; VI.1992. Boatswain Point, E.J. Gerberg (FSCA): XII.20.1975; VI.21.1976. Boatswain Point, Lime Tree Estate, E.J. Gerberg (FSCA): VII.15.1976; II.20.1984. Botanic Garden, uv trap, M.C. Thomas, R.H. Turnbow, B.K. Dozier (FSCA): VI.4.2008; VI.9.2008. Georgetown, UCCI, bl trap, V.29.2009, Thomas, Turnbow, Ball (FSCA). 3.2 km N Georgetown, flooded freshwater pond, V.26.1975, P.J. Spangler (USNM). 4.8 km N Georgetown, V.26.1975, P.J. Spangler (USNM). W end of Georgetown, light trap, IV.22.1938, C.B. Lewis, G.B. Thompson (MCZC). Vic. Gun Bay, VI.4.2008, R. Turnbow (FSCA). Mastic Trailhead S, V.20.2009, R. Turnbow (FSCA). Mastic Trailhead S, bl trap, Thomas, Turnbow, Ball (FSCA): V.21.2009; V.28.2009. Queen Elizabeth Botanic Park, bl trap, V.21.2009, Thomas, Turnbow, Ball (FSCA). SW Point, suction trap, J. Davies (BMNH): XI.10-11.1970; XI.11-12.1970. West Bay, trap G, X.7.1980, M.E.C. Giglioli (UCDC). West Bay (Town Hall Crescent), uv trap, VII.21-VIII.1.1986, D. Gicca (FSCA). **Little Cayman.** North Coast Rd., 0.1 km W jct. Olivine Kirk Rd., bl trap, V.27.2009, Thomas, Turnbow, Ball (FSCA). Pirate’s Point, MV light, VII.19-20.1975, R.R. Askew (BMNH). **Cuba.** (AMNH), (HNHM). **Camaguey.** Baragua, VIII.19.1927 (MCZC). Baragua, in soil, X.28.1930, L.D. Christenson (MCZC). Baragua, at light, Christenson (USNM): V.16.1932; V.23.1932; V.24.1932; V.26.1932; V.29.1932; V.30.1932; V.31.1932; VI.1.1932; VI.3.1932; VI.4.1932; VI.5.1932; VI.11.1932; VI.14.1932. Camaguey, IV.21-V.5.1933, H.J. MacGillavry (ZMAN). Jaronu, L.C. Scaramuzza [?spelling], VI.16-18.1936 (MHNC). **Cienfuegos.** Cayamas, E.A. Schwarz (USNM): V.6; V.7; V.8; V.20; V.21; V.31. Cayamas, Baker (USNM). Central Constancia, V.1914, J.F. Merrill (USNM). Mina Carlota, Trinidad Mts., VII.1939, Parsons (MCZC). Central Soledad, VII.1.1932, B.B. Leavitt (MCZC). Soledad, G. Salt (MCZC): VI.9.1925; VI.27.1925; VII.3.1925. Soledad, Darlington (MCZC): X.15.1926; X.22.1926; X.25.1926; X.30.1926; XI.9.1926; XII.1.1926; VI.1929; VIII.2-12.1934; IV.1936; V.1936; flood! flow.fd., XI.7.1926; roadside trash, X.14.1926. Soledad (MCZC): V-VI.1939; IX.2.1932, B.B. Leavitt; VIII.6-20, N.A. Weber; berlese funnel, VIII.6-20, N.A. Weber. **Guantanamo.** Guantanamo U.S.Navy Base, uv, IX.3.1964, T.S. Josey (FSCA). Guantanamo Bay, Center Bargo, uv, S. Calhoun (FSCA): VI.19-22.1972; VIII.8.1972. Guantanamo Bay, Beach Housing Area, uv trap, III.24-27.1972, S. Calhoun (FSCA). Guantanamo Bay Navy Base, Kittery Beach Housing Area, UV light, S. Calhoun (FSCA): III.29-30.1972; V.15-18.1972; VII.21.1972; VII.24-25.1972. Guantanamo Bay Navy Base, Caravella Point, uv, S. Calhoun (FSCA): IX.6.1972; X.17.1972; X.30.1972; XI.16.1972; XI.21.1972. Guantanamo Bay Navy Base, Caravella Point, UV trap, IX.26.1972, S. Calhoun (FSCA). Maisi, VII.17.1936, Darlington (MCZC). Savana [sic] la Mar, VIII.5.1936, Darlington (MCZC). San Carlos Est. (AMNH): X.4-8.1913; V.7.1914, C.T. Ramsden. **Havana.** Almendares (IZAC): V.7.1932; V.24.1932; V.27.1932. Almendares (MCZC): VI.13.1928, Staudinger & Bang-Haas; V.25.1932, Jaume. Camoa, VII.15.1931 (MCZC). El Lucero, VI.1945 (IZAC). Havana, Baker (USNM). Havana, from Muche, 1951, Staudinger & Bang-Haas (MCZC). Bosque de la Habana, XI.27.1932, M. Barro (MNHC). Jibacoa, Litoral Costa Norte, V.1962 (MNHC). L. de Ariguanabo, VI.1954, P. de Zayas (IZAC). Marianao, V.15.1932, Jaume (MCZC). Melena del S., Camp Boniato, VII.16.1997, E. Fonseca (PVRC). Playa Iva [?spelling], VI.19.1996, P. & A. (PVRC). Quivican, La Chapa, luz, VIII.9.1997, E. Fonseca (PVRC). S. Ant. Banos, V.1971, L. Armas (IZAC). **Holguin.** Calotones, Gibara, II.18.1998, P. & A. (PVRC). Piloto, Moa, VI.1954, Zayas & Alayo (IZAC). Sierra de Nipe, 23 km S Mayari, Pinares de Mayari, 650 m, VII.1-2.1990, V.O. Becker (WIBF). **Matanzas.** Los Arabos, V.8.1953, M.L. Jaume (INHS). **Pinar del Rio.** 10 km S Pinar del Rio, IX.12-23.1913 (AMNH). **Santiago de Cuba.** Aguadores, VI.6.1936, Darlington (MCZC). Gran Piedra Range, Oriente, 609.6-914.4 m, V.30-31.1936, Darlington (MCZC). coast below Pico Turquino, VI.26-30.1936, Darlington (MCZC). Santiago de Cuba (AMNH). **Villa Clara.** north end of Lago del Hanabanilla, VII.1.1990, J. Rawlins, S. Thompson (CMNH). Santa Clara, III.15-IV.21.1933, H.J. MacGillavry (ZMAN). Est. Exp. Agronomica, Zapata, VIII.18.1960 (IZAC). **not located.** Sierra de Arafe [?spelling] I.26.1947 (MNHC). **Hispaniola: DominicaN RepUBLIC. Barahona.** Barahona, IX.1938, Darlington (MCZC). **Dajabon.** 13 km S Loma de Cabrera, 400 m, V.20-22.1973, D. & M. Davis (USNM). **Distrito Nacional.** Sto. Domingo City, from light in hotel, V.10.1937, Roys (MCZC). **El Seibo.** 17 km SE El Siebo, Rio Chavon, uv, V.28.1985, Woodruff & Stange (FSCA). Pedro Sanchez, UV light at small stream, VI.10.1976, R.E. Woodruff (FSCA). **Espaillat.** Moca, Ciferri (MCSN): 1926; V.1927; VI.1927; VIII.1927; V.1929. **Independcia.** 4 km S Los Pinos, Loma de Vientos, 455 m, 18°35'N, 71°46'W, semiarid deciduous forest with pastures, VII.23.1992, C. Young, S. Thompson et al (CMNH). **La Altagracia.** 2 km N Bayahibe, 10 m, 18°23'N, 6851'W, Dry seasonal forest on limestone, VII.3.1992, C. Young, S. Thompson et al (CMNH). Laguna Nisibon at Rio Maimon, blacklight trap, VI.18.1998, R. Woodruff (FSCA). Nisibon Finca Papagallo, blacklight, VI.16-19.1999, R.E. Woodruff, R.M. Baranowski (FSCA). vic. P.N. de Este, Boca de Uma, 18°21.904'N, 68°37.094'W, at light, 2 m, VIII.5.1999, M.A. Ivie, K.A. Guerrero (WIBF). Oyo Clara, 18°33'48"N” 68°26'50"W, uv light, VII.24-VIII.5.2002, K.W. Will, C. Chaboo (EMEC).Punta Cana Resort, 18°30'16"N, 68°22'37"W, at light, VII.24-VIII.5.2002, K.W. Will, C. Chaboo (EMEC). **La Romana.** Cacata, citrus, VI.11.1976, R.E. Woodruff (FSCA). La Romana, uv, IX.1976, R.E. Woodruff (FSCA). La Romana, uv, IX.16.1976, E. Folch (FSCA). La Romana, uv, R.E. Woodruff (FSCA): IX.17.1976; IX.18.1976; IX.21.1976; IX.22.1976. La Romana, in sugar cane field, uv trap, IX.18.1976, E. Folch (FSCA). **La Vega.** Constanza to Jarabacoa, 609.6-1219.2 m, VIII.1938, Darlington (MCZC). La Cienega, 19°04.07'N, 70°51.68'W, at light, 1100 m, VII.29.1999, M.A. Ivie, K.A. Guerrero (WIBF). La Cienega de Manabao, Park Hdqt, 914 m., blacklight, VII.3-5.1999, R.E. Woodruff (FSCA). 2 km E Manabao, mv & bl, VII.18.1996: M.C. Thomas, R. Turnbow (FSCA); R. Turnbow (RHTC). **Monsenor Nouel.** Bonao, Ciferri (MCSNM): VIII.1926; VI.1927. **Monte Cristi.** 5 km NNE Botoncillo, 50 m, 19°46'N, 7142'W, arid thornscrub, XI.29-30.1992, C. Young, S. Thompson et al (CMNH). 3 km N Villa Elisa, uv, X.1.1983, Woodruff & Stange (FSCA). **Pedernales.** along Rio Mulito, 13km N Pedernales, 230 m, 18°09'N, 71°46'W, riparian woodland, VII.17.1992, C. Young, S. Thompson et al (CMNH). Cabo Rojo, in pool & at lights, 0-10 m, VIII.28-29.1988, M. Ivie, Philips, Johnson (UASM). Cabo Rojo, 10 m, 1955'N, 71°39'W, coastal desert and brackish tidal flats, VII.15-181992, C. Young, S. Thompson et al (CMNH). **Puerto Plata.** Los Hidalgos, VI.4-5.1969, Flint & Gomez (USNM). Pto. Plata (MCZC): Hurst; VII.20.1937, Clench. **Samana.** Sanchez: VI.7-12.1915 (AMNH); VII.1938, Darlington (MCZC). **San Juan.** Rio Mijo, uv, V.20.1985, Woodruff & Stange (FSCA). San Juan de M., V.10.1928, Ciferri (MCSN). **Hispaniola: Haiti. Ouest.** Gonave I., VII.18.1927, S.T. Danforth (MCZC). Manneville, XI.16-17.1934, Darlington (MCZC). Port-au-Prince, III.1925, G.N. Wolcott (MCZC). **Nord.** St. Michel, XII.25, E.C. Leonard (USNM). **Sud-Est.** Jacmel, III.5.1898, E.A. Klages (CUIC). **Jamaica. Clarendon Parish.** 2 km S Rocky Point, nr Jackson Bay Cave, uv light, XII.10.1975, G.F. Hevel (USNM). Milk River Bath, VI.19.1970, E.G. Farnsworth: uv light trap (FSCA); mosquito trap (FSCA), (UASM). Milk River Bath, uv, R.E. Woodruff: V.14.1969 (FSCA); XI.19.1968 (FSCA), (UASM). Portland Cottage (label reads St. Catherine Paris), uv, VII.4.1970, E.G. Farnsworth (FSCA), (UASM). Portland Ridge, PWD Fishing Club, uv, VIII.20.1969, Woodruff (FSCA). **Manchester Parish.** Mandeville, uv trap, J.H. Frank (UASM): VIII.22-25.1969; IX.19-21.1969. Mandeville, uv light trap, J.H. Frank (UASM): I.15-17.1970; VI.4-5.1970; VII.16-18.1970; VIII.14-16.1970. nr Oxford Caves, uv trap, VII.19.1970, J. Farradane (BMNH). Mandeville, A.E. Wight (MCZC). **Pedro Cays.** SW Cay, IV.13.1940, C.B. Lewis (IJSM). **Portland Parish.** Pt. Antonio, A.E. Wight (MCZC): III.28; IV.25; IV.29; V.24. **St. Andrew Parish.** 41 Sunrise Crescent, X.1977, T.H. Farr (IJSM). Beverley Hills, R.P. Bengry (IJSM): X.1961; VI.1962. Constant Spring, H.B. Tordoff (IJSM): X.16.1946; X.28.1946; X.30.1946. Crossroads, V.25.1949, R.P. Bengry (IJSM). Half-Way Tree: R.P. Bengry (IJSM); IX.10.1950; IX.14.1950; IX.15.1950; IX.23.1950; IX.8.1951; IX.10.1951. Hermitage, 2987 m, II.4.1947, G.B. Thompson (IJSM). Kingston: C.T. Brues (MCZC); XI.1952, G.R. Proctor (IJSM). Kingston, Mona Hotel, uv, X.16.1971, R.M. Baranowski (FSCA). Liguanea, nr Kingston, VI.10.1931, M. Kisliuk (USNM). Molynes Road, VI.10.1949, A.M. Wiles (IJSM). Red Hills Road, XII.3.1962, T.H. Farr (IJSM). Stony Hill, VI.1953, H.W. Stockhausen (IJSM). Swallowfield, X.1950, C.B. Lewis (IJSM). Upper Mountain View, X.1.1946, G.B. Thompson (IJSM). **St. Ann Parish.** Ocho Rios, VIII.20-24.1934, Darlington (MCZC). Runaway Bay, uv, VII.12.1970, E.G. Farnsworth (FSCA), (UASM). **St. Catherine Parish.** Caymanas Estate, uv light trap, V.10.1970, J.H. Frank (UASM). Caymanas Estate, uv trap, VIII.27.1970, J.H. Frank (UASM). Linstead, uv: VI.17.1970, E.G. Farnsworth (FSCA), (UASM); IV.8.1971, R.M. Baranowski (FSCA). Old Harbour, IX.4.1908, M. Cameron (BMNH). Port Henderson, uv, VII.12.1970, E.G. Farnsworth (FSCA), (UASM). Rio Cobre, 8 km above Spanish Town, VIII.29.1934, Darlington (MCZC). Spanish Town, uv, E.G. Farnsworth (FSCA): VII.10.1970; VII.15.1970; VIII.5.1970. Spanish Town, Jamaica School Agr., uv, E.G. Farnsworth (FSCA): III.24-25.1970; IV.11.1970; IV.28.1970; V.3.1970; V.8.19780; VI.3.1970; VI.14.1970. Spanish Town, Jamaica School Agr., mosquito trap, VI.23.1970, E.G. Farnsworth (FSCA). Twickenham Park, uv trap, III.24-25.1970, J.H. Frank (UASM). Twickenham Park, uv light trap, V.8.1970, E.G. Farnworth (UASM). White Marl, ex light shade, III-VII, T.H. Farr, R. Vanderwal (IJSM). Worthy Park, uv: VI.3.1970, E.G. Farnsworth (FSCA), (UASM); VI.10.1971, R.M. Baranowski (FSCA). **St. Elizabeth Parish.** Balaclava, stn #19, VII.27.1935, Chapin & Blackwelder (USNM). Black R., uv trap, VII.14.1970, E.G. Farnsworth (FSCA), (UASM). Chatham, nr Newmarket, V.23.1953, I.H. Sibley (IJSM). **St. James Parish.** Montego Bay, D. Miller (AMNH): VII.18.1975; VII.25.1975. Montego Bay, H.B. Southby (IJSM): X.25.1950; X.30.1950. **St. Mary Parish.** Highgate, uv trap, VI.1.1970, E.G. Farnsworth (IJSM). Oracabesia, III.17.1987, J.A. Sjuey (WIBF). **St. Thomas Parish.** Golden Grove, uv trap, IX.9-10.1969, J.H. Frank (UASM). Jamaica Sugar Estates (nr. Golden Grove), VII.7.1969 (UASM). Yallahs, uv, VI.25.1970, E.G. Farnsworth (FSCA), (UASM). **Trelawny Parish.** Duncans, Howden & Becker (CNCI): VIII.1.1966; VIII.15.1966; VIII.21.1966. Duncans, XI.25.1960, T.H. Farr (IJSM). Duncans, at light, VIII.15.1966, Howden & Becker (CNCI). **NAVASSA ISLAND.** Clench (MCZC): I.1-9.1930; I.1930. near lighthouse, 18°23.82'N, 75°00.74'W, 80 m, VII.30.1998, W.E. Steiner, J.M. Swearingen (USNM). **Puerto Rico.** Caja de Muertos, III.22-23.1935, V. Biaggi Jr. (MCZC). Cangrejos, II.22 (AMNHM). Cangrejos, coconut trash, II.22 (AMNH). Caguas, “12-4-1912”, S.S. Crossman (AMNH). Coamo Springs, VI.5-7.1915 (AMNH). Ensenada, VI.14-19.1915 (AMNH).Faro de Cabo Rojo, V.16.1936, J.A. Ramos (MCZC). Guanica Forest, VII.25-26.1969, H. & A. Howden (UASM). Humacao, IX.18.1930 (MCZC). Lajas, XI.22.1935, H.L. Dozier (USNM). La Parguera, H. & A. Howden (UASM): VII.28.1969; VII.30.1969. L. Cartagena, II.28.1927 (MCZC). Rio Pietras, VIII.23.1930, A.S. Mills (AMNH). San German, Reserva For. Maricao, Km 16.2 on Rte 120, at uv light, VIII.1999, P.W. Kovarik (WIBF). Santa Rita, VII-VIII, (AMNH). San Vicente, VII.6-10.1956, C. & P. Vaurie (MCZC). **Cayo Norte.** off Culebra, IV.14.1965, H. Heatwole, F. McKenzie (USNM). **Culebra Island**. III.3.1906, Wheeler (AMNH). **St. Croix.** H.A. Beatty (MCZC), (WIBF). stn #320, XI.7.1936, Blackwelder (USNM). stn #328, XI.12.1936, Blackwelder (USNM). Golden Grove, uv light, D.F. Keaveny: IV.24.1980 (UASM); V.16.1980 (WIBF); V.23.1980 (WIBF). Point Udall, under leaf litter on sand at edge of ground cover vegetation on dry zone of upper beach, II.12.1996, W.E. Steiner, J.M. Swearingen (USNM). slope above Christiansted, VII.28.1976, R.H. Pine (USNM). **Virgin ISLANDs. Anegada.** III.31.1925 (AMNH). airport, VII.19.1985, S. & P. Miller (WIBF). NW side, W of Bones Bight, X.26.2000, B. & B. Valentine (BDVC). **Camanoe.** at uv light, X.16.2005, B.D. Valentine family (BDVC). **Great Camanoe.** (Upper) uv, (BDVC): X.9.2007, B.D.V. family; X.27.2008, B.D. & S.C. Valentine; X.9.2008, B.D. Valentine, D. Dennis. uv, X.7.2008, B.D. & S.C. Valentine (BDVC). uv, B.D. Valentine family (BDVC): X.8.2007; X.9.2007. uv, X.12.2008, B.D.Val. family (BDVC). uv, B.D. & S.C. Valentine (BDVC): X.16.2008; X.17.2008; X.18.2008; X.19.2008; X.21.2008. **Guana Island.** VII.1-14.1984, S.E. & P.M. Miller (WIBF). X.12.1991, W. Lu (BDVC). 0-80 m (WIBF): VII.13-26.1986, S.E. Miller, M.G. Pogue; VII.9-23.1987, S.E. Miller, V.O. Becker. at uv light: B. & B. Valentine (BDVC): X.8-14.2001; X.9-15.2002; X.23-30.2002. at uv light: B.D. Valentine, S.C. Valentine-Cooper (BDVC): X.19.2003; X.20.2003; X.21.2003; X.22.2003; X.1-6.2004; X.8.2004; X.8-14.2004; X.24.2004; X.28.2004. at uv light, B.D. & B.S. Valentine (BDVC): X.7.2005; X.12.2005; X.13.2005; X.14.2005. sweeping, X.8.1991, W. Lu (BDVC). berlese funnel, X.18.2000, B. & B. Valentine (BDVC). uv, B.D.V. (BDVC): X.9.2005; X.12.2005; X.17.2005. uv, B.D. & S.C. Valentine (BDVC): IX.22.2005; IX.23.2005. uv, B.D. Valentine (BDVC): X.7.2005; X.8.2005. **Norman Island.** above the Bight, VII.7.1976, R.H. Pine (USNM). **St. John.** Est. A Piece of Land, East End, Point Udall, under rocks, I.9.1993, VIBFP (WIBF). Est. Carolina, NW of Cord Bay, at uv light, 76.2 m, V.31.1982, W.B. Muchmore (WIBF). Lameshur Bay, V.I.E.R.S., at uv light, VIII.13.1980, M.A. Ivie (WIBF). Lameshur Bay, VIERS, uv light trap, III.16-17.1984, W.B. Muchmore (WIBF). Ram Head, on point, II.7.1986, W.B. Muchmore (WIBF). **St. Thomas.** VII.1941, C. Parsons (MCZC). Red Hook, uv light, VIII.21.1980, M.A. Ivie (WIBF). **Virgin Gorda.** Coppermine Pt., under rocks, VII.18.1994, M.A. Ivie (WIBF). Windy Hill, at light, XI.13.1992, M.A. Ivie (WIBF). **LESSER ANTILLES. Anguilla.** The Farrington, IX.26-X.1.1986, E. Censky (CMNH). Lower Shoal Bay, (1.5 km SW), 18°15'10N 63°02'20W, III.28.1992, W.E. Steiner, J.M. Swearingen (USNM). Low Ground, Black Garden Rd., 18°13.795'N, 63°03.959'W, in/under rot. loblolly, V.17.2004, M.A. Ivie (WIBF). **Antigua.** D. & L. Stoner (AMNH): VI.18; VI.18-24; VI. (MCZC). Big Buers, V.27.1978, Chalumeau (IREC). Christian Hill, V.27.1978, Chalumeau (IREC). Christian Valley, bl trap, FAO Insect Survey (CMNC): VII.18.1991; VII.26.1991. Christian Valley, blacklight trap, IX.23.1991, FAO Insect Survey (CMNC). Christian Valley Agr. Stn., uv light trap, FAO Insect Survey, R.E. Woodruff (CMNC): IX.11.1991; IX.25.1991; X.18-21.1991. St. Anns Hill, at light, IV.19.1958, J.F.G. Clarke (USNM). **Barbados.** IX.1900, D.L. Uyttenboogaart (ZMAN). coast 9.7 km N Bridgetown, VII.25-30.1972, D. Miller (AMNH). Graeme Hall, Entomology Compound, blacklight trap, IX.6.1988, R. Adams (CMNC). **St. George.** Groves, uv, IX.10.1972, M.M. Alam (FSCA). **St. Michael.** Cavehill, uv, M.M. Alam: VII.10-30.1972 (FSCA), (UASM); VII.31-VIII.2.1972 (FSCA), (UASM); VIIII.5-7.1972 (FSCA); VIII.8-10.1972 (FSCA), (UASM); VIII.11-13.1972 (FSCA), (UASM); VIII.14.1972 (FSCA), (UASM); VIII.18-20.1972 (FSCA). **Barbuda.** Codrington, at light: IV.26.1958, J.F.G. Clarke (USNM); VII.2.1962, E.J. Pearce (BMNH). Codrington, 0-15 m, VII.1976, N.L.H. Krauss (AMNH). Codrington, 0-20 m, VII.1976, N.L.H. Krauss (AMNH). Codrington Vill., V.10.1982, B. Espinal (IREC). NW of Codrington Village, stn #603, VII.5.1955, P. Wagenaar Hummelinck (ZMAN). S part of Goat I., stn #601, VII. 11.1955, P. Wagenaar Hummelinck (ZMAN). Indigo Well, IV.26.1958, J.F.G. Clarke (USNM). **BUCK ISLAND.** B.I. Reef Nat. Mon., Dedrick’s Shack, uv light, VIII.23.1996, A. Poponi (WIBF). **Desirade.** Baie-Mahault, IX.13.1975, Chalumeau (IREC). Point Double, I.24.1964, Hummelinck (CMNH), (IREC). **Dominica. St. George Parish.** Roseau, soil, VI.1.1969, B. Knight (FSCA). **St. Paul Parish.** Springfield Estate, 2.5km ENE Canefield, 15°21'N, 61°22'W 450 m, VI.11-18.1991, J.E. Rawlins, S.A. Thomson (CMNH). **Guadeloupe.** Anse Lagourde’, Chalumeau (IREC): IX.10.1972; IX.17.1972; IX.18.1972; IX.24.1972. Anse Lagourde’, IX.24.1972, Chalumeau (CMNH). Anse l’Eau, Chalumeau (IREC): II.4.1973; II.11.1978; IV.2.1972; V.30.1971. Clugny, VIII. 2.1970, Chalumeau (IREC). Deux-Mamelles, IV.17.1973, Chalumeau (IREC). Duclos, VI.14.1973, Chalumeau (CMNH). Ilet Petite-Terre, V.1.1978, Chalumeau (IREC). Ilet Pigeon: V.20.1972, F. Chalumeau (CMNH); V.21.1978, Chalumeau (IREC). Petite Terre Islands, Terre de Bas, VII.20.2003, J. Touroult (WIBF). Pointe de Jarry, X.3.1979, Chalumeau (IREC). Ste. Rose, VIII. 8.1945, H. Stehle (USNM). **Les Saintes.** (TDH), Chalumeau (IREC): IV.14.1973; IX.16.1978. **Marie Galante.** Capesterre de Marie-Galante, Hotel Le Soleil Levant, at neon light, IX.22.2013, J.-M. Lemaire (JMLC). **Martinique.** Caravelle, VIII.15.1973, Camb. (IREC). St-Pierre, V.1.1975, Chalumeau (IREC). **Montserrat.** Brades, blacklight trap, V.6.1993, P. Jeffers (CMNC). Cassava Ghaut, Beattie House, 16°45.91'N, 62°12.95'W, uv light, 192.6 m, A. Krakower (WIBF): III.23-IV.3.2002; V.30-VI.6.2002. nr Plymouth, 1.6 km W at jct rd to Fox’s Bay, UV trap, VI.25.1977, R.E. Woodruff (FSCA). Rendevouz Bay, 16°48.50'N, 62°12.30'W, uv light, VII.26-31.2005, WIBF grp (WIBF). Richmond Hill, nr Plymouth, VIII.29.1975, J. Cooter (BMNH). Woodlands Beach, at uv light, VIII.13.2005, I.A. Foley (WIBF). **MUSTIQUE.** l’Ancecoy Pont, 12°53'N, 61°11'W, dry scrub woodland, uv trap, 1 m, 08-72, VII.30.2008, S. Peck, M. de Silva (CMNC). Gallicaux Bay, 12°53'N, 61°11'W, dry scrub woodland, uv trap, 20 m, 08-70, VII.29.2008, S. Peck, M. de Silva (CMNC). **Nevis.** VI.29.1936, S.T. Danforth (MCZC). **SABA.** windwardside, III.25.1986, R.S. Miller (WIBF). windwardside, blacklight trap, VIII.19.1992, H.V. & R.M. Baranowski (CMNC). Bottom, at light, XI.29.2010, M. Gillet (WIBF). Cove Bay, Tide Pools Trail, below airport, beating, V.20.2008, M.A. Ivie (WIBF). Mountain Road, light, IX.8.2010, M. Gillet (WIBF). Spring Bay Trail, 17.63737°N 63.22126°W, rocky beach, 37.9 m, III.10.2008, D.S. Sikes (WIBF). **SOMBRERO.** 18°35.17'N, 63°25.63'W, XI.12-13.1999, M.A. Ivie, J.B. Runyon (WIBF). **St. BarthÉlemy.** Lorient, IV.25.1974, Chalumeau (IREC). Quat./Flamands, V.29.1973, Chalumeau (IREC). **ST. EUSTATIUS.** Venus Bay, Boven Sec Natl. Park, 17.51544°N 62.98960°W, beach, beating, 0-6 m, V.26.2008, M. Ivie, N. Esteban (WIBF). **St. Kitts.** Frigate Bay, in cow dung, IV.12.1956, J.F.G. Clarke (USNM). **St Lucia.** Anse La Raye, Anse Galet, 1 km SSW Anse La Raye, 13°56'N, 61°03'W, 50 m, VI.21-30.1991, J.E. Rawlins, S.A. Thompson (CMNH). Barre de l’Isle, 13.9368°N 60.9594°W, trap site, uvlight, 340 m, VI.25-28.2009, E.A. Ivie (WIBF). Escap Community, 13.83242°N 60.89864°W, 46 m (WIBF): at light, IV.26-30.2009, I.A. Foley, R.C. Winton; ridge, uv light, V.1.2009, I.A. Foley; at house, V.1-10.2009, I.A. Foley, R.C. Winton; VI.28-VII.3.2009, M.A. Ivie et al. Escap Community, 13.8324°N 60.8986°W, 46 m, R.C. Winton et al (WIBF): VI.1-4.2009; at house, VI.1-16.2009. Escap Community, 13.8324°N 60.8986°W, at house, VI.28-VII.3.2009, WIBF group (WIBF). Escap Comm., 13.8324°N 60.8986°W, 30 m, I.A. Foley (WIBF): at light, IV.30.2009; merc vap light, V.1.2009. Gros Ilet, IV.11.1978, Chalumeau (IREC). Grande Anse, 14.0052°N 6-.8973°W, trap site, malaise, 38 m, V.31-VI.4.2009, C.A. Maier, R.C. Winton (WIBF). Gros Islet, Belle vue, 14°5.271'N, 60°56.576'W, at light, XII.19.2009, H. Dupal (WIBF). La Porte, 13.8404°N 60.9740°W, sweeping, 272 m, VI.1.2009, S.M. Clark et al (WIBF). Marigot Bay, blacklight trap, VIII.22.1988, M. Paul (CMNC). Mon Repos, Fox Grove Inn, 13°51.8'N, 60°54.4'W, uv light into trees, 90 m, S. & J. Peck (CMNC): 07-50, VII.8-18.2007; 07-75, VII.20-28.2007. **St. Martin.** Baie de Friar, V.26.1973, Chalumeau (IREC). Cul de sac, II.3.1978, Chalumeau (IREC). Pic Paradis, V.2.1981, Chalumeau: (CMNH), (IREC). Pt. Blanche Bay, stn #606, VI.5.1955, P. Wagenaar Hummelinck (ZMAN). N of Pt. Blancha, stn #074, V.2.1973, P. Wagenaar Hummelinck (ZMAN).

### *Selenophorus striatopunctatus* Putzeys

**UNITED STATES. Florida.** Miami-Dade County. Chekika State Rec Area, Grossman Hammock For., 50 km SW Miami, malaise, FIT, S. & J. Peck (UASM): XI.1.1984-III.3.1985; III.3-IV.28.1985; V.1-VIII.2.1985. Everglades Nat. Pk.: XI.29.1970, G.E. Ball (UASM); junk hammock, VII.10.1959, R.M. Baranowski (MCZC). Homestead, D.E. Bright (CNCI): V.2.1967; V.11.1967. Homestead (MCZC): VI.1929, Darlington; III.13.1957, R.M. Baranowski. Homestead, at light, VI.2.1957, R.M. Baranowski (AMNH). Monroe County. Key Largo, light trap, S. Kemp (USNM): VII.11.1965; VII.14.1965. Middle Torch Key, Sec 17, Lazelle Place, uv light, VIII.10-19.1992, S. & J. Peck (UASM). Sugarloaf Key, Sec 25 SE1/4, Kitchings Hammock, uv trap, VIII.10-19.1992, S. & J. Peck (UASM). [**not mapped, north of map used**: Alachua County. Gainesville, VII.27.1976, L.R. Davis, Jr. (DRMC). Gainesville, 2510 NE 10^th^ Terrace, black light, L.R. Davis, Jr. (DRMC): VIII.6.1976; VIII.7.1976; VIII.9.1976; VIII.10.1976; VIII.16.1976.] [**not mapped, north of map used**: Duval County. Jacksonville, VIII.1943, G.S. Hensill (CASC).] **BAHAMAS. Andros Island.** BARC, pasture adjacent to low interior coppice, black light, VII.8.1987, J. Browne (CMNC). Forfar Field Station, Stafford Creek, uv light in coastal coppice, VI.4.2001, M.C. Thomas (FSCA). Mennenite’s Farm, crop, black light, VII.30.1987, J. Browne (CMNC). **Grand Bahama.** Freeport, VI.20-27.1987, W.E. Steiner, M.J. & R. Molineaux (USNM). Pine Ridge, at light, V.13.1953, E.B. Hayden, G.B. Rabb (AMNH). **Man-O-War Cay.** VIII.15.1971, H. & A. Howden (CNCI). **New Providence Island.** Carmichael area, 25°01'N, 77°25'W, at uv light in Caribbean pine forest and scrub, W.E. Steiner, J.M. Swearingen (USNM): IV.14.2007; IV.17.2007. Nassau, UV trap, VI.24.1972, FD Bennett (FSCA). **South Bimini Island.** VIII.10.1951, C. & P. Vaurie (AMNH), (MCZC). **GREATER ANTILLES. CAYMANS. Grand Cayman.** uv light, P. Fitzgerald (FSCA): 8.V.1992; VI.1992. Botanic Garden, uv light, VI.4.2008, M.C. Thomas, R.H. Turnbow, B.K. Dozier (FSCA). Mastic Trailhead S, bl trap, V.21.2009, Thomas, Turnbow, Ball (FSCA). SW Point, suction trap, XI.11-12.1970, J. Davies (BMNH). West Bay, Town Hall Crescent, uv trap, VII.21-VIII.1.1986, D. Gicca (FSCA). **Little Cayman.** Pirates’ Point, MV trap, VII.18.1975, R.R. Askew (BMNH). **Cuba.** [illegible label]: C. Wright (MCZC); E. Gutierrez (MNHC). **Camaguey.** Baragua, at light, XI.12.1929, L. Scaramuzza (MCZC). Galbis, IV.21.1920 (CUIC). **Cienfuegos.** Soledad, G. Salt (MCZC): VI.9.1925; VI.27.1925. Soledad, Darlington (MCZC): XI.1.1926; XI.7.1926; VI.1929; VIII.2-12.1934; IV.1936; V.1936; VIII.1936; berlese funnel, VIII.6-20; big flood, X.21.1926. Soledad: XI.7.1926, Darlington (USNM); V-VI.1939, Parsons (MCZC); VI.2.1955 (IZAC). Central Soledad, VII.1.1932, B.B. Leavitt (MCZC). Trinidad Mts., Buenos Aires, VI.1939, Parsons (MCZC). Trinidad Mts., Hanabanillo Falls, IV.30.1936, Darlington (MCZC). **Granma.** Rio Yara, 38.1-304.8 m, V.15-20.1948, J. Ferras (PVRC). **Guantanamo.** Guantanamo U.S. Navy Base, uv, IX.3.1964, T.S. Josey (FSCA). Guantanamo Bay, Center Bargo, uv, VI.19-22.1972, S. Calhoun (FSCA). Guantanamo Bay Navy Base, Caravella Point, uv, X.27.1972, S. Calhoun (FSCA). **Havana.** Havana, Barbour (MCZC). Est. Exp. Agronomica, Havana, V.1958, E.E.A. (IZAC). Almendares: VI.20.1930, Jaume (MCZC); 1932, Jaume (MCZC); V.24.1932 (IZAC); VI.20.1930 (IZAC). Camoa, VII.15.1931 (MCZC). Laguna Ariguanabo: VI.4.1960 (MNHC); VI.23.1963 (IZAC). Quivican, La Chapa, luz, VIII.9.1997, E. Fonseca (PVRC). Stgo. de las Vegas: VI.1959 (IZAC); VI.6.1960, M. Barro (MNHC). Santiago de Las Vegas, “7-3-17”, P. Cardin(USNM). **Isla de la Juventud.** Nueva Gerona, I.18.1967, R. Bielawski, A. Riedel (IZWP). **Matanzas.** Matanzas, VI.3.1933, M. Barro (IZAC). Playa Larga, Cienaga Zapate, luz, IV.1997, P. Valdez R. (PVRC). **Pinar del Rio.** Guane, VII.6.1933, S.T. Danforth (MCZC). Rangel (AMNH). Vinales, San Vicente, VIII.19.1963 (USNM). **Villa Clara.** north end of Lago del Hanabanilla, VII.1.1990, J. Rawlins, S. Thompson (CMNH). **Hispaniola: Dominican RepUBLIC. Barahona.** 6 km NW Paraiso, Rio Nizao, 170 m, 18°02'N, 71°12'W, VII.25-26.1990, Thompson, Young et al (CMNH). **Distrito Nacional.** Santo Domingo, VII.16.1966, L.H. Rolston (TAMU). **Espaillat.** Moca, V.1929, Ciferri, (MCSN). **Independencia.** ESE Jimani, S Lago Limon, 18°24'N, 71°44'W, uv light, 20 m, VII.3.1992, M.A. & R.O. Ivie (WIBF). 4 km S Los Pinos, Loma de Vientos, 455 m, 18°35'N, 71°46'W, semiarid deciduous forest with pastures, VII.23.1992, C. Young, S. Thompson et al (CMNH). **La Altagracia.** vic. Oyo Clara, 18°33'48"N, 68°26'50"W, uv light, VII.24-VIII.5.2002, K.W. Will, C. Chaboo (EMEC). Punta Cana Resort, 18°30'16"N, 68°22'37"W, at light, VII.24-VIII.5.2002, K.W. Will, C. Chaboo (EMEC). **La Vega.** Jarabacoa, VI.3-4.1969, Flint & Gomez (USNM). near mouth Arroyo Los Dajaos, 5 km E Manabao, 140 m, 19°04N 7045"W, riparian woodland, X.9.1991, C. Young, S. Thompson et al (CMNH). La Cienega, 19°04.07'N, 70°51.68'W, 1100 m, VII.29.1999, M.A. Ivie, K.A. Guerrero (WIBF). La Cienega de Manabao, Park Hdqt., 914 m, blacklight, VII.3-5.1999, R.E. Woodruff (FSCA). 2 km E Manabao, ultraviolet & mercury vapor light, VII.18.1996, M.C. Thomas, R. Turnbow (FSCA). **La Romana.** La Romana, uv trap, IX.13.1976, E. Folch (FSCA). La Romana, uv, R.E. Woodruff (FSCA): VIII.22.1977; IX.21.1976. **Monte Cristi.** 5 km NNE Bontoncillo, 50 m, 19°45'N, 71°42'W, arid thornscrub, XI.29-301992, Davidson, Klingler (CMNH). Carbonera, VII.24.1978, R.O. Schuster, R.S. Rominger (UCDC). 3 km N Villa Elisa, uv, X.1.1985, Woodruff & Stange (FSCA). 5 km N Villa Elisa, V.10-18.1985, E. Giesbert (FSCA). **Pedernales.** Cabo Rojo, in pool & at lights, 0-10 m, M. Ivie, Philips, Johnson (WIBF): VIII.24-28.1988; VIII.28-29.1988. Cabo Rojo, Alcoa Headquarters, blacklight, VI.20-24.1999, R. Woodruff, R. Baranowski (FSCA). 23.5 km N Cabo Rojo, 540 m, 18°06'N, 71°38"W, wet deciduous forest, IX.26-271991, Davidson, Klingler et al (CMNH). 24 km N Cabo Rojo, 18°07'N, 71°38'W, uv light, VII.9.1993, D. Sikes, R. Rosenfeld (WIBF). **Puerto Plata.** Los Hidalgos, VI.4-5.1969, Flint & Gomez (USNM). **San Juan.** Rio Mijo, uv, V.20.1985, Woodruff & Stange (FSCA). 11 km S San Juan, at light, VII.12.1985, C. Nunez (FSCA). **Santiago.** Santiago, III.26.1936, S. del Rosario (MCZC). **Jamaica. Claredon Parish.** Milk River Bath, uv, XI.19.1968, R.E. Woodruff (FSCA). Milk River Bath, mosquito trap, VI.19.1970, E.G. Farnsworth (FSCA), (UASM). Portland Cottage (label reads St. Catherine Parish), uv, VII.4.1970, E.G. Farnsworth (FSCA). **Manchester Parish.** Mandeville, uv light trap, J.H. Frank (UASM): IV.10-12.1970; V.22-24.1970; VI.4-5.1970; VI.18-20.1970; VIII.14-16.1970; XI.19-21.1970. Mandeville, uv trap, J.H. Frank (UASM): VIII.22-25.1969; IX.24-26.1970; X.3-5.1969; X.10-11.1970; X.24-26.1970; XI.6-8.1970. **St. Andrew Parish.** Constant Spring, X.28.1946, H.B. Tardoff (MCZC). Kingston, Palisadoes, VIII.25.1966, A.T. Howden (UASM). Mona, XII.18.1946, G.B. Thompson (MCZC), (IJSM). Upper Mtn. View, VII.10.1958, C.B. Lewis (IJSM). **St. Ann Parish.** Runaway Bay, uv trap, VII.12.1970, E.G. Farnsworth (FSCA). **St. Catherine Parish.** Caymanas Estate, V.10.1970, J.H. Frank (UASM): uv light trap; uv trap. Linstead, uv, R.M. Baranowski (FSCA): IV.4.1971; IV.5.1971; IV.8.1971. Port Henderson, uv, VII.12.1970, E.G. Farnsworth (FSCA), (UASM). Spanish Town, uv, E.G. Farnsworth (FSCA): VII.10.1970; VII.15.1970; VIII.1970. Spanish Town, Jamaica School Agr., uv, E.G. Farnsworth (FSCA): III.24-25.1970; IV.11.1970; IV.28.1970; V.3.1970; V.8.1970; VI.14.1970. Spanish Town, Jamaica School Agr., mosquito trap, E.G. Farnsworth (FSCA): III.26.1970; IV.6.1970; IV.20.1970; VI.23.1970. Spanish Town, Twickenham Pk., mosquito lite trap, E.G. Farnsworth (FSCA): V.18.1970; V.22.1970. Twickewnham Park, uv light trap, V.8.1970, E.G. Farnworth (UASM). Twickewnham Park, uv trap, III.24-25.1970, J.H. Frank (UASM). Worthy Park, mosquito lite trap, V.14.1970, E.G. Farnsworth (FSCA). Worthy Park, uv, E.G. Farnsworth (FSCA): III.14.1970; VI.1970. Worthy Park, uv, VI.3.1970, E.G. Farnsworth (UASM). Worthy Park, uv trap, V.11.1969, R.E. Woodruff (FSCA). **St. Elizabeth Parish.** Black River, uv trap, VII.14.1970, E.G. Farnsworth (FSCA), (UASM). **St. Mary Parish.** Highgate, uv, VI.1.1970, E.G. Farnsworth (FSCA). **St. Thomas Parish.** Yallahs, uv, VI.25.1970, E.G. Farnsworth (FSCA), (UASM). **Trelawny Parish.** Vale Royal, X.1-3.1969, R. Arscott (UASM). **Puerto Rico.** Bosque de Guanica, 17°58'50"N, 66°52'37"W, 15W BL, sheet in dry coastal forest at Cobanus Rd., VI.9.2005, R. Brown, S. Lee (MEMU). Ensenada, VI.14-19.1915 (AMNH). Guayama, IX.1916 (CUIC). Isabela Expt. Stn., uv trap, VII.2.1972, A.E. Agostini, R.E. Woodruff (FSCA). Mercedita, X.1976, J. Micheli (USNM). Mercedita, at light, J. Micheli: IV.19.1972 (USNM); III.13.1973 (JMPR); VI.12.1972 (JMPR); VI.16.1972 (JMPR). Ponce, at light, J. Micheli: V.16.1971 (JMPR); V.24.1971 (JMPR); VI.6.1974 (USNM); IX.5.1974 (USNM); IX.27.1974 (USNM). Ponce, at light, VI.1-15.1945, J.M. Capriles (USNM). Ponce, in u-v light trap, IX.24-30.1977, J. Micheli (JMPR), (UASM). Ponce, By-Pass Hwy., at light, J. Micheli (USNM): IX.25.1974; IX.26.1974. Rte. 132, Km 20, at lights, IV.28.1972, J. Micheli (JMPR). San German, Reserva For. Maricao, Km 16.2 on Rte 120, at uv light, VII.8.1999, P.W. Kovarik (WIBF). Santa Rita, VII.13, E.G. Smyth (CASC). Utuado, E. Vales (MCZC): VII.17.1930; VII.23.1930; VII.26.1930; VII.30.1930. **LESSER ANTILLES. Barbados.** Graeme Hall, Entomology Compound, blacklight trap, IX.6.1988, R. Adams (CMNC). **St. George.** Groves, uv, IX.26.1972, M.M. Alam (FSCA). **St. Michael.** Cavehill, M.M. Alam (FSCA): VII.1-9.1972; VII.10-30.1972; VII.31-VIII.2.1972; VIII.11-13.1972; VIII.18-20.1972. Cavehill, uv light, VII.10-30.1972, M.M. Alam (CMNC). **St. Thomas.** Edgehill, uv, M.M. Alam (FSCA): X.6-7.1972; X.11-12.1972; X.13-14.1972; X.14-15.1972; X.19-20.1972; X.26-27.1972; XI.5-6.1972. Jack-in-Box Gully, 13°11'N, 59°34.3'W, forest, uv light, 230 m, 07-03, VI.5.2007, S. & J. Peck (CMNC). **Grenada. St. Andrew Parish.** Balthasar R., 1.6 km E Grenville, uv light, VI.13.1977, R.E. Woodruff, E.E. Grissell (FSCA). Mirabeau, Agr. Lab., blacklight trap, X.28.1990, J. Telesford (CMNC). Mirabeau, Agr. Lab., bl trap, III.17.1990, J. Telesford (CMNC). Mirabeau, Agr. Lab., l. trap, J. Telesford (CMNC): IX.12.1990; IX.14.1990. Mirabeau, Agr. Lab., l. trap, (CMNC): II.28.1990, R.E. Woodruff; IV.11.1990, A. Thomas. Mirabeau Agr. Lab., blacklight trap, IX.29.1990, R.E. Woodruff (FSCA). Mirabeau Agr. School, at light, II.28.1990, R.E. Woodruff (FSCA). **St. George Parish.** Point Salinas, l. trap, VI.27.1987, R. Woodruff (CMNC). **St. Patrick Parish.** Plains, l. trap, V.13.1990, F. Noel (CMNC). **Guadeloupe.** Basse-Terre, Matouba, VIII.13.2003, J. Touroult (WIBF). Saint-Francois, V.10.1983, Chalumeau (IREC). **St. Lucia.** Castries, uv trap, VI.19.1977, R.E. Woodruff (FSCA), (CMNC). Union Agricultural Sta., IX.11.1986, Crop Protection Unit (CMNC). **St. Vincent. St. George Parish.** Cane Hall, Rick’s Apt., blacklight trap, X.1.1991, R.E. Woodruff (FSCA).

### *Stenomorphus californicus manni* Darlington

**HISPANIOLA. DOMINCAN REPUBLIC. Independencia**. 4 km S Los Pinos, Loma de Vientos, 18°35'N, 71°46'W, semiarid deciduous forest with pastures, R. Davidson, J. Rawlins, S. Thompson, C. Young (CMNH): 455 m, VII.23.1992; 475 m, X.12.1991.

### *Discoderus beauvoisii* (Dejean)

**BAHAMAS. Mayaguana Island.** uv, C.M. Murvosh (FSCA): VIII.1.1963; VIII.3.1963; VIII.24.1963; VIII.25.1963; VIII.26.1963; VIII.27.1963; VIII.28.1963. uv light trap, VIII.28.1963, C.M. Murvosh (UASM). **GREATER ANTILLES. CUBA. Guantanamo.** Calatones, Gibara, luz, II.16.1998, P. & A. (PVRC). Guantanamo Bay Navy Base, Kittery Beach Housing Area, uv light, S. Calhoun (FSCA): III.24-27.1972; III.29-30.1972; VIII.21.1972. Guantanamo Bay Navy Base, Kittery Housing Area, uv, S. Calhoun (FSCA): III.24-27.1972; III.29-30.1972; V.8-11.1972; V.15-18.1972; VII.24-25.1972. Guantanamo Bay Navy Base, Kittery Housing Area, uv lights, V.15-18.1972, S. Calhoun (UASM). Guantanamo Bay, Caravella Point, uv light, S. Calhoun (FSCA): VII.24-25.1972; IX.26.1972; X.2.1972; X.10.1972; X.11.1972; X.17.1972; X.19.1972; X.23.1972; X.27.1972; X.30.1972; XI.6.1972; XI.8.1972; XI.13.1972; XI.14.1972; XI.15.1972; XI.16.1972; XI.20.1972; XI.21.1972; XII.14.1972. Guantanamo Bay, Center Bargo, uv, S. Calhoun (FSCA): VI.19-22.1972; VIII.3.1972; IX.7.1972. Guantanamo Bay, Center Bargo, New Jersey trap, IX.9.1972, S. Calhoun (FSCA), (UASM). Guantanamo U.S. Navy Base, uv, IX.3.1964, T.S. Josey (FSCA). Sierra Maguey, Manuel Tames, VI.12.1990 (MNHC). Tortuguilla, VI.1967, Alayo & Zayas (IZAC). **Santiago de Cuba.** Cobagan, El Cobre, VIII.1975, L.F. Armas (IZAC). La Gran Piedra, XI.1971, L.F. Armas (IZAC). coast below Pico Turquino, VI.26-VI.30.1936, Darlington. Versalles, VI.6.1967, Alayo, Zayas, Garcia (IZAC). **HISPANIOLA: DOMINICAN REPUBLIC. Azua.** El Numero, VI.27.1985, K. Guerrero (CMNH). 8 km NE Padre Las Casas, Rio Las Cuevas, 18-46N 70-53W, 580 m, VIII.7.1990, J. Rawlins, S. Thompson (CMNH). 8 km NE Padre Los Casas, Rio Las Cuevas, 580 m, 18°46'N, 70°53'W, riparian growth in arid thornscrub, VIII.7.1990, J. Rawlins, S. Thompson et al (CMNH). **Barahona.** IX.1938, Darlington (MCZC). 12.6 km NE Las Minas, Azua-Barahona Hwy, desert, 140 m, VII.19.1999, M.A. Ivie, R.S. Miller (WIBF). 4 km NE Polo, 1260 m., VII.9.1987, R. Davidson, J. Rawlins (CMNH). **Distrito Nacional.** Santo Domingo, V.13.1967, L.H. Rolston (TAMU). **Espaillat.** Moca, Ciferri (MCSN): V.1927; VI.1927. **Independcia.** 4 km S Los Pinos, Loma de Vientos, 455 m, 18°35'N, 71°46'W, semiarid deciduous forest with pastures, VII.23.1992, C. Young, S. Thompson et al (CMNH). **La Altagracia.** 2 km N Bayahibe, 10 m, 18°23'N, 6851'W, dry seasonal forest on limestone, VII.3.1992, C. Young, S. Thompson et al (CMNH). **La Vega.** Constanza, VI.2-VI.6.1969, Flint & Gomez (USNM). road S Balneario R. Grande, 5.5km S Constanza, 1500 m, pine, under rock, II.6.1975, W.L. & D.E. Brown (MCZC). 6 km SE Constanza, 18°52'N, 70°42'W, 1400 m, disturbed fields with scattered pines, XI.241992, Klingler, Davidson et al (CMNH). **Monte Cristi.** 5 km NNE Bontoncillo, 50 m, 19°45'N, 71°42'W, arid thornscrub, XI.29-30.1992, Davidson, Klingler et al (CMNH). Monte Cristi, VI.21.1967, L.H. Rolston (TAMU). 4.8 km N Villa Elisa, mv & bl l, V.31.1994, R. Turnbow (UASM). 3 km N Villa Elisa, uv, Woodruff & Stange (FSCA): X.1.1983; X.1.1985. 5 km N Villa Elisa, V.10-18.1985, E. Giesbert (FSCA). 8.2 km N Villa Elisa, mv & bl l, VI.1.1994, R. Turnbow (UASM). **Pedernales.** Cabo Rojo, at light, 0-10 m, M. Ivie, Philips, Johnson (WIBF): VIII.24.1988; IX.8-9.1988; IX.10.1988. Cabo Rojo, hotel, VIII.21.1992, D. Sikes, J. Brodzinsky (WIBF). Cabo Rojo, in pool & at light, 0-10 m, M. Ivie, Philips, Johnson (WIBF): VIII.18-23.1988; VIII.24-28.1988; VIII.28-29.1988; IX.8-9.1988. Cabo Rojo, sweeping pool, VII.9.1993, D.S. Sikes, R.P. Rosenfeld (WIBF). Cabo Rojo, coast thorn scrub, swim pool surface, 10 m, XI.28-XII.2.1991, 91-362, Masner & Peck (CMNC). Cabo Rojo, 17-55N 71-39W, 10 m, VII.27.1990, Rawlins, Young, Thompson (CMNH). Cabo Rojo, 10 m, 19°55'N, 71°39'W, coastal desert and brackish tidal flats, VII.15-181992, C. Young, S. Thompson et al (CMNH). Cabo Rojo, 10 m, 19°55'N, 71°39'W, coastal desert, C. Young, S. Thompson et al (CMNH): IX.26-27.1991; X.19-231991. Cabo Rojo, sea level, 19°55'N, 71°39'W, edge of salt marsh, X.21.1991, C. Young, S. Thompson et al (CMNH). Cabo Rojo, uv light, VII.8.1993, D.S. Sikes, R.P. Rosenfeld (WIBF). Cabo Rojo, Alcoa Headquarters, blacklight trap, VI.20-24.1999, R. Woodruff, R. Baranowski (FSCA). 1 km E Cabo Rojo, 17-55N 71-39W, 10 m, VII.30.1990, Rawlins, Young, Thompson (CMNH). 9.5 km N Cabo Rojo, 18°00'N, 71°39'W, at uv light, 42m, VII.10.1993, D.S. Sikes, R.P. Rosenfeld (WIBF). 10 km E Cabo Rojo, under dry cow dung in desert, VI.20-24.1999, R.E. Woodruff (FSCA). 14.5 km N Cabo Rojo, 165 m, 18°03'N, 71°39'W, thorn scrub: IX.26-27.1991, Davidson, Young et al (CMNH); VII.19.1990, Rawlins, Davidson, Thompson (CMNH). Cabo Rojo, XI.4.1986, W.J. Pulawski (CASC). **Peravia.** 8 km W Bani, uv, V.25.1985, Woodruff & Stange: (FSCA), (UASM). 12.4 km E Rio Ocoa, VII.3.1992, M.A. & R.O. Ivie (WIBF). 2 km E Los Ranchitos, 10 km SSE San Jose de Ocoa, 700 m, 18°28'N, 70°28'W, semiarid woodland, X.4.1991, Young, Thompson et al (CMNH). San Jose de Ocoa, VII.4.1978, Chalumeau & Abud (IREC). **Puerto Plata.** Los Hidalgos, VI.4-5.1969, Flint & Gomez (USNM). **San Jose de Ocoa.** 8 km S Las Carreras, VIII.31.1997, P. Kovarik (WIBF). **San Juan.** 11 km WNW Hato Nuevo, 1 km SE Ingenito, Presa de Sabaneta, 19°02'N, 71°18'W, near shoreline, Rawlins, Davidson, Onore (CMNH). Rio Mijo, uv, V.20.1985, Woodruff & Stange (FSCA). 11 km S San Juan, at light, VII.12.1985, C. Nunez (FSCA), (UASM). San Juan de Mag., V.10.1928, Ciferri (MCSN). Vallejuelo, at light, VII.12.1985, C. Nunez (FSCA), (UASM). **Santiago.** Santiago, 1930, Ciferri (MCSN). Par. Nat. Armando Bermudez, Rio Bao, 1212 m, VII.10.1992, M.A. & R.O. Ivie (WIBF). **HISPANIOLA: HAITI. Artibonite.** Ennery, nr 304.8 m, IX.6-11.1934, Darlington (MCZC). Gonaives, II.4-9.1908, M. Cameron (BMNH). Montrouis, IX.6.1934, Darlington (MCZC). 2 km S Montrouis, uv trap, VII.2.1977, J.H. Frank (UASM). 5 km S Montrouis, under dry grass, V.2.1977, J.H. Frank (UASM). St. Marc, Mann (MCZC). **Ouest.** Camp Perrin, nr 304.8 m, X.8-27.1934, Darlington (MCZC). Damien, (Port-au-Prince), VI.6.1933, D. Gaston (MCZC). Kenskoff (nr Port-au-Prince), 1219.2-1828.8 m, Darlington (MCZC): IX.2.1934; IX.16.1934; IX.23.1934; XI.9.1934. Manneville, Mann (MCZC). 2 km S Montrouis, uv trap, VII.2.1977, J.H. Frank (UASM). 5 km S Montrouis, uv trap, V.2.1977, J.H. Frank (UASM). Port-au-Prince: VIII.1924, G.N. Wolcott (USNM); V.17.1934, M.M. Saylor (CASC); VII.18-21.1955, A.F. Archer (MCZC); E.M. Ducasse (MCZC). Port-au-Prince (USNM). Port-au-Prince, Thor (a suburb), X.10-12.1970, J.E. Porter (FSCA): UV light; P. Daniels res., uv. **Nord-Ouest.** Tortue I., Bassin Bleu, IV.1929, E.C. & G.M. Leonard (USNM). **Sud-Oueste.** Parc National La Visite, vic. Park headquarters, 1880 m, V.10.1984, M.C. Thomas (USNM). **JAMAICA. Clarendon Parish.** Milk River Bath, uv, XI.19.1968, R.E. Woodruff (FSCA), (UASM). **St. Andrew Parish.** Half-Way-Tree: I.28.1937, Chapin & Blackwelder (USNM); I.28.1937, Chapin & Blackwelder (MCZC); IX.3.1950, R.P. Bengry (IJSM); IX.18.1950, R.P. Bengry (IJSM); X.10.1951, R.P. Bengry (IJSM). Kingston, I.1931, Chapin & Blackwelder (USNM), (MCZC). Kingston: VIII.27.1934, Darlington (MCZC); VII.8.1958, VC Sanderson (CNCI). Kingston, stn #50, IV.22.1941, Chapin (USNM). Kingston, West Race Course, A. Shaw (IJSM): VI.3.1946; X.11.1946. nr N end of Race Course, XI.12.1954, G.R. Proctor (IJSM). Stonyhill, I.1931, Chapin & Blackwelder (USNM). Swallowfield, C.B. Lewis (IJSM): X.1950; XI.5.1951; X.27.1951. Upper Mountain View, G.B. Thompson (IJSM): X.11.1946; X.12.1946; X.19.1946; X.20.1946; X.22.1946; X.24.1946; X.25.1946. Upper Mountain View, C.B. Lewis (IJSM): V.18.1947; IX.26.1947; XI.4.1947; XII. 12.1949; IV.4.1950; III. 16.1953; X.31.1954; XI.19.1954. **St. Catherine Parish.** Port Henderson Hill, VII.7.1958, T.H. Farr (IJSM). Spanish Town, Jamaica School Agr., uv, IV.11.1970, E.G. Farnsworth: (FSCA), (UASM). Spanish Town, stn #377, II.2.1937, Chapin & Blackwelder (USNM). Worthy Prk, uv, VI.3.1970, E.G. Farnsworth (FSCA), (UASM). **St. Elizabeth Parish.** Black R., IV.17.1937, C. Roys (MCZC). Gt. Pedro Bluff, V.10.1956, G.R. Proctor (IJSM). **PUERTO RICO.** Aguirre: IV.17.1925, H.E. Box (USNM); VI.29.1931, (AMNH). Banos de Coamo, under cow pie, VII.20.1976, J. Micheli (JMPR). Banos de Coamo, under dung, VII.30.1976, J. Micheli (UASM). Hwy 100, SW Cabo Rojo, dead tree with pine wood, VIII.17.2002, S.C. Jones, G. Lopez (BDVC). Coamo Springs, IV.30.1932, S.T. Danforth (MCZC). Ensenada: VI.14-19.1915 (AMNH); XI.13.1925 (AMNH); VII.1928 (AMNH); II.2.1929, S.T. Danforth (MCZC); III. 21.1936, J.A. Ramos (MCZC). Fortuna A.E.S., I.1961, F. Fisk (OSUC). Guanica: X.2.1913, E.G.S. (AMNH); X.2.1913, E.G.S. (MCZC); I.1914, R.T. Cotton (CUIC). Guanica Bay nr. Jaboncilla, VIII.18.2002, S.C. Jones, G. Lopez (BDVC). Juan Diaz, “11.12.1925” (AMNH). La Parguera, VII.28.1969, H. & A. Howden (UASM). Paguera, “II-18” (AMNH). Rio Guanajibo at Sabana Grande, 100 m, V.4.1985, Hoebeke, Liebherr, Nichols (CUIC). San German, 1923, G.N. Wolcott (AMNH), (MCZC). Santa Rita, VII-VIII (AMNH). **VIRGIN ISLANDS. Camanoe.** at uv light, X.16.2005, B.D. Valentine family (BDVC). **Great Camanoe.** (Upper) uv (BDVC): X.8.2005, B.D. Valen. family; X.12.2005, B.D.V. family, uv, B.D. & S.C. V. (BDVC). **Guana Island.** X.15.1993, C.Bartlett (WIBF). at uv light, B.D. Valentine, S.C. Valentine-Cooper (BDVC): X.2-4.2004; X.8.2004; X.8-14.2004; X.14.2004; X.18.2004; X.19.2004; X.22.2004; X.24.2004; X.252004. at uv light, B.D. & B.S. Valentine (BDVC): X.12.2005; X.17.2005. uv, B.D. & B.S. V. (BDVC): X.7.2005; X.9.2005; X.12.2005; X.13.2005; X.14.2005. **not located.** Quail Dove, malaise trap, V.2008 (BDVC). **Little Tobago.** VII.4.1966 (USNM). **Marina Cay.** VII.5.1988, S.E. Miller, C. O’Connell (BPBM). **Salt Island.** V.24.1966 (USNM). **Tortola.** East end, Queen Elizabeth Bridge, under bark, X.23.1992, M.A. Ivie (WIBF). **Virgin Gorda.** Coppermine Pt., under rocks, VII.18.1994, M.A. Ivie, T.R. Hughes (WIBF). Pond Bay, 50 m, VII.10.1984, D. Ford et al (WIBF). Windy Hill, at light, XI.13.1992, M.A. Ivie (WIBF).

### *Discoderus cinctus* (Putzeys)

**GREATER ANTILLES. CUBA. Guantanamo.** Guantanamo: VII. 27.1913, C.T. Ramsden (AMNH); IV.14.1919, C.T. Ramsden (MCZC); X.15.1934, M.M. Saylor (CASC). Guantanamo, under cow dung, Manati, IV.14.1919, C.T. Ramsden (MCZC). Guantanamo Bay, Beach Housing Area, UV light, II.24-27.1972, S Calhoun (FSCA). Guantanamo Bay, Center Bargo, uv, S. Calhoun (FSCA): VI.19-22.1972; VIII.3.1972. Guantanamo Bay, Center Bargo, uv, S. Calhoun (UASM): VI.22.1972; VIII.3.1972. Guantanamo Bay, Kittery Beach Housing Area, uv light, III.29-30.1972, S. Calhoun (FSCA). Guantanamo Bay Navy Base, Caravella Point, uv, S. Calhoun (FSCA): X.19.1972; X.27.1972; X.30.1972; XI.6.1972; XI.8.1972; XI.15.1972; XI.20.1972. Guantanamo Bay Navy Base, Caravella Point, uv, S. Calhoun (UASM): XI.8.1972; XI.20.1972. Guantanamo Bay Navy Base, Kittery Housing Area, uv, S. Calhoun: V.8-11.1972 (FSCA); V.15-18.1972 (FSCA); V.15-18.1972 (UASM). Imias, VIII.1975, L.F. Armas (IZAC). Imias, Est. Exp. Agronomica, VI.1961 (IZAC).

### *Discoderus cyaneopacus* (Darlington)

**GREATER ANTILLES. HISPANIOLA: DOMINICAN REPUBLIC. Independcia.** 3 km up road from Descubierta to Los Pinos, blacklighting, VII.15.2004, S.W. Lingafelter (USNM). 4 km S Los Pinos Loma de Vientos, 455 m, 18°35'N, 71°46'W, semiarid forest with pastures, VII.231992, Davidson et al (CMNH). **Monte Cristi.** Monte Cristi, VII.1937, Clench (MCZC). **HISPANIOLA: HAITI. Nord-Ouest.** Jean Rabel, II.1929, E.C. & G.M. Leonard (USNM). **OUEST.** Port-au-Prince, VIII.1924, G.N. Wolcott (USNM).

### *Discoderus thoracicus* (Putzeys)

**GREATER ANTILLES. CUBA. [not mapped.** F.C. Bowditch (MCZC), labelled: “loc. doubtful by P.J.D.”]. **HISPANIOLA: DOMINICAN REPUBLIC. Barahona.** 5 km W Barahona Agricultural Expt. Stn., uv trap, VI.9-30.1978, Woodruff et al (FSCA). **Indenpendencia.** 4.8 km up road from La Descubierta to Los Pinos, blacklighting, VII.15.2004, S.W. Lingafelter (USNM). **Monte Cristi.** 5 km NNE Bontoncillo, 50 m, 19°45'N, 71°42'W, arid thornscrub, XI.29-301992, Davidson, Klingler et al (CMNH). Vasquez, VI.19.1927, S.T. Danforth (MCZC). 3 km N Villa Elisa, uv, Woodruff & Stange (FSCA), (UASM): X.1.1983; X.1.1985. 4.8 km N Villa Elisa, mv & bl l, V.31.1994, R. Turnbow (UASM). 5 km N Villa Elisa: V.31.1994, R Turnbow (UASM); V.10-18.1985, E. Giesbert (FSCA). 8.2 km N Villa Elisa, mv & bl l, VI.1.1994, R. Turnbow (UASM). **Pedernales.** Cabo Rojo, XI.4.1986, W.J. Pulawski (CASC). Cabo Rojo, 17-55N 71-39W, 10 m, VII.27.1990, Rawlins, Young, Thompson (CMNH). Cabo Rojo, at light, 0-10 m, M. Ivie, Philips, Johnson (WIBF): VIII.24.1988; IX.8-9.1988; IX.10.1988. Cabo Rojo, in pool & at light, 0-10 m, M. Ivie, Philips, Johnson (WIBF): VIII.18-23.1988; VIII.24-28.1988; VIII.28-29.1988; IX.8-9.1988. Cabo Rojo, sea level, 19°55'N, 71°39'W, edge of salt marsh, X.21.1991, C. Young, S. Thompson et al (CMNH). Cabo Rojo, dead in light fixture, IX.10.1988, M. Ivie, Philips, Johnson (WIBF). Cabo Rojo, Alcoa Headquarters, blacklight trap, VI.20-24.1999, R. Woodruff, R. Baranowski (FSCA). 0.5 km N Cabo Rojo, 18°00'N, 71°39'W, uv light, 42.7 m, VII.10.1993, D.S. Sikes, R.P. Rosenfeld (WIBF). 9.5 km N Cabo Rojo, 18°00.042'N, 71°38.793'W, lights and beating, 33 m, VIII.8.1993, M.A. Ivie, K.A. Guerrero (WIBF). 9.6 km N Cabo Rojo, 18°00'N, 71°39'W, at light, 42m, VII.10.1993, D.S. Sikes, R.P. Rosenfeld (WIBF). 14 km N Cabo Rojo, 150 m., thorn scrub trop. dry forest, VIII.19.1988, M Ivie, Philips, Johnson (WIBF). 14.5 km N Cabo Rojo, 165m, 18°03'N, 71°39'W, arid thorn scrub, IX.26-27.1991, Davidson, Young et al (CMNH). **Puerto Plata.** Los Hidalgos, VI.4-5.1969, Flint & Gomez (USNM). **San Juan.** Rio Mijo, uv, V.20.1985, Woodruff & Stange (FSCA), (UASM). **HISPANIOLA: HAITI. Artibonite.** source Matelas, IX.5.1934, Darlington (MCZC). St. Marc, W.M. Mann (AMNH), (MCZC), (UASM). **Ouest.** Cape Haitien, W.M. Mann (MCZC). Manneville, W.M. Mann (MCZC). Port-au-Prince. Thor (suburb), P. Daniels res., uv, X.10-12.1970, J.E. Porter (FSCA). **Nord-Ouest.** Tortue I., Bassin Bleu, IV.1929, E.C. & G.M. Leonard (USNM).
